# Clustered Core- and Pan-Genome Content on *Rhodobacteraceae* Chromosomes

**DOI:** 10.1093/gbe/evz138

**Published:** 2019-07-03

**Authors:** Karel Kopejtka, Yan Lin, Markéta Jakubovičová, Michal Koblížek, Jürgen Tomasch

**Affiliations:** 1 Laboratory of Anoxygenic Phototrophs, Center Algatech, Institute of Microbiology CAS, Třeboň, Czech Republic; 2 Faculty of Science, University of South Bohemia, České Budějovice, Czech Republic; 3 Department of Physics, School of Science, Tianjin University, China; 4 SynBio Research Platform, Collaborative Innovation Center of Chemical Science and Engineering, Tianjin, China; 5 Faculty of Information Technology, Czech Technical University in Prague, Czech Republic; 6 Department of Molecular Bacteriology, Helmholtz Centre for Infection Research, Braunschweig, Germany

**Keywords:** genome architecture, genome evolution, origin of replication, *Rhodobacteraceae*

## Abstract

In Bacteria, chromosome replication starts at a single origin of replication and proceeds on both replichores. Due to its asymmetric nature, replication influences chromosome structure and gene organization, mutation rate, and expression. To date, little is known about the distribution of highly conserved genes over the bacterial chromosome. Here, we used a set of 101 fully sequenced *Rhodobacteraceae* representatives to analyze the relationship between conservation of genes within this family and their distance from the origin of replication. Twenty-two of the analyzed species had core genes clustered significantly closer to the origin of replication with representatives of the genus *Celeribacter* being the most apparent example. Interestingly, there were also eight species with the opposite organization. In particular, *Rhodobaca barguzinensis* and *Loktanella vestfoldensis* showed a significant increase of core genes with distance from the origin of replication. The uneven distribution of low-conserved regions is in particular pronounced for genomes in which the halves of one replichore differ in their conserved gene content. Phage integration and horizontal gene transfer partially explain the scattered nature of *Rhodobacteraceae* genomes. Our findings lay the foundation for a better understanding of bacterial genome evolution and the role of replication therein.

## Introduction

Replication is assumed to be a key factor in the evolution of genome structure and organization (Rocha 2004; Rocha 2008; Cagliero et al. 2013; Jun et al. 2018). In contrast to eukaryotes and archaea, where the chromosome replication proceeds simultaneously from multiple sites, replication of bacterial chromosomes starts from a single origin of replication (*oriC*) and continues equally along both replichores (two halves of the chromosome extending from *oriC*) up to the terminus of replication (*terC*). Since cell division is often shorter than the time required for the replication of the chromosome itself, it leads to the occurrence of multiple replication complexes in the cell. In result, the genes located in the early replicating regions near the *oriC* can be present in multiple copies and hence have a higher expression level compared with genes in the late replicating regions. This so-called gene-dosage effect is especially pronounced in fast-growing bacteria, where strongly expressed genes are preferentially concentrated near the *oriC* (Rocha 2004; Couturier and Rocha 2006). Bacterial chromosome architecture can also be shaped by large-scale interreplichore translocations, as was recently shown using genome sequence comparisons between 262 closely related pairs of bacterial species (Khedkar and Seshasayee 2016).

Relative distance of genes from the *oriC* is commonly thought to be one of the most conserved properties of genome organization (Eisen et al. 2000; Sobetzko et al. 2012). Results of an extensive analysis comprising a set of 131 gammaproteobacterial genomes showed strong conservation in the relative distance of conserved genes coding for regulatory elements from the *oriC* (Sobetzko et al. 2012).

A replication-biased genome organization was also revealed in archaeal genomes. Flynn et al. (2010) selected six *Sulfolobus* genomes each with multiple replication origins, to test the hypothesis that genes situated close to *oriC* tend to be more conserved than genes more distant from *oriC*. Results of this study clearly demonstrated a bias in the location of conserved orthologous genes (orthologs) toward the *oriC*. Moreover, the analysis of evolutionary rates of these orthologs revealed their slower evolution when compared with genes more distant from *oriC* (Flynn et al. 2010). Another study of *Sulfolobus* genome architecture showed a mosaic of recombinant single nucleotide polymorphisms along the chromosomes of ten closely related *Sulfolobus islandicus* strains. This comparative genome analysis revealed large genomic regions surrounding all *oriC* sites that show reduced recombination rates (Krause et al. 2014).

Based on an analysis of the codon composition of genomes from 59 prokaryotic organisms, it was shown that genes organized close to the *ter* are in many cases A + T-enriched at the third codon position thought to reflect a higher evolutionary rate (Daubin and Perriere 2003). In addition, horizontally transferred DNA was suggested to cluster near the *terC* (Rocha 2004); this was documented in the genome of *Escherichia coli* (Lawrence and Ochman 1998), as well as in other prokaryotic genomes (Touchon and Rocha 2016). Early studies presenting complete genome sequences of *Bacillus subtilis* (Kunst et al. 1997) and *E. coli* (Blattner et al. 1997; Lawrence and Ochman 1998) reported a frequent occurrence of prophages around the *terC*. Furthermore, a recent study conducted on a large and diverse sample of bacterial species revealed a positive bias in the occurrence of hot-spots for HGT containing prophages toward the *terC* (Oliveira et al. 2017). In addition, it has been speculated that the gene-dosage effect might lead to fixing of typically weakly expressed (or not at all) horizontally transferred genes closer to the *terC* (Rocha 2004).

To date, there is no comprehensive study on the distribution of conserved genes in the bacterial chromosome solely focusing on one bacterial family. We therefore decided to analyze the relationship between the degree of gene conservation and its distance from the origin of replication for the core- and pan-genome of all *Rhodobacteraceae* (Alphaproteobacteria) with closed genomes. All the gene families present in a certain (microbial) clade are the pan-genome. The gene families with representatives present in genomes of all strains are defined as core genome, whereas the term accessory genome describes partially shared gene families and strain specific genes (Medini et al. 2005; Tettelin et al. 2005). *Rhodobacteraceae* were selected as a family with dynamic evolution, and a large number of sequenced genomes. The data set comprises 109 species originating from diverse habitats as soil, freshwater, marine, and hypersaline environment (Simon et al. 2017). The frequent occurrence of plasmids (Petersen et al. 2013), transposable elements (Vollmers et al. 2013), and gene-transfer agents (Shakya et al. 2017) suggest that HGT plays an important role in shaping the genomes of this family.

## Materials and Methods

### Data

**Fig. 1.**
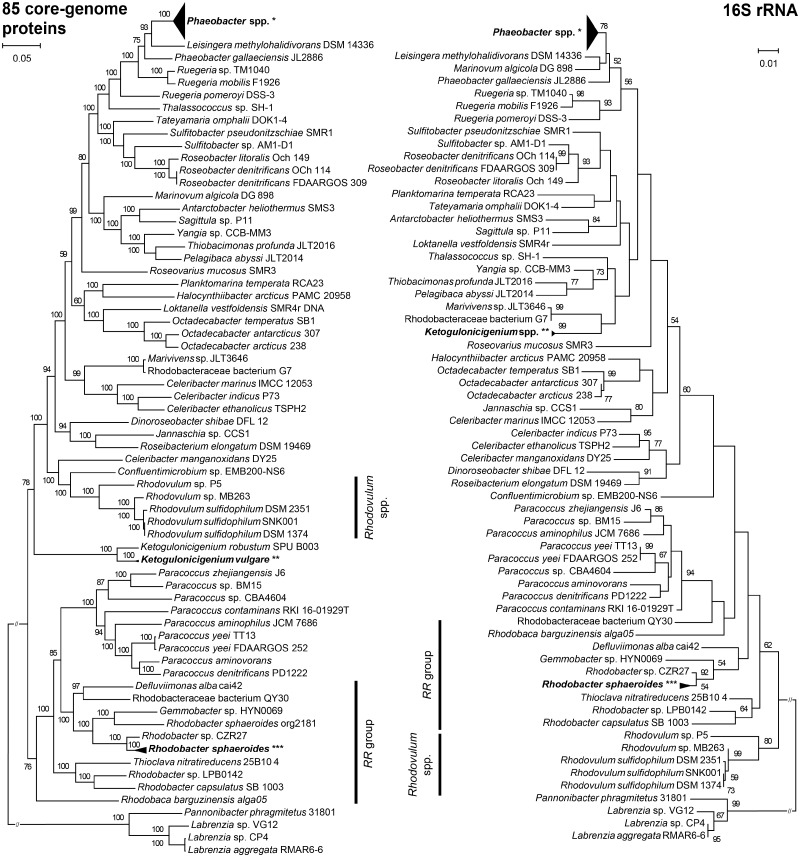
—Comparison of phylogenomic and 16S rRNA trees. Both trees comprise the same set of 109 *Rhodobacteraceae* strains. *Pannonibacter phragmitetus* 31801, *Labrenzia* sp. VG12, *Labrenzia* sp. CP4, and *Labrenzia aggregata* RMAR6-6 were used to root the trees as outgroup species. Scale bars represent changes per position. Bootstrap values >50% are shown. Bold vertical bars refer to different clustering patterns of the *Rhodovulum* spp. and *RR* (*Rhodobacter*-*Rhodobaca*) group inside both trees. *Collapsed *Phaeobacter* (*P.*) branches involve species *Phaeobacter gallaciensis* (strains DSM 26640, P11, P63, P73, P75, P128, and P129), *Phaeobacter inhibens* (strains 2.10, DOK1-1, DSM 17395, P10, P24, P30, P48, P51, P54, P57, P59, P66, P70, P72, P74, P78, P80, P83, P88, and P92), *Phaeobacter piscinae* (strains P13, P14, P18, P23, P36, P42, and P71), and *Phaeobacter porticola* P97; **Collapsed *Ketogulonicigenium vulgare* branches involve strains Hbe602, SKV, SPU B805, WSH-001, and Y25; ***Collapsed *Rhodobacter sphaeroides* branches involve strains ATCC 17025, ATCC 17029, MBTLJ-8, MBTLJ-13, MBTLJ-20, and KD131 in both trees with additional strain org2181 in the 16S rRNA tree. Maximum-likelihood (ML) tree (left panel) based on concatenated alignments of amino acid sequences of the 85 highly conserved core-genome proteins (27,668 common amino acid positions). Amino acid sequences were identified using Proteinortho with cut-off criteria of e-value ≤1e-10, sequence identity ≥ 60%, and sequence coverage ≥ 80%. The ML tree was calculated with 100 bootstrap replicates. 16S rRNA phylogenetic tree (right panel). Nucleotide sequences were aligned using ClustalX version 2.1 resulting in alignment with 1,260 common nucleotide positions after applying G-blocks. The phylogenetic tree was inferred using the ML algorithm with the GTR nucleotide substitution model and 1,000 bootstrap replicates. When possible, the strains were listed in the same order as in the phylogenomic tree.

Nucleotide genomic sequences and corresponding Genbank FASTA files for 109 fully sequenced *Rhodobacteraceae* strains (Fig. 1) were obtained from NCBI GenBank (May 2018). 16S rRNA gene sequences for the same set of strains were obtained either from the SILVA database (Quast et al. 2012) or NCBI GenBank (May, 2018).

### Software

The programs and packages used in our analysis are summarized in the supplementary table S1, Supplementary Material online. Commands lines for Prokka and ProteinOrtho can be found in supplementary table S2, Supplementary Material online. Custom scripts as well as the obtained pan-genome data sets are available from the authors upon request.

### Comparative Genomic Analysis

In order to standardize further analysis, we reannotated all genomes using Prokka (Seemann 2014). Orthologous gene cluster analysis was performed using the Proteinortho dataframe (Lechner et al. 2011). As in previous comparative studies (Kalhöfer et al. 2011; Thole et al. 2012; Vollmers et al. 2013), orthologous protein sequences were identified with three cut-off criteria: 1) e-value, 2) alignment coverage, and 3) sequence identity. Three pan-genome data sets (pan60, pan30, and pan15) were produced that differ in stringency of ortholog identification. Cut-off criteria as well as the number of identified protein families and core genome are summarized in table 1.

**Table 1:** Characteristics of Pan-Genome Data Sets Used in This Study

Data Set	e*-*Value	Minimum Sequence Coverage	Minimum Identity	Number of Protein Families	Core Protein Families (including paralogs)	Soft-Core^a^ Protein Families (including paralogs)	Core Protein Families (no paralogs)	Core Protein Families (no paralogs and connectivity >0.9)
pan60	10^−10^	80	60	37,326	161	499	141	85
pan30	10^−10^	70	30	25,143	464	911	411	352
pan15	10^−05^	70	15	24,317	479	936	422	362

^a^Soft-Core is defined as protein families found in 95% of the strains (104 out of 109).

### Phylogenomic Analysis

Since for highly conserved proteins with >50% sequence identity the probability of completely incorrect annotation is very low (<6%) (Sangar et al. 2007), we used the core genome identified with the most stringent parameters (pan60 data set: e-value <10^−10^, 80% sequence coverage and 60% sequence identity) for constructing the phylogenomic tree. Furthermore, we excluded all protein families that contained paralogs in one or more of the genomes. As a last step, we excluded all protein families with a Proteinortho connectivity score <0.9. Amino acid sequences for these 85 highly conserved core genome proteins were individually aligned using ClustalX version 2.1. Sites containing gaps and ambiguously aligned regions were removed from each alignment using Gblocks (Talavera and Castresana 2007) and finally these alignments were concatenated with Geneious version 8.1.2 (Biomatters Ltd.). The phylogenomic tree was inferred by MEGA 6.0 software using the maximum likelihood (ML) algorithm with LG model (Le and Gascuel 2008) that has been used for this bacterial family before (Simon et al. 2017). For statistical support, 100 bootstrap replicates were employed. 16S rRNA gene sequences for the same set of strains as in phylogenomic tree were aligned using ClustalX version 2.1, ambiguously aligned regions and gaps were excluded from the alignment using Gblocks. The 16S rRNA tree was constructed by PhyML/MEGA 6.0 software using the ML algorithm with GTR nucleotide substitution model (Koblížek et al. 2015) and 1,000 bootstrap replicates. The four strains of the deep branching “*Stappia* group” (Pujalte et al. 2014), that is, *Pannonibacter phragmitetus* 31801, *Labrenzia* sp. VG12, *L*. sp. CP4, and *Labrenzia aggregata* RMAR6-6, were used as outgroup organisms to root both trees.

### Identification of *oriC*

The origin of replication (*oriC*) of studied strains were identified using Ori-Finder (Gao and Zhang 2008; Luo et al. 2018), which was developed mainly based on the analysis of nucleotide composition asymmetry using the Z-curve approach and the distribution of DnaA boxes. Three different DnaA box motives (i.e., TTATCCACA, TGTTTCACG, and TGTGGATAT) were used during the search. Typically, the *E. coli* perfect DnaA box (TTATCCACA) is the most used motive for regular prediction (Mackiewicz et al. 2004). When only one unmatched site was allowed, the *oriC*s of a few genomes could not be identified. Whereas, a number of alternative *oriC*s were predicted when we allowed two unmatched sites. The output of Ori-Finder (examples are shown in supplementary fig. S1, Supplementary Material online) was manually curated. When a number of alternative *oriC*s were predicted, we decided which one most likely was the right one considering these criteria: 1) proximity to the GC disparity minimum, 2) location within a local GC minimum, and 3) proximity to the *parAB* genes. Within the pan15 data set, all analyzed strains harbored only one *parA* gene. Next to *parA*, *parB* was identified in all strains except for *Paracoccus yeei* TT13.

### Identification of Phages and Horizontally Transferred Genes

The web-based PHAge Search Tool—Enhanced Release (PHASTER) was used for predicting prophage sequences or remnants of those (Arndt 2016). For prediction of genomic islands (GIs), we used AlienHunter (Vernikos and Parkhill 2006) and the web-based tool IslandViewer (Bertelli et al. 2017). AlienHunter predicts GIs using Interpolated Variable Order Motifs (IVOMs). This approach exploits compositional biases by determining variable order motif distributions. IslandViewer integrates three different GI prediction tools: IslandPath-DIMOB (Hsiao et al. 2003), SIGI-HMM (Waack et al. 2006), and IslandPick (Langille et al. 2008). All GIs predicted by at least one method were considered for further analysis.

### Statistical Analysis

Analysis was performed for all three pan-genome data sets (pan15, pan30, and pan60): For each gene of each strain the number of strains with at least one ortholog to the respective gene was obtained. For simplicity, we named this number the ortholog score. Next, we calculated the mean ortholog score as well as the midpoint distance to *oriC* for sliding windows of 20 genes. Linear and quadratic models were fitted to the data. For the linear models, slope and corresponding *P* value were extracted. In order to account for the overrepresentation of several genera, only one strain per genus was selected from the pan15 data set and the same analysis as described earlier was performed. The resulting slope values were compared with the slope values of the full data set.

Furthermore, the chromosomes were separated into eight equally sized segments and the mean and SD for the ortholog scores of the genes in these segments was calculated. The number of phage regions and GIs were calculated for three parts with increasing distance from *oriC*. The distribution of the number of loci was visualized using boxplots. Analysis of variance (ANOVA) was used to test for significant differences between the eight chromosomal segments as well as the three parts used for Phage and HGT analysis. Tukey’s test was used to identify the segments and parts with significant differences in the ortholog score as well as the number of phages and HGT regions, respectively.

## Results and Discussion

### Phylogenomic Analysis

To show the overall picture of phylogenetic relationships between the studied strains, we constructed a phylogenomic species tree and 16S rRNA tree as a reference. For given cut-off values (pan60: e-value ≤1e-10, sequence identity ≥ 60%, sequence coverage ≥ 80%, no paralogs, connectivity >0.9), analysis of the selected *Rhodobacteraceae* genomes identified a core genome of 85 protein families. These were used to construct a robust phylogenomic tree (fig. 1, left). The obtained tree had good statistical support and also agreed well with other recent phylogenomic studies of this family (Simon et al. 2017; Brinkmann et al. 2018). The 16S rRNA phylogenetic tree (fig. 1, right), which was based on an alignment with 1,260 common nucleotide positions, shows a mosaic branching pattern with considerably lower statistical support when compared with the phylogenomic tree. The most striking difference between both methods was in the clustering of the *Rhodovulum* species. In the phylogenomic tree, these strains clearly clustered with the Roseobacter group (Simon et al. 2017), whereas in the 16S rRNA tree they were placed close to the *Rhodobacter*/*Rhodobaca* (*RR*) group, as we found before (Kopejtka et al. 2017; Kopejtka et al. 2018). The selected strains represent the full spectrum of *Rhodobacteraceae* from various environments (supplementary table S3, Supplementary Material online) although marine Roseobacter species and in particular, the genus *Phaeobacter* (Freese et al. 2017) are overrepresented.

### Identification of *oriC* Locus

We started the data analysis by assigning to each genome the coordinates of its *oriC* region. After manual curation of the predictions made by Ori-Finder, we were able to clearly pinpoint the *oriC* for 101 strains. Due to missing overrepresentation of DnaA boxes and/or lack of distinct differences in nucleotide composition compared with the rest of the genome, we could not unambiguously identify the *oriC* of eight strains, which we excluded from further analysis (supplementary table S3, Supplementary Material online).

### Location of Conserved Genes in Relation to *oriC*

**Fig. 2.**
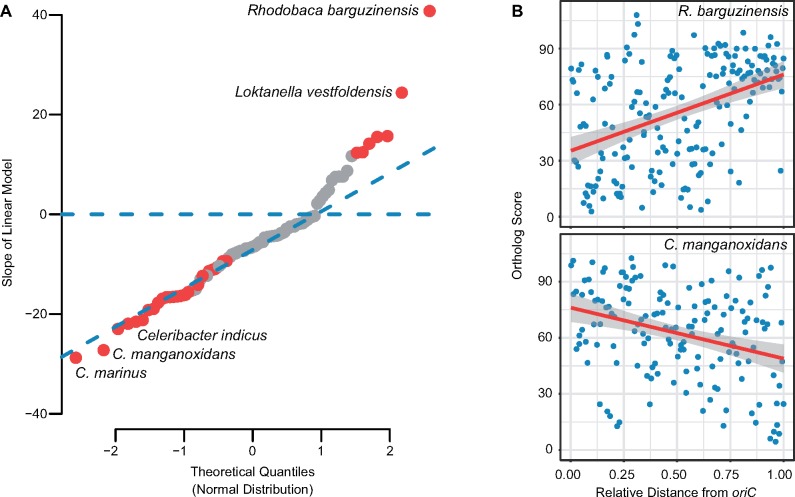
—Gradient in number of conserved genes with increasing distance from *oriC*. Analysis is based on the pan15 data set. A linear model was fitted for the average ortholog score within sliding windows of 20 genes in relation to the midpoint distance of the sliding window to *oriC* for each strain. (*A*) Quantil–quantil plot comparing the slope values extracted from the linear model of each genome to a theoretical normal distribution. Increasing slope values reflect the increase in ortholog score with increasing distance from *oriC*. Deviations from the normal distribution are indicated by increasing distance from the sloped blue dashed line. The horizontal blue dashed line highlights the coordinate on the *y* axis where the slope value is equal to 0. Red dots represent strains with slope values significantly different from 0 (*P* < 0.05). Names of the strains with the highest negative and positive slope values are shown. These strains represent groups with different genome architecture. (*B*) Average ortholog score compared with distance from *oriC* for *Rhodobaca barguzinensis* (upper panel) and *Celeribacter manganoxidans* (lower panel). The linear function (red line) fitted to the data showed a significant increase (upper panel) or decline (lower panel) in average ortholog score with increasing relative distance from *oriC.* Results for all three data sets (pan15, pan30, pan60) are shown in supplementary figures S2–S5, Supplementary Material online.

We analyzed each of the 101 genomes with identified *oriC* for a potential bias in localization of conserved genes along the chromosome. Therefore, we counted for each gene of each chromosome the number of strains with at least one ortholog to the respective gene. For simplicity, we refer to this number as ortholog score. We compared the average ortholog score within sliding windows of 20 genes to the midpoint distance of the sliding window to *oriC*. Next, we fitted linear and quadratic models of the ortholog score for increasing distances from *oriC* for all these 101 genomes and for all three pan-genome data sets (supplementary figs. S2–S4). We identified strains with statistically significant negative (i.e., average ortholog score decreasing with distance to *oriC*) or positive (i.e., average ortholog score increasing with distance to *oriC*) slope values of the linear model (fig. 2*A* andsupplementary table S4, Supplementary Material online). The obtained result was similar for all three pan-genome data sets used and for the remaining part of the analysis we focused on the pan15 data set—representing the most relaxed ortholog identification criteria. For this data set, our analysis yielded 35 strains with statistically significant negative or positive slope values (supplementary fig. S5 and table S4, Supplementary Material online). In some cases—in particular, for *Paracoccus* sp. CBA4604—the quadratic model showed a better fit than the linear model. This is an indication that for the chromosomes of these strains the ortholog score is on an average higher or lower for genes in the middle of the replichores rather than close to the *oriC* or *terC* regions (supplementary figs. S2–S4, Supplementary Material online). However, the quadratic models were not considered in further analysis. Reducing the data set to only one strain per genus yielded highly similar results (supplementary fig. S6, Supplementary Material online).

The majority of strains (83 out of 101) had a negative slope. Thus, the genomes showed a tendency toward having highly conserved genes clustered closer to *oriC.* While the negative slope values perfectly followed a normal distribution, the positive slope values were systematically higher than what would be expected from normally distributed data (fig. 2*A*). We then specifically focused on the strains from genus *Celeribacter* (*C.*), with the lowest slope values indicating a significant increase (fig. 2*B*, upper panel) in ortholog scores with increasing distance from *oriC.* At the other extreme, we focused on *Rhodobaca* (*R.*) *barguzinensis* and *Loktanella* (*L.*) *vestfoldensis*, the species with the highest slopes and the conserved genes mostly clustered around *terC* (fig. 2*B*, lower panel).

### Clustering of Core- and Pan-Genome Content in Representative *Rhodobacteraceae*

We created chromosome maps for all *Rhodobacteraceae* with identified *oriC* (supplementary fig. S7, Supplementary Material online) and particularly focused on the five strains representing two different extremes of chromosome architecture. The strain with the most negative slope value, *Celeribactermarinus*, showed a conspicuous switch in GC-skew within the right replichore (supplementary fig. S8, Supplementary Material online). This indicates either a recent genomic inversion event or a misassembled genome. Thus, this genome is not further discussed.

**Fig. 3.**
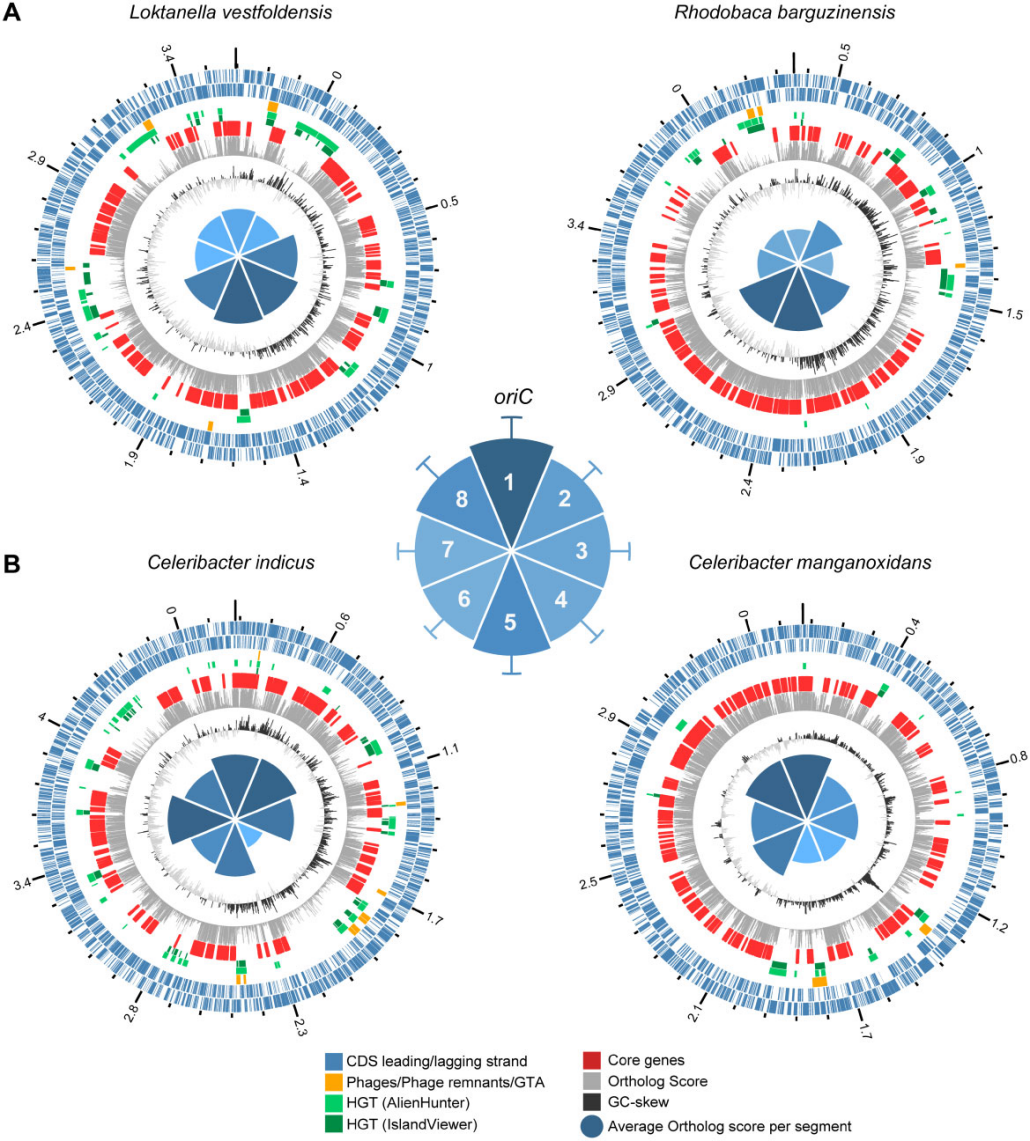
—Chromosome plots of four strains representing two different kinds of chromosome architecture in *Rhodobacteraceae*. (*A*) Two representatives from the group of strains for which the ortholog score increases with the distance from the origin of replication. (*B*) Two representatives from the major group of strains for which the ortholog score decreases with the distance from the origin of replication. The outer to inner rings represent: scale of genome size in Mb and position of *oriC*; position of ORFs encoded on the plus strand; position of ORFs encoded on the minus strand; groups of HT genes as defined in the graphical legend below; position of core genes with orthologs in all 108 *Rhodobacteraceae* strains; barchart displaying the ortholog score of each representative’s genes; GC-skew; polar plot showing the average ortholog score in each of eight segments. Polar plot in the middle: average ortholog score in each segment calculated as an average for all strains; the darker the shade of blue the higher the number. See supplementary figure S6, Supplementary Material online, for Tukey’s HSD test for the eight segments. Orthologs were identified using Proteinortho with cut-off criteria of e-value ≤1e-05, sequence identity ≥ 15%, and sequence coverage ≥ 70% (pan15 data set).

The four remaining analyzed strains, *L. vestfoldensis* and *R. barguzinensis*, *Celeribacterindicus*, and *Celeribactermanganoxidans*, showed highly conserved regions in which the core genes (with orthologs in all other strains) clustered, interrupted by regions of genes with orthologs in only a small number or even no other strains (fig. 3). However, besides the distance to *oriC*, the distribution of conserved genes also varied between both replichores. We separated the chromosome into eight equally sized segments and calculated the average ortholog score for each segment (segment 1 surrounding *oriC* and counting proceeding clockwise). On an average, the ortholog score was higher in two opposing segments surrounding *oriC* and *terC* (fig. 3, center panel). Only segment 1 showed a significant enrichment in conserved genes compared with all others (supplementary fig. S9, Supplementary Material online).

*Loktanella* *vestfoldensis* and *R. barguzinensis* both showed highly conserved and core genes concentrated around *terC* and bigger regions of low conservation around *oriC* (fig. 3*A*). The *L. vestfoldensis* genome showed differences between segments with higher average of conserved genes in the segments 3–6, thus around *terC* and on the right replichore. In contrast, the genome *R. barguzinensis* showed a concentration of highly conserved and core genes in the segments 4–6, while in particular, the segments 1, 3, 7, and 8 contained large stretches of regions with only few conserved orthologs in the genomes of other strains. Thus, the increase of conserved genes toward *terC* can be attributed to large regions of low conservation on both replichores.

*Celeribacter* *indicus* and *C. manganoxidans* both showed a tendency of clustering highly conserved and core genes toward *oriC* (fig. 3*B*). However, in both cases, the distribution of highly conserved and core genes followed a more complex pattern. The genome of *C. indicus* showed a pronounced mosaic pattern with alternating regions of core and accessory genes. The core genes were concentrated around *oriC* (segments 1 and 2), *terC* (segment 5), and segments 3 and 6 in the middle of both replichores. A huge region of low conservation is found in segment 4. The *C. manganoxidans* genome shows a concentration of core genes on the left replichore, with low conserved regions concentrated in segments 2–5. Thus, for both genomes, the decrease of conserved genes toward *terC* was the result of uneven distribution of highly conserved (core) and accessory genes between replichores.

### Influence of Phages and HGT on Architecture of *Rhodobacteraceae* Genomes

**Fig. 4.**
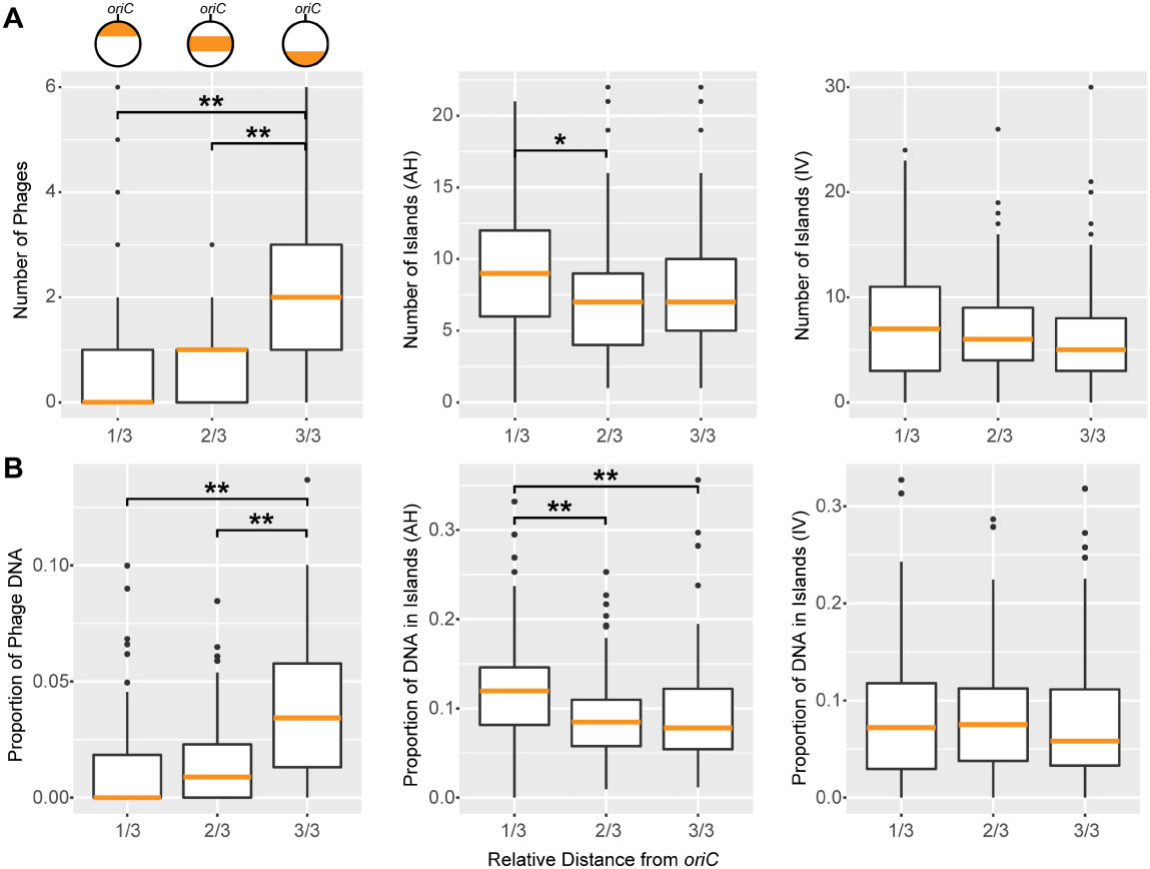
—Distribution of HT genes along the chromosome in 101 *Rhodobacteraceae*. (*A*) Mean numbers of phage regions identified by Phaster (phages, left panel), Genomic Islands identified by AlienHunter (AH, middle panel), and IslandViewer (IV, right panel) were calculated for each third of the chromosome. (*B*) Proportion of DNA found in phages or genomic islands, panel order as in (*A*). The orange horizontal lines represent median values. ANOVA was used to test for significant differences between the three parts of the chromosome. Asterisks indicate significant differences between comparisons identified using Tukey’s HSD test (*P < 0.05, **P < 0.01).

We identified prophage sequences and regions putatively acquired through horizontal gene transfer and compared those to the clustering of core and accessory genes. The strains with the core genes shifted more toward the *ter* region had phages integrated near *oriC* (fig. 3*A*).The strains with the core genes more shifted toward *oriC* had phages integrated near *terC* (fig. 3*B*). Regarding all analyzed strains, there was a significant enrichment in the absolute number of phages and the proportion of phage DNA near *terC* (fig. 4), confirming previous results (Oliveira et al. 2017).

Both methods used for HGT identification found overlapping regions of putative foreign origin. GIs of *R. barguzinensis* and *C. manganoxidans* were always found closer to *oriC* and *terC*, respectively (fig. 3). GIs of *L. vestfoldensis* and *C. indicus* were scattered throughout the genome with no preference toward *oriC* or *ter* (fig. 3). Not all regions with low conservation were identified as horizontally transferred. In particular, only parts of the huge accessory genome regions in *R. barguzinens* (segments 3 and 8) and *C. indicus* (segment 4) contained identifiable GIs. Interestingly, on an average, the number of HGT regions was higher closer to *oriC* (fig. 4). However, a significant enrichment of HGT regions and the proportion of DNA within those were only found for the AlienHunter but not the IslandViewer results. In summary, phages and other sources of foreign DNA can only explain part of the observed core- and pan-genome clustering.

## Conclusion

Our comparative genomic analysis revealed an unexpected bias in the clustering of conserved genes along the *oriC* → *terC* replication axis in several *Rhodobacteraceae* representatives. We observed the general trend that the part of the genome closer to *oriC* contains on an average a higher density of core genes. This finding is in line with previous publications (Rocha 2004; Touchon and Rocha 2016; Oliveira et al. 2017). However, we also identified remarkable exceptions to this trend, namely *L. vestfoldensis* and *R. barguzinensis*. Further investigation of the strains with the distribution of core genes most biased regarding distance to *oriC* revealed complex patterns with conserved regions and core genes often clustered in one half of one replichore. The analysis was restricted to *Rhodobacteraceae* with closed genomes. However, this subset contains strains of different genera from various habitats. Furthermore, using a data set that contained only one strain per genus, we obtained highly similar results to the full data set in which some genera (in particular, *Phaeobacter*) were overrepresented. Therefore, we expect that the observed patterns will not change substantially when more genomes are included which would impact the core- and pan-genome content of the data set.

The forces that may have driven the evolution of the observed pattern in this prokaryotic family remain to be elucidated. Selective gene loss alone cannot explain the huge regions containing only accessory genes. The genome of the last common ancestor of the *Rhodobacteraceae* must be assumed unrealistically large to contain the pan-genome of this family (Dagan and Martin 2007). Gene loss and gene gain by HGT might both have contributed to the evolution of clustered genomes. Phages have also been identified closer to the origin in cases where conserved genes were clustered at the terminus, and vice versa. However, their presence does not explain longer stretches of the chromosomes with genes weakly conserved in *Rhodobacteraceae*. The role that replication might have played during evolution has to be investigated in greater detail. However, our data clearly shows, that a model of preferential integration of transferred genes and phages at the terminus of replication, for example, as compensation for dosage effects might not be generalizable. A comprehensive determination of the transcriptional landscape as well as growth rates and replication timing within this bacterial family might reveal characteristics of the exceptional strains that could help to explain their chromosome architecture.

## Supplementary Material

Supplementary data are available at *Genome Biology and Evolution* online.

## Supplementary Material

evz138_Supplementary_Data
