# Supplementary material for: Clustered Core- and Pan-Genome Content on Rhodobacteraceae Chromosomes
Source: Genome Biol Evol. 2019 Jul 3;11(8):2208–17. doi: 10.1093/gbe/evz138 (PMC6699656; doi:10.1093/gbe/evz138)
Supplement: evz138_Supplementary_Data [file evz138_supplementary_data.zip › Supplementary information.pdf]

Supplementary information

Clustered core- and pan-genome content on *Rhodobacteraceae* chromosomes

Karel Kopejtka[1,2], Yan Lin[3,4], Markéta Jakubovičová[5], Michal Koblížek[1,2], Jürgen Tomasch[6]*

[1]*Laboratory of Anoxygenic Phototrophs, Center Algatech, Institute of Microbiology CAS, Třeboň, Czech Republic*

[2]*Faculty of Science, University of South Bohemia, České Budějovice, Czech Republic*

[3]*Department of Physics, School of Science, Tianjin University, Tianjin, China*

[4]*SynBio Research Platform, Collaborative Innovation Center of Chemical Science and Engineering, Tianjin, China*

[5]*Faculty of Information Technology, Czech Technical University in Prague, Czech Republic*

[6]*Department of Molecular Bacteriology, Helmholtz Centre for Infection Research, Braunschweig, Germany*

**Author for correspondence: Juergen.Tomasch@helmholtz-hzi.de*

This file contains:

Legends for supplementary Figures S2, S3, S4, and S7 and Supplementary Tables S3 and S4

Supplementary Figures S1, S5, S6, S8, and S9 and Supplementary Tables S1 and S2

Supplementary Figures S2, S3, S4 and S7 are included as separate PDF files.

Supplementary Tables S3 and S4 are included as separate Excel files.

Supplementary Figure S2 – Gene conservation with increasing distance from *oriC* for all strains for dataset pan15. The average number of strains with orthologs to the respective genes (ortholog score) and distance to the origin of replication was calculated for sliding windows of 20 genes and a linear and a quadratic model were fitted. Confidence intervals of both fitted curves are shown. Pan-genome was determined using Proteinortho with cut-off criteria of e-value ≤ 1e-05, sequence identity ≥ 15%, and sequence coverage ≥ 70%.

Supplementary Figure S3 – Gene conservation with increasing distance from *oriC* for all strains for dataset pan30. The average number of strains with orthologs to the respective genes (ortholog score) and distance to the origin of replication was calculated for sliding windows of 20 genes and a linear and a quadratic model were fitted. Confidence intervals of both fitted curves are shown. Pan-genome was determined using Proteinortho with cut-off criteria of e-value ≤ 1e-10, sequence identity ≥ 30%, and sequence coverage ≥ 70%.

Supplementary Figure S4 – Gene conservation with increasing distance from *oriC* for all strains for dataset pan60. The average number of strains with orthologs to the respective genes (ortholog score) and distance to the origin of replication was calculated for sliding windows of 20 genes and a linear and a quadratic model were fitted. Confidence intervals of both fitted curves are shown. Pan-genome was determined using Proteinortho with cut-off criteria of e-value ≤ 1e-10, sequence identity ≥ 60%, and sequence coverage ≥ 80%.

Supplementary Figure S7 – Circular representation of all 101 *Rhodobacteraceae* chromosomes with identified *oriC*. The outer to inner rings represent: scale of genome size in Mb and origin of replication (*oriC*); position of ORFs encoded on the plus and minus strand; groups of HT genes as defined in Figure 2; position of genes with orthologs in all other 108 *Rhodobacteraceae* strains; barchart displaying the proportion of strains in which orthologs of

each representative's genes have been found (pan15 dataset); GC-skew. Note that chromosomes have not been reoriented towards the marked *oriC*.

Supplementary Table S3 – Accession numbers and characteristics of the 109 strains used for core- and pangenome analysis. Habitats are abbreviated: water (w), soil (s), clinical (c), freshwater (f) and marine (m). The location of identified *oriC*s is indicated.

Supplementary Table S4 – Location of *oriC* and results of linear models for the 101 strains with identified *oriC*. Linear models of the average ortholog score on the midpoint distance to *oriC* within sliding windows of 20 genes were determined. Results (slope, intercept, p-value) for all three pan-genome datasets used (pan15, pan30, and pan60) and the dataset with one strain of each genus are included.

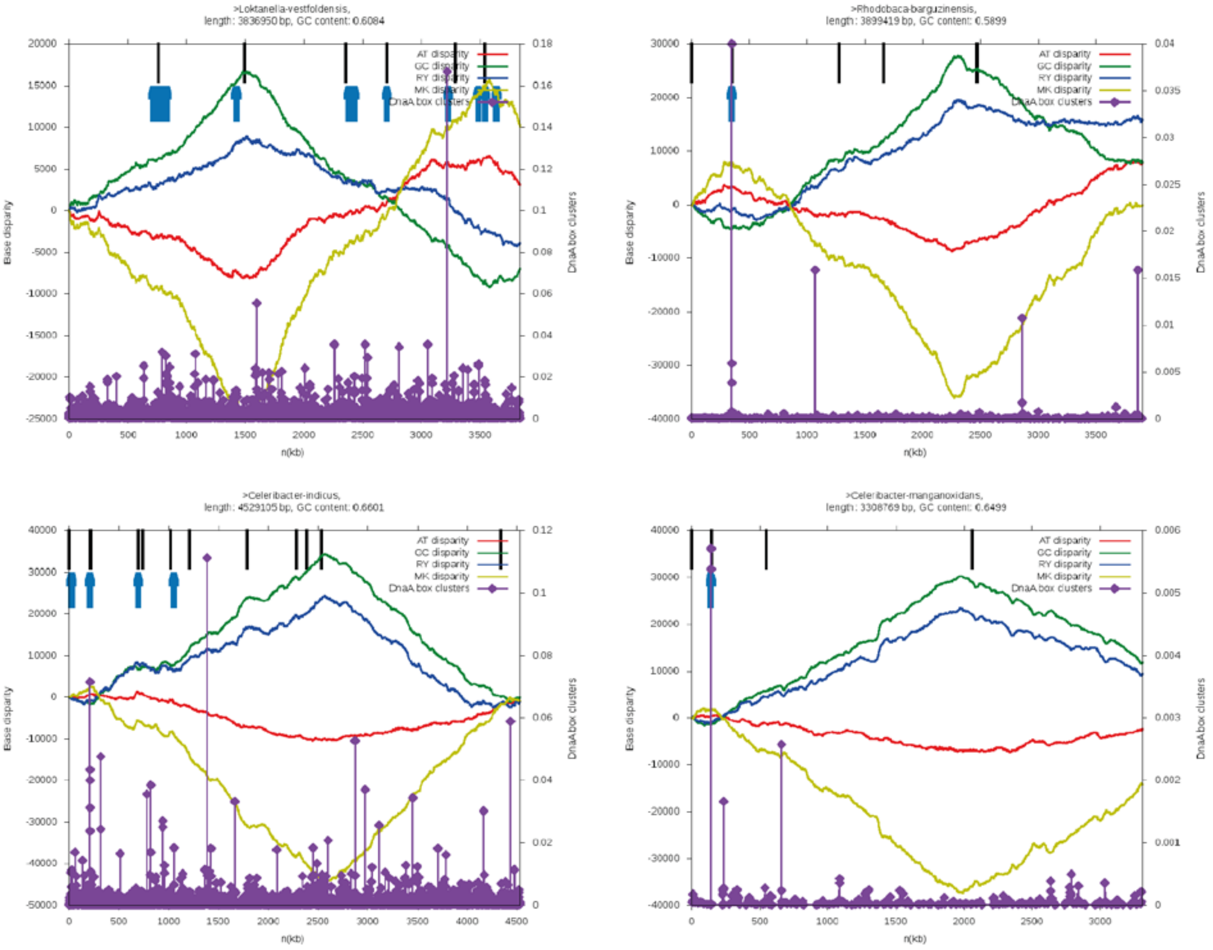

Supplementary Figure S1 – Representative output of the Ori-Finder analysis. Base disparites and enrichment of DnaA boxes are shown. Putative oriCs are indicated by blue arrows.

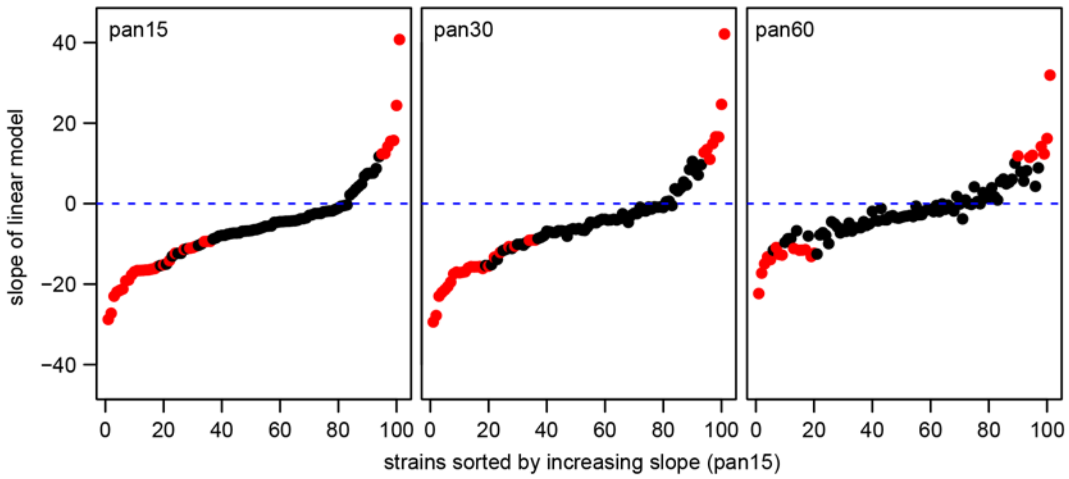

Supplementary Figure S5 – Slopes of the linear models for all strains. Results for all three pan-genome datasets used (pan15, pan30, and pan60) are shown. The average ortholog score and distance to the origin of replication was calculated for sliding windows of 20 genes. A linear model was fitted and the slope value extracted. Red dots represent strains with slope values significantly different from 0 ($p < 0.05$).

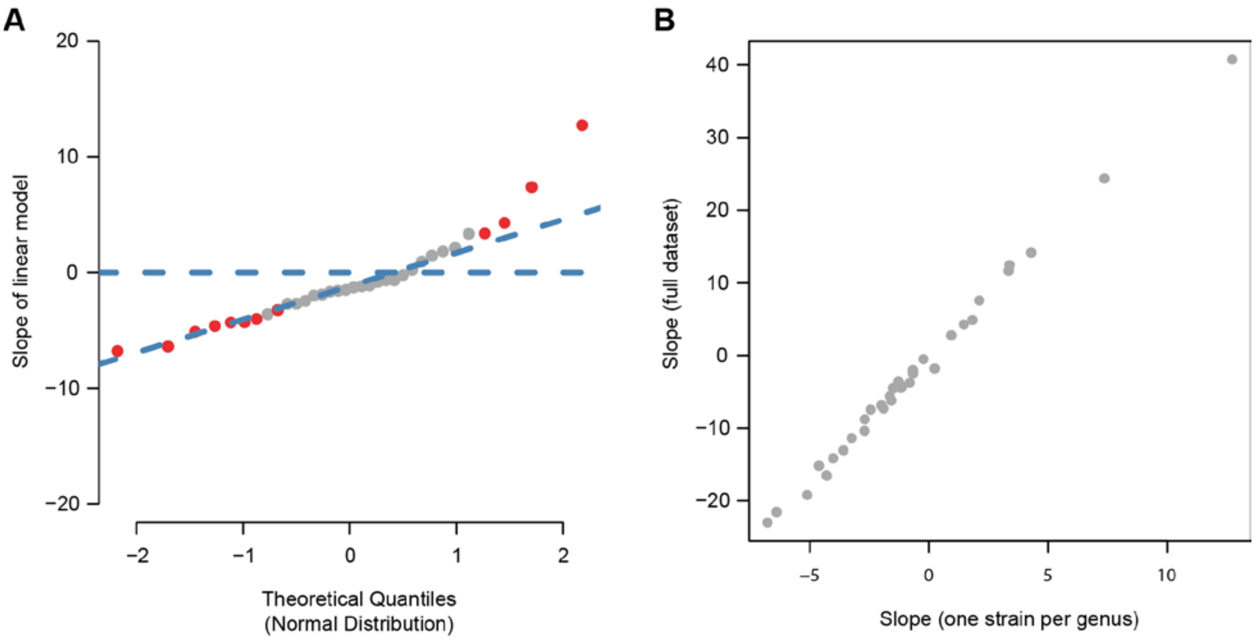

Supplementary Figure S6 – Slopes of linear models derived from a dataset with one strain of each genus. A linear model was fitted for the average ortholog score within sliding windows of 20 genes in relation to the midpoint distance of the sliding window to oriC for one strain per genus (34 strains in total). **(A)** Quantil-quantil plot comparing the slope values extracted from the linear model to a theoretical normal distribution. Deviations from the normal distribution are indicated by increasing distance from the sloped blue dashed line. The horizontal blue dashed line highlights the coordinate on the y-axis where the slope value is equal to 0. Red dots represent strains with slope values significantly different from 0 ($p < 0.05$). Note, that this dataset yielded highly similar results as the full dataset (pan15). **(B)** Comparison of slope values derived from the reduced and the full dataset (pan15).

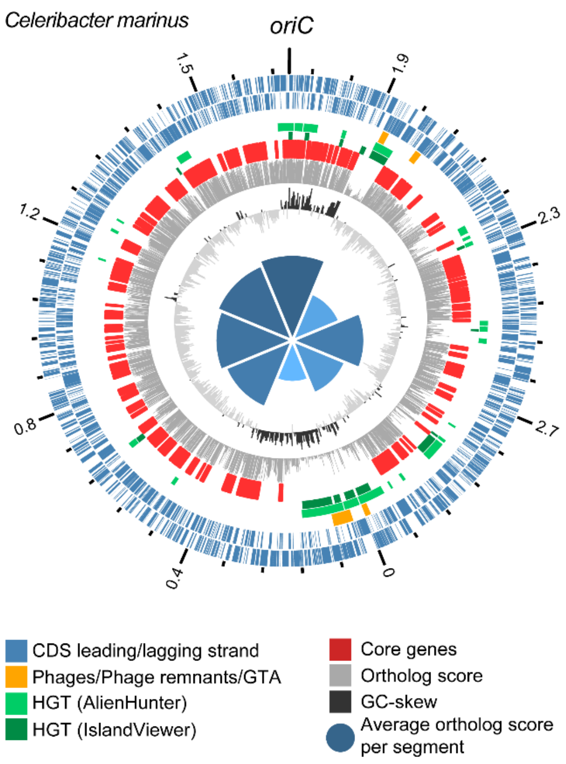

Supplementary Figure S8 – Circular representation of the *Celeribacter marinus* chromosome. The outer to inner rings represent: scale of genome size in Mb and origin of replication(*oriC*); position of ORFs encoded on the plus strand; position of ORFs encoded on the minus strand; groups of HT genes as defined in the graphical legend below; position of core genes with orthologs in all 108 *Rhodobacteraceae* strains; barchart displaying the ortholog score; GC-skew; polar plot showing the average ortholog score in each segment. Orthologs were identified using Proteinortho with cut-off criteria of e-value ≤ 1e-05, sequence identity ≥ 15%, and sequence coverage ≥ 70%.

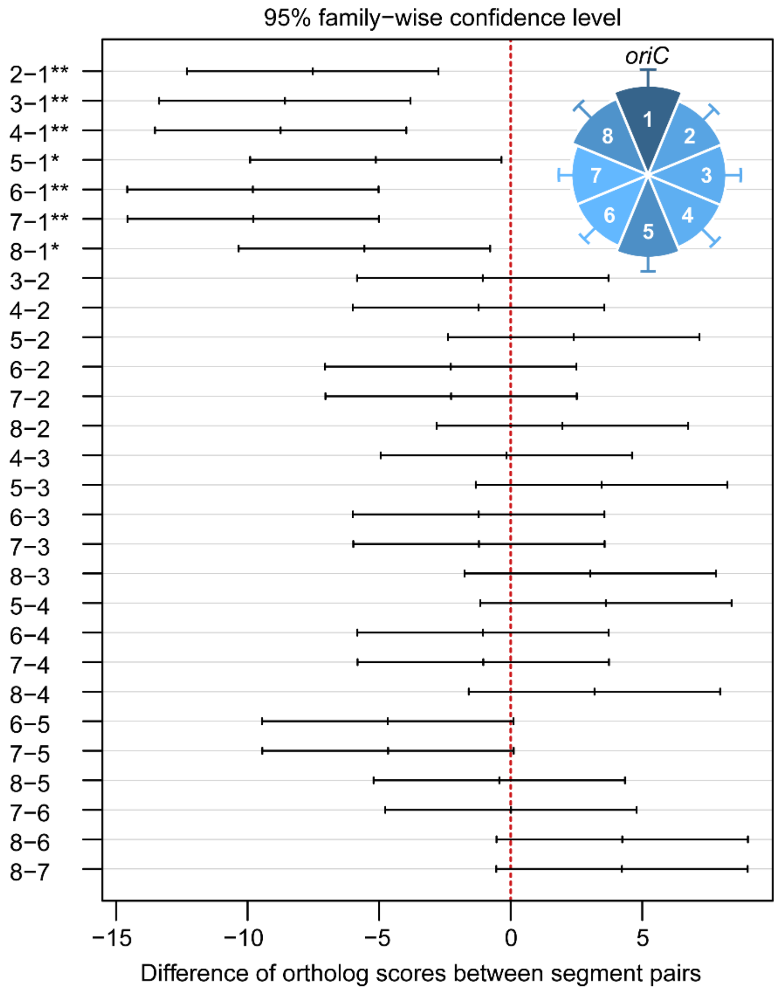

Supplementary Figure S9 – Result of Tukey's HSD test comparing all pairs of segments. Mean and 95% confidence interval of the differences in ortholog score are shown. Asterisks indicate significant differences between the compared segment pair (* $p < 0.05$, ** $p < 0.01$). Orthologs were identified using Proteinortho with cut-off criteria of e-value ≤ 1e-05, sequence identity ≥ 15%, and sequence coverage ≥ 70% (pan15 dataset).

Supplementary Table S1 – Programs and packages used in this study.

Software	Version	Reference
AlienHunter	1.7	Vernikos and Parkhill, 2006
MEGA	6.0	Tamura *et al.*, 2013
Prokka	1.12	Seemann T. 2014
ProteinOrtho	6.0b	Lechner *et al.*, 2011
R	3.4.1	-
Rstudio	1.0.15	-

**R packages**		
Biostrings	2.46.0	-
GenomicRanges	1.30.3	-
ggbio	1.26.1	-
tidyverse	1.2.1	-

Web Tool	Version	
Gblocks server	0.91b	Talavera and Castresana, 2007
PHaster	version 6	Arndt *et al.*, 2016
IslandViewer	version 4	Bertelli *et al.* 2017
Ori-Finder	Version 1	Gao and Zhang, 2008

Supplementary Table S2 – Command line options used for Prokka and ProteinOrtho.

PROKKA	--outdir [X] --prefix [X]
ProteinOrtho	-cpus=12 -verbose -e=1e-05 -identity=15 -cov=70 -synteny<br><br>-cpus=12 -verbose -e=1e-10 -identity=30 -cov=70 -synteny<br><br>-cpus=12 -verbose -e=1e-10 -identity=60 -cov=80 -synteny
