# Supplementary material for: Clustered Core- and Pan-Genome Content on Rhodobacteraceae Chromosomes
Source: Genome Biol Evol. 2019 Jul 3;11(8):2208–17. doi: 10.1093/gbe/evz138 (PMC6699656; doi:10.1093/gbe/evz138)
Supplement: evz138_Supplementary_Data [file evz138_supplementary_data.zip › Supplementary_Figure_S3_models_pan30.pdf]

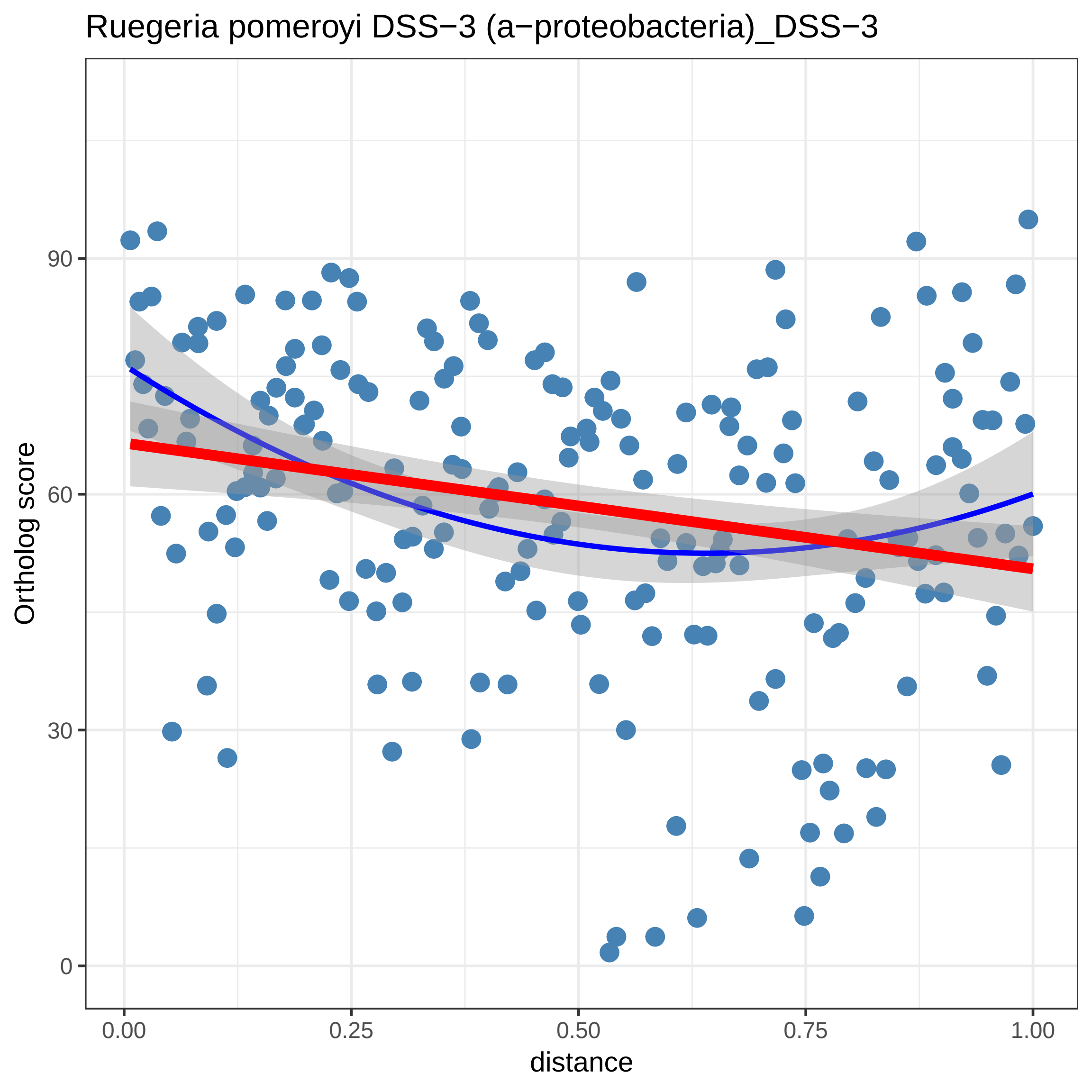
Ruegeria pomeroyi DSS−3 (a−proteobacteria)_DSS−3
Ortholog score
90
60
30
0
0.00
0.25
0.50
0.75
1.00
distance

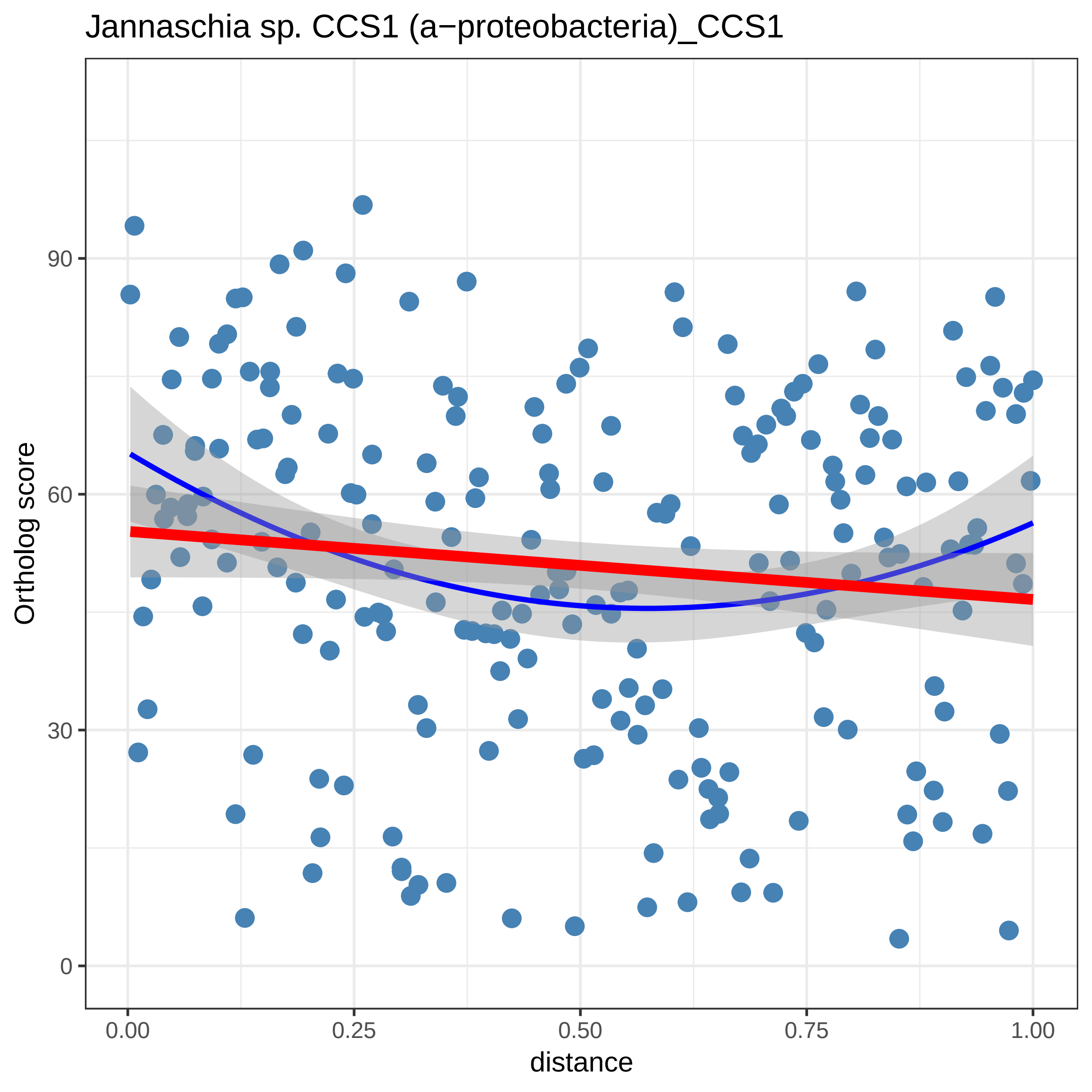

Jannaschia sp. CCS1 (a−proteobacteria)_CCS1
Ortholog score
90
60
30
0
0.00
0.25
0.50
0.75
1.00
distance

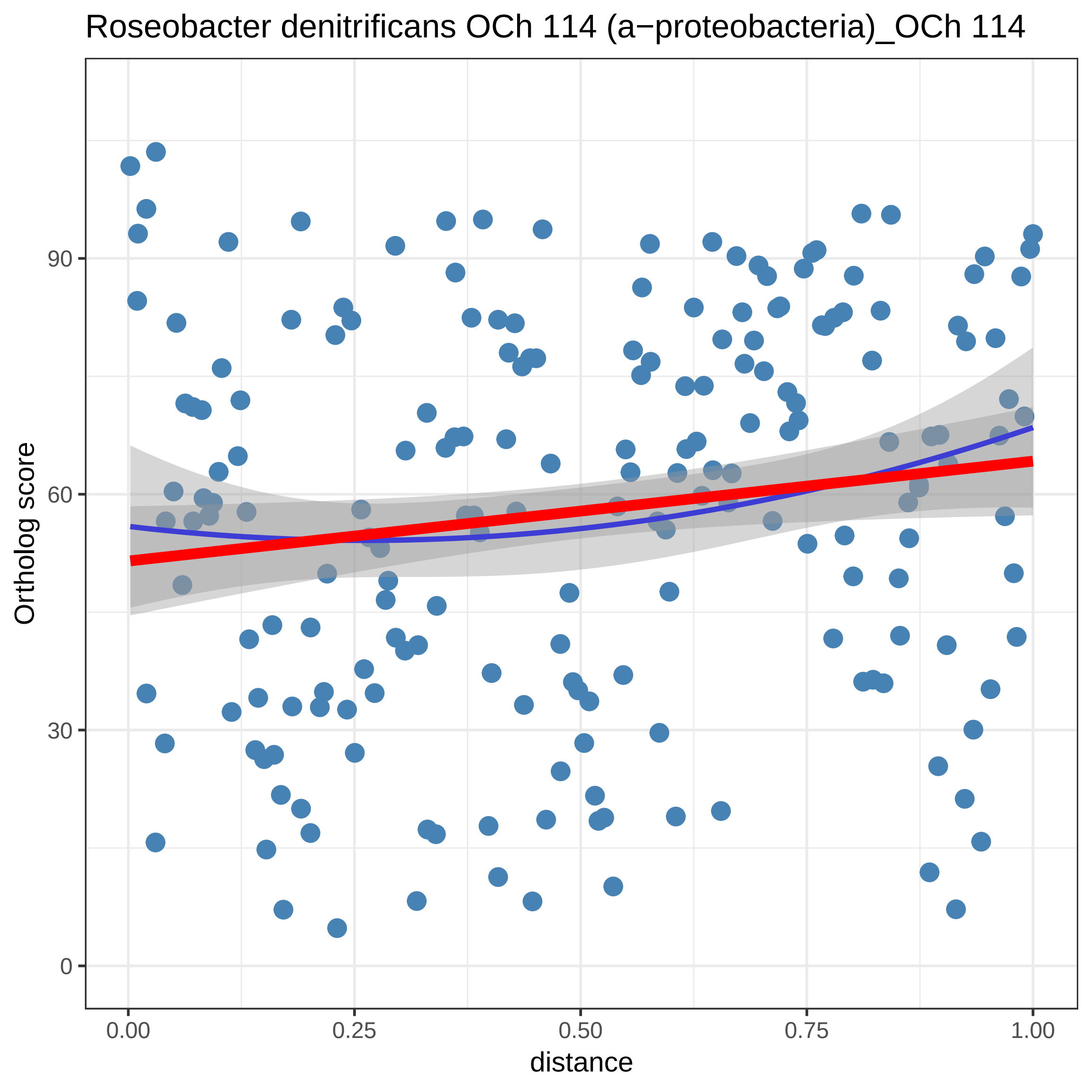

Roseobacter denitrificans OCh 114 (a−proteobacteria)_OCh 114
Ortholog score
90
60
30
0
0.00
0.25
0.50
0.75
1.00
distance

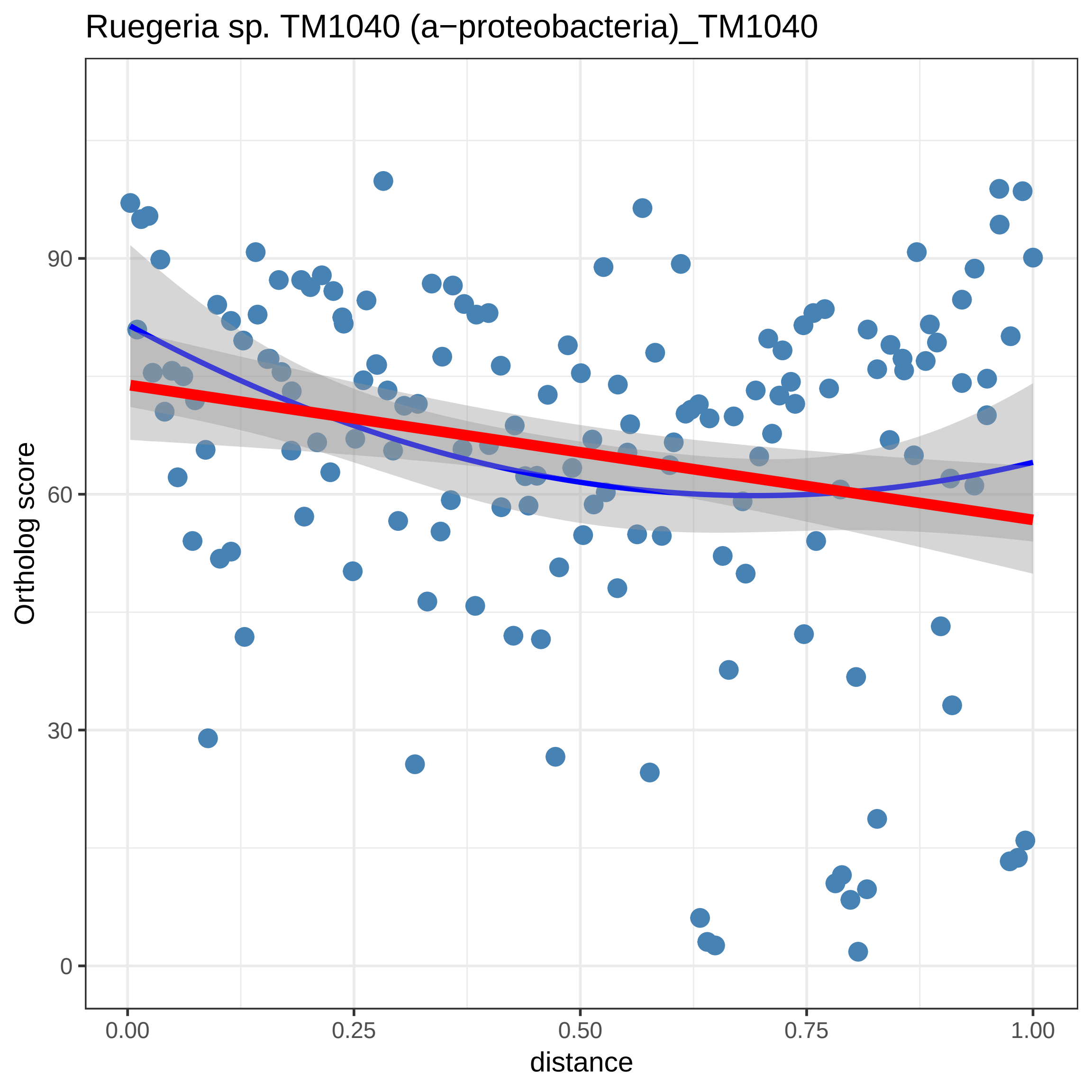

Ruegeria sp. TM1040 (a−proteobacteria)_TM1040
Ortholog score
90
60
30
0
0.00
0.25
0.50
0.75
1.00
distance

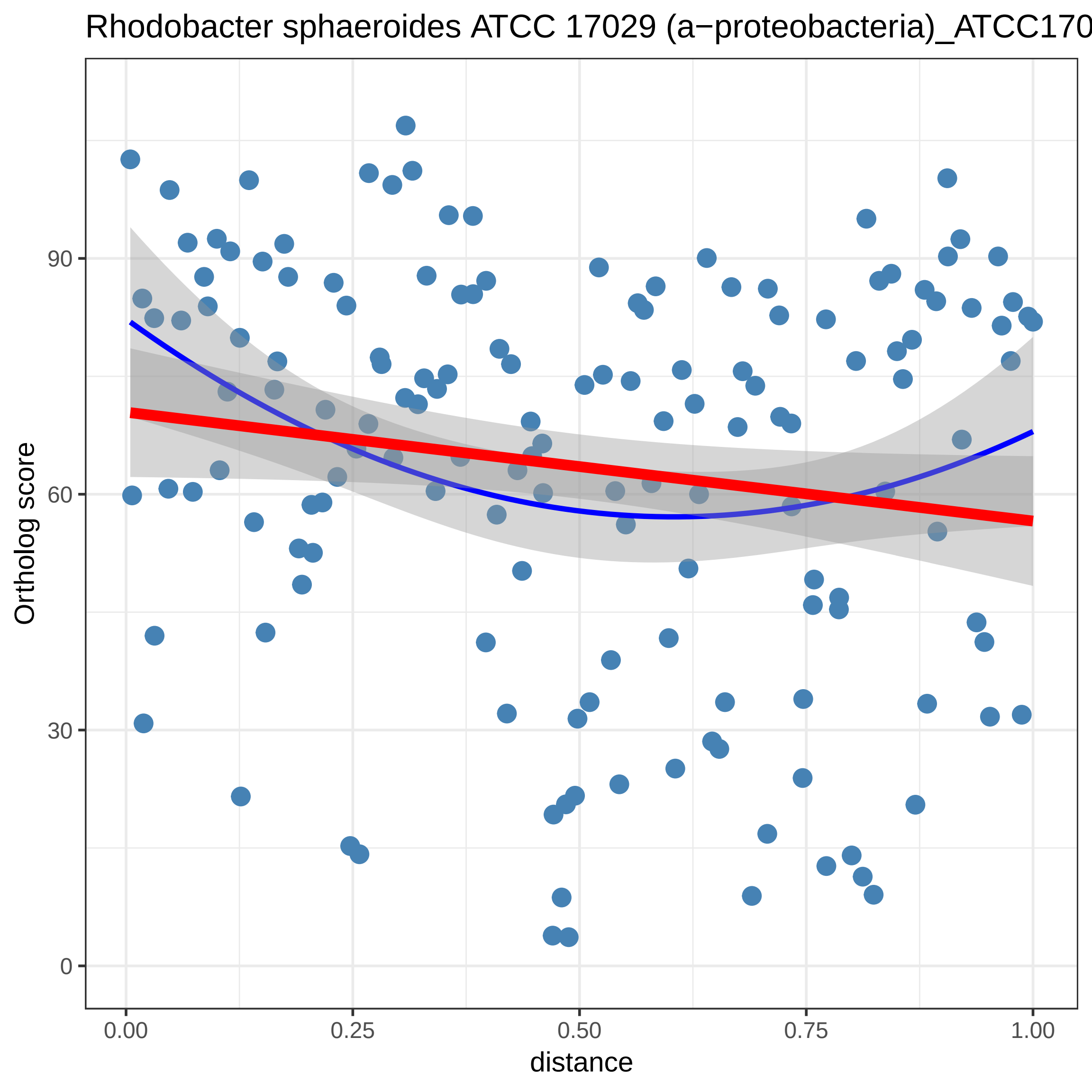

Rhodobacter sphaeroides ATCC 17029 (a−proteobacteria)_ATCC170
Ortholog score
90
60
30
0
0.00
0.25
0.50
0.75
1.00
distance

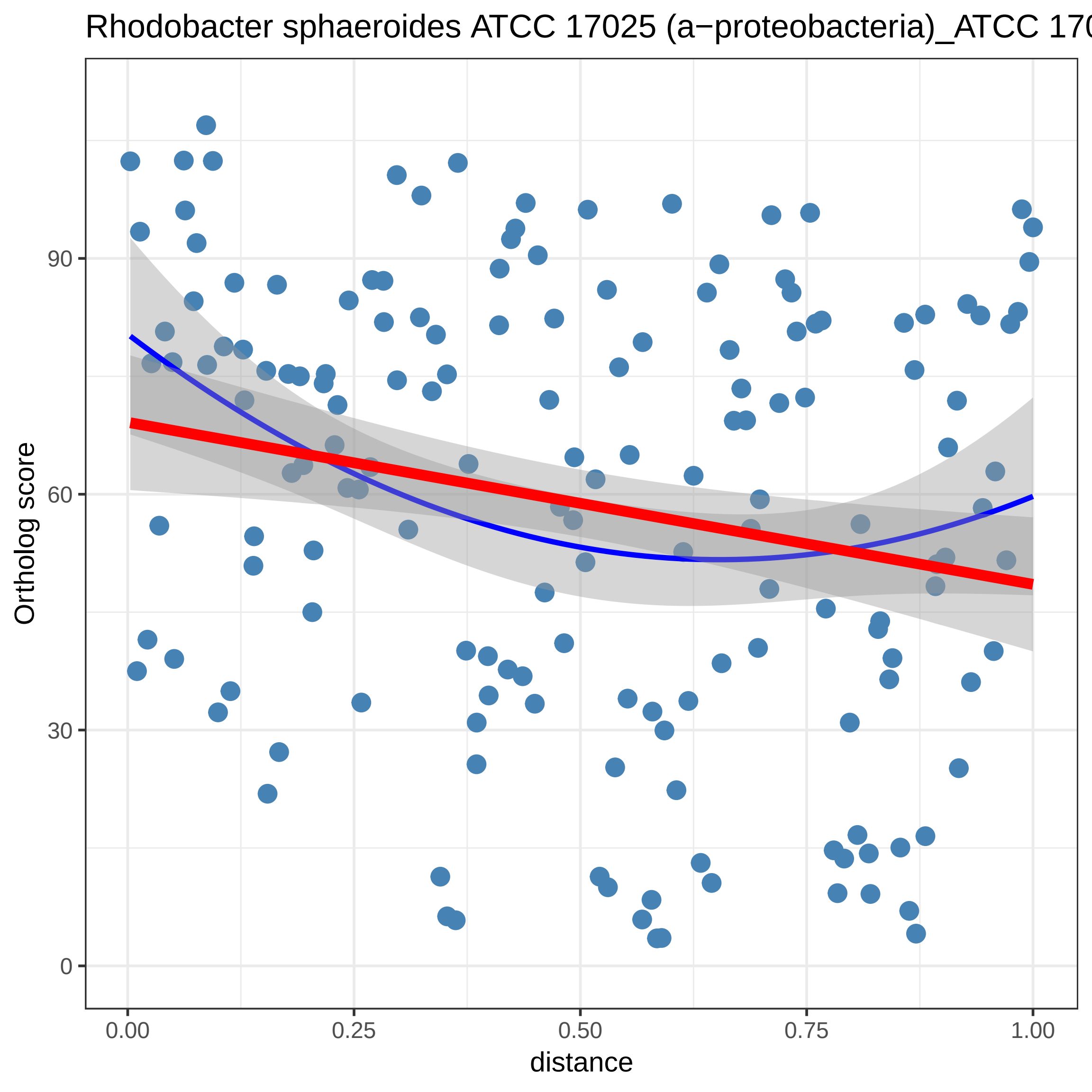

Rhodobacter sphaeroides ATCC 17025 (a−proteobacteria)_ATCC 170
Ortholog score
distance
0
30
60
90
0.00
0.25
0.50
0.75
1.00

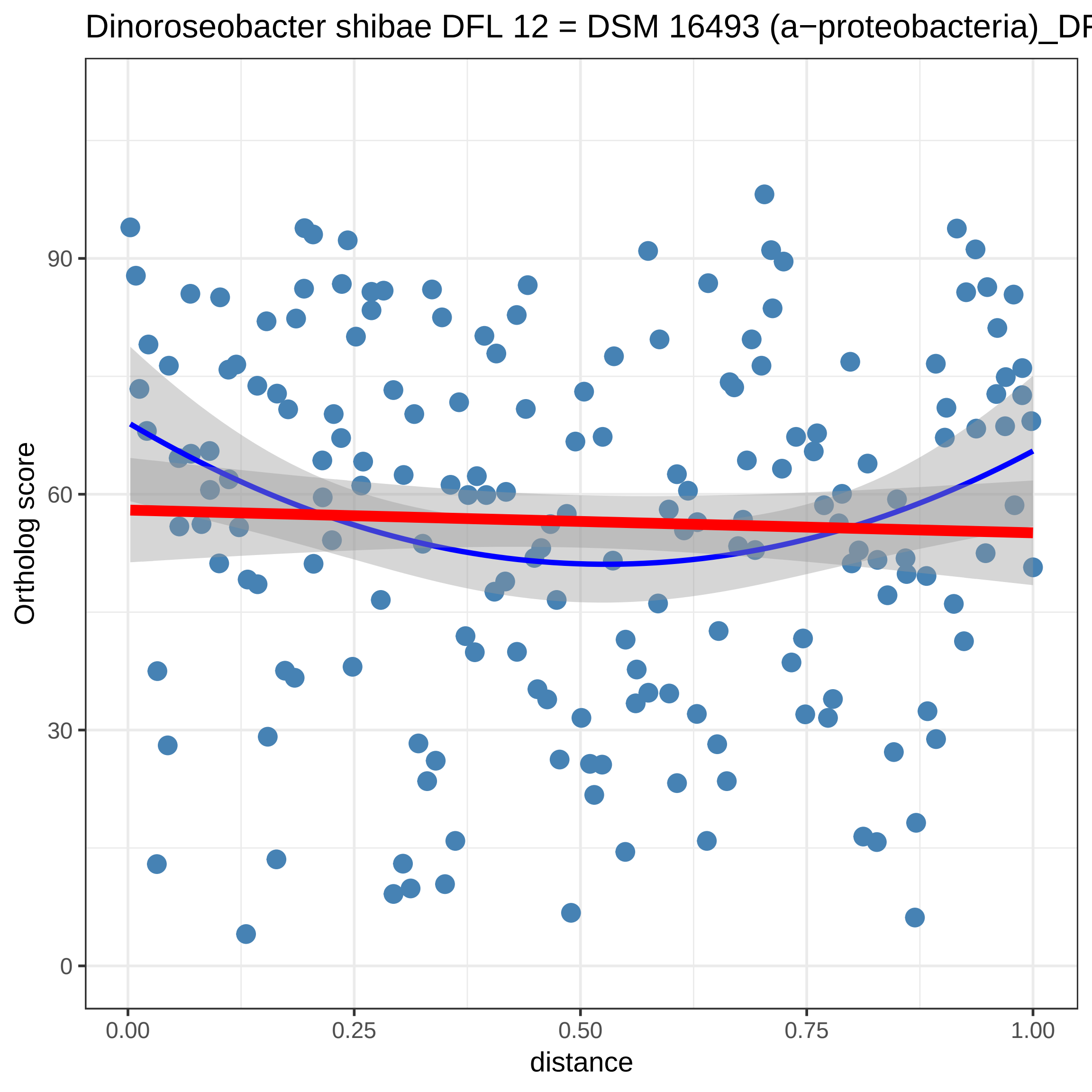

Dinoroseobacter shibae DFL 12 = DSM 16493 (a−proteobacteria)_DF
Ortholog score
90
60
30
0
0.00
0.25
0.50
0.75
1.00
distance

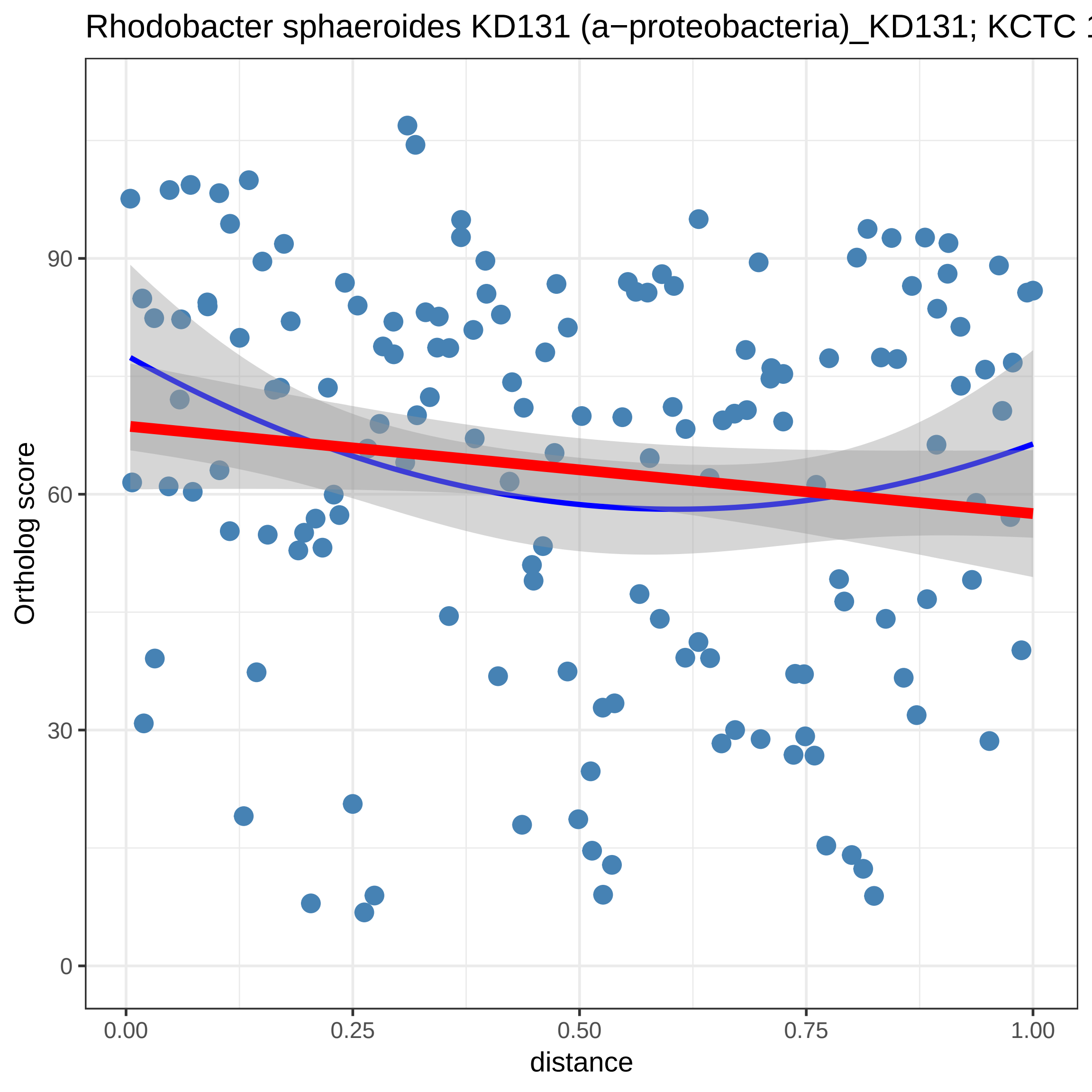
Rhodobacter sphaeroides KD131 (a−proteobacteria)_KD131; KCTC 1
Ortholog score
0
30
60
90
0.00
0.25
0.50
0.75
1.00
distance

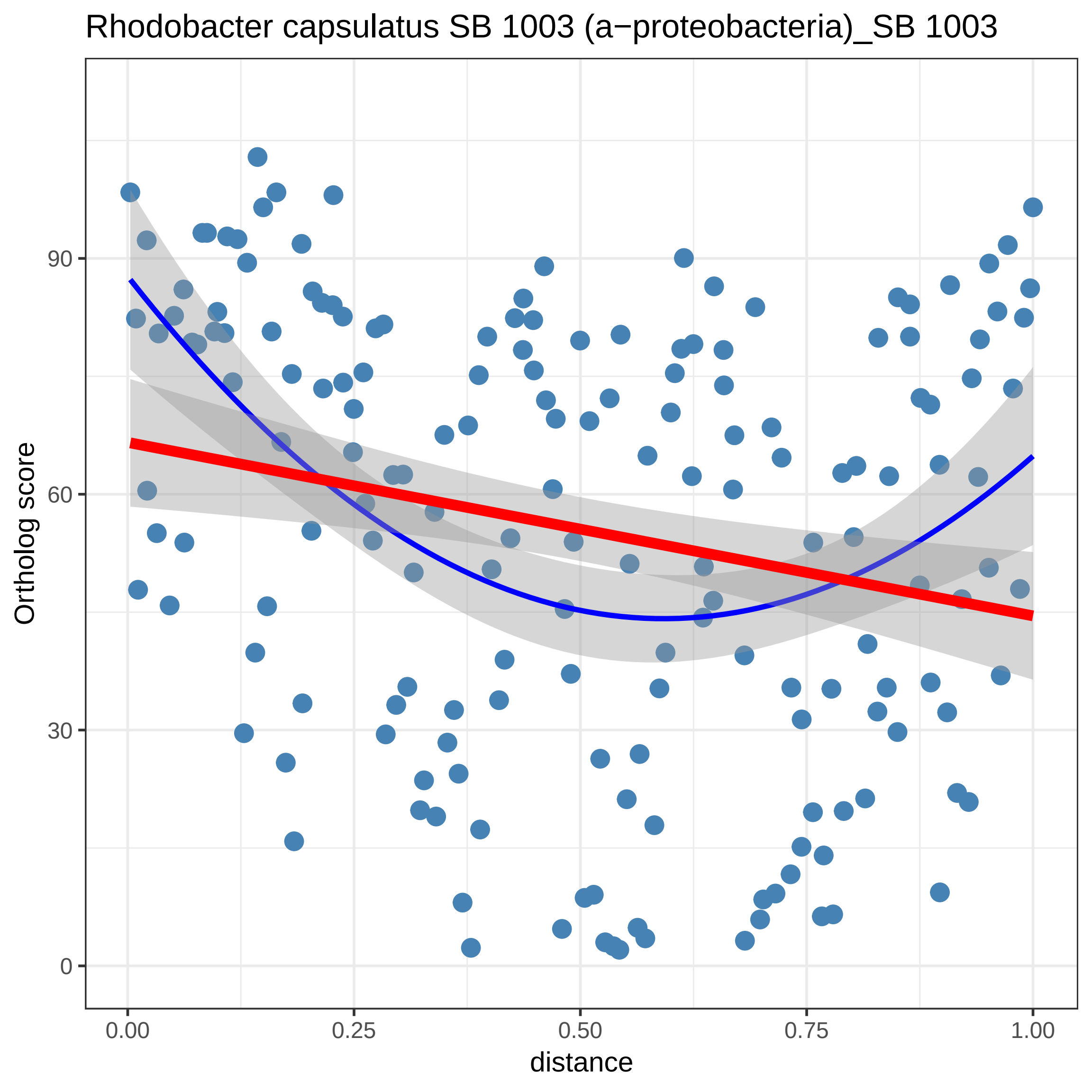
Rhodobacter capsulatus SB 1003 (a−proteobacteria)_SB 1003
Ortholog score
90
60
30
0
0.00
0.25
0.50
0.75
1.00
distance

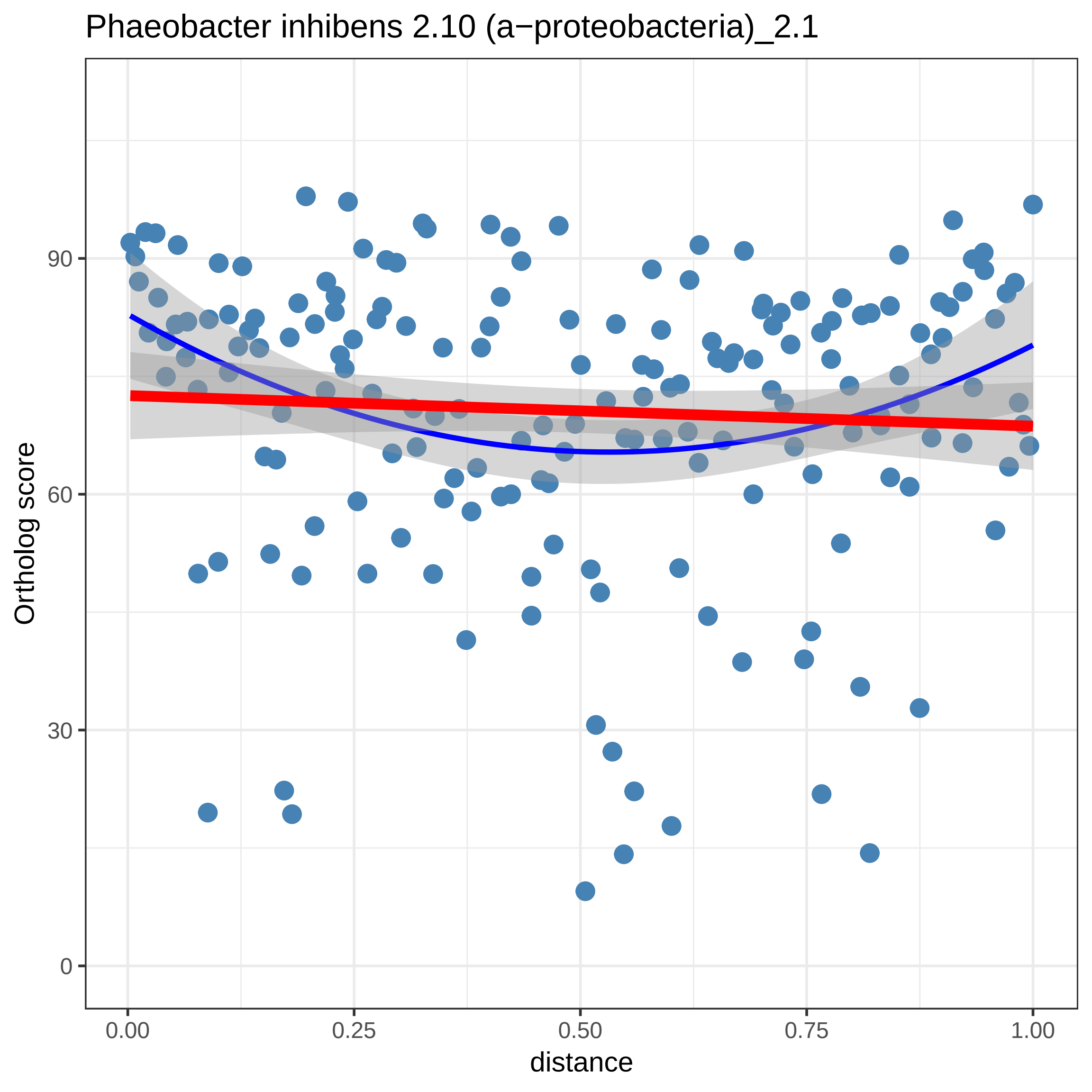
Phaeobacter inhibens 2.10 (a−proteobacteria)_2.1
Ortholog score
90
60
30
0
0.00
0.25
0.50
0.75
1.00
distance

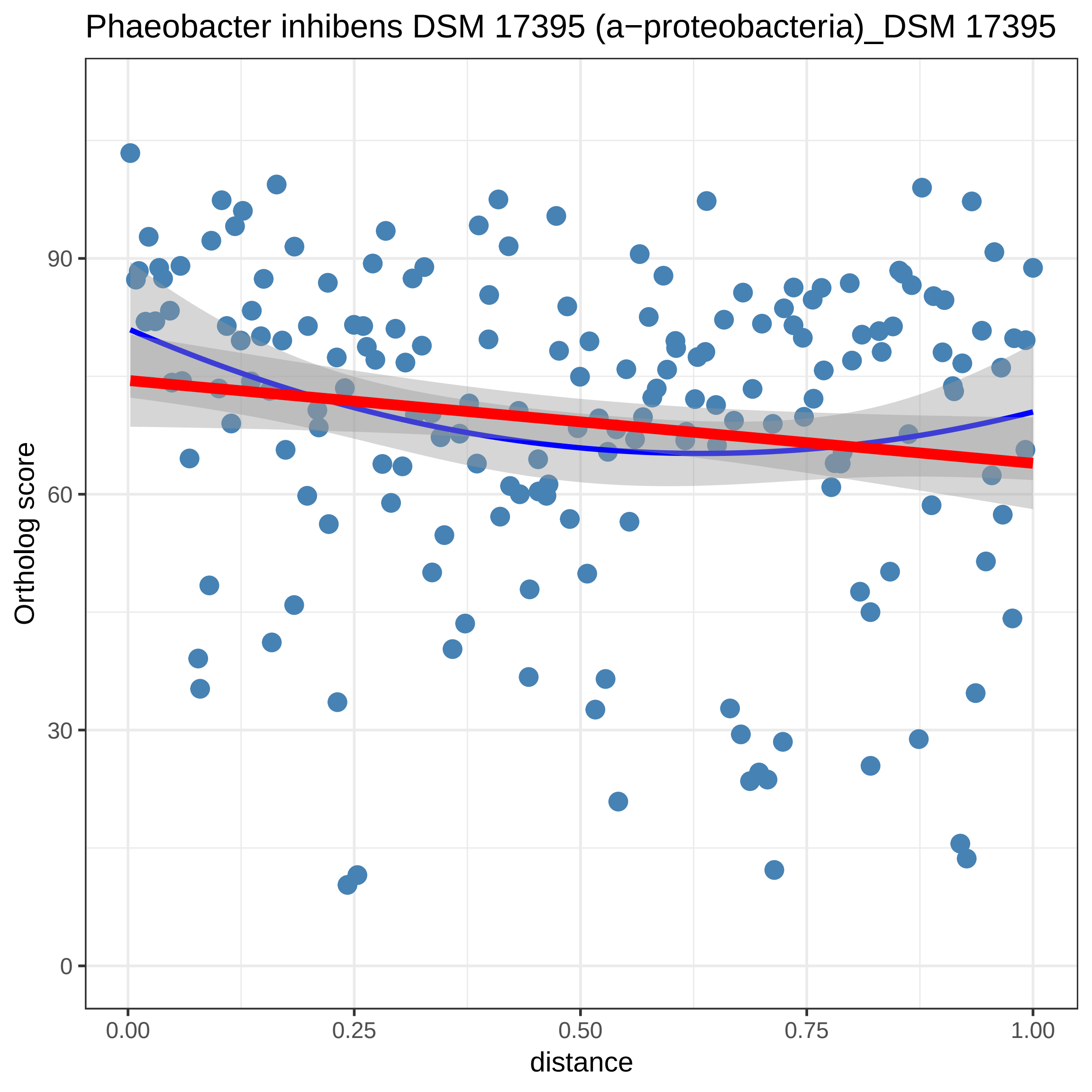
Phaeobacter inhibens DSM 17395 (a−proteobacteria)_DSM 17395
Ortholog score
90
60
30
0
0.00
0.25
0.50
0.75
1.00
distance

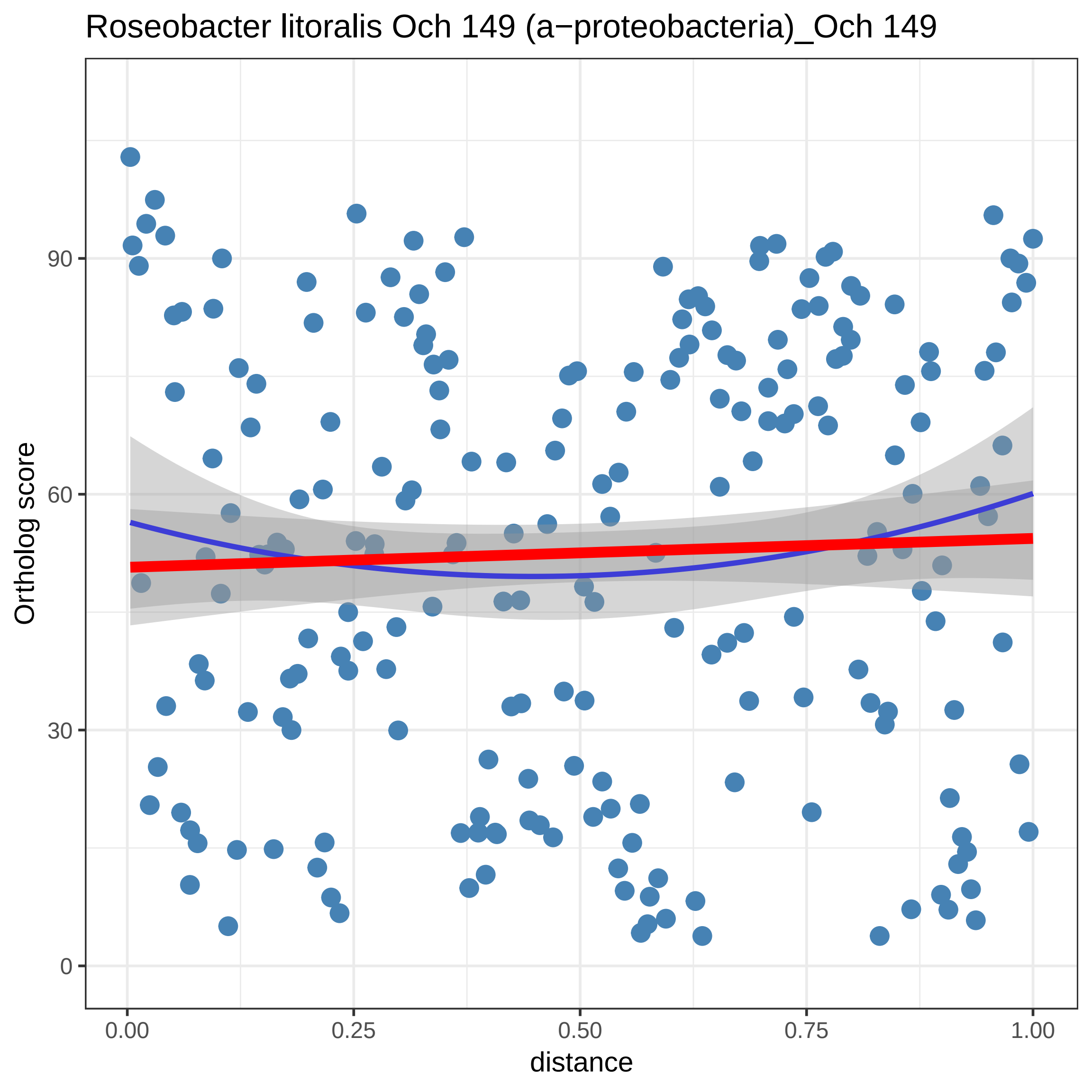
Roseobacter litoralis Och 149 (a−proteobacteria)_Och 149
Ortholog score
90
60
30
0
0.00
0.25
0.50
0.75
1.00
distance

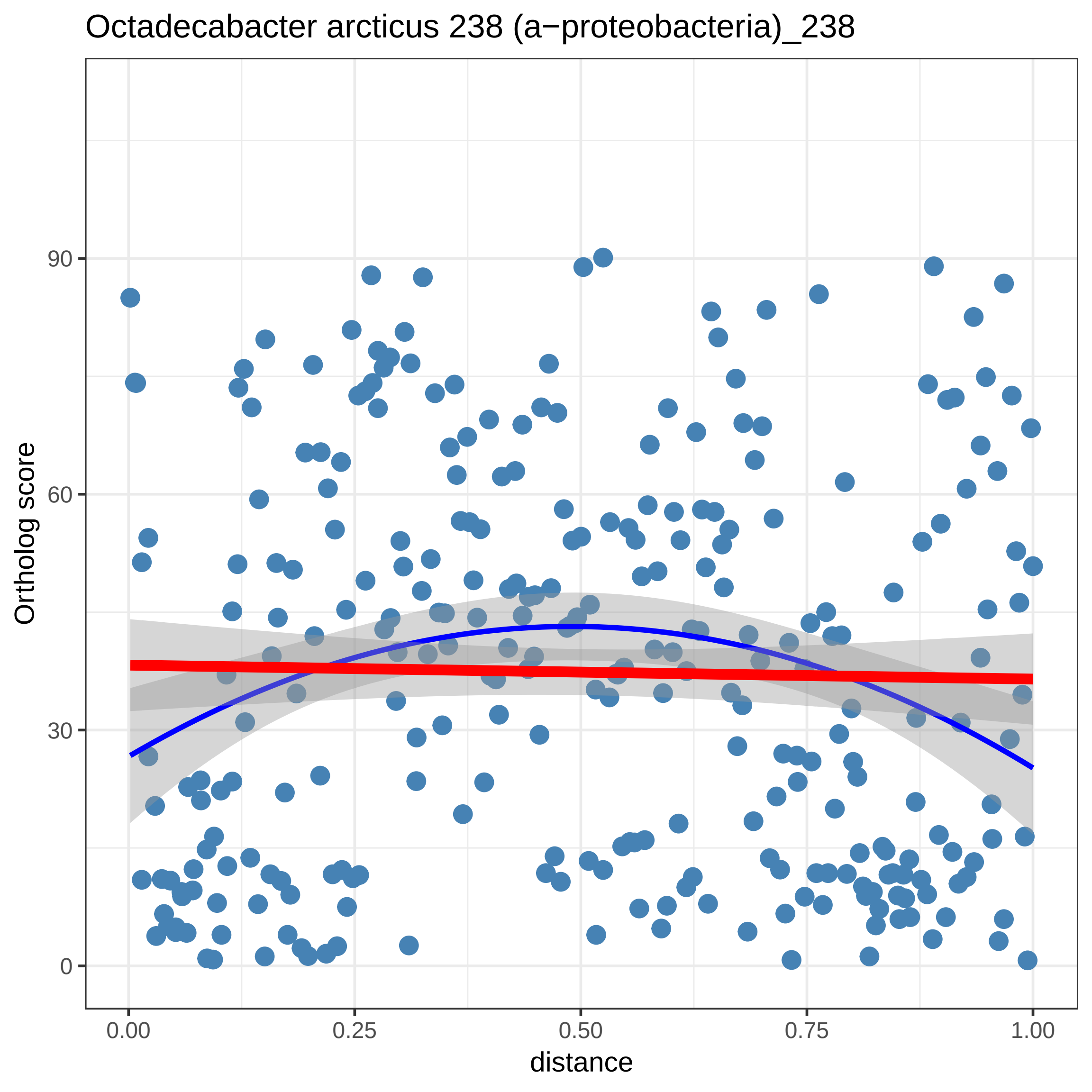

Octadecabacter arcticus 238 (a−proteobacteria)_238
Ortholog score
0
30
60
90
0.00
0.25
0.50
0.75
1.00
distance

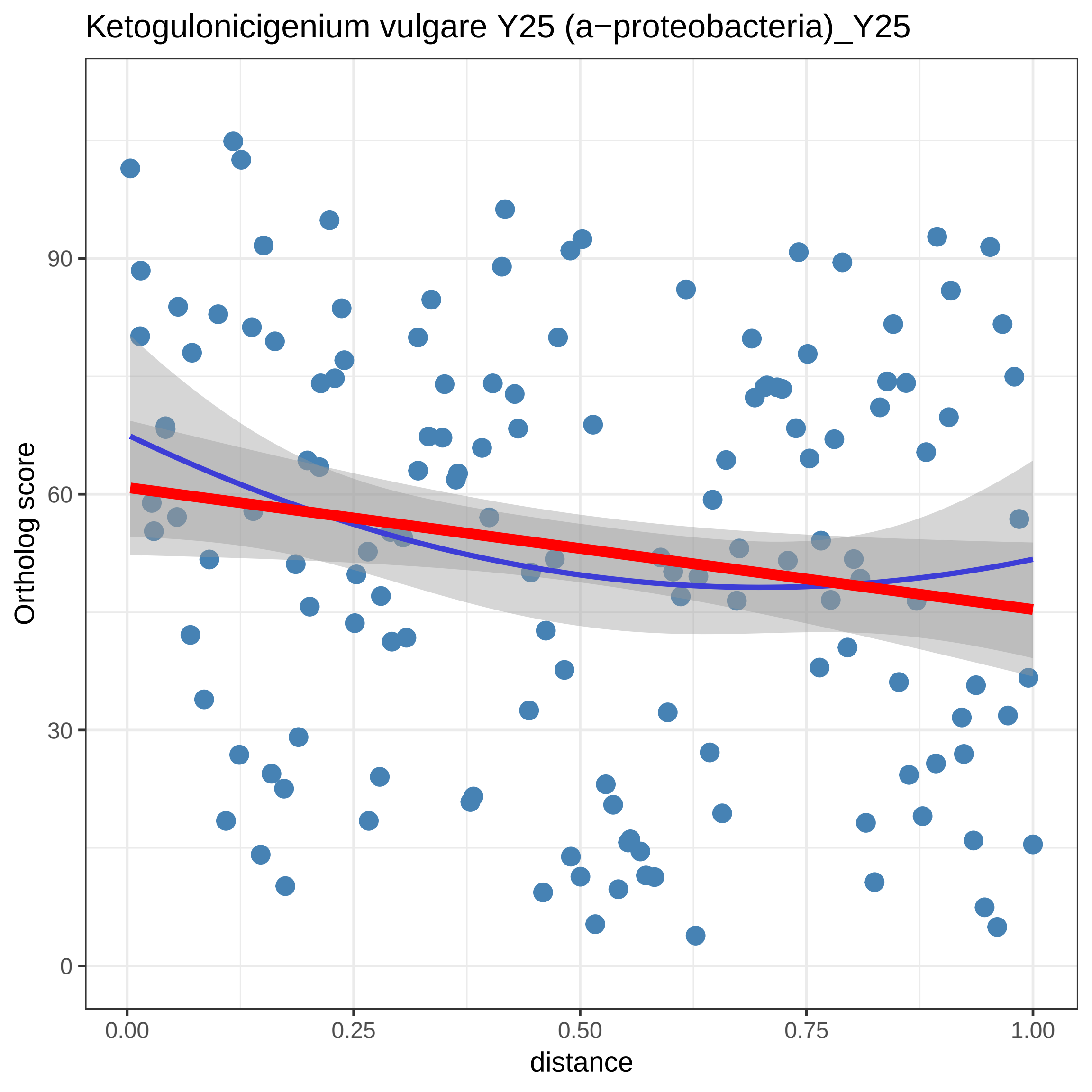

Ketogulonicigenium vulgare Y25 (a−proteobacteria)_Y25
Ortholog score
0
30
60
90
0.00
0.25
0.50
0.75
1.00
distance

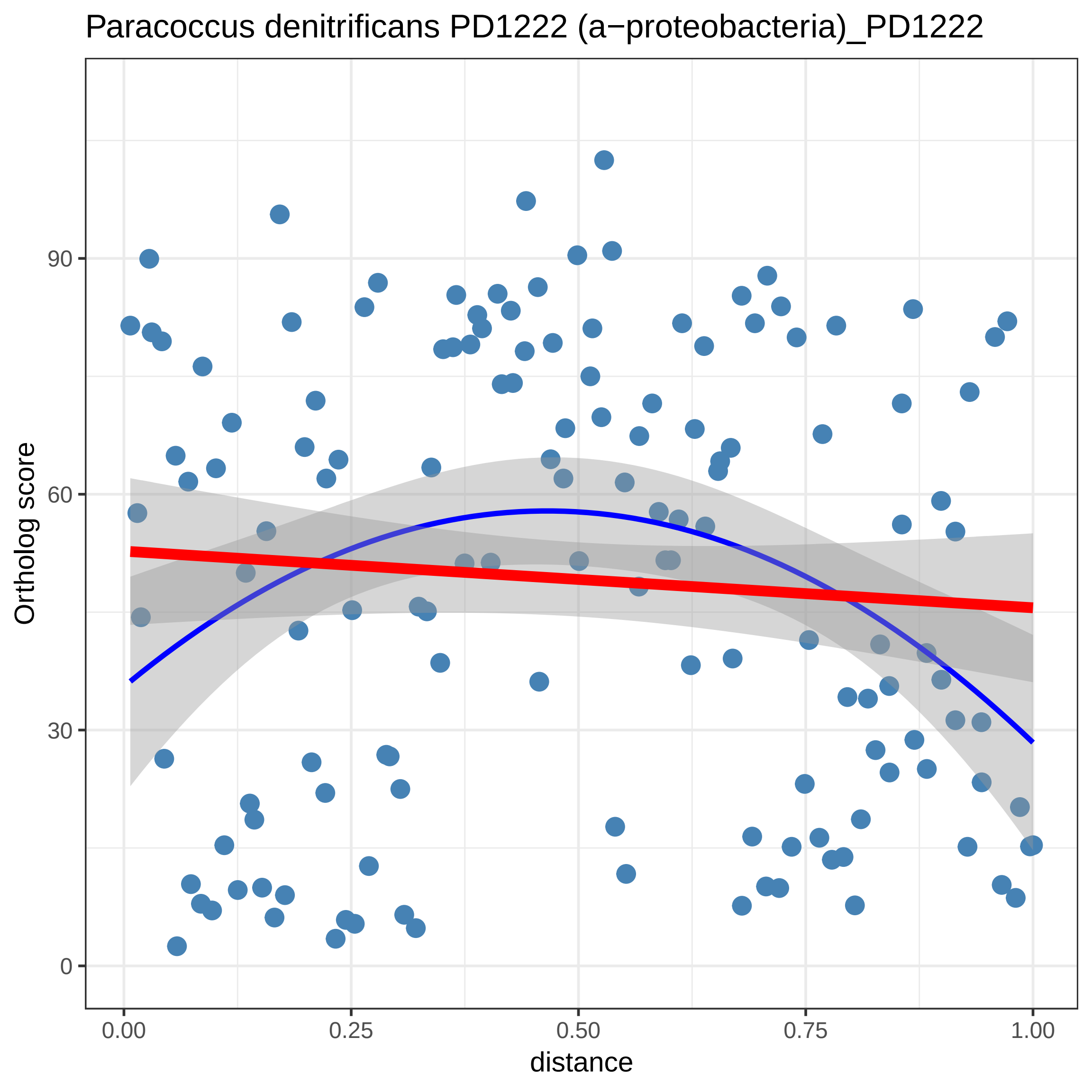
Paracoccus denitrificans PD1222 (a−proteobacteria)_PD1222
Ortholog score
0
30
60
90
0.00
0.25
0.50
0.75
1.00
distance

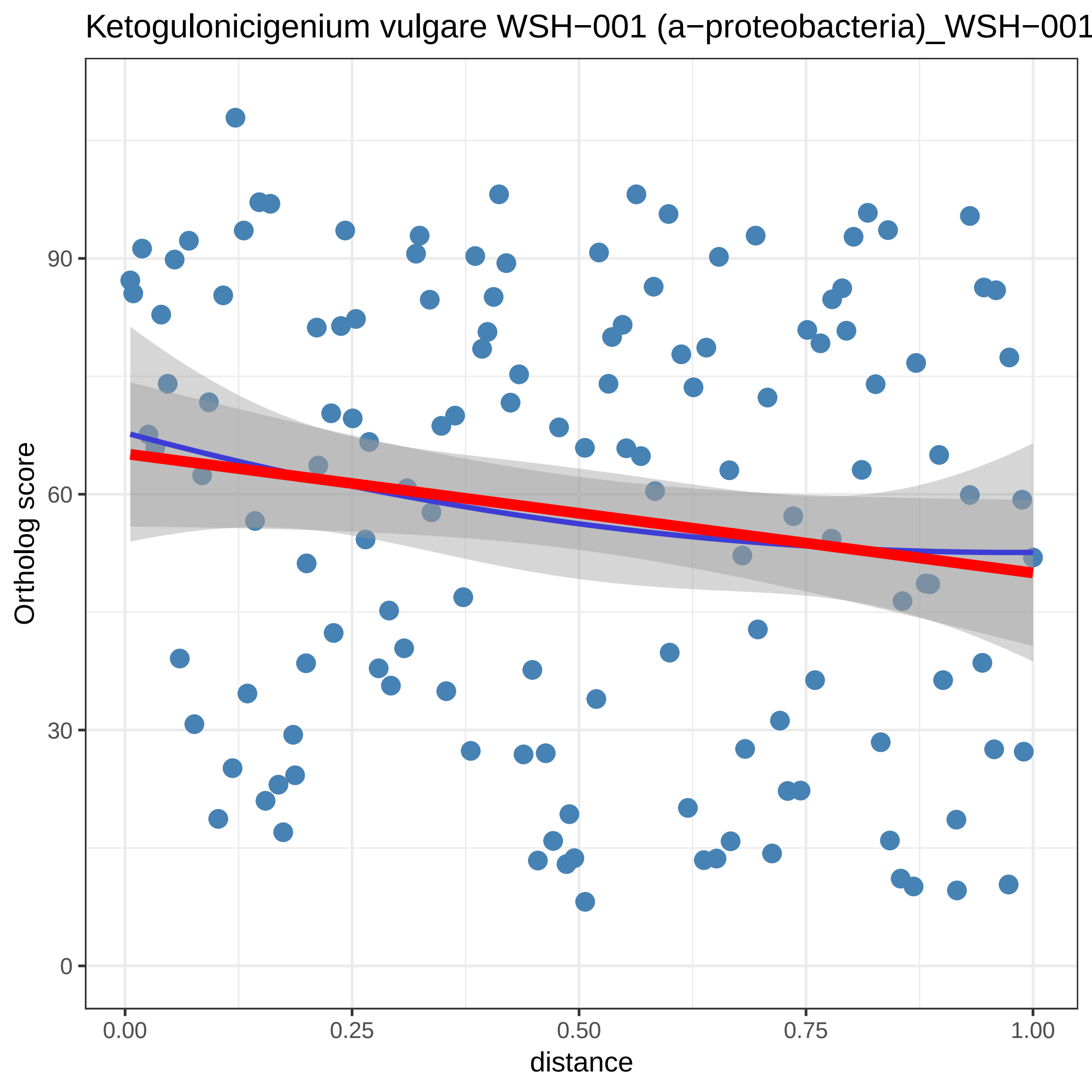

Ketogulonicigenium vulgare WSH−001 (a−proteobacteria)_WSH−001
Ortholog score
0
30
60
90
0.00
0.25
0.50
0.75
1.00
distance

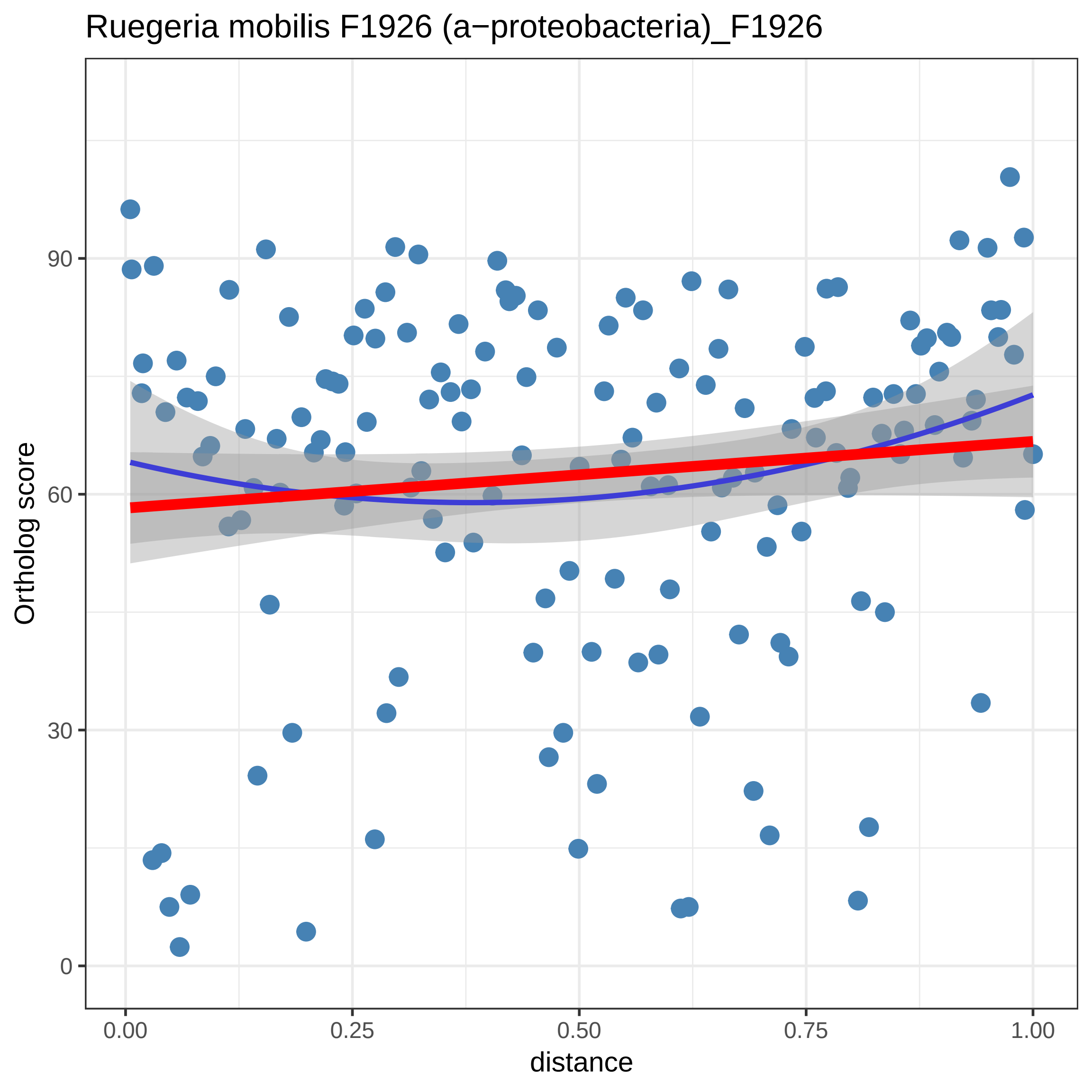
Ruegeria mobilis F1926 (a−proteobacteria)_F1926
Ortholog score
90
60
30
0
0.00
0.25
0.50
0.75
1.00
distance

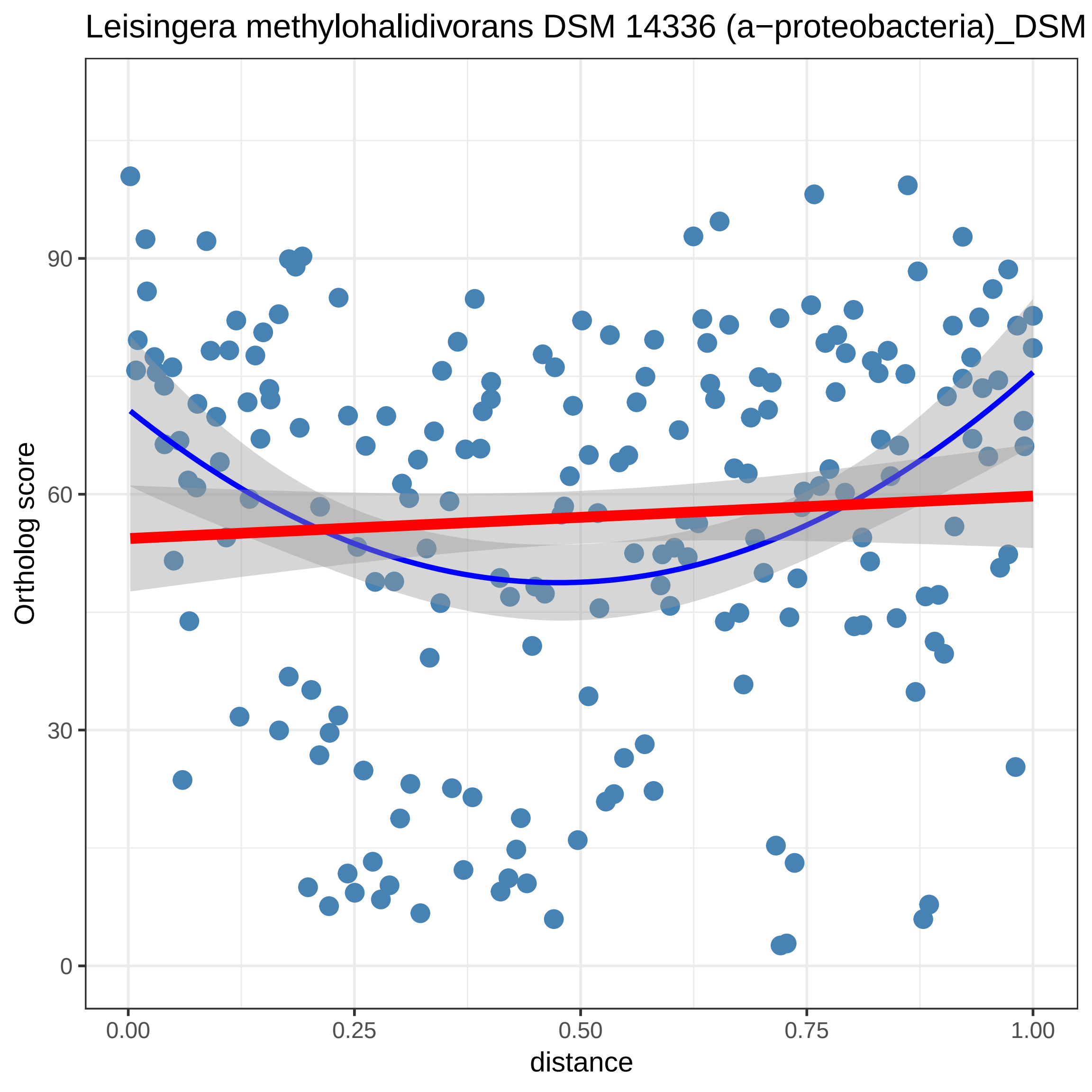

Leisingera methylohalidivorans DSM 14336 (a−proteobacteria)_DSM
Ortholog score
90
60
30
0
0.00
0.25
0.50
0.75
1.00
distance

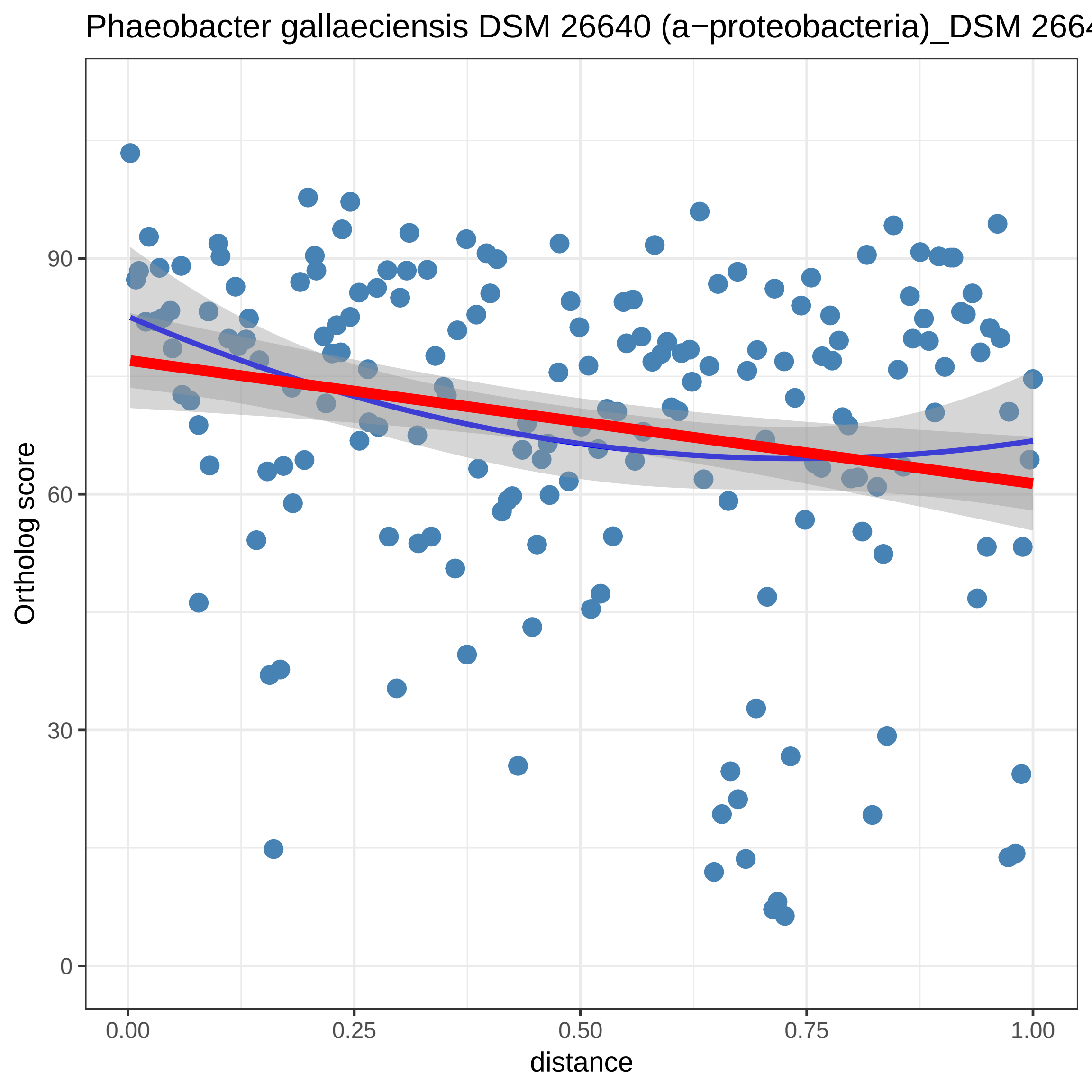
Phaeobacter gallaeciensis DSM 26640 (a−proteobacteria)_DSM 2664
Ortholog score
distance
0
30
60
90
0.00
0.25
0.50
0.75
1.00

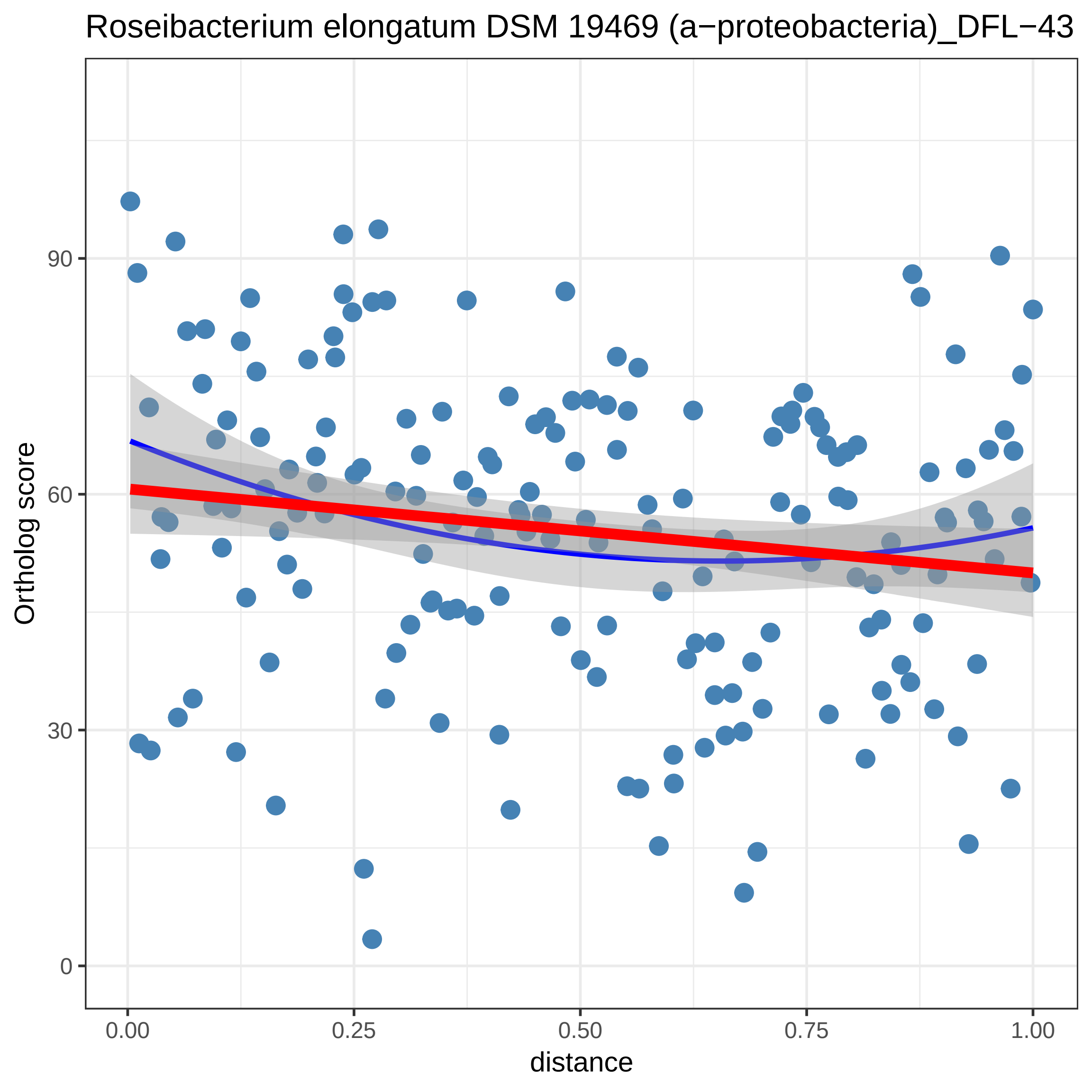
Roseibacterium elongatum DSM 19469 (a−proteobacteria)_DFL−43
Ortholog score
90
60
30
0
0.00
0.25
0.50
0.75
1.00
distance

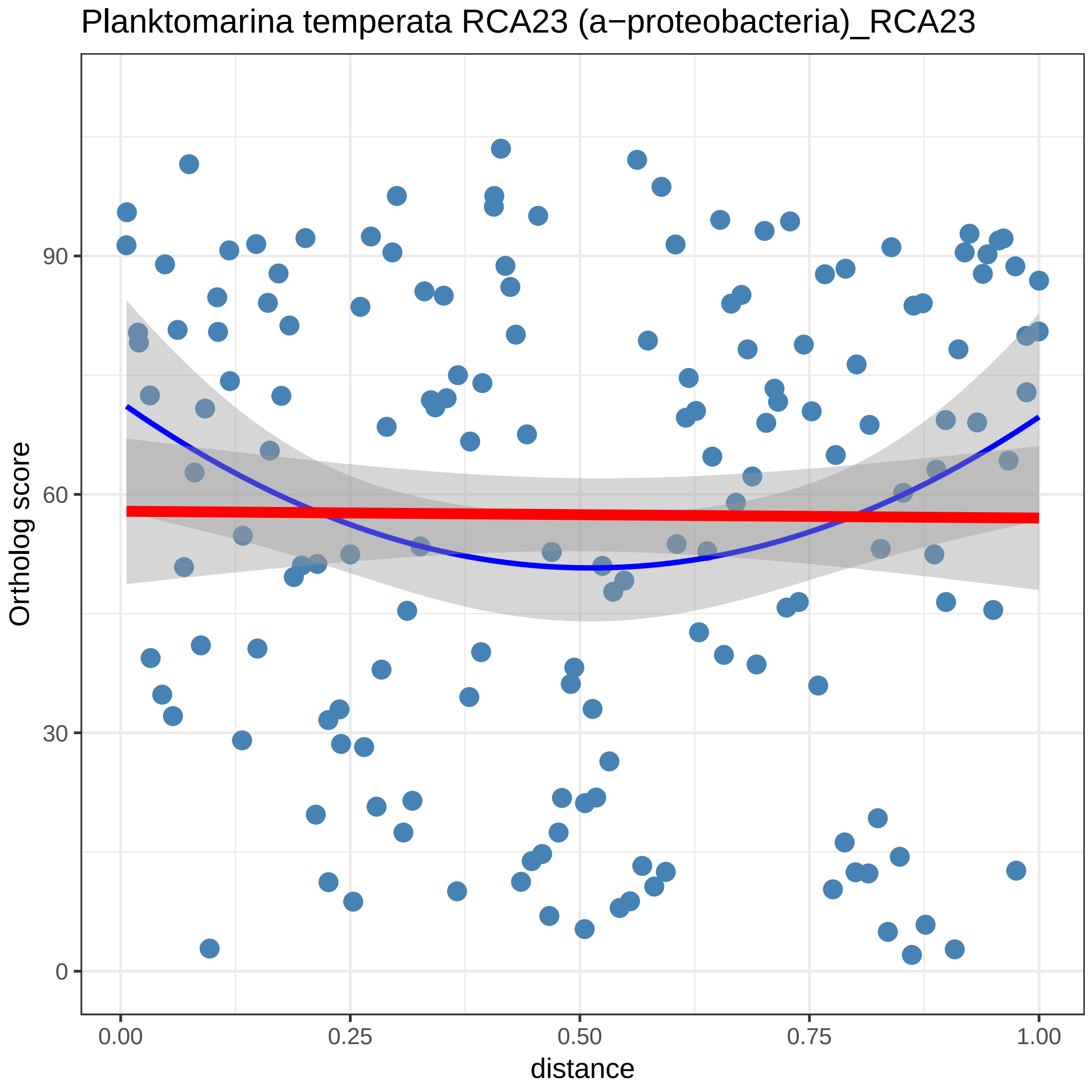
Planktomarina temperata RCA23 (a−proteobacteria)_RCA23
Ortholog score
90
60
30
0
0.00
0.25
0.50
0.75
1.00
distance

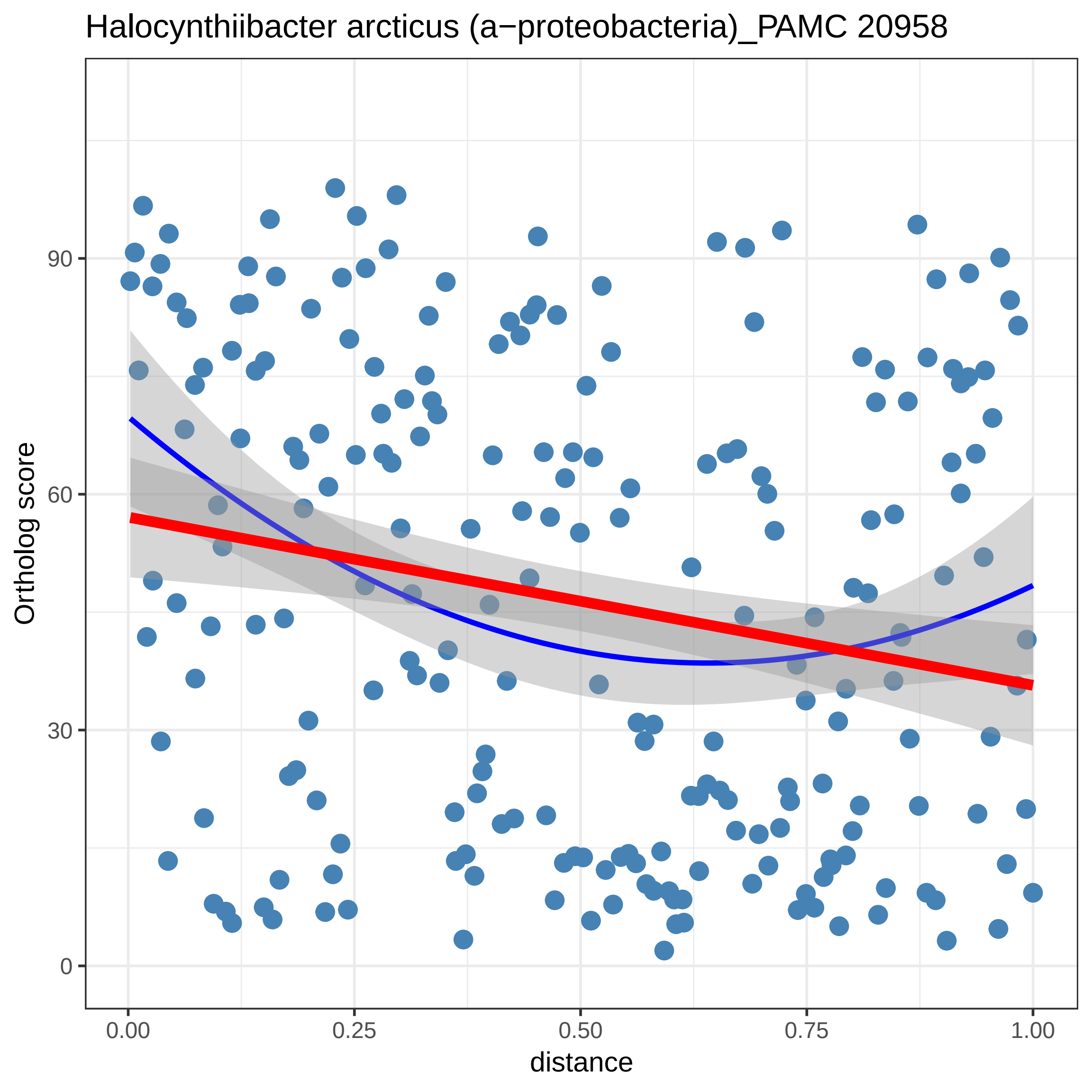

Halocynthiibacter arcticus (a−proteobacteria)_PAMC 20958
Ortholog score
0
30
60
90
0.00
0.25
0.50
0.75
1.00
distance

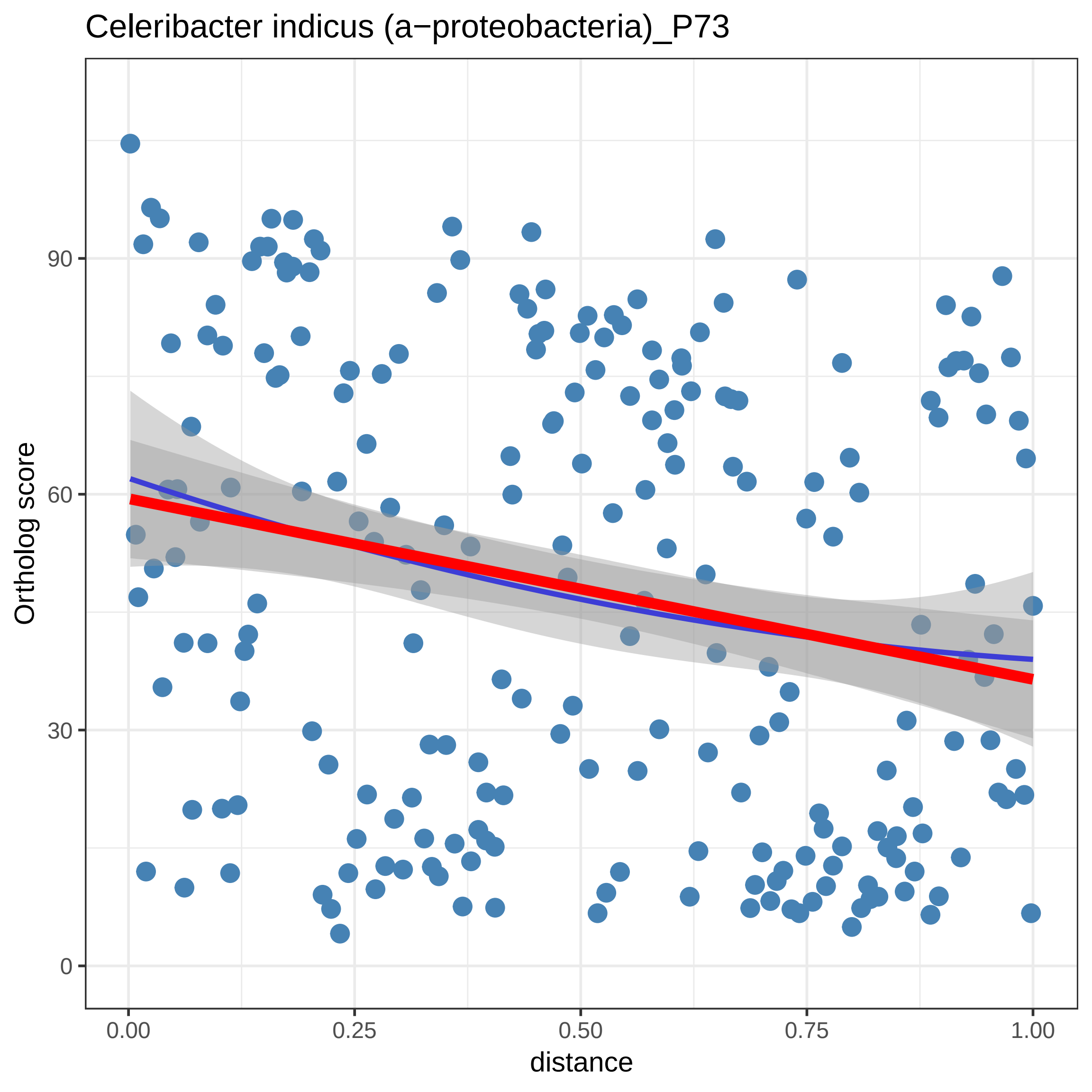

Celeribacter indicus (a−proteobacteria)_P73
Ortholog score
distance
0
30
60
90
0.00
0.25
0.50
0.75
1.00

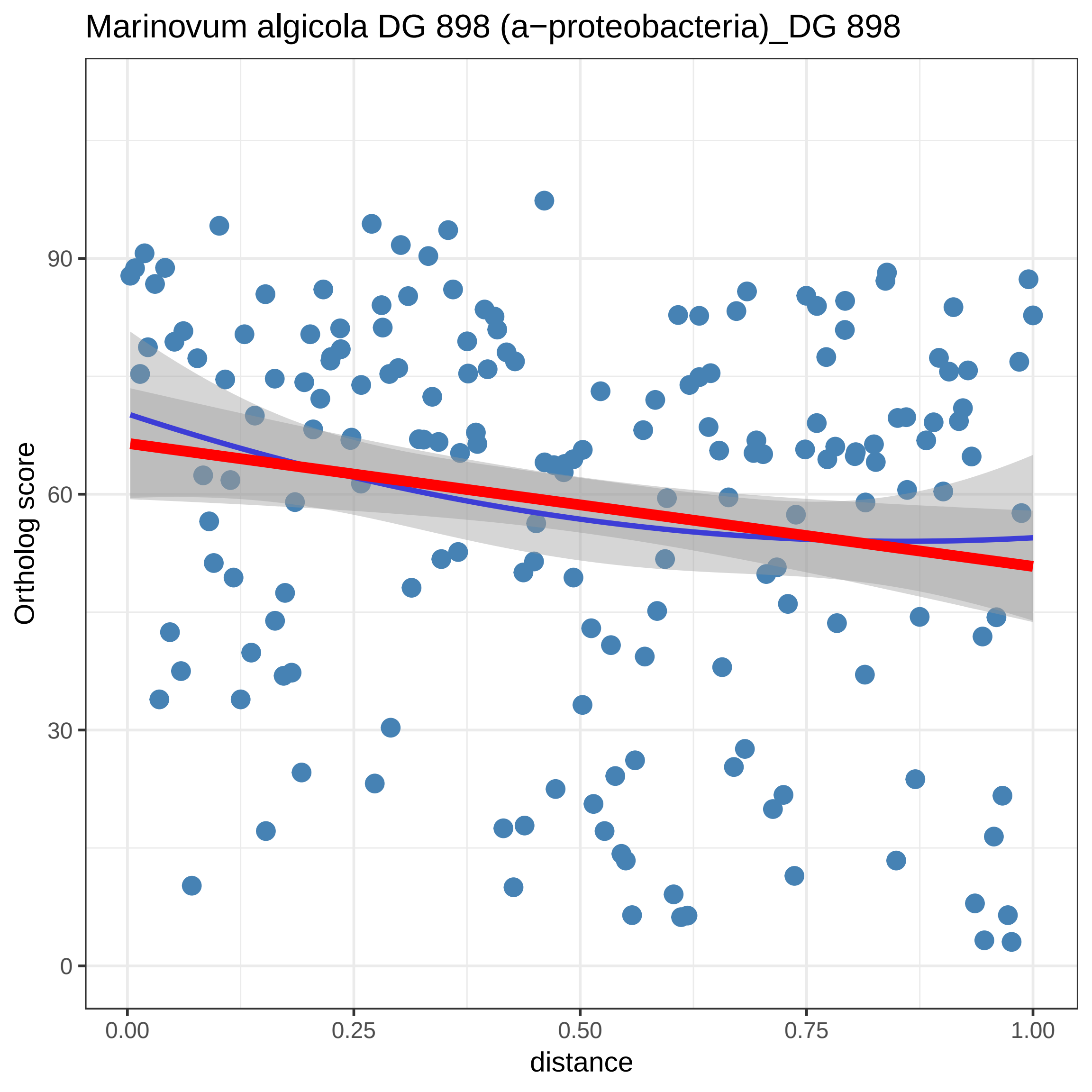

Marinovum algicola DG 898 (a−proteobacteria)_DG 898
Ortholog score
distance
0
30
60
90
0.00
0.25
0.50
0.75
1.00

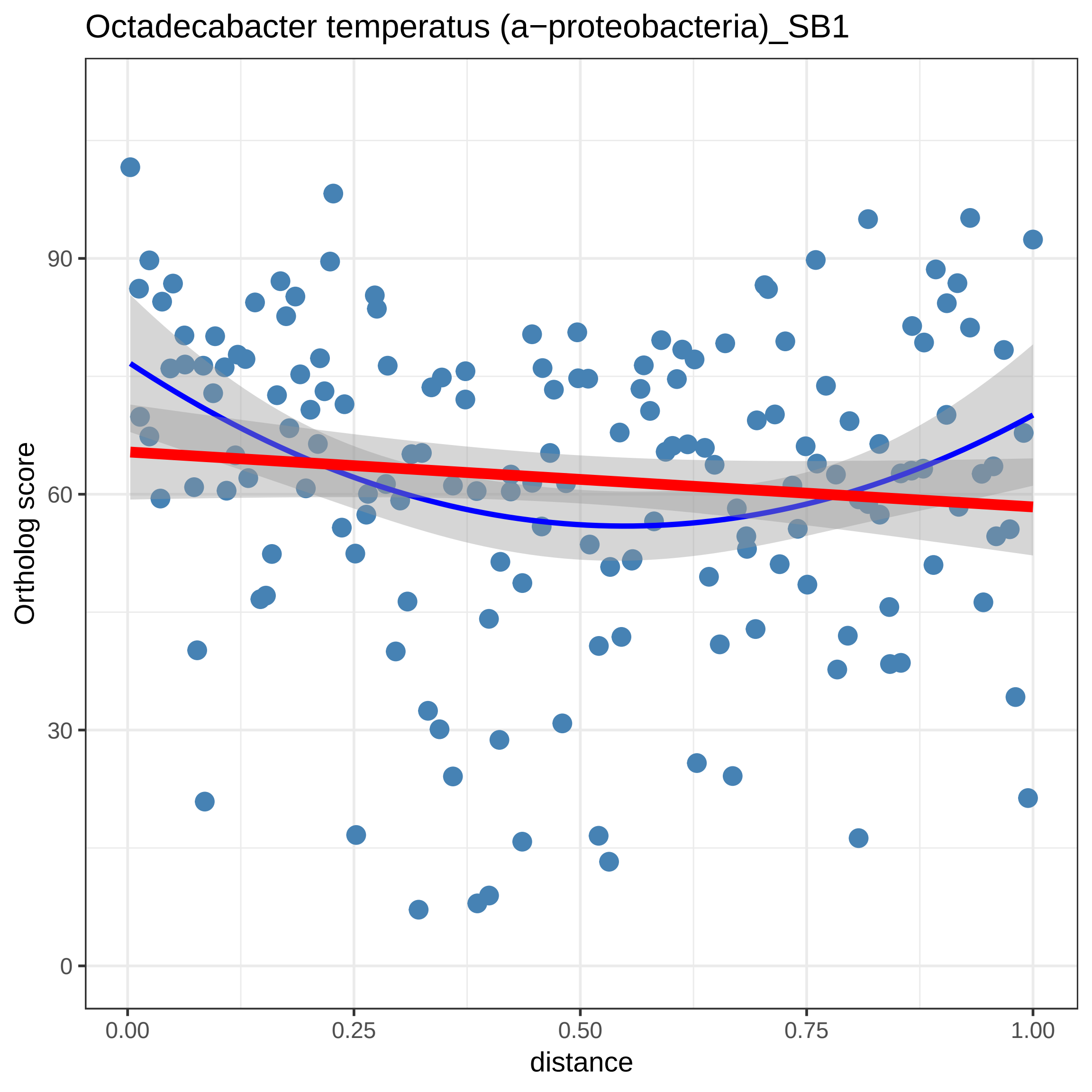

Octadecabacter temperatus (a−proteobacteria)_SB1
Ortholog score
0
30
60
90
0.00
0.25
0.50
0.75
1.00
distance

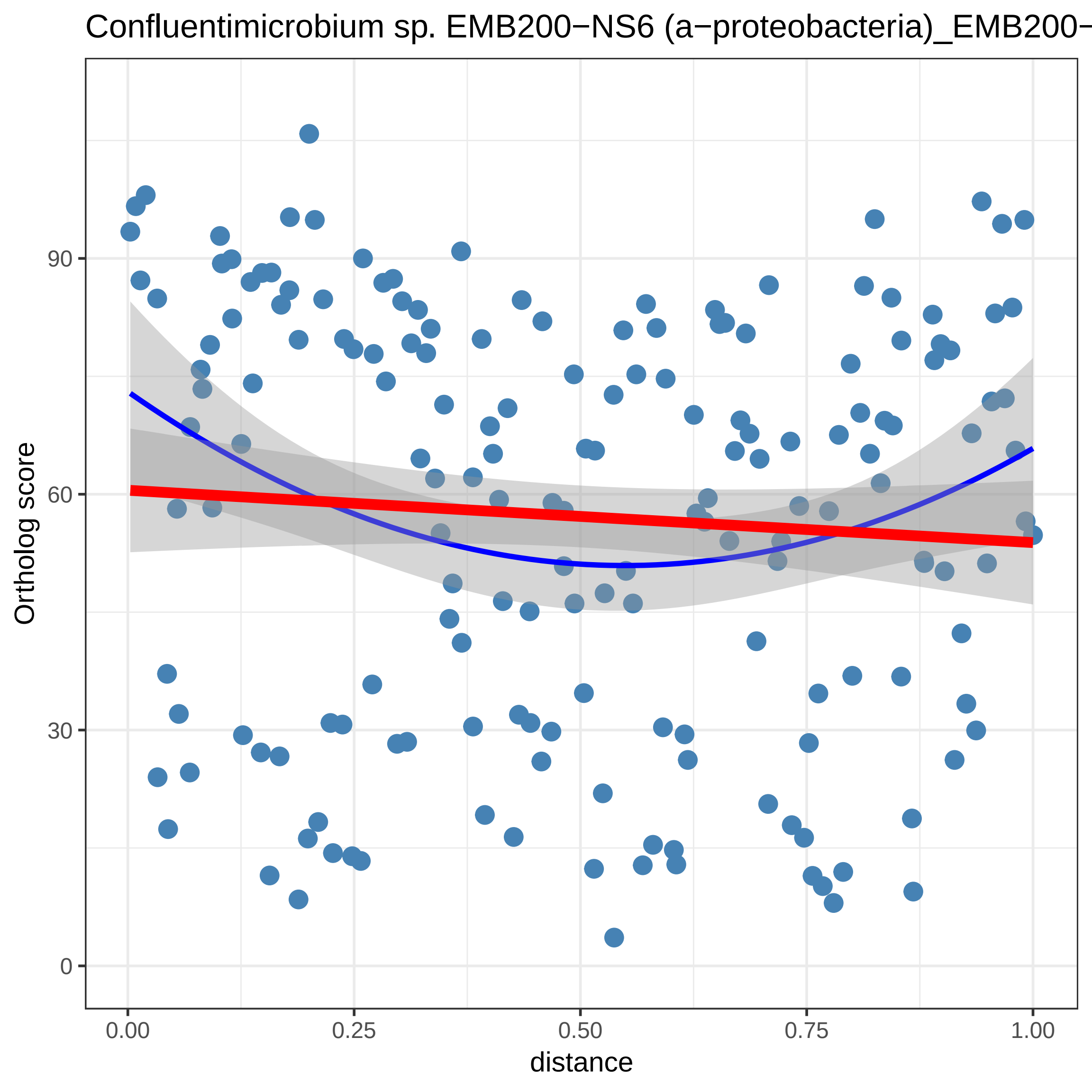

Confluentimicrobium sp. EMB200−NS6 (a−proteobacteria)_EMB200−
Ortholog score
0
30
60
90
distance
0.00
0.25
0.50
0.75
1.00

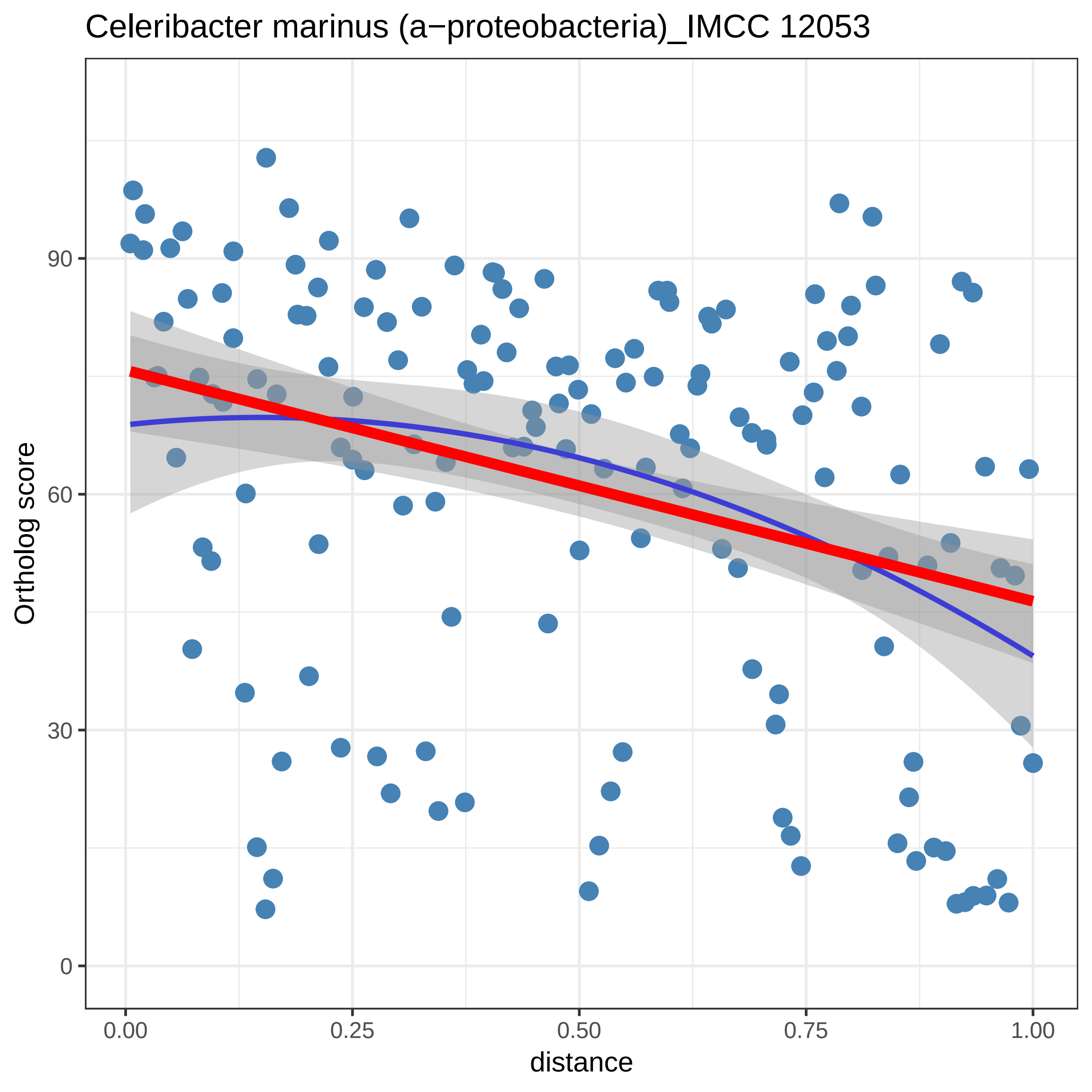

Celeribacter marinus (a−proteobacteria)_IMCC 12053
Ortholog score
distance
0
30
60
90
0.00
0.25
0.50
0.75
1.00

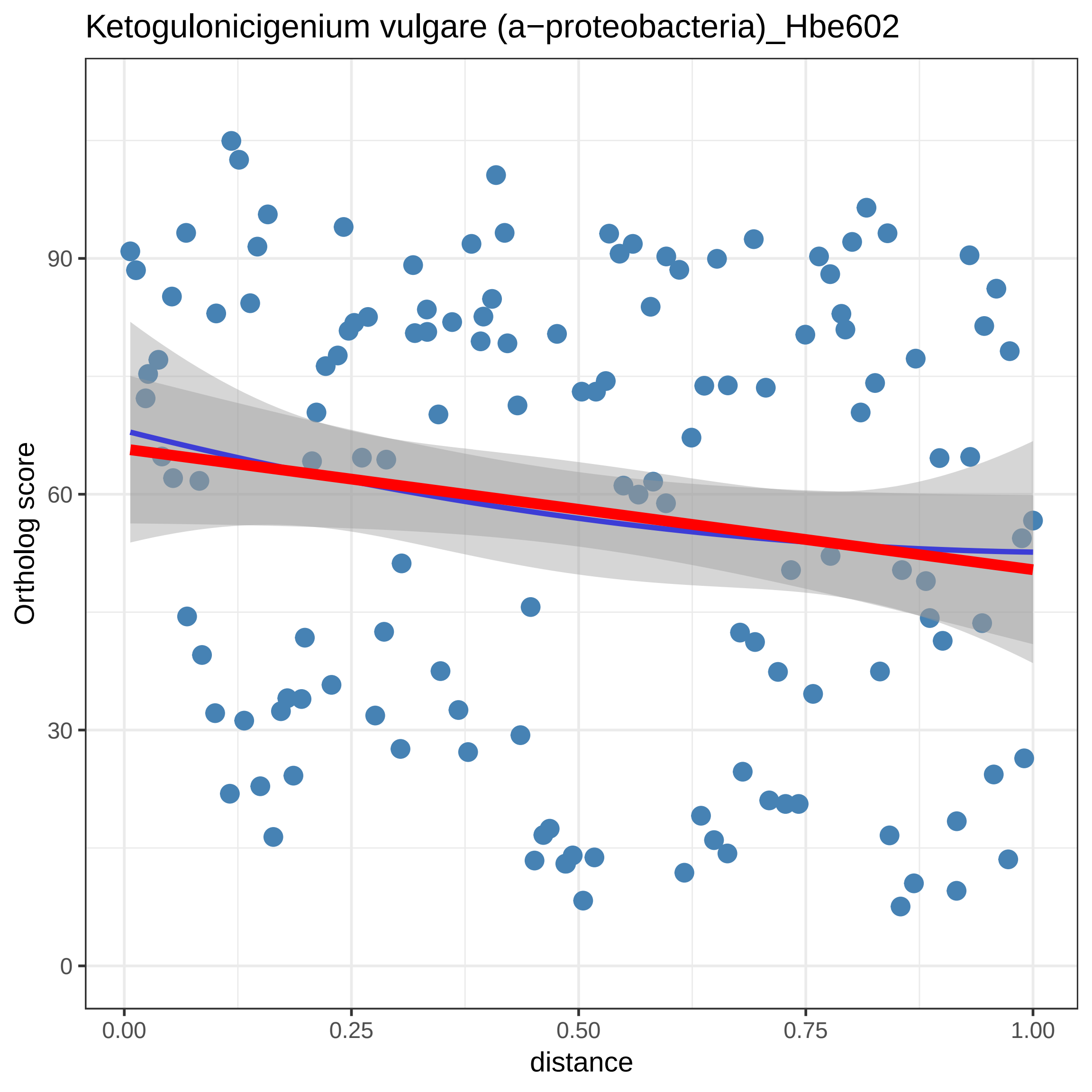

Ketogulonicigenium vulgare (a−proteobacteria)_Hbe602
Ortholog score
0
30
60
90
0.00
0.25
0.50
0.75
1.00
distance

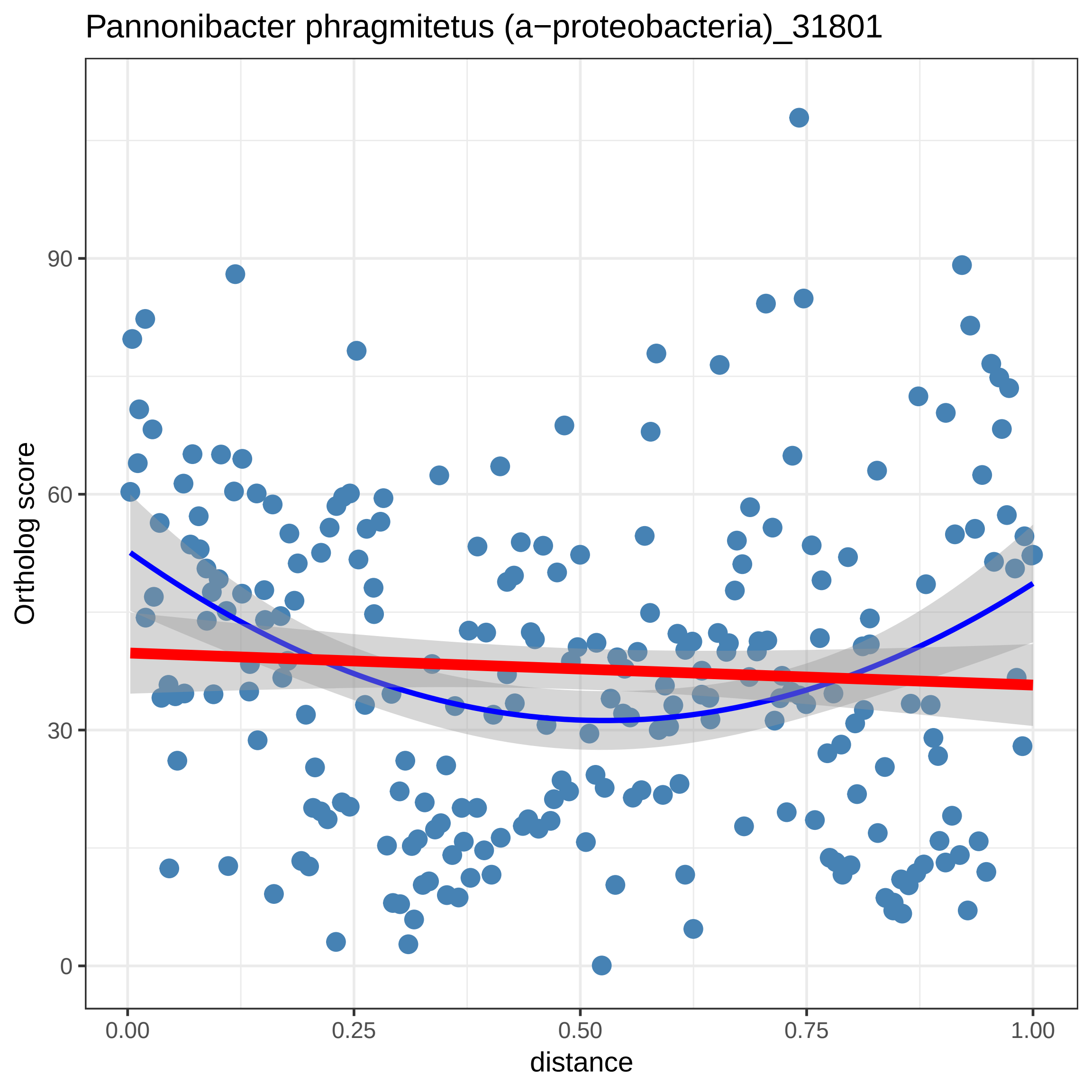

Pannonibacter phragmitetus (a−proteobacteria)_31801
Ortholog score
0
30
60
90
0.00
0.25
0.50
0.75
1.00
distance

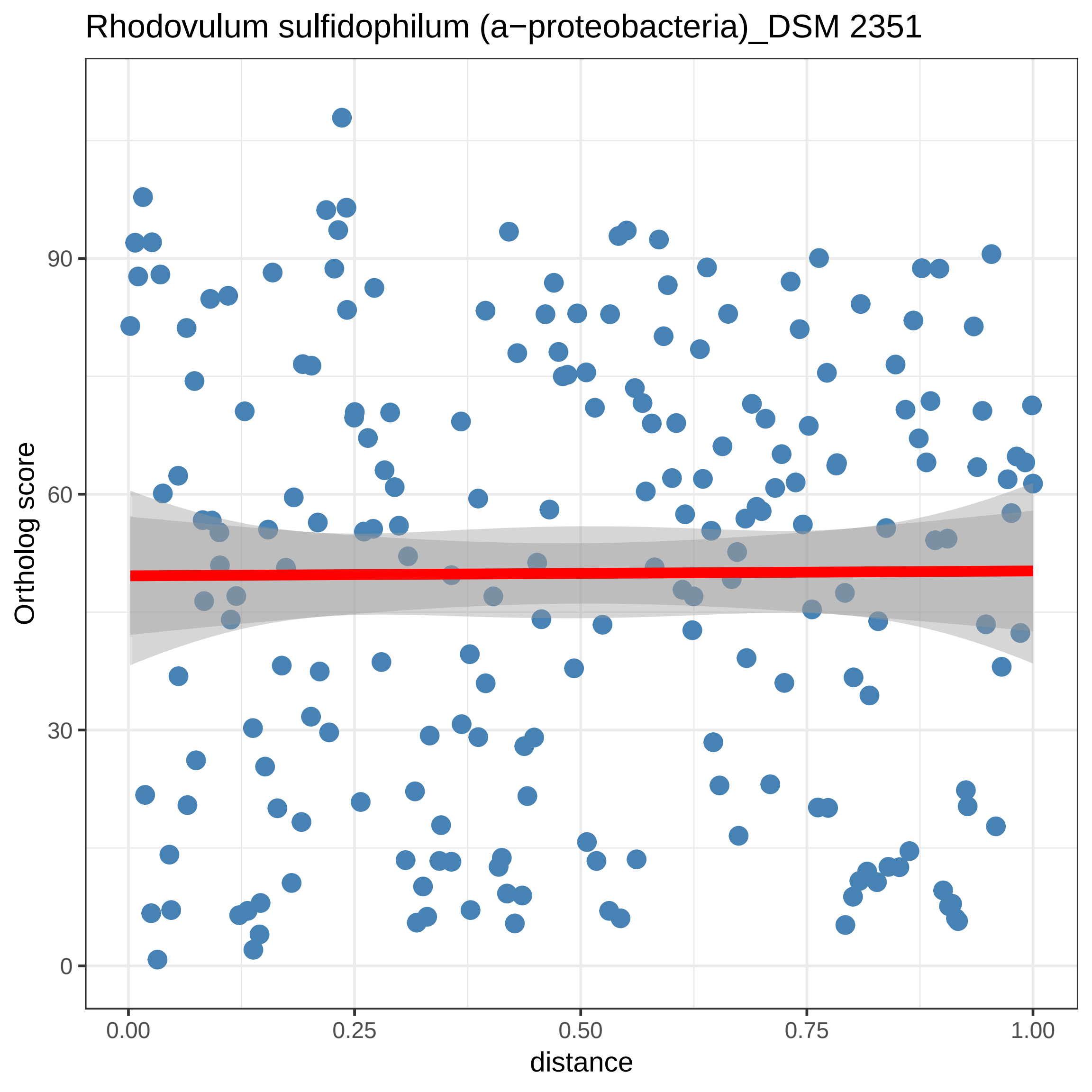

Rhodovulum sulfidophilum (a−proteobacteria)_DSM 2351
Ortholog score
90
60
30
0
0.00
0.25
0.50
0.75
1.00
distance

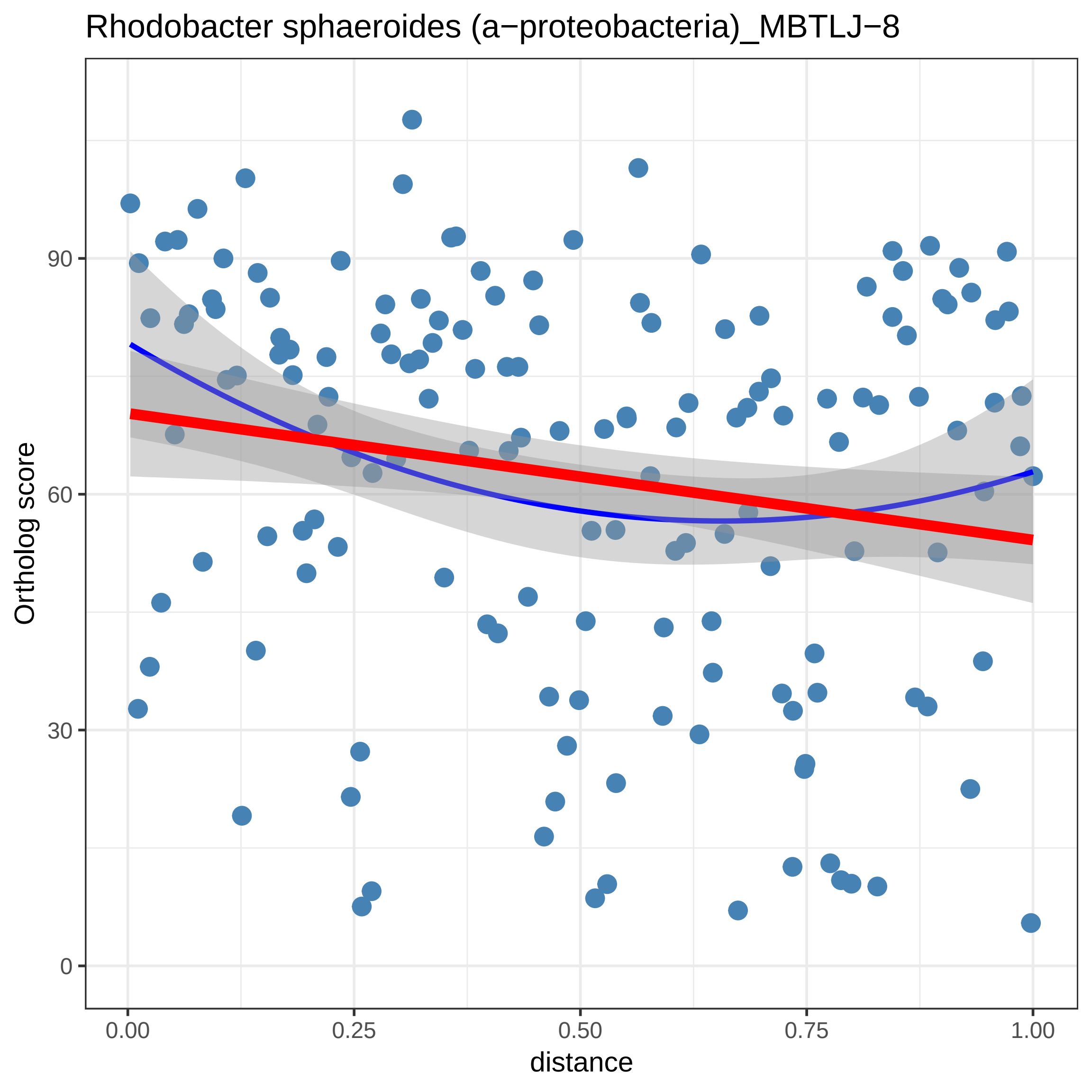

Rhodobacter sphaeroides (a−proteobacteria)_MBTLJ−8
Ortholog score
0
30
60
90
0.00
0.25
0.50
0.75
1.00
distance

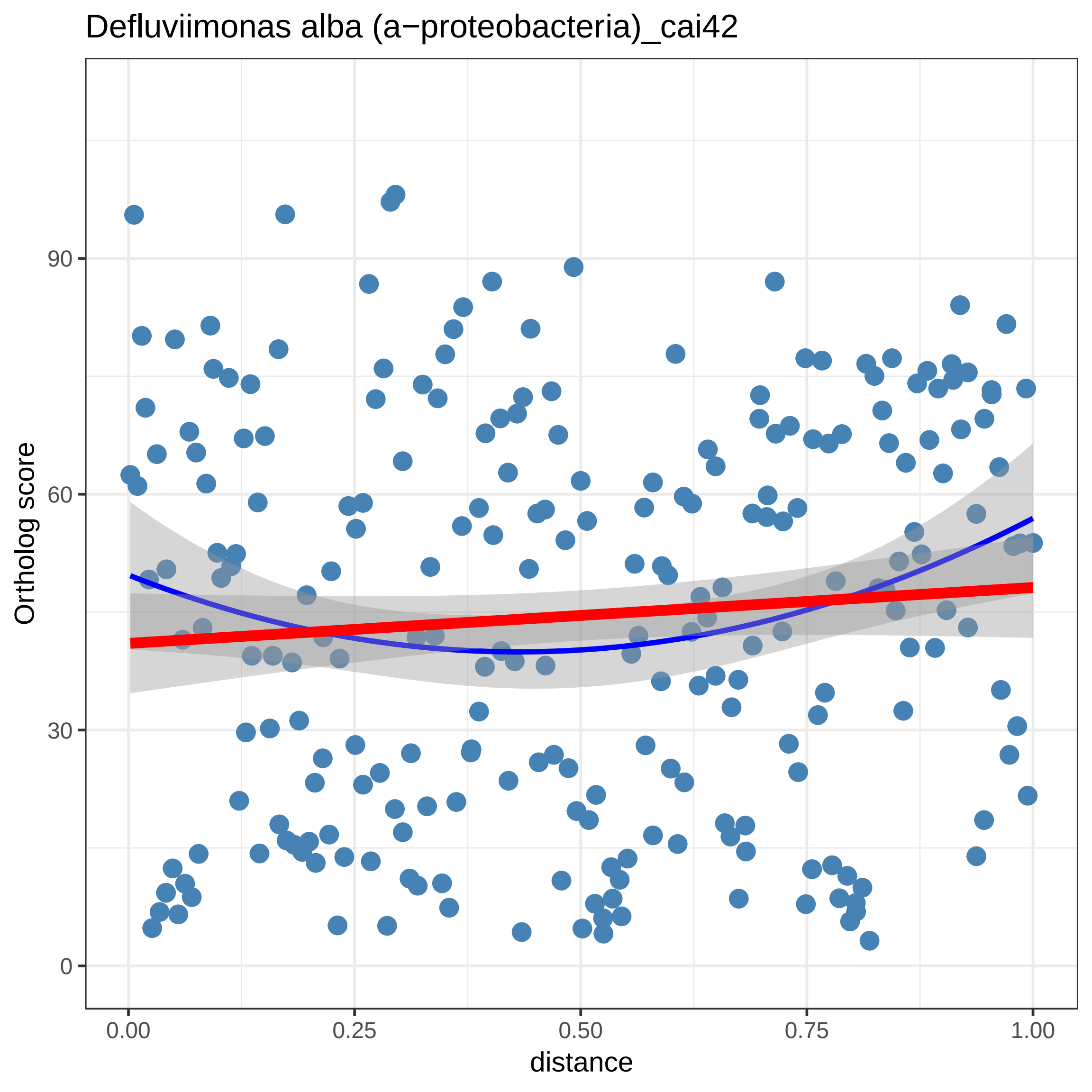

Defluviimonas alba (a−proteobacteria)_cai42
Ortholog score
0
30
60
90
0.00
0.25
0.50
0.75
1.00
distance

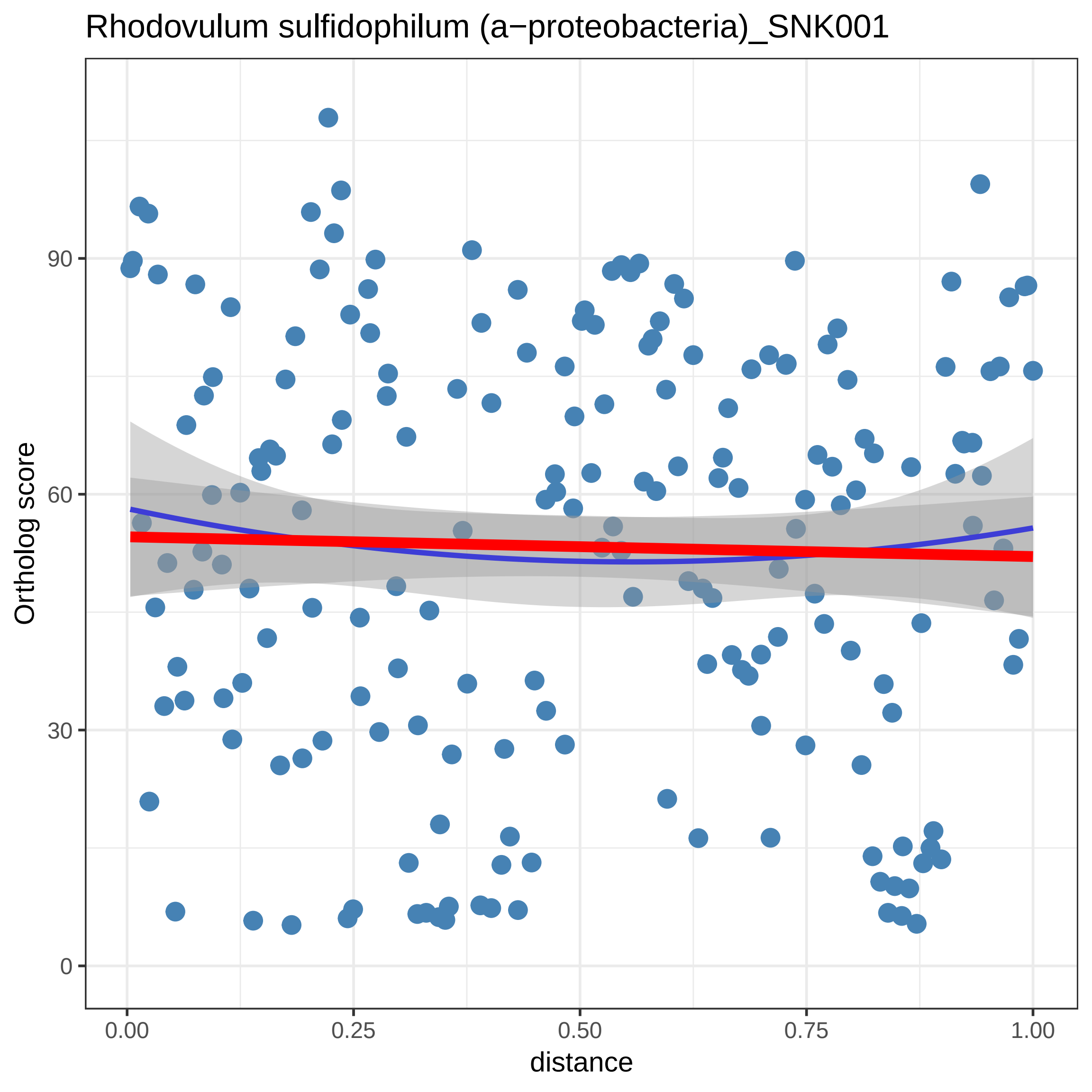
Rhodovulum sulfidophilum (a−proteobacteria)_SNK001
Ortholog score
90
60
30
0
0.00
0.25
0.50
0.75
1.00
distance

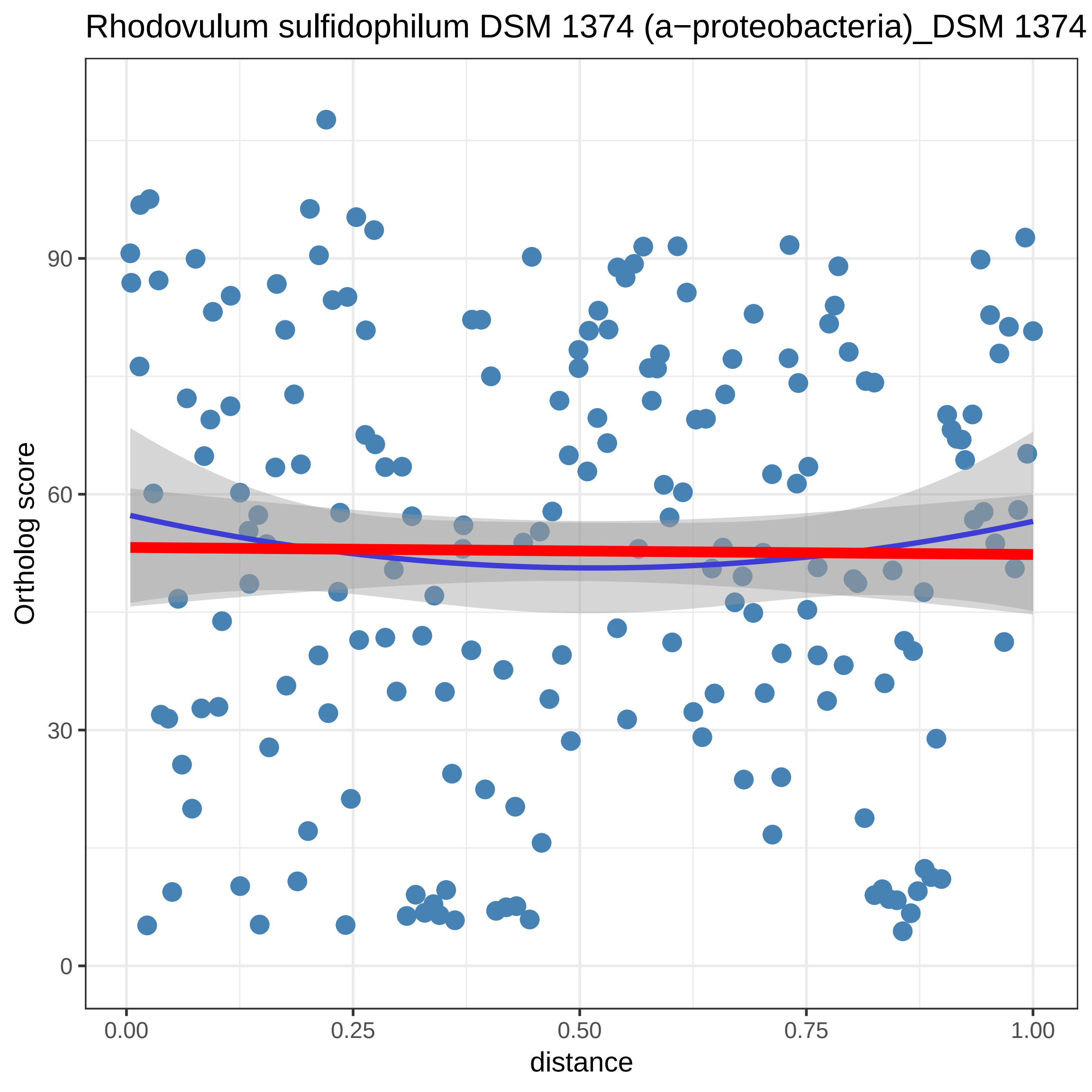

Rhodovulum sulfidophilum DSM 1374 (a−proteobacteria)_DSM 1374
Ortholog score
90
60
30
0
0.00
0.25
0.50
0.75
1.00
distance

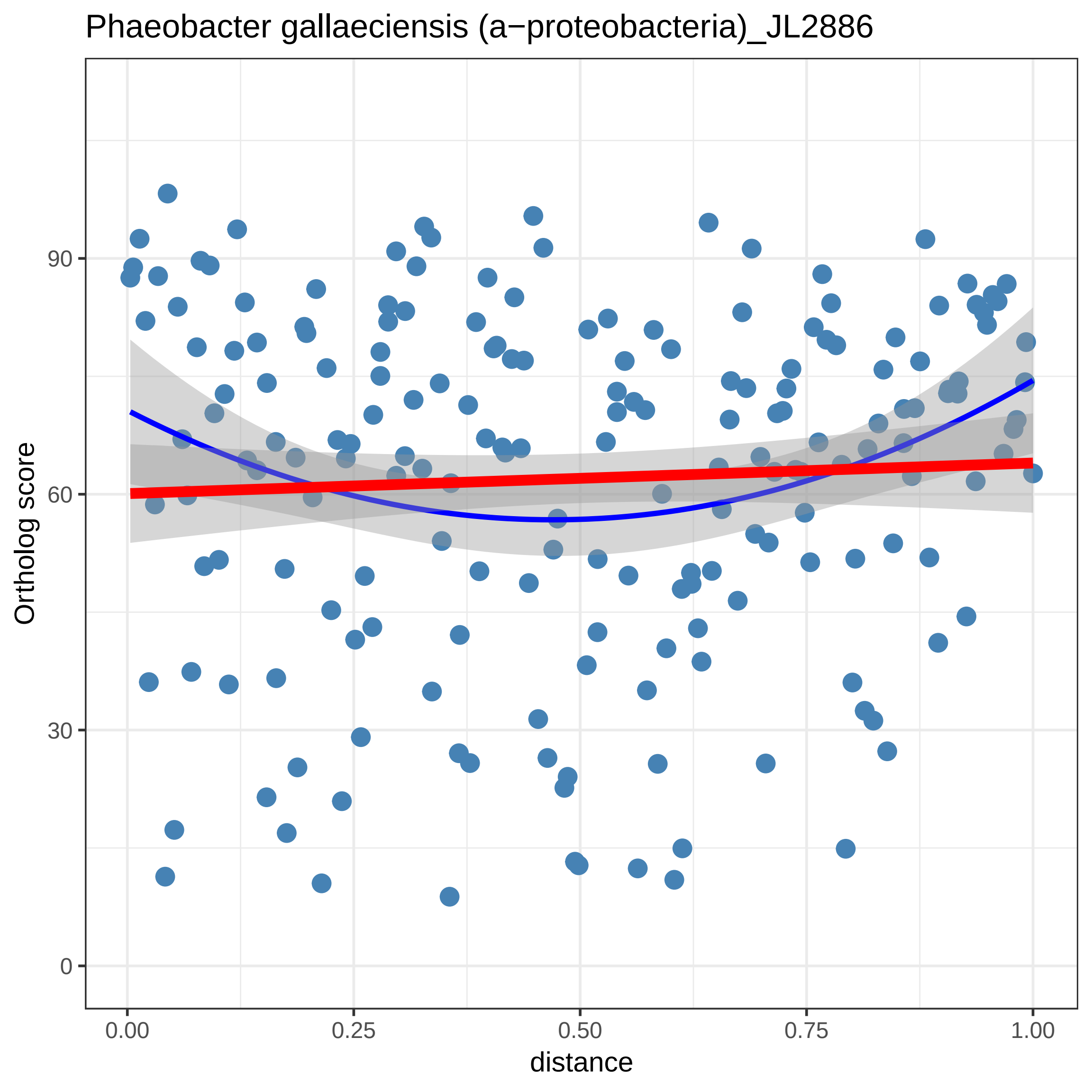

Phaeobacter gallaeciensis (a−proteobacteria)_JL2886
Ortholog score
90
60
30
0
0.00
0.25
0.50
0.75
1.00
distance

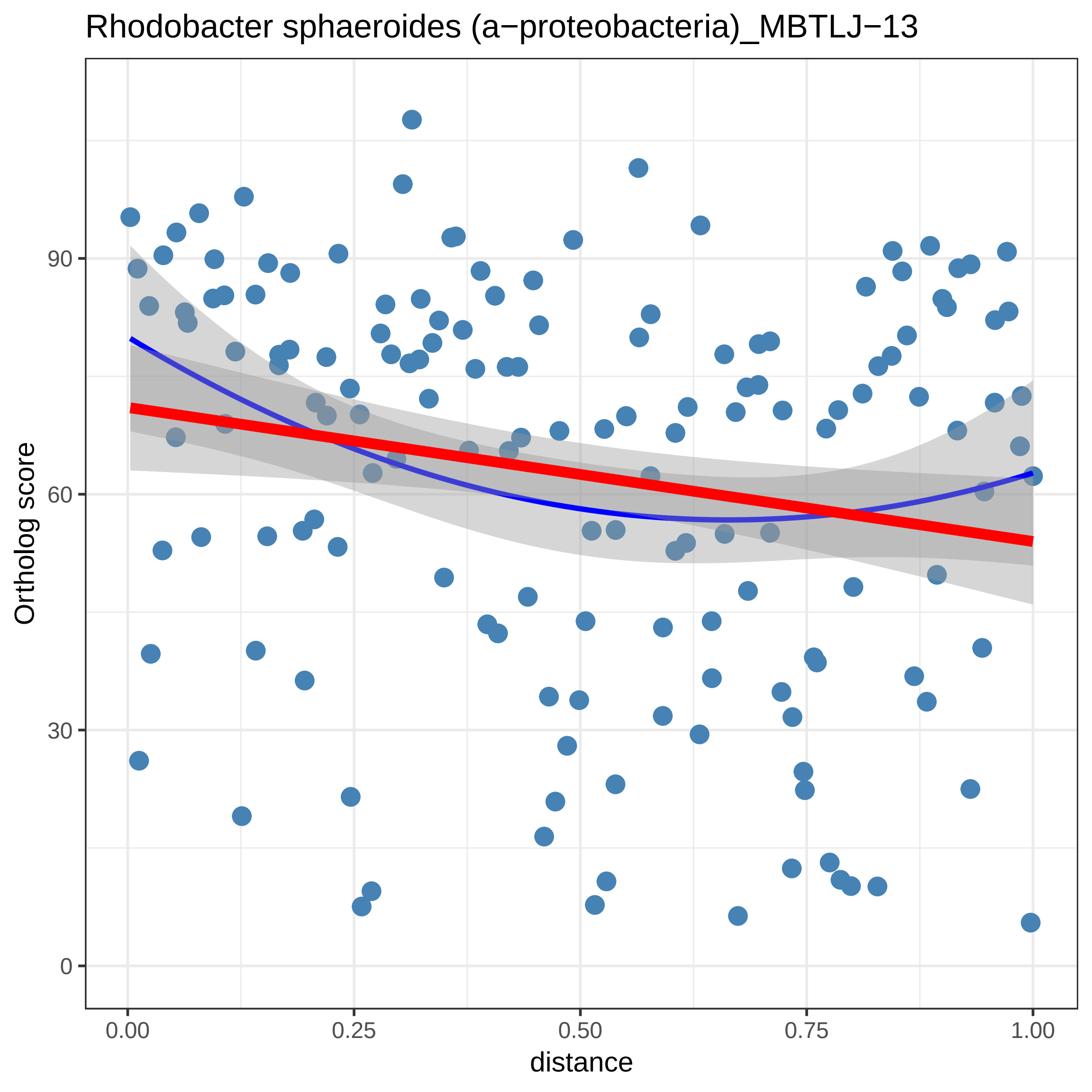

Rhodobacter sphaeroides (a−proteobacteria)_MBTLJ−13
Ortholog score
90
60
30
0
0.00
0.25
0.50
0.75
1.00
distance

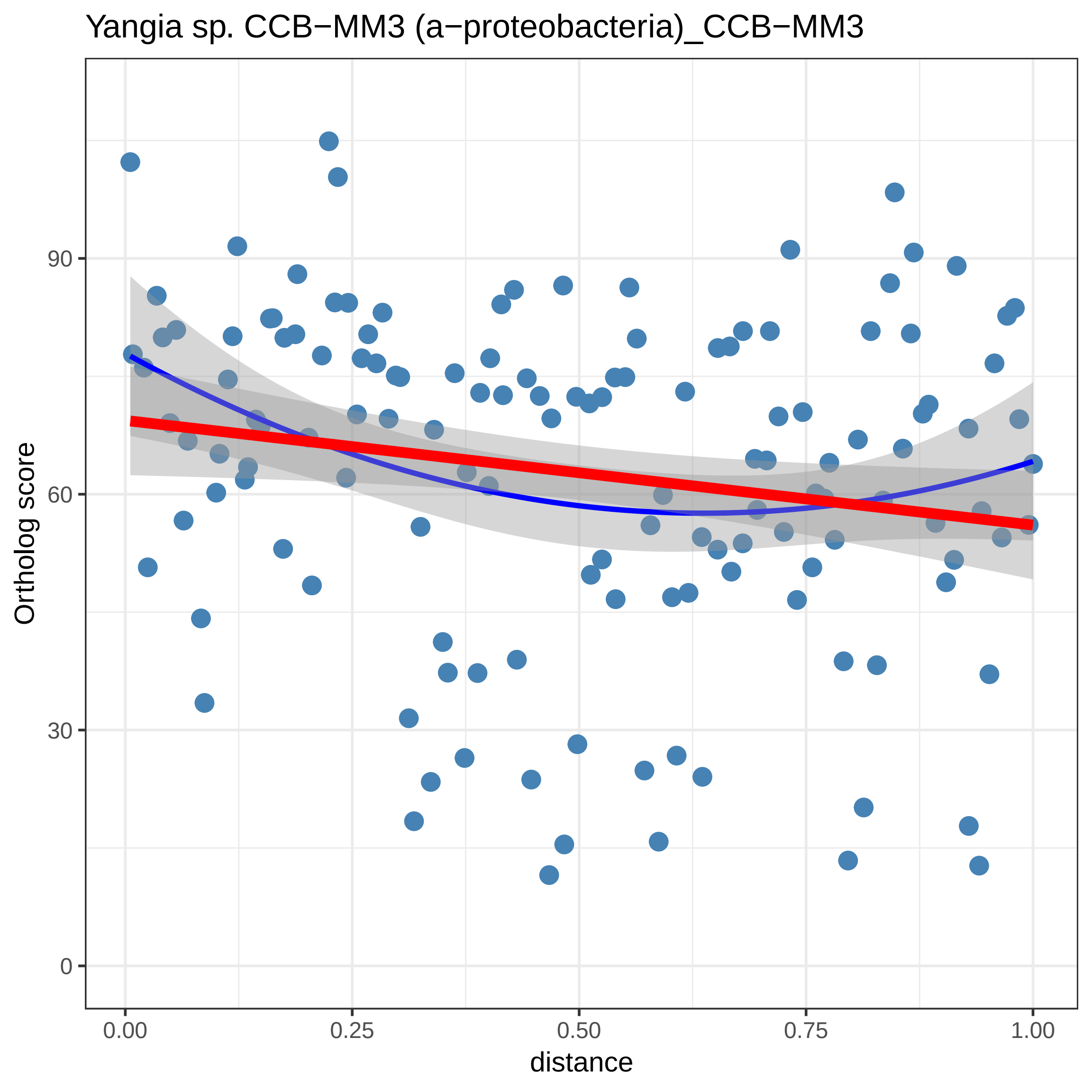

Yangia sp. CCB−MM3 (a−proteobacteria)_CCB−MM3
Ortholog score
90
60
30
0
0.00
0.25
0.50
0.75
1.00
distance

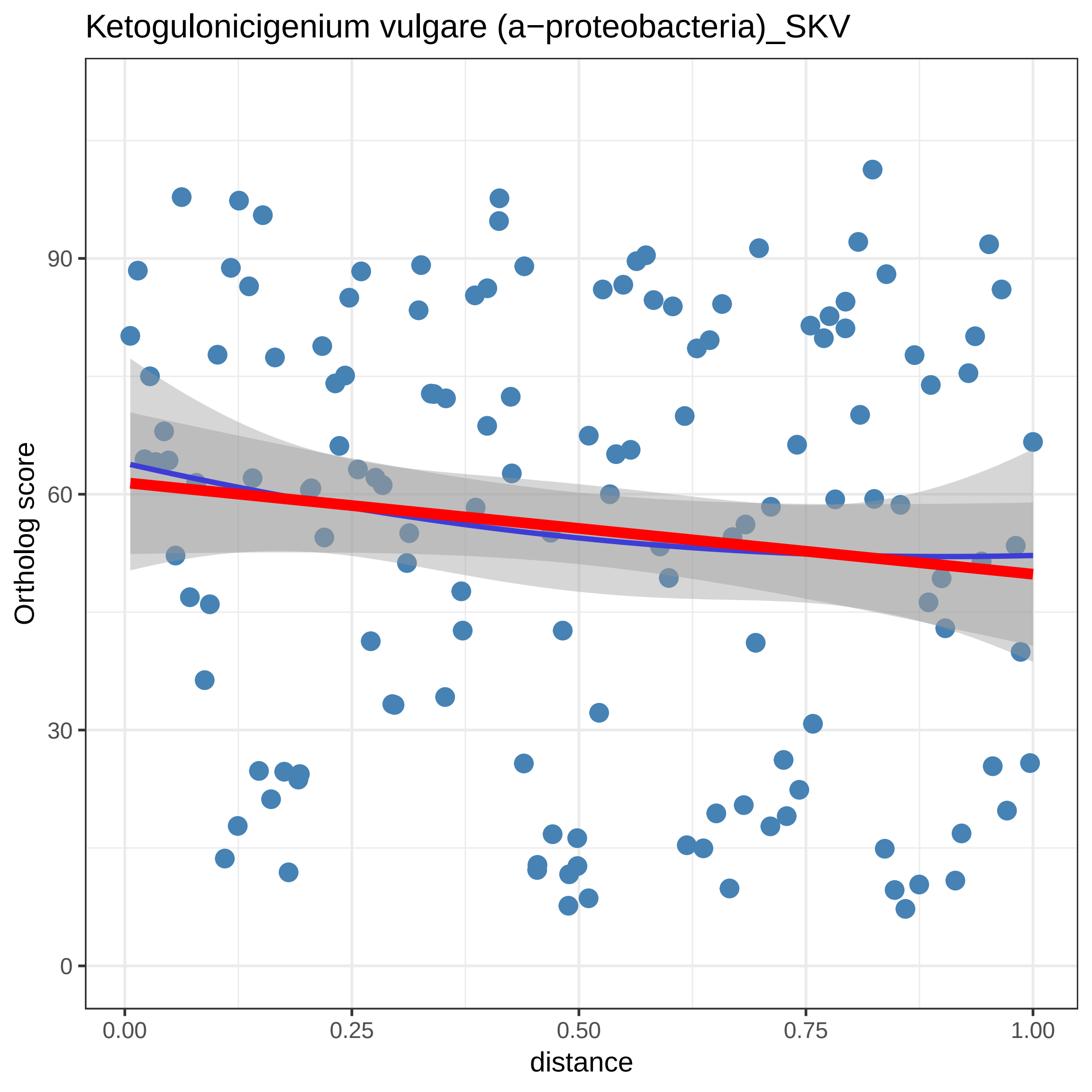
Ketogulonicigenium vulgare (a−proteobacteria)_SKV
Ortholog score
0
30
60
90
distance
0.00
0.25
0.50
0.75
1.00

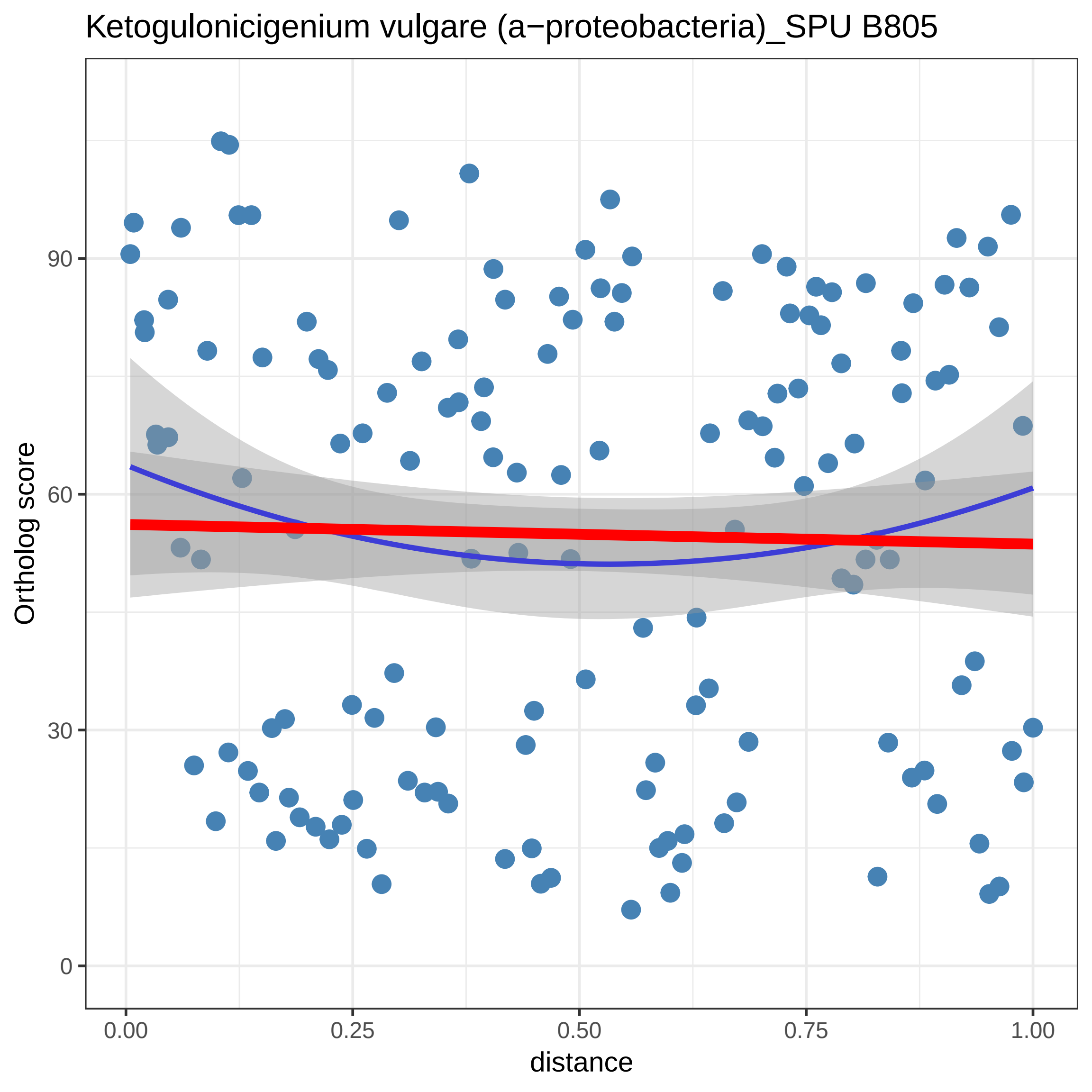
Ketogulonicigenium vulgare (a−proteobacteria)_SPU B805
Ortholog score
0
30
60
90
0.00
0.25
0.50
0.75
1.00
distance

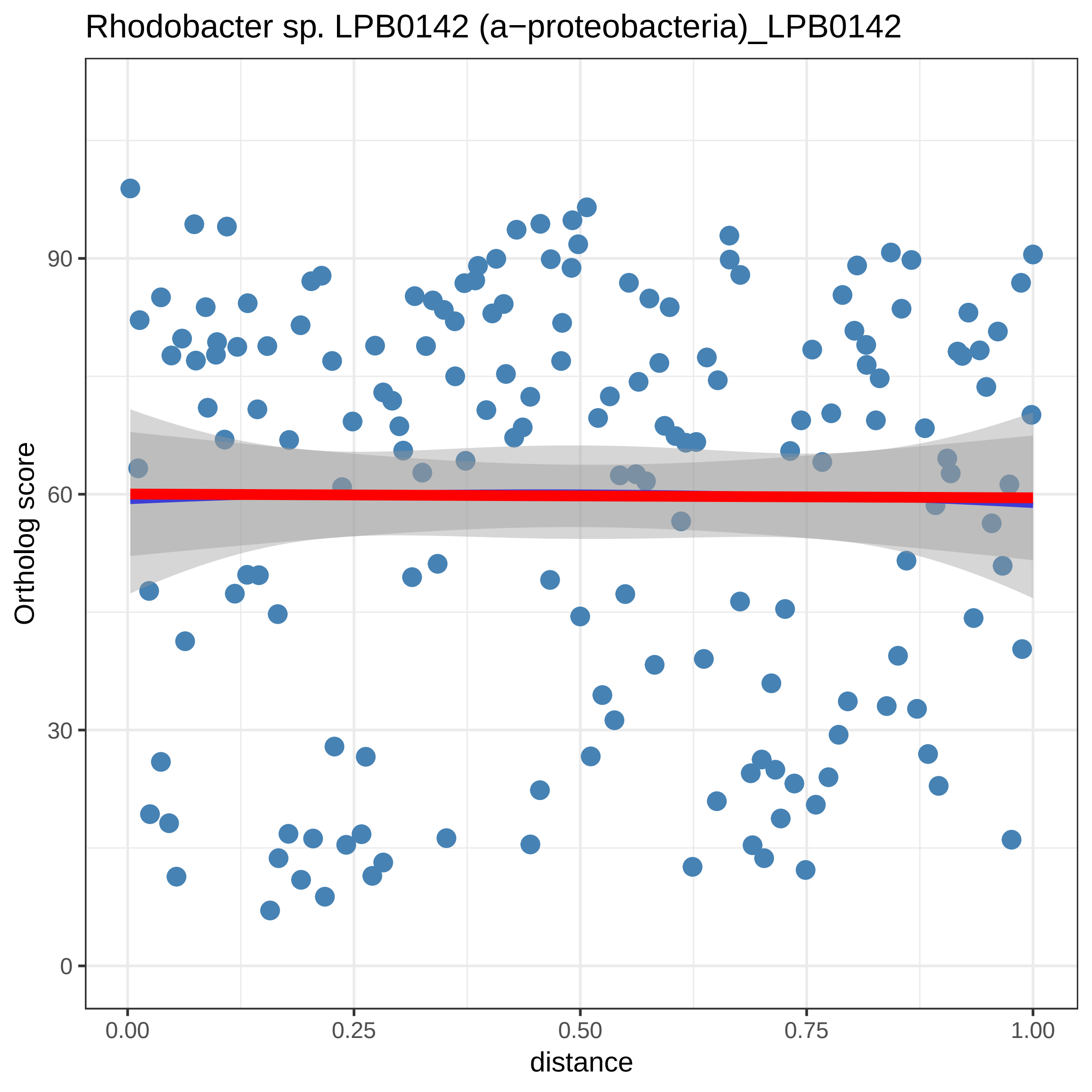

Rhodobacter sp. LPB0142 (a−proteobacteria)_LPB0142
Ortholog score
distance
0
30
60
90
0.00
0.25
0.50
0.75
1.00

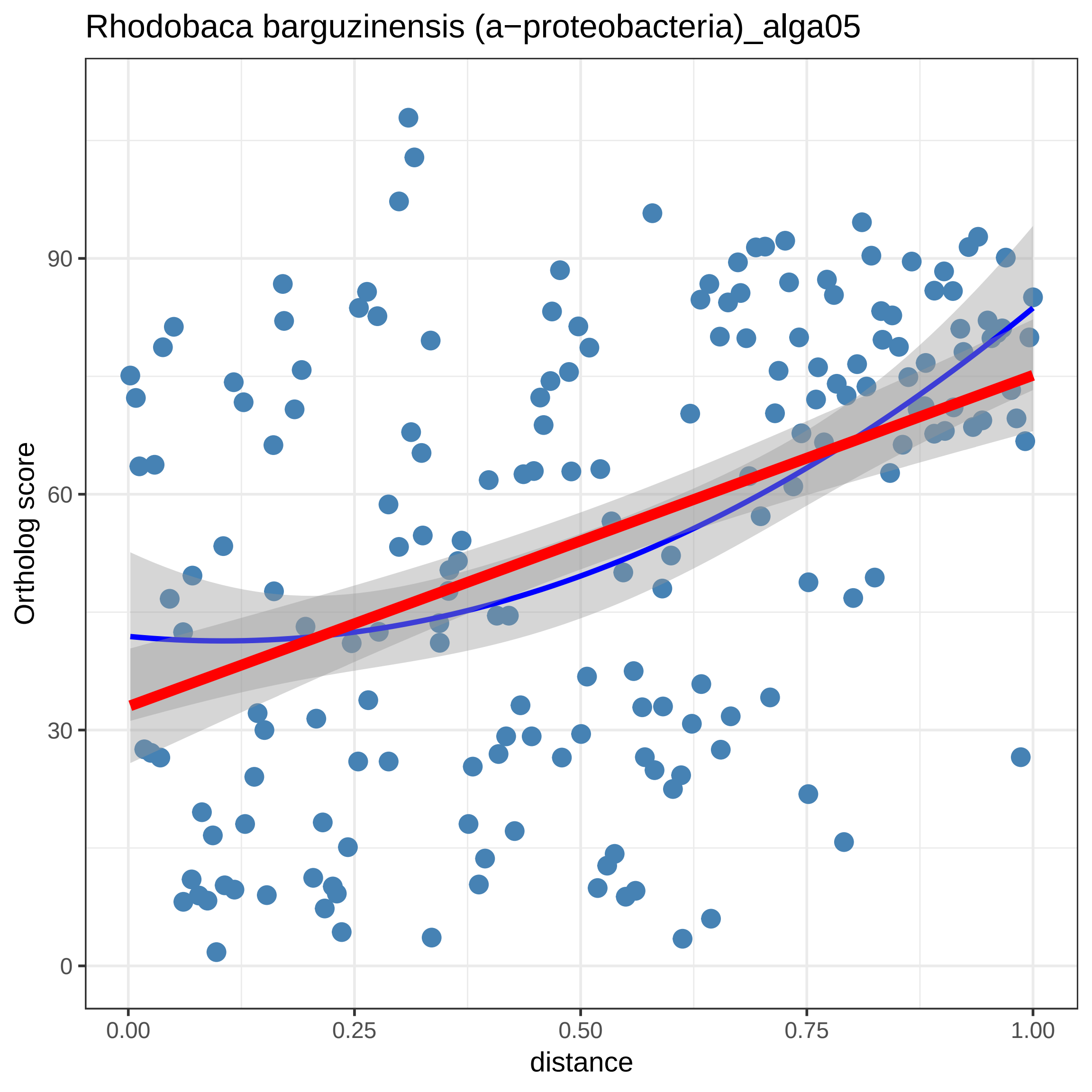

Rhodobaca barguzinensis (a−proteobacteria)_alga05
Ortholog score
90
60
30
0
0.00
0.25
0.50
0.75
1.00
distance

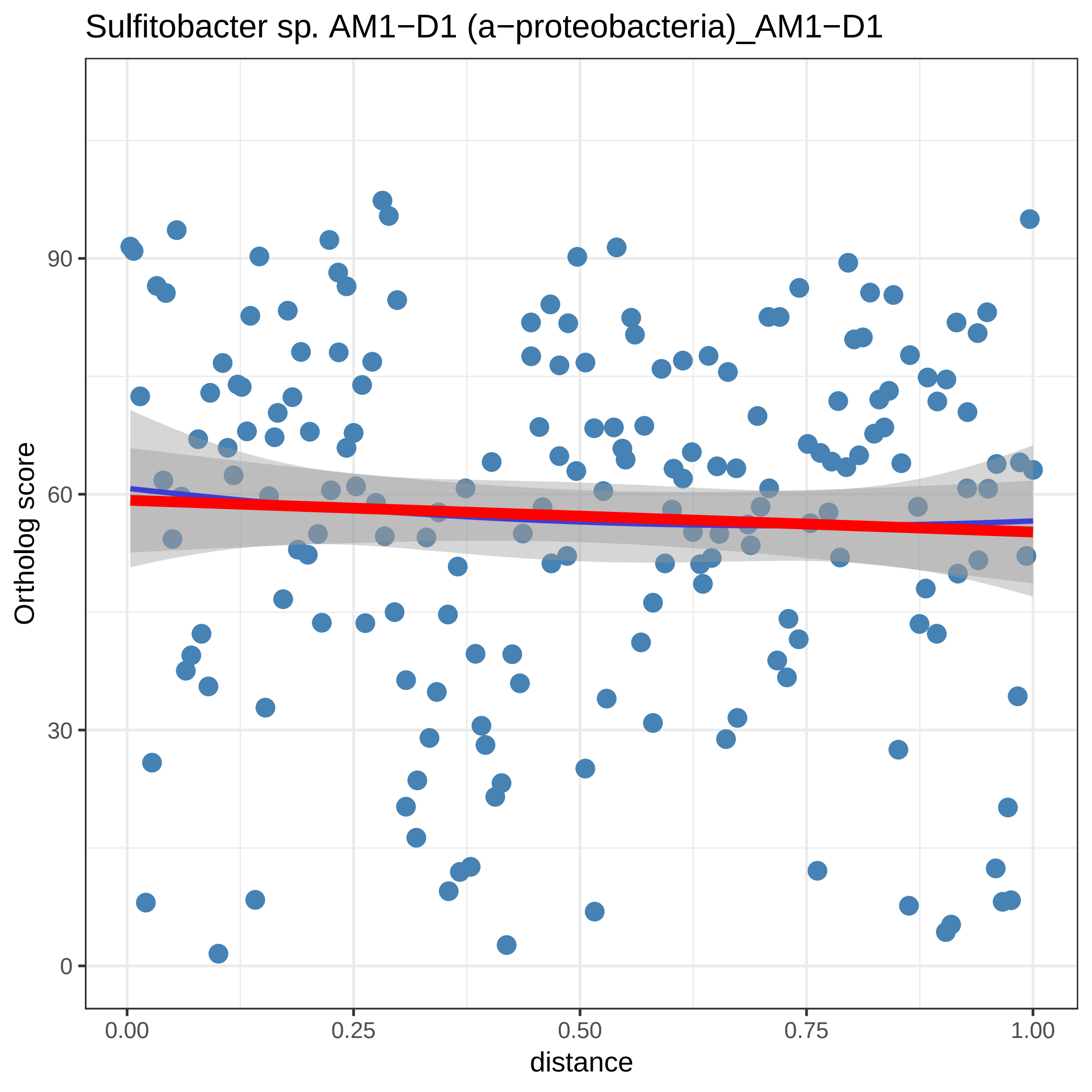
Sulfitobacter sp. AM1−D1 (a−proteobacteria)_AM1−D1
Ortholog score
0
30
60
90
distance
0.00
0.25
0.50
0.75
1.00

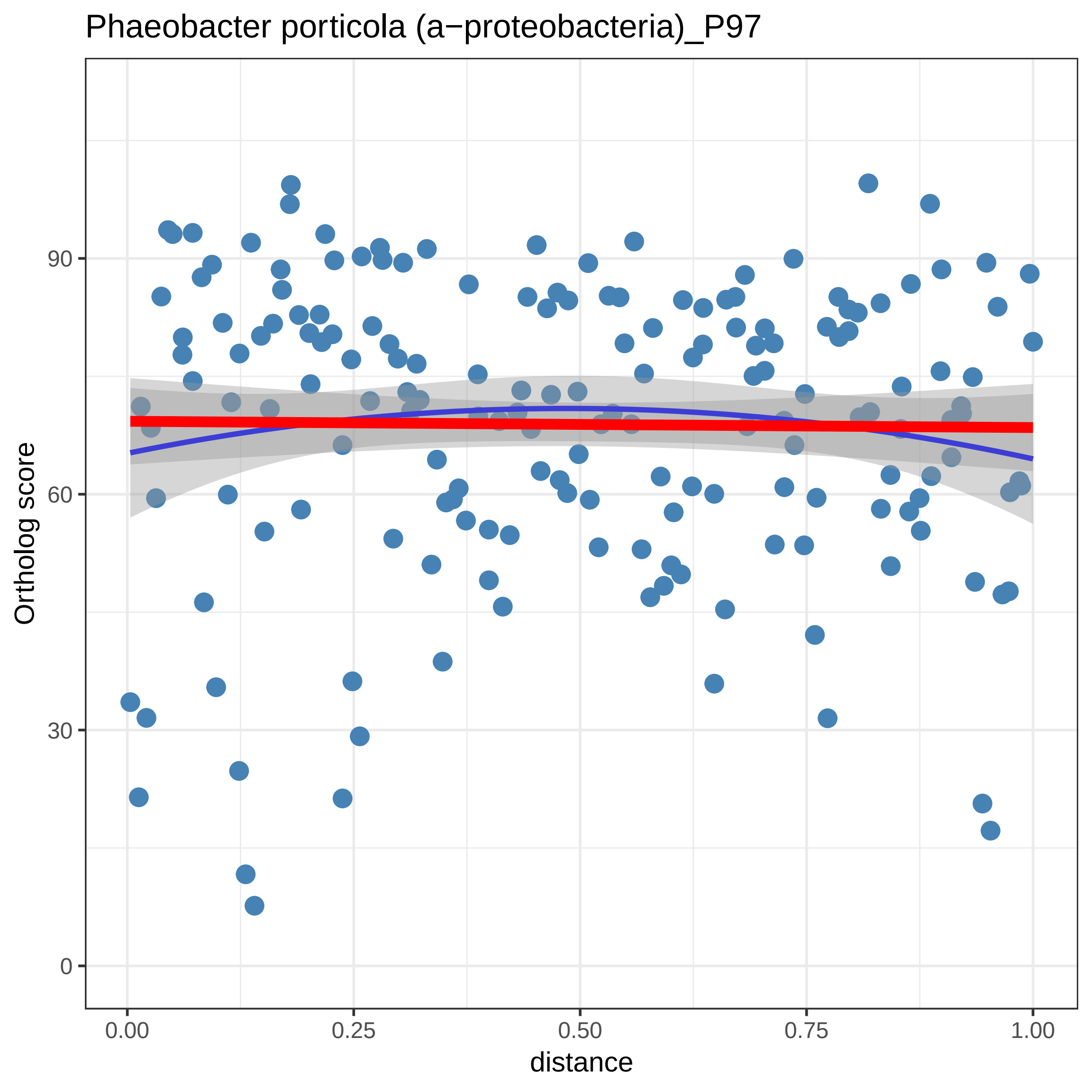

Phaeobacter porticola (a−proteobacteria)_P97
Ortholog score
distance
0
30
60
90
0.00
0.25
0.50
0.75
1.00

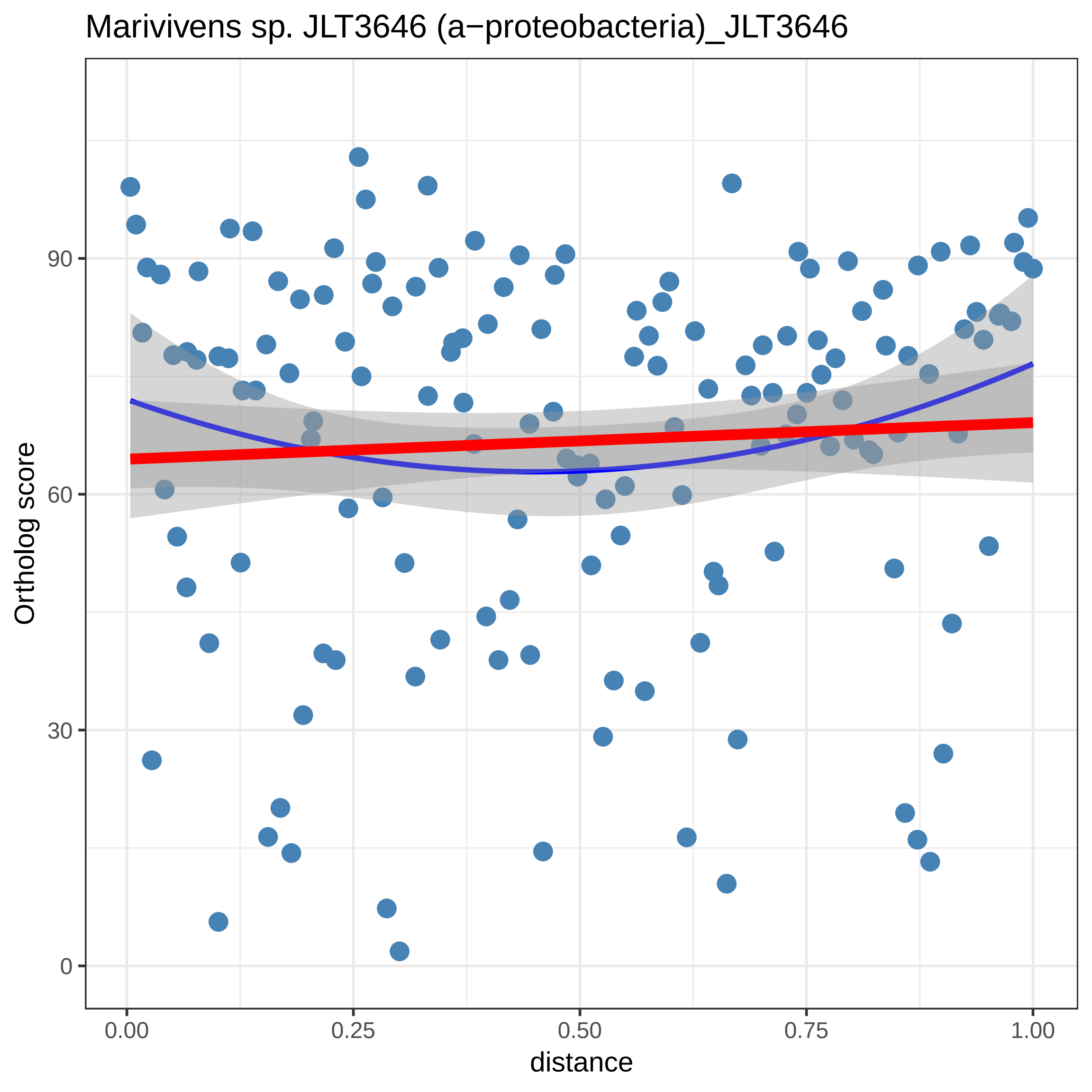

Marivivens sp. JLT3646 (a−proteobacteria)_JLT3646
Ortholog score
90
60
30
0
0.00
0.25
0.50
0.75
1.00
distance

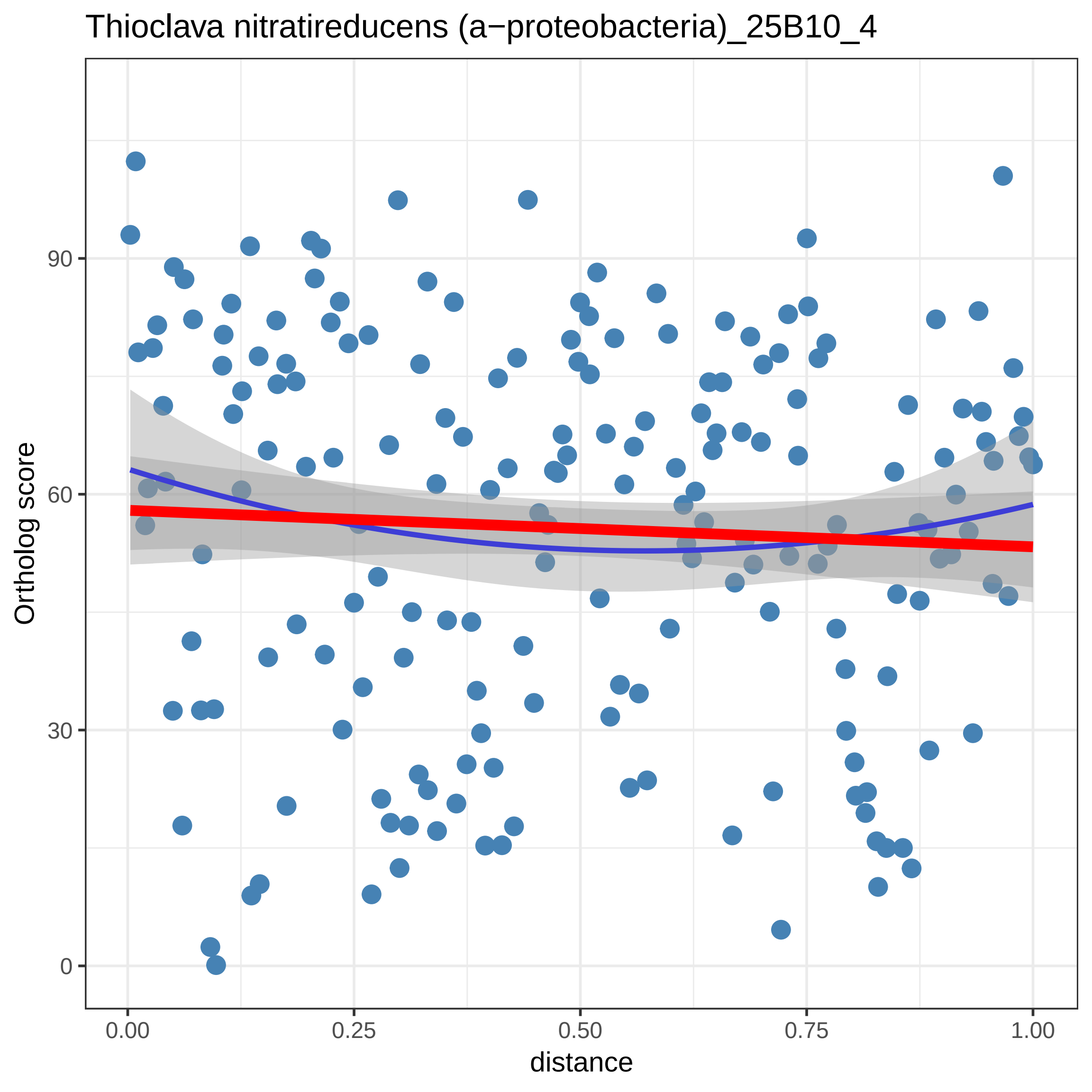
Thioclava nitratireducens (a−proteobacteria)_25B10_4
Ortholog score
distance
0
30
60
90
0.00
0.25
0.50
0.75
1.00

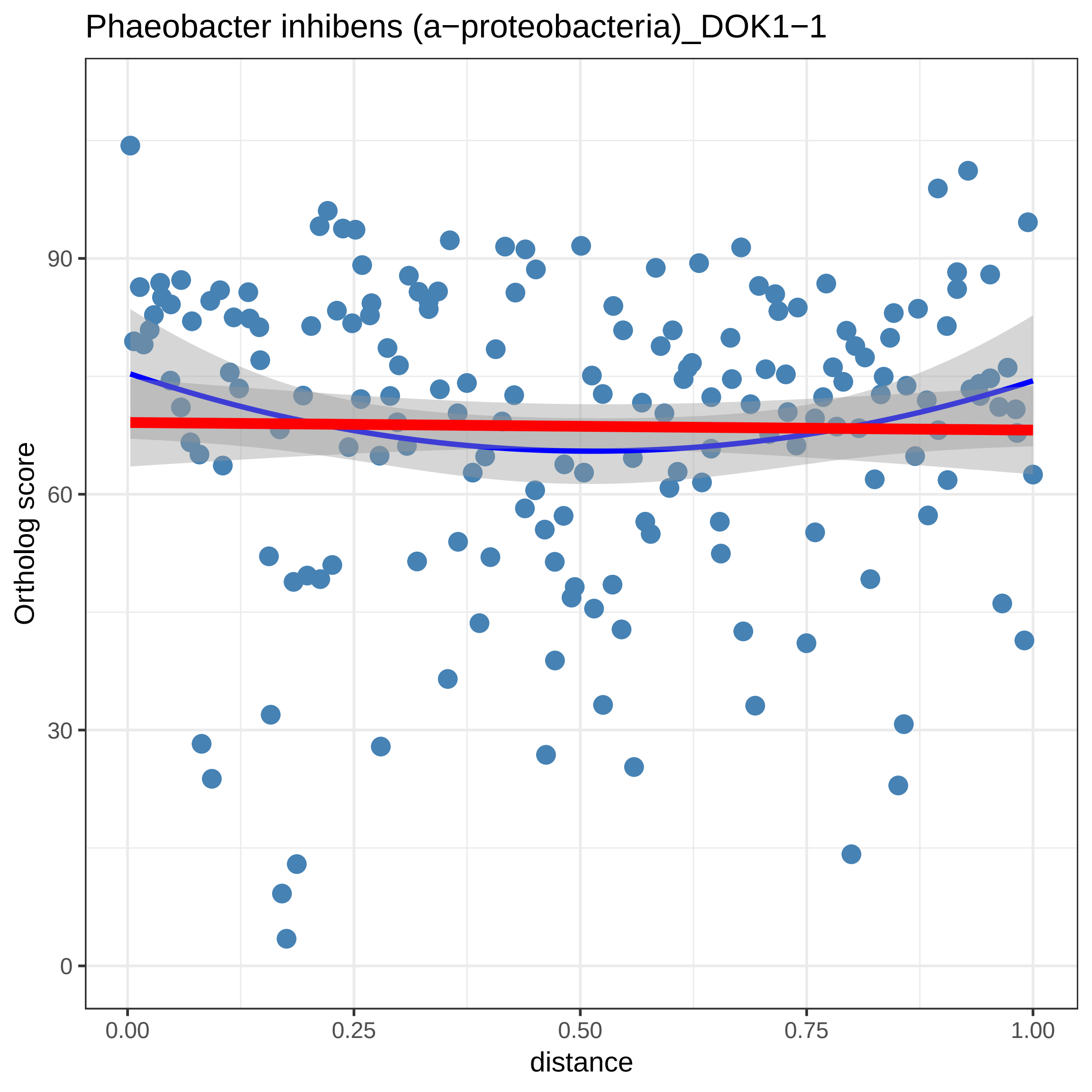

Phaeobacter inhibens (a−proteobacteria)_DOK1−1
Ortholog score
0
30
60
90
0.00
0.25
0.50
0.75
1.00
distance

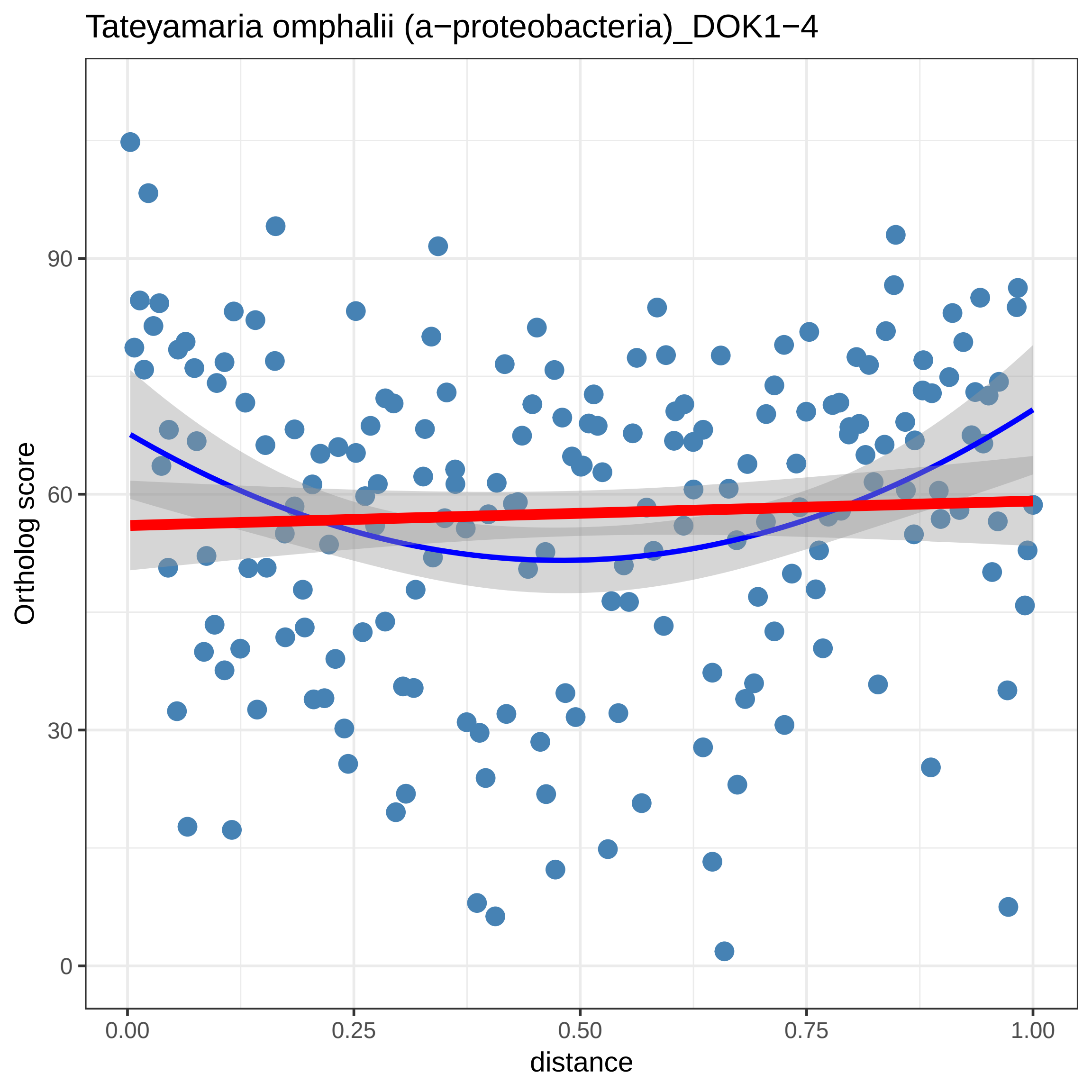

Tateyamaria omphalii (a−proteobacteria)_DOK1−4
Ortholog score
90
60
30
0
0.00
0.25
0.50
0.75
1.00
distance

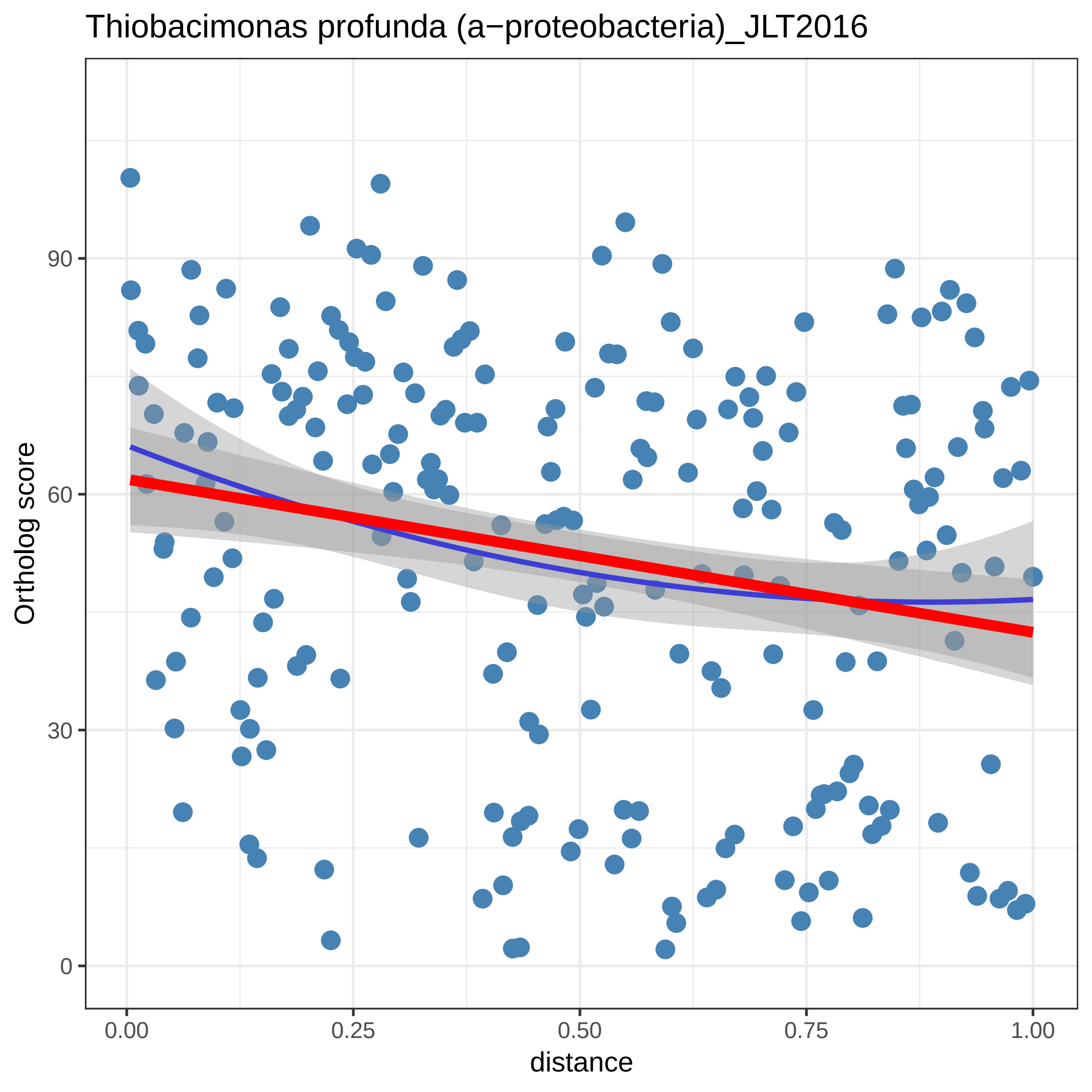
Thiobacimonas profunda (a−proteobacteria)_JLT2016
Ortholog score
90
60
30
0
0.00
0.25
0.50
0.75
1.00
distance

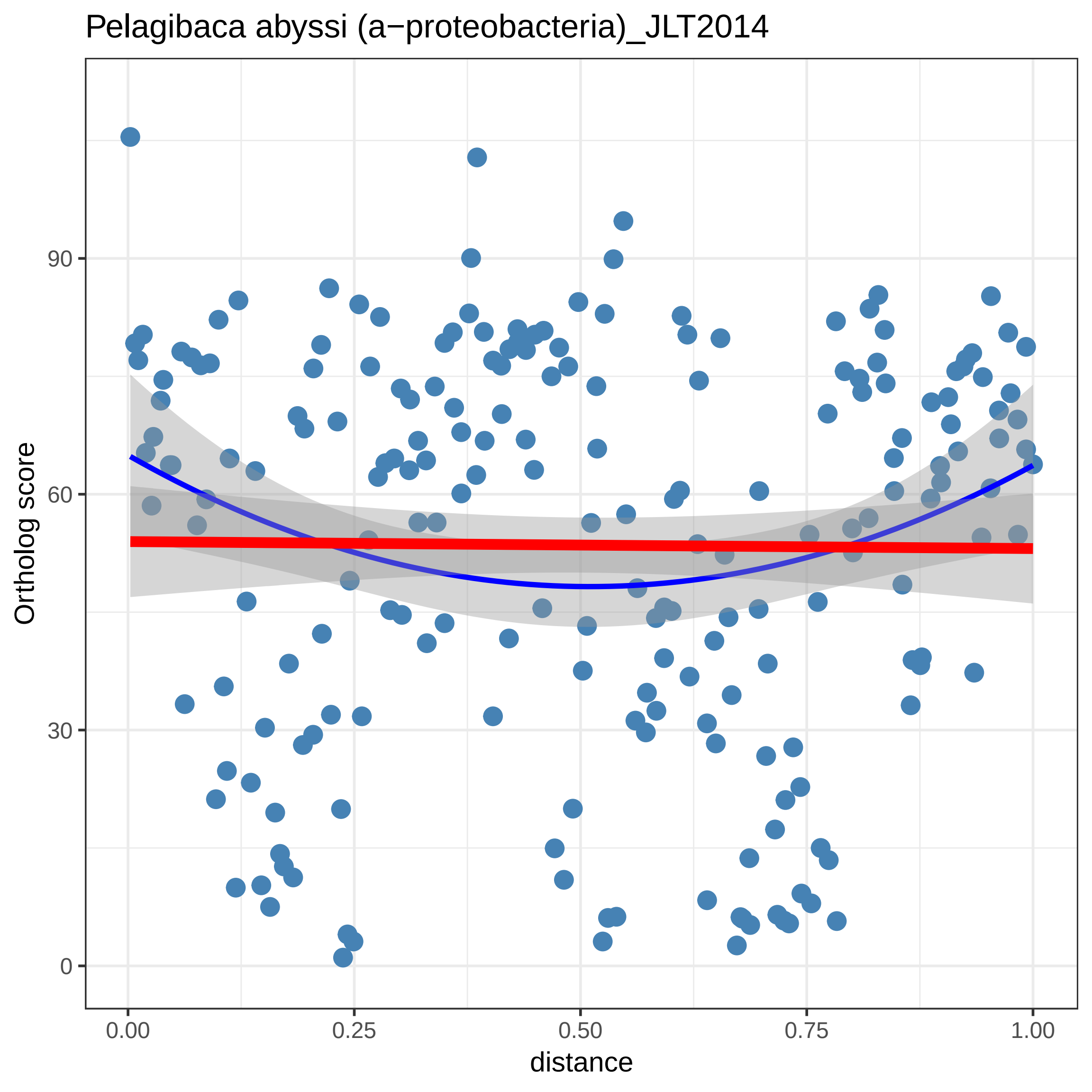

Pelagibaca abyssi (a−proteobacteria)_JLT2014
Ortholog score
90
60
30
0
0.00
0.25
0.50
0.75
1.00
distance

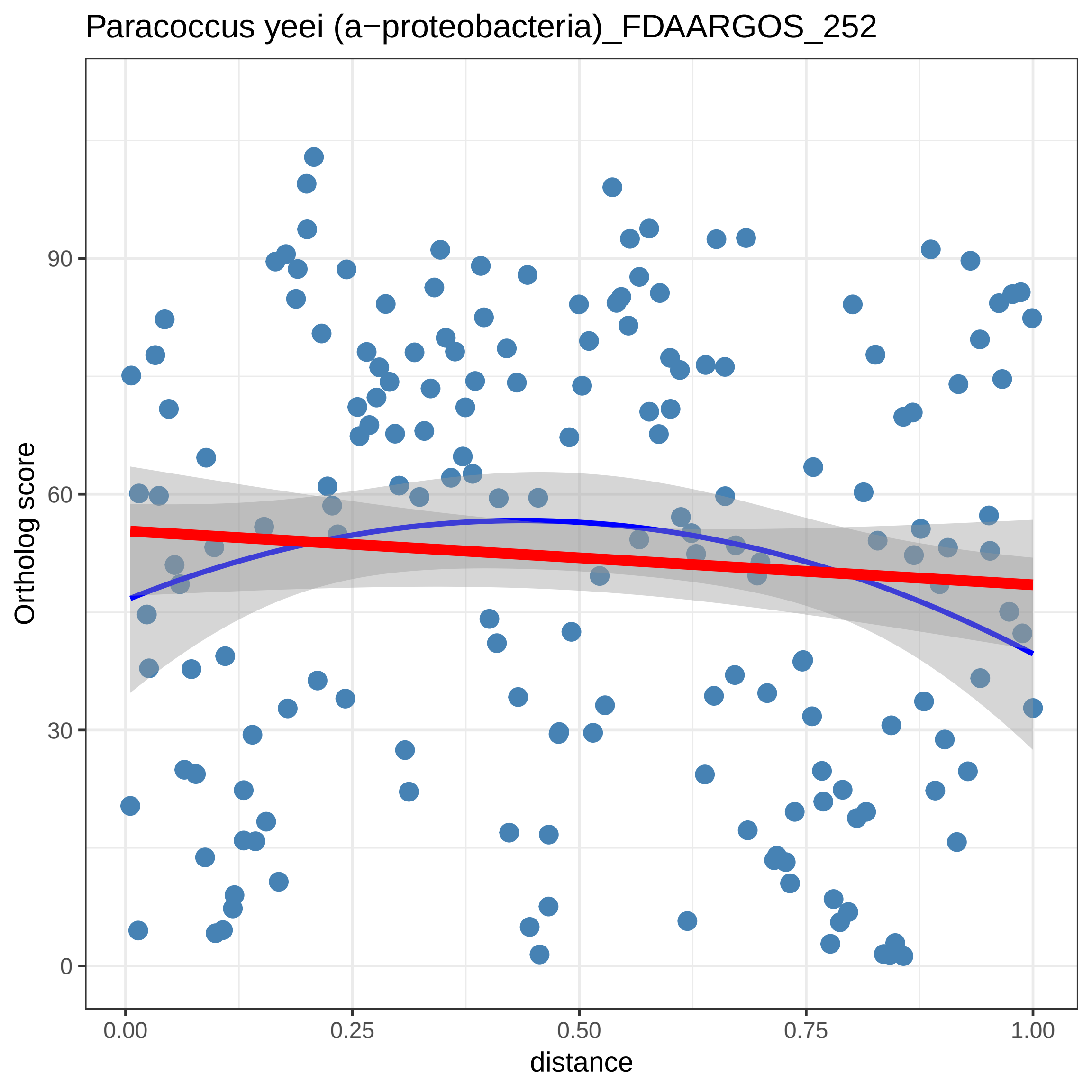

Paracoccus yeei (a−proteobacteria)_FDAARGOS_252
Ortholog score
0
30
60
90
0.00
0.25
0.50
0.75
1.00
distance

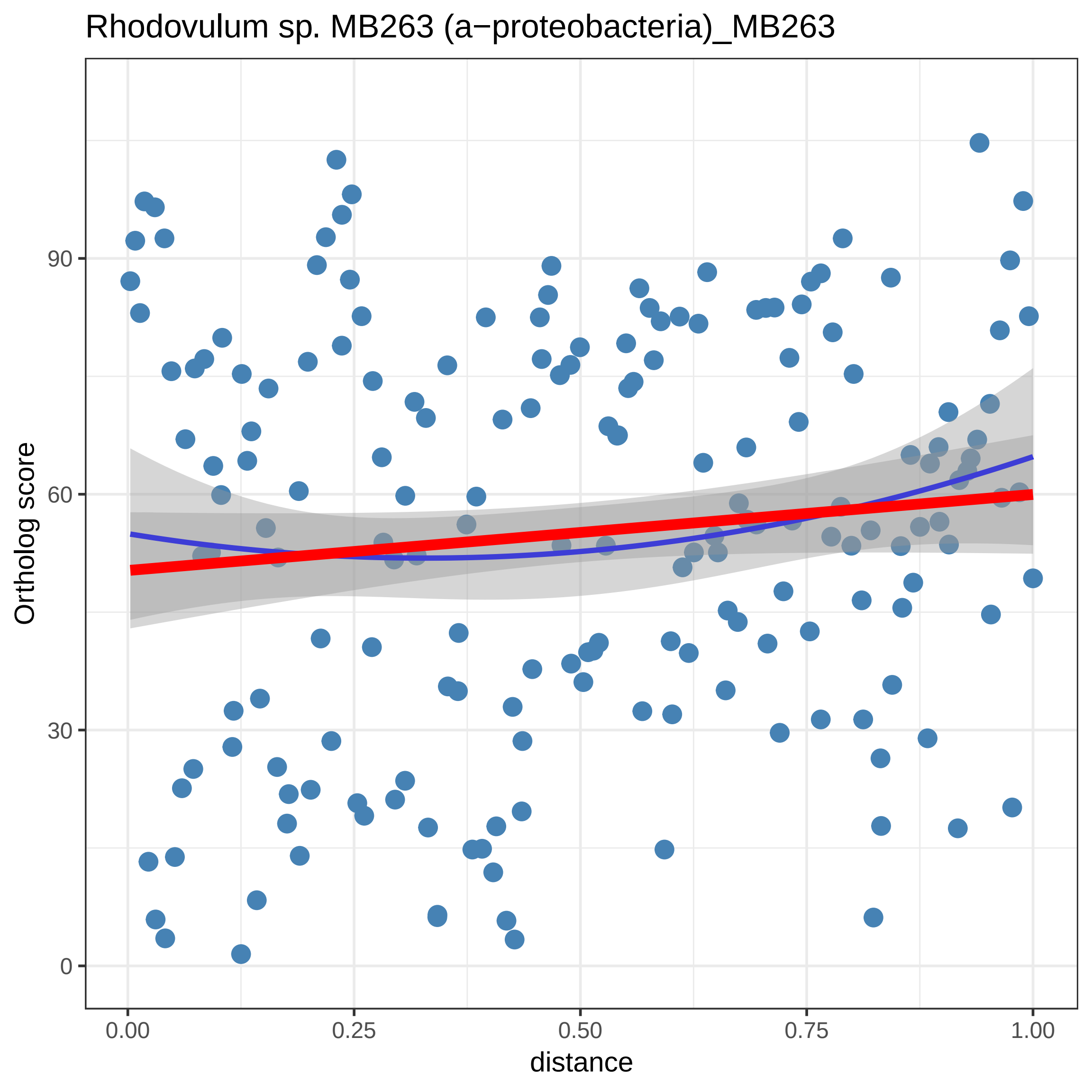
Rhodovulum sp. MB263 (a−proteobacteria)_MB263
Ortholog score
0
30
60
90
distance
0.00
0.25
0.50
0.75
1.00

Rhodovulum sp. P5 (a−proteobacteria)_P5

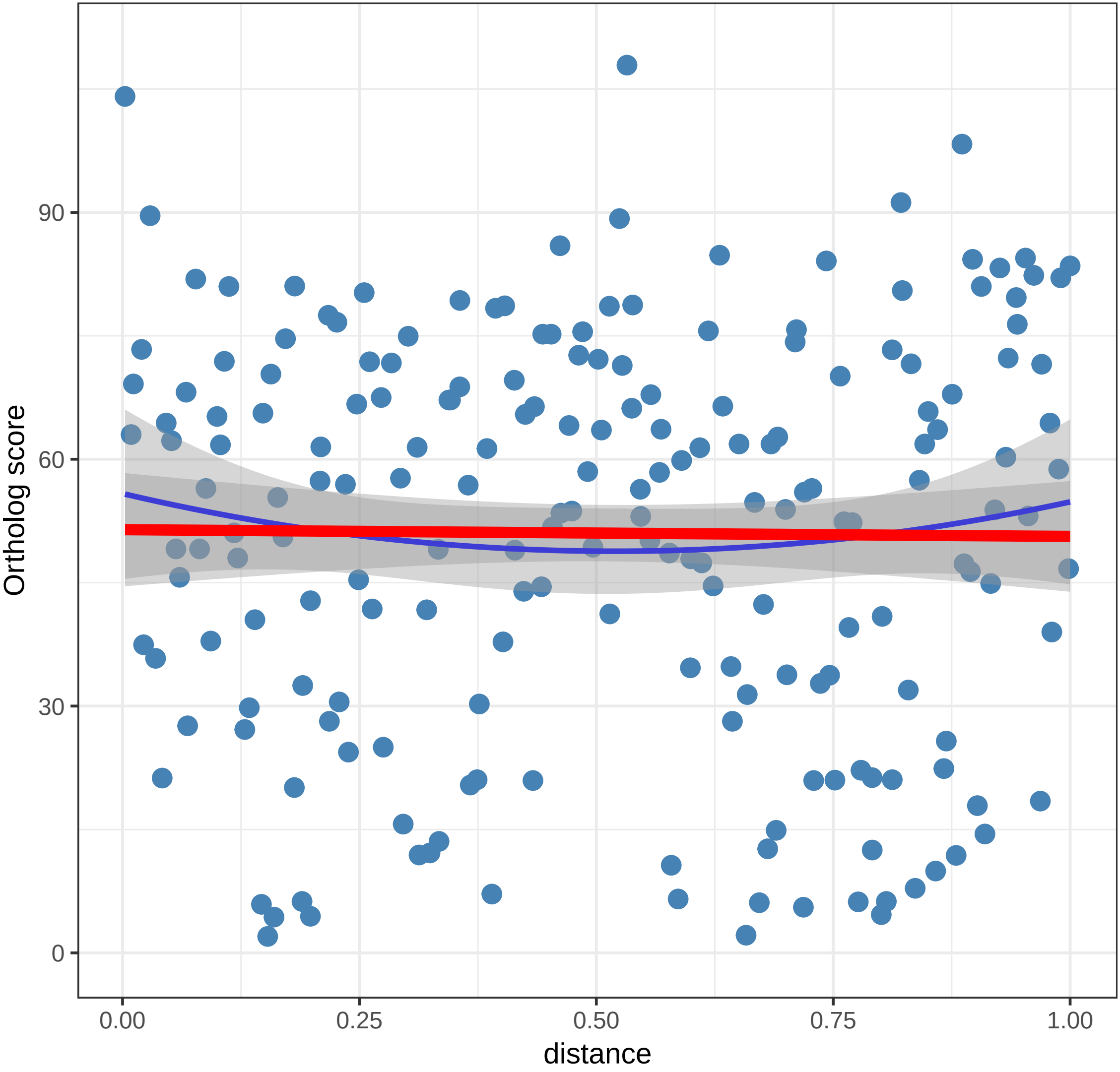

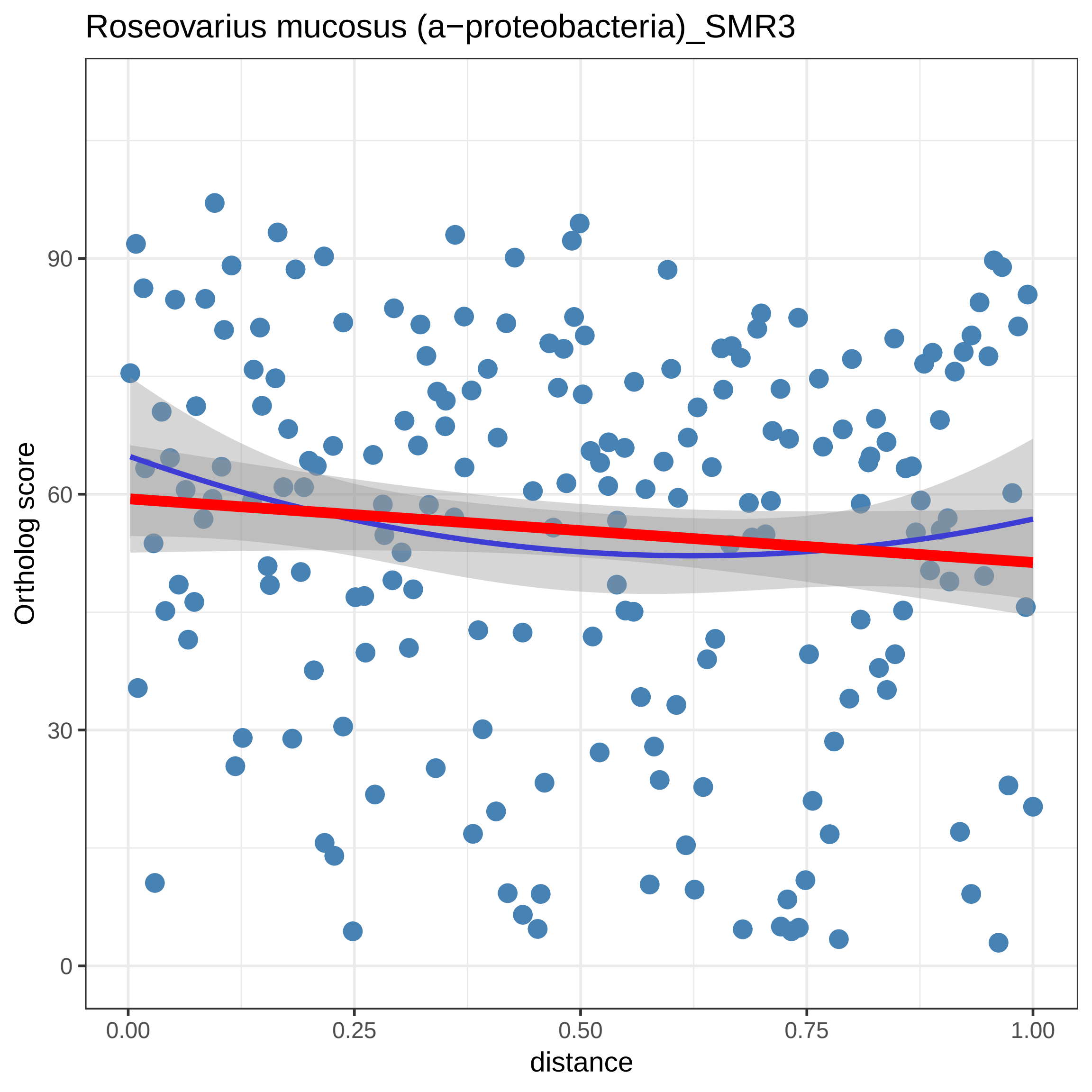
Roseovarius mucosus (a−proteobacteria)_SMR3
Ortholog score
0
30
60
90
0.00
0.25
0.50
0.75
1.00
distance

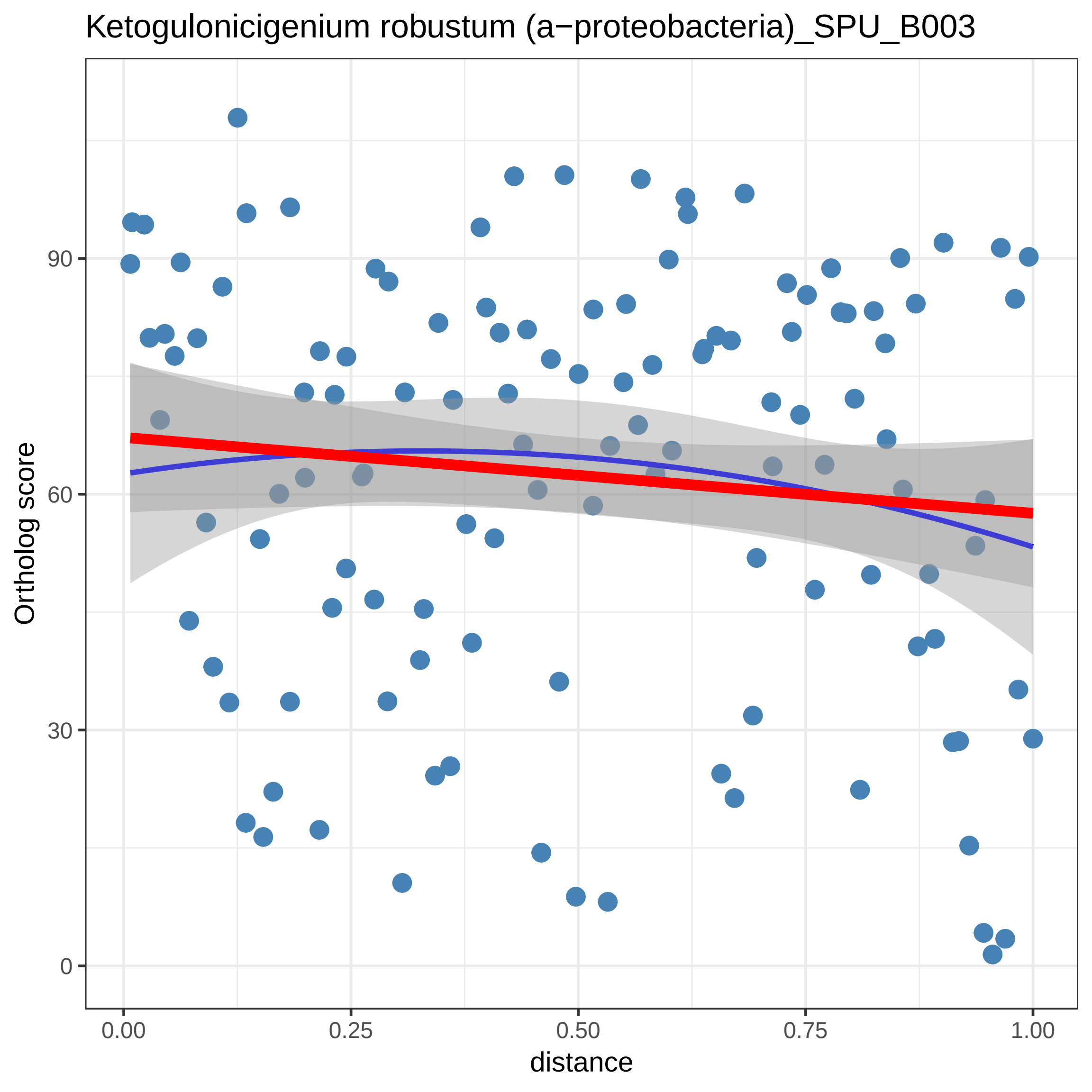

Ketogulonicigenium robustum (a−proteobacteria)_SPU_B003
Ortholog score
distance
0
30
60
90
0.00
0.25
0.50
0.75
1.00

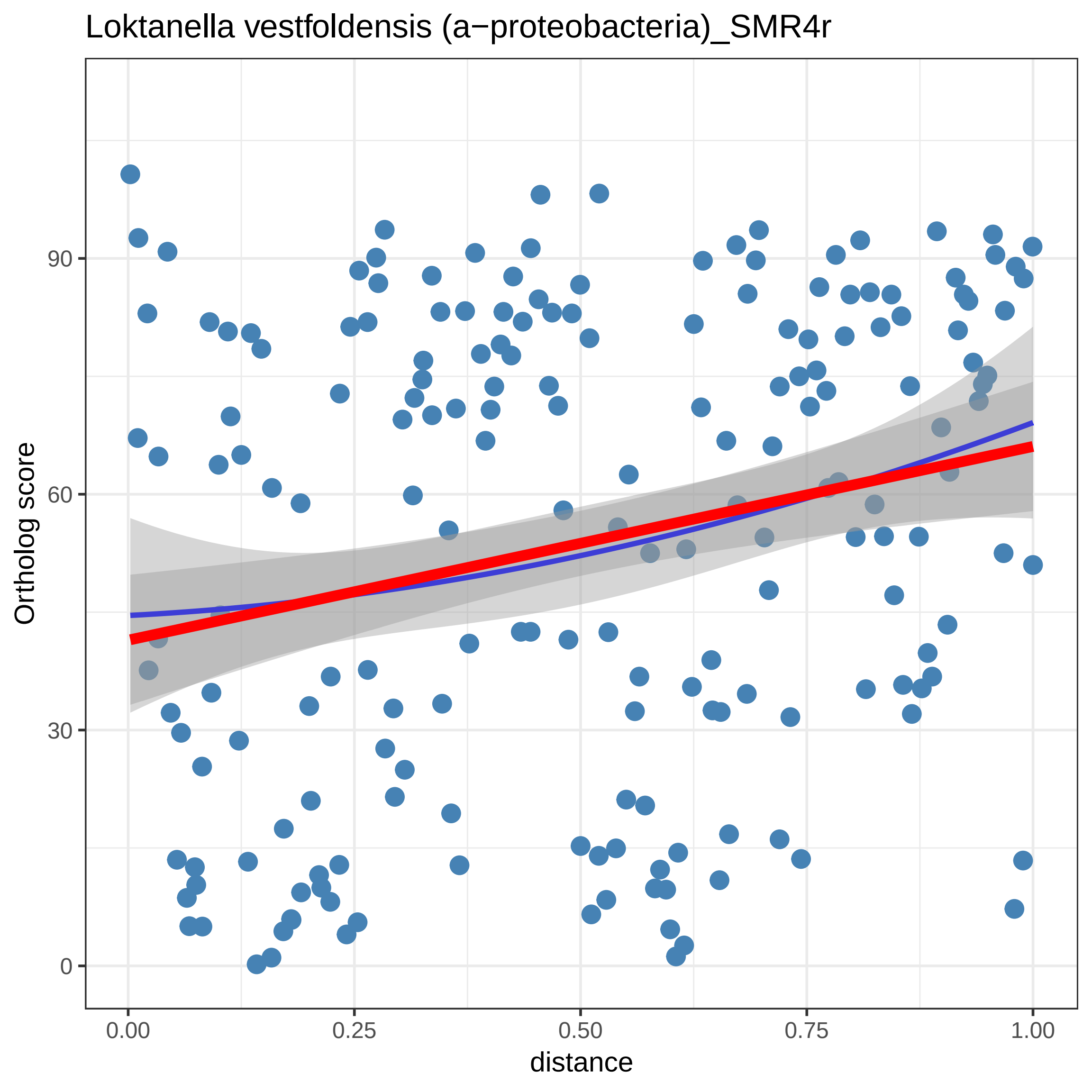

Loktanella vestfoldensis (a−proteobacteria)_SMR4r
Ortholog score
distance
0
30
60
90
0.00
0.25
0.50
0.75
1.00

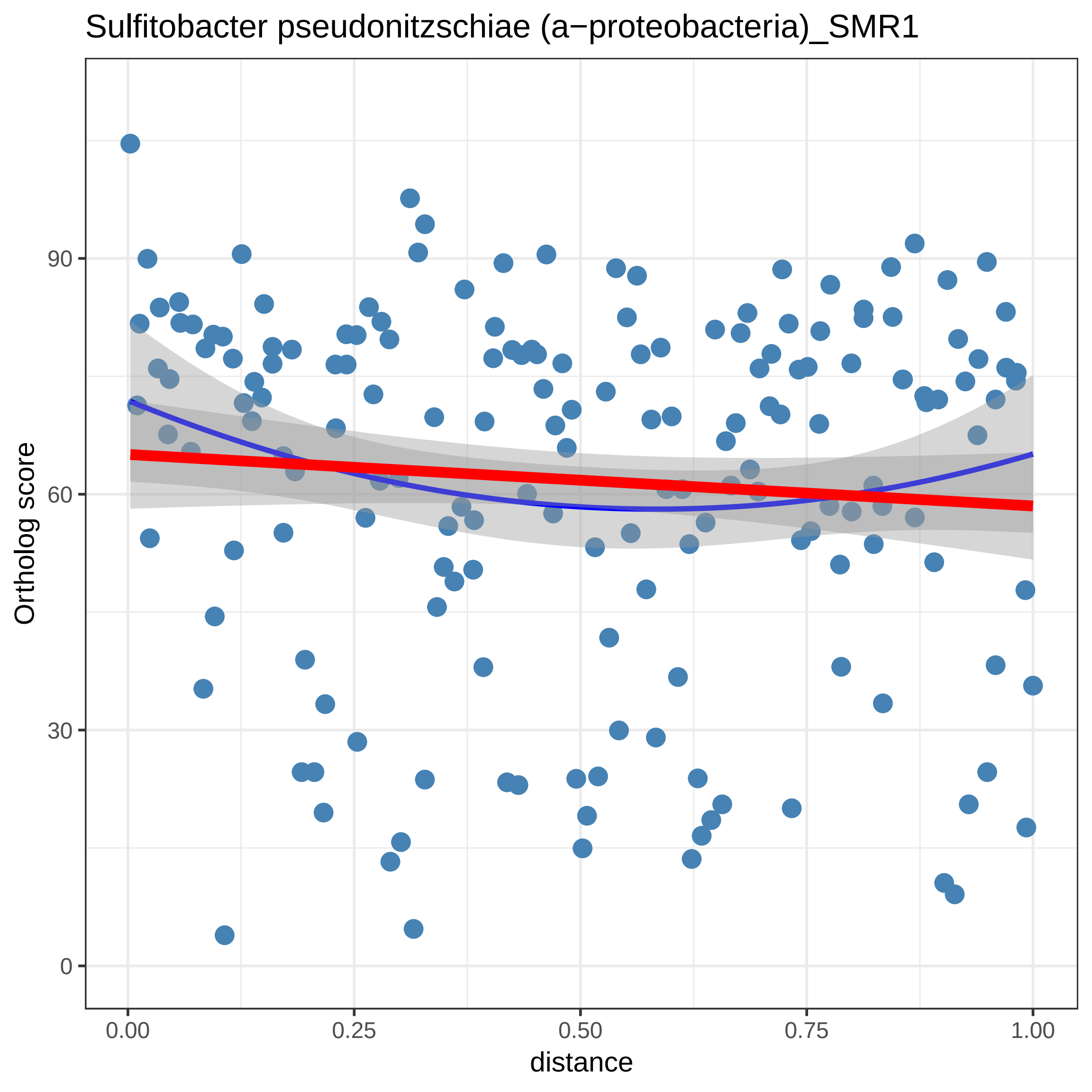
Sulfitobacter pseudonitzschiae (a−proteobacteria)_SMR1
Ortholog score
90
60
30
0
0.00
0.25
0.50
0.75
1.00
distance

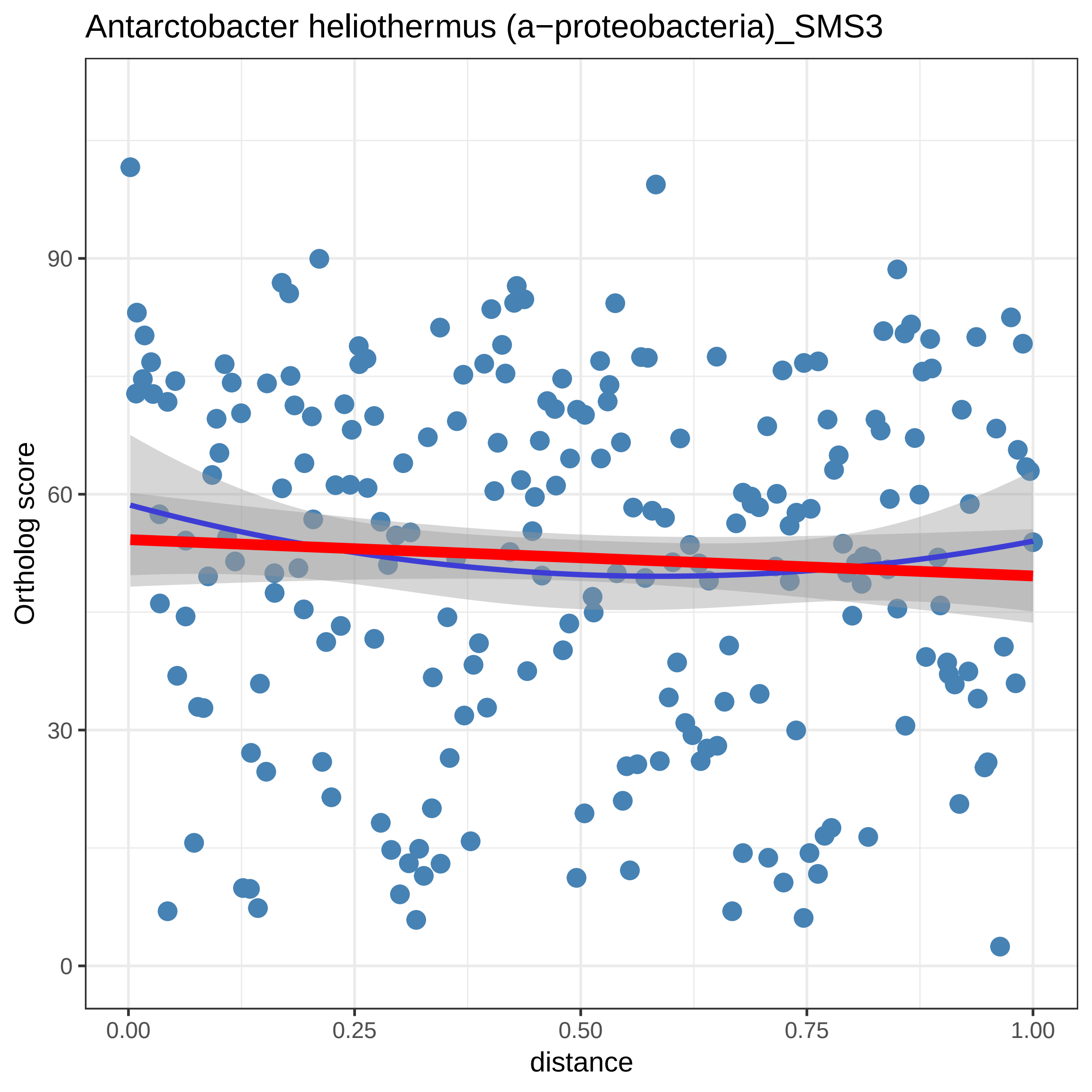
Antarctobacter heliothermus (a−proteobacteria)_SMS3
Ortholog score
90
60
30
0
0.00
0.25
0.50
0.75
1.00
distance

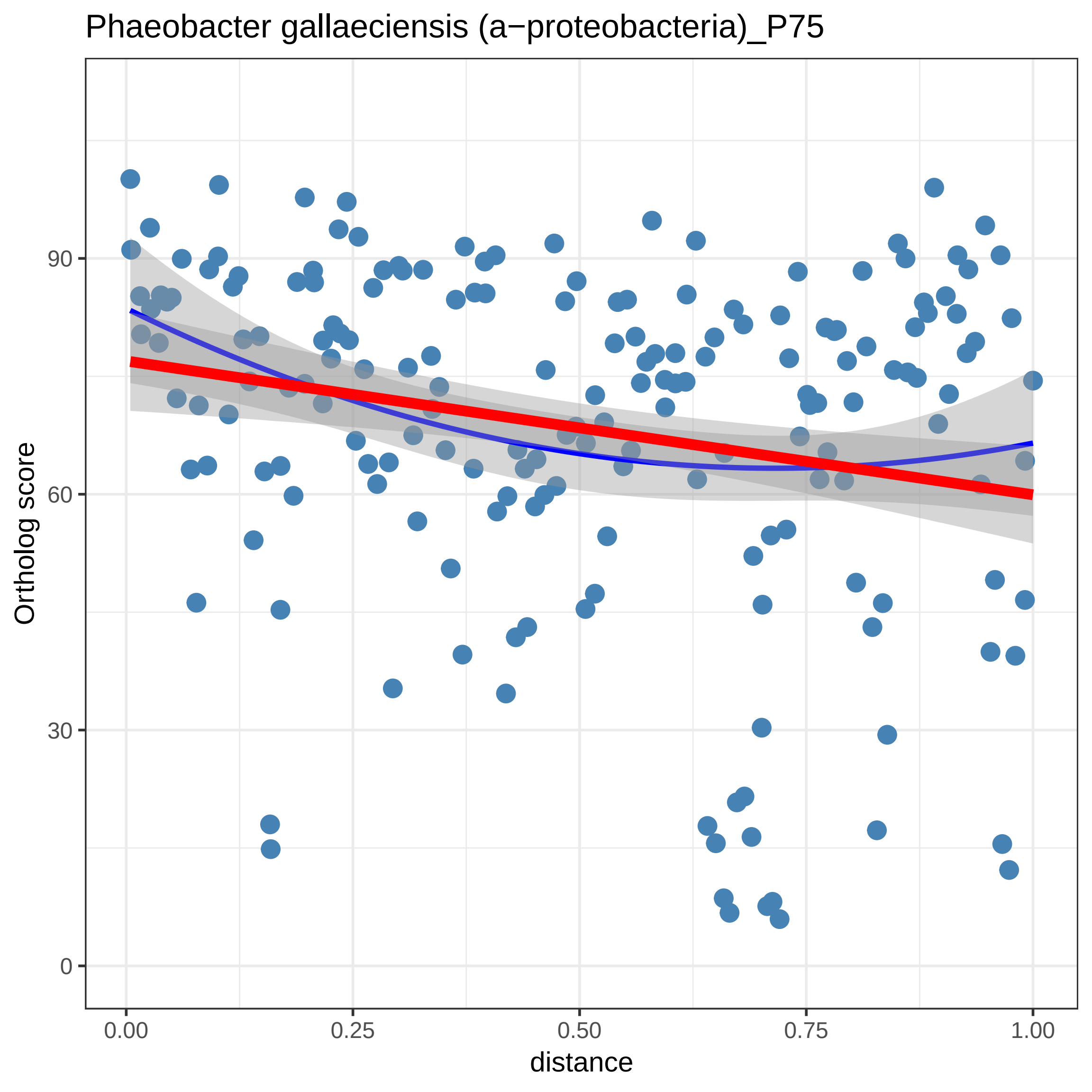
Phaeobacter gallaeciensis (a−proteobacteria)_P75
Ortholog score
distance
0
30
60
90
0.00
0.25
0.50
0.75
1.00

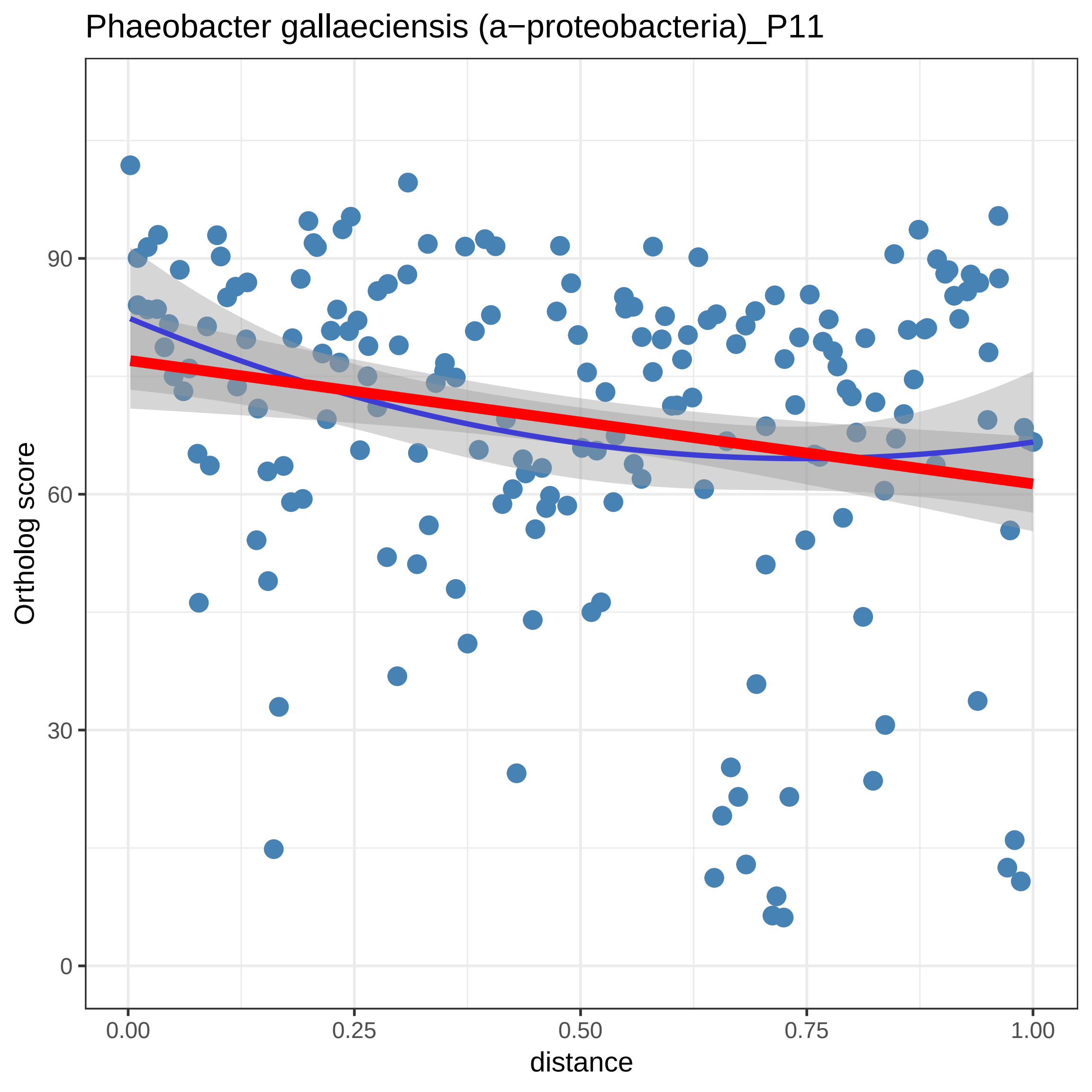
Phaeobacter gallaeciensis (a−proteobacteria)_P11
Ortholog score
90
60
30
0
0.00
0.25
0.50
0.75
1.00
distance

Phaeobacter gallaeciensis (a−proteobacteria)_P73

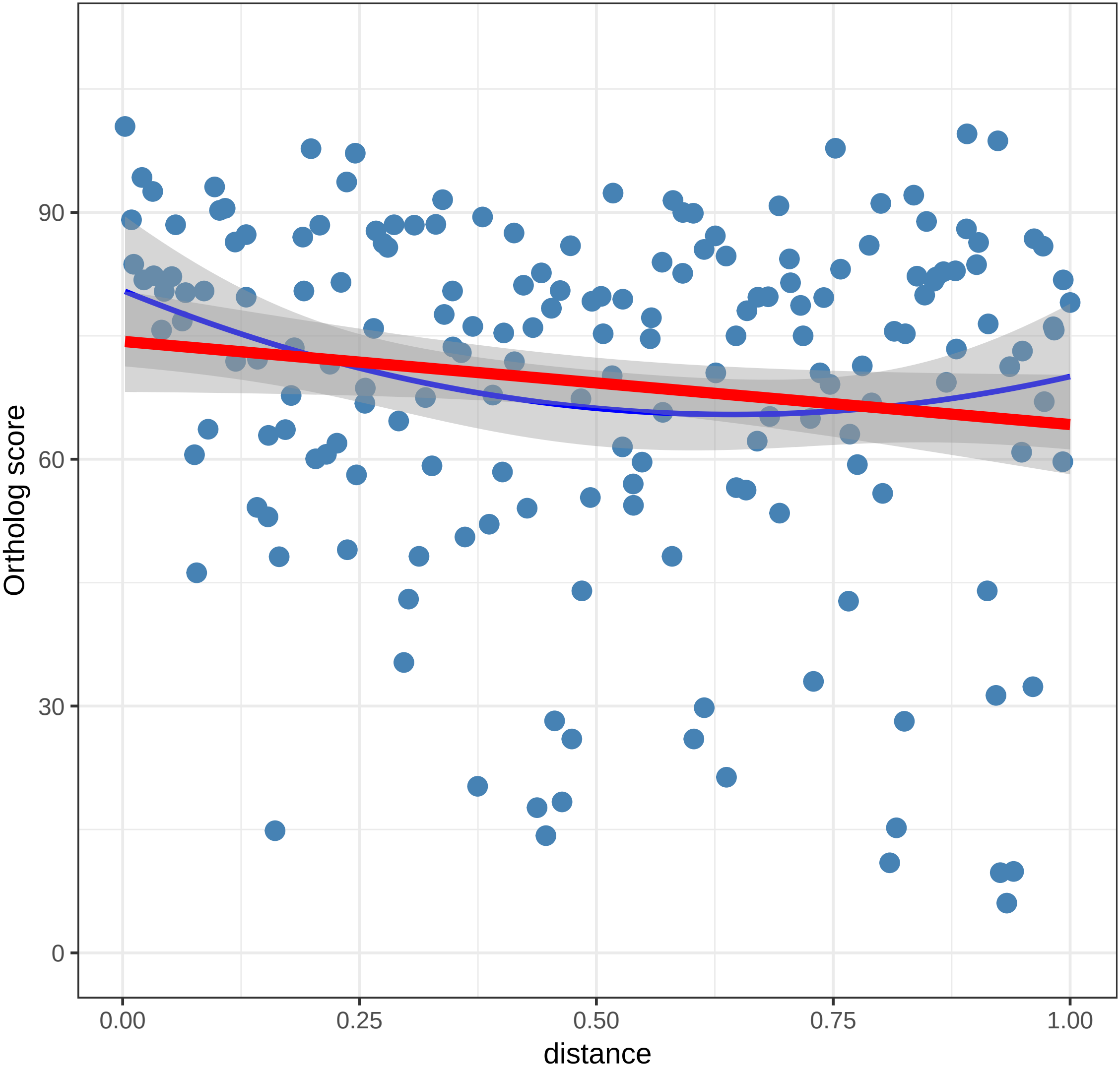

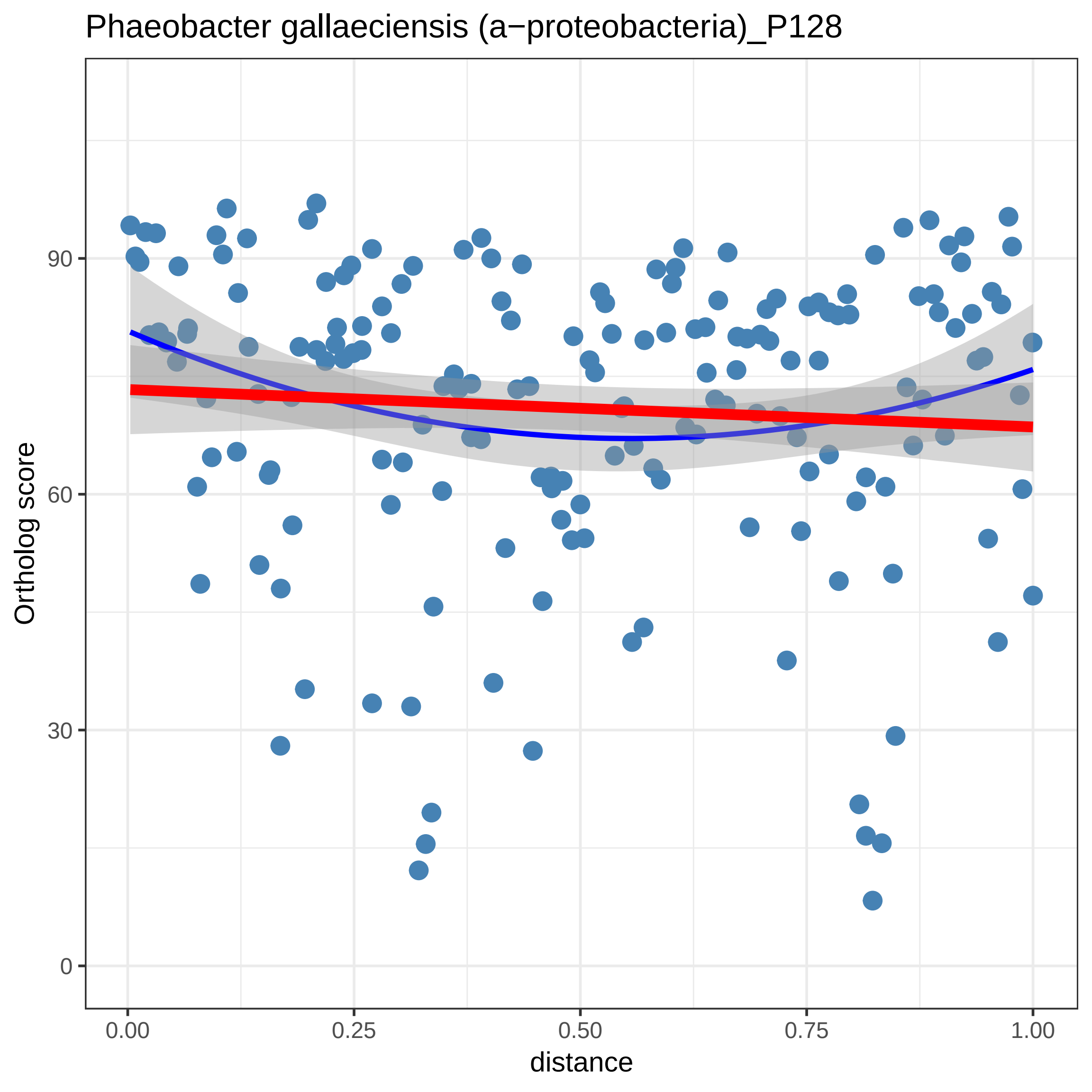

Phaeobacter gallaeciensis (a−proteobacteria)_P128
90
60
30
0
Ortholog score
0.00
0.25
0.50
0.75
1.00
distance

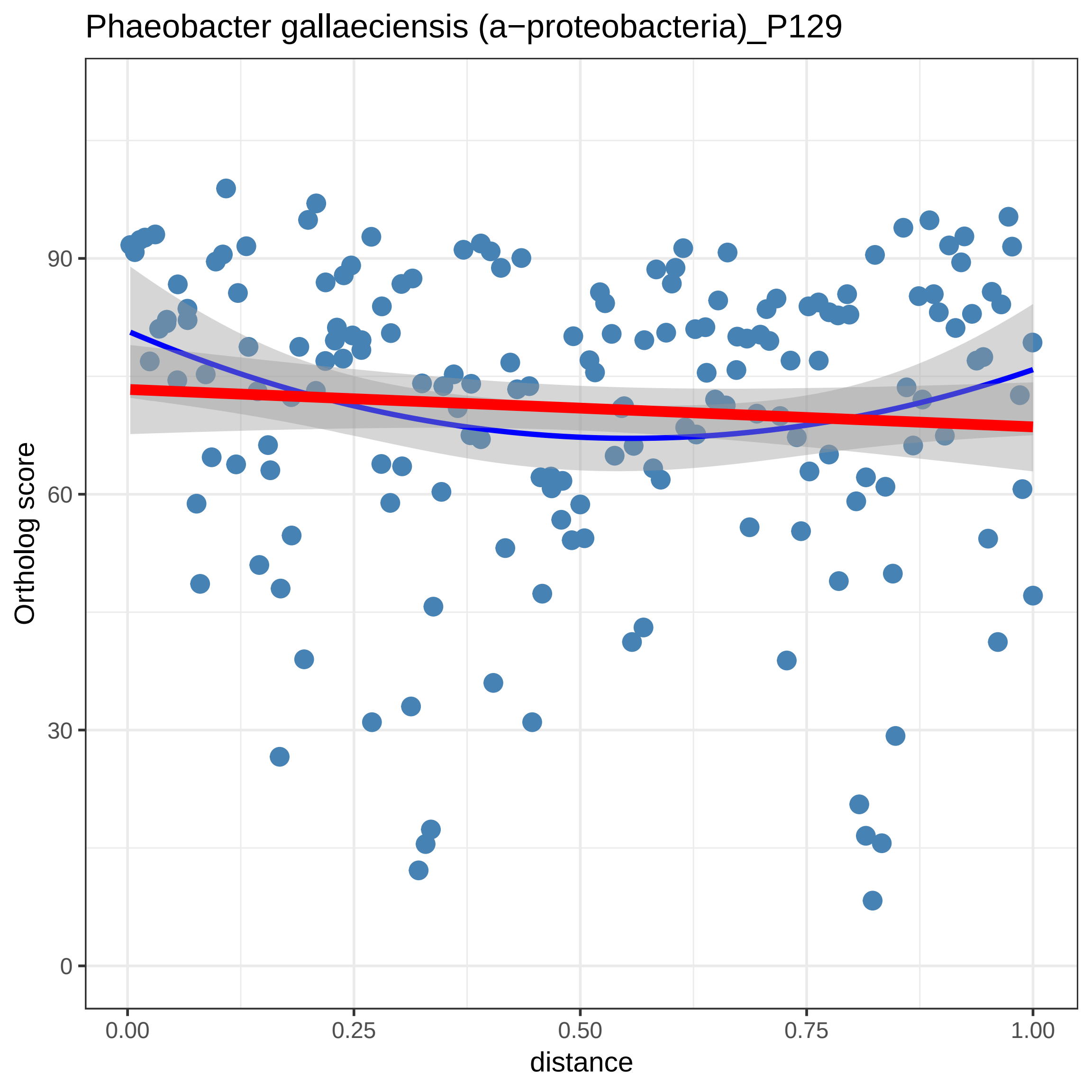

Phaeobacter gallaeciensis (a−proteobacteria)_P129
Ortholog score
90
60
30
0
0.00
0.25
0.50
0.75
1.00
distance

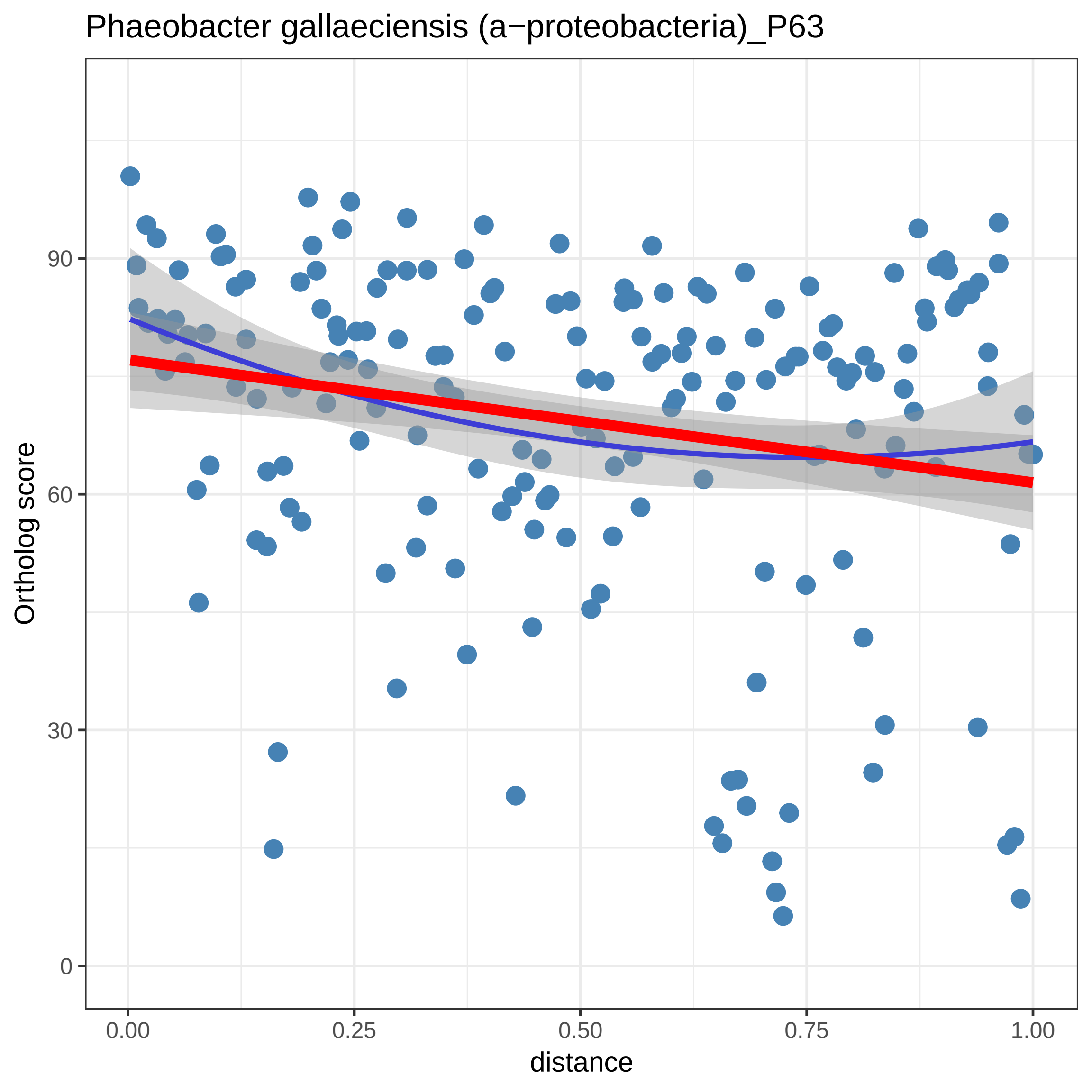

Phaeobacter gallaeciensis (a−proteobacteria)_P63
Ortholog score
90
60
30
0
0.00
0.25
0.50
0.75
1.00
distance

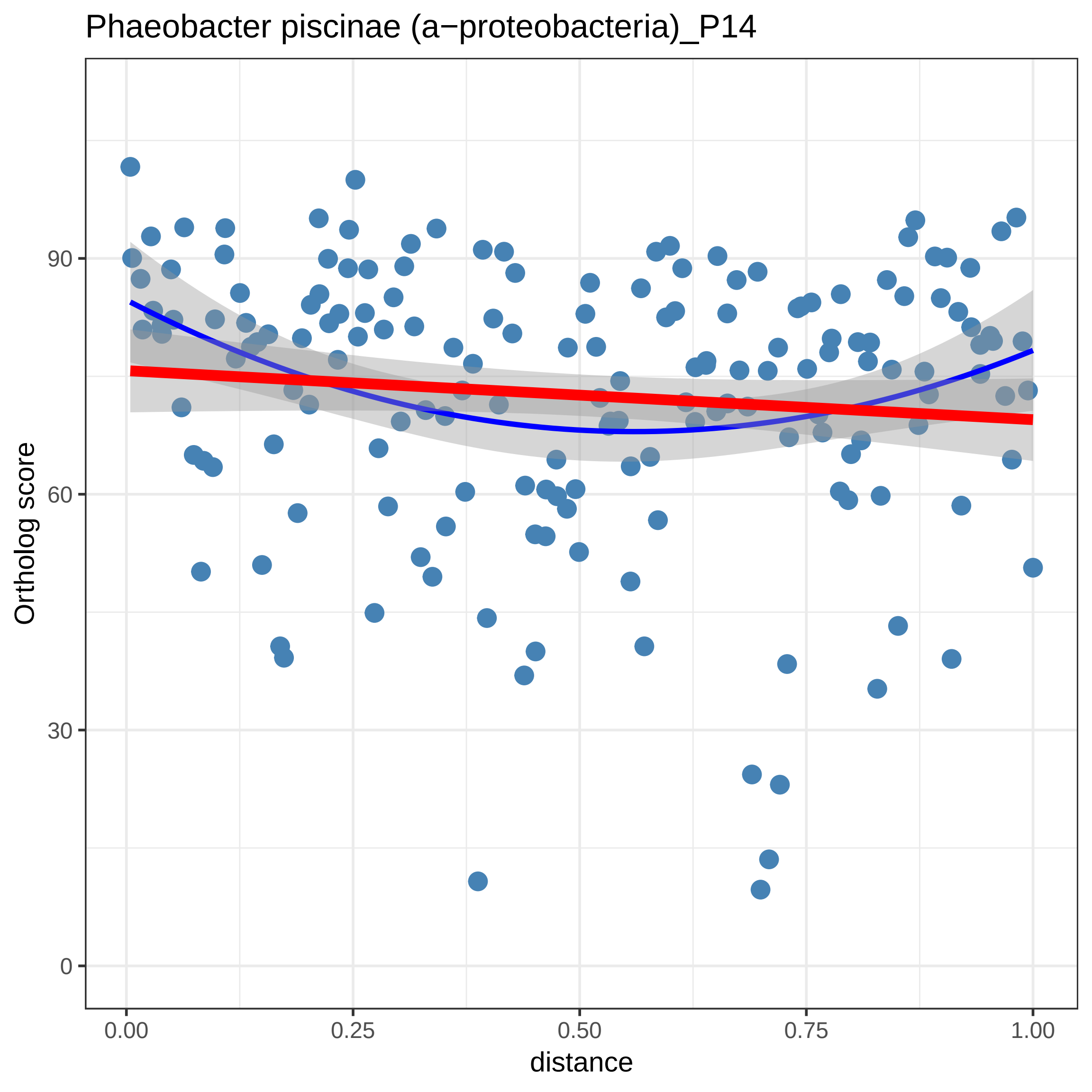

Phaeobacter piscinae (a−proteobacteria)_P14
Ortholog score
90
60
30
0
0.00
0.25
0.50
0.75
1.00
distance

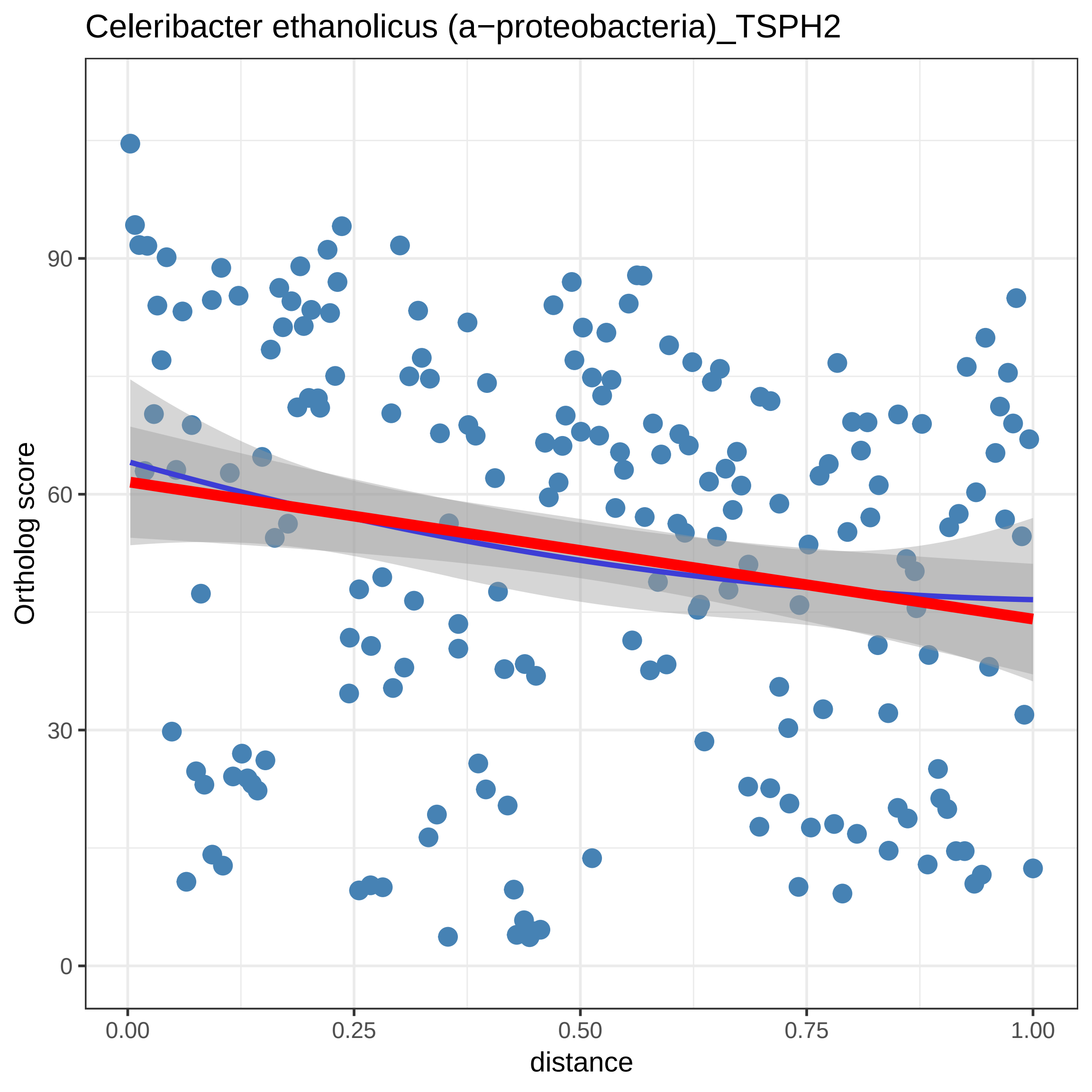
Celeribacter ethanolicus (a−proteobacteria)_TSPH2
Ortholog score
0
30
60
90
0.00
0.25
0.50
0.75
1.00
distance

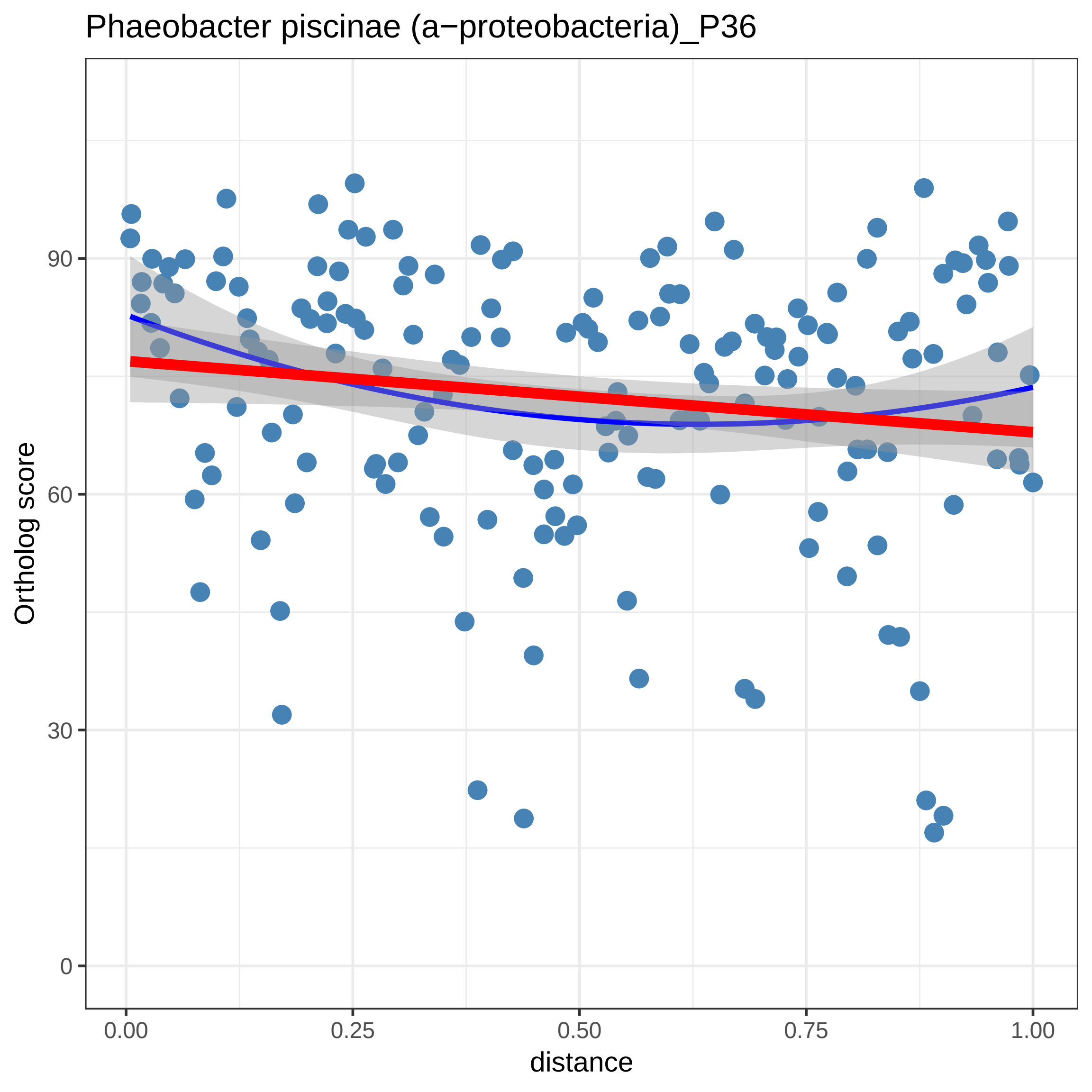

Phaeobacter piscinae (a−proteobacteria)_P36
Ortholog score
90
60
30
0
0.00
0.25
0.50
0.75
1.00
distance

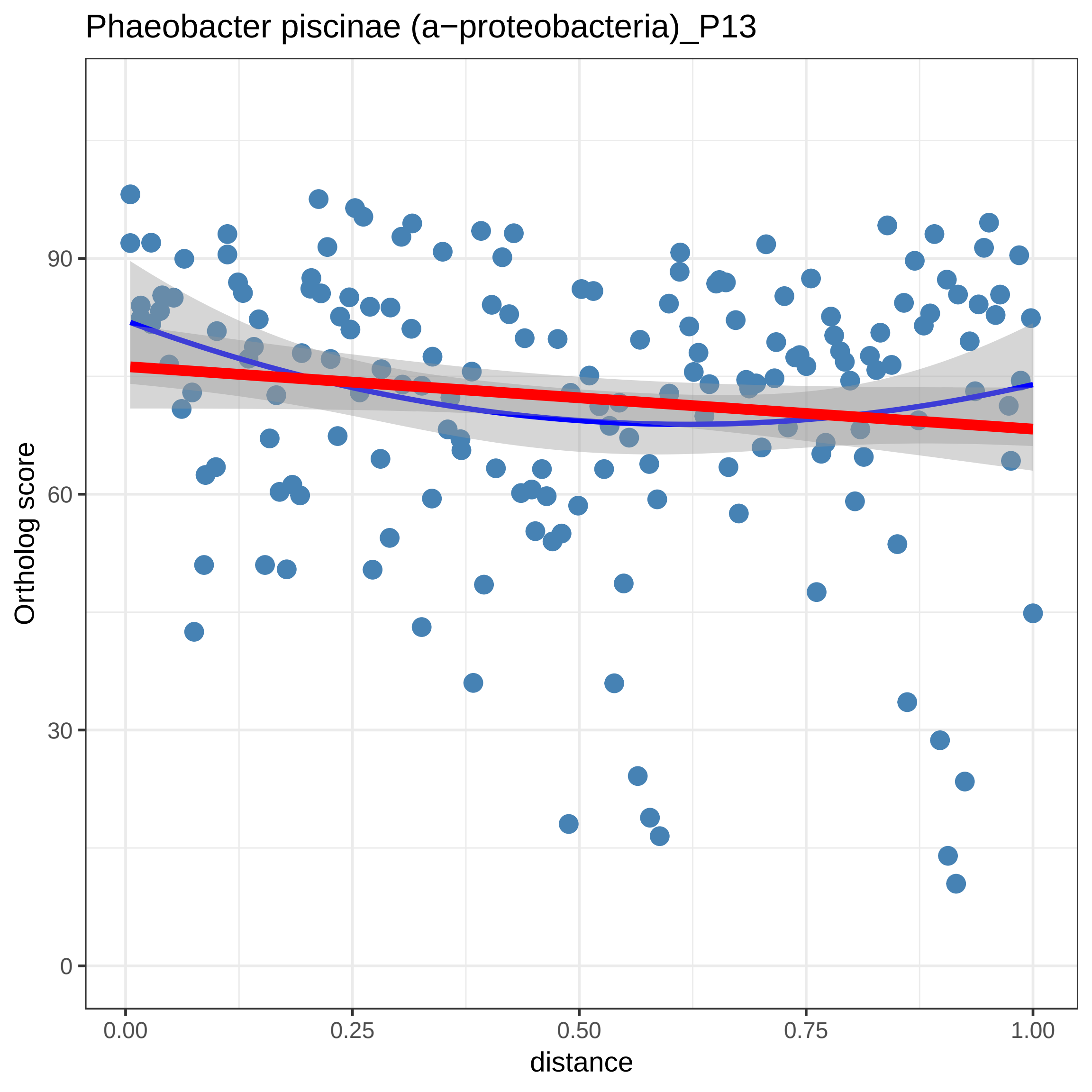
Phaeobacter piscinae (a−proteobacteria)_P13
Ortholog score
90
60
30
0
0.00
0.25
0.50
0.75
1.00
distance

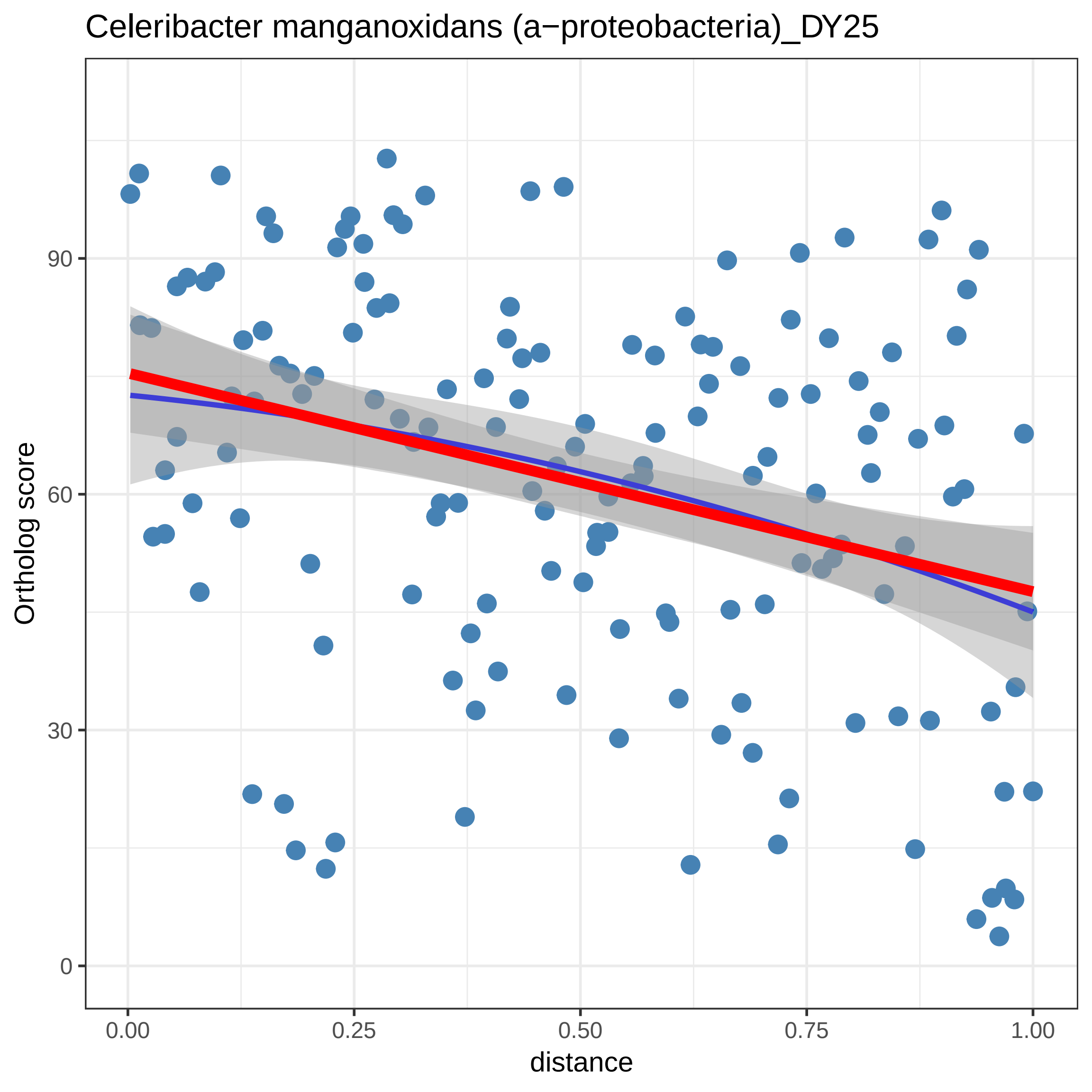
Celeribacter manganoxidans (a−proteobacteria)_DY25
Ortholog score
distance
0
30
60
90
0.00
0.25
0.50
0.75
1.00

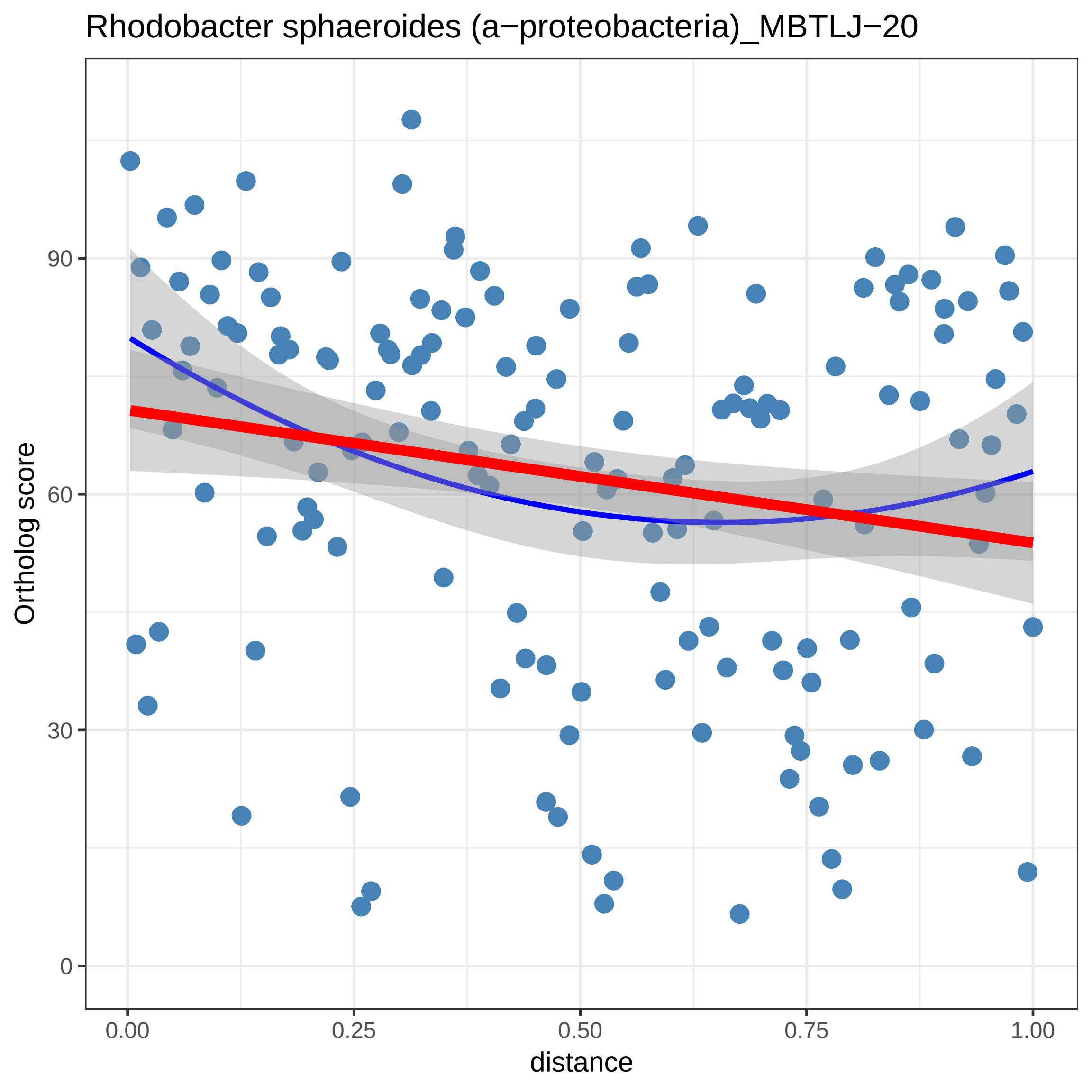
Rhodobacter sphaeroides (a−proteobacteria)_MBTLJ−20
Ortholog score
distance
0
30
60
90
0.00
0.25
0.50
0.75
1.00

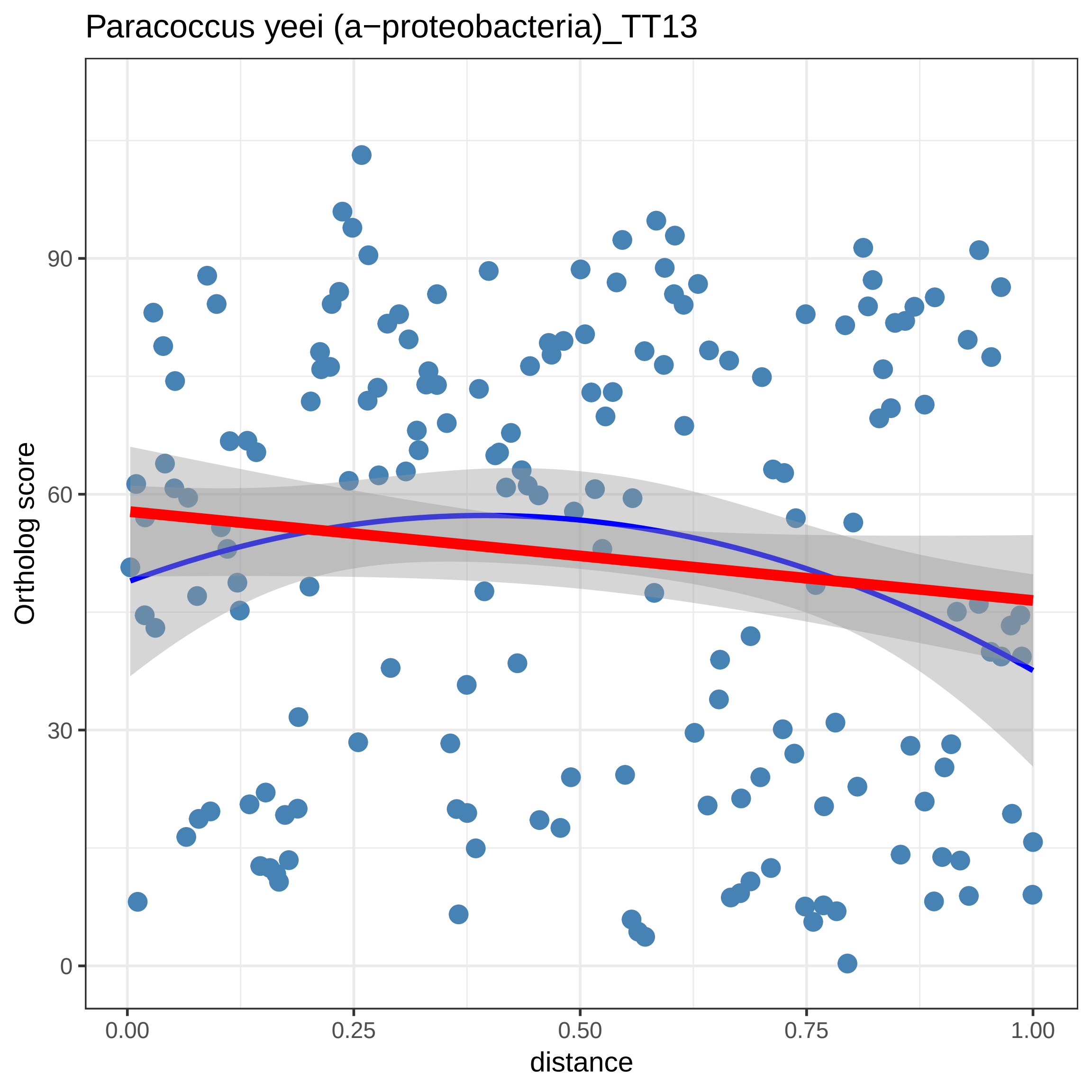

Paracoccus yeei (a−proteobacteria)_TT13
Ortholog score
distance
0
30
60
90
0.00
0.25
0.50
0.75
1.00

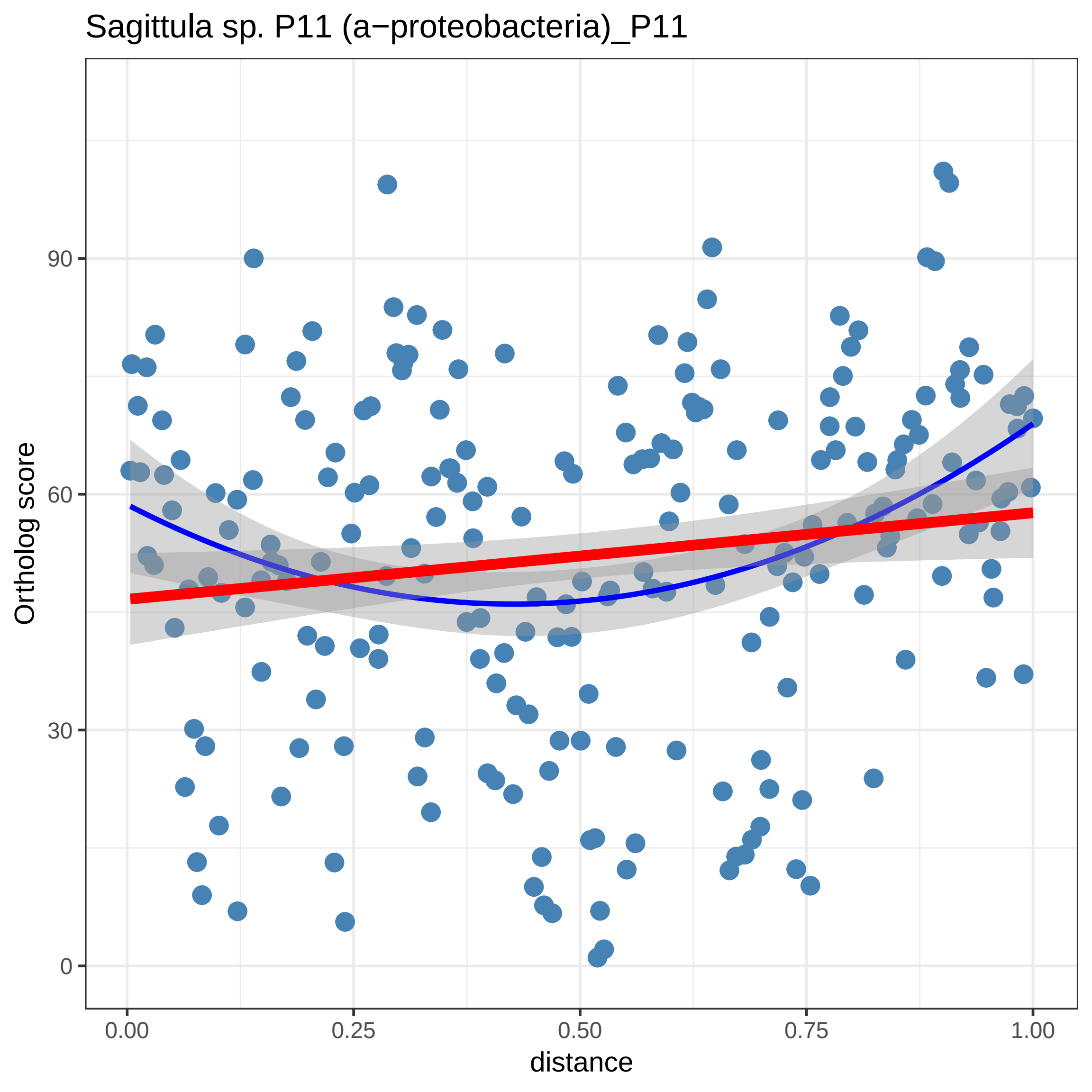
Sagittula sp. P11 (a−proteobacteria)_P11
Ortholog score
90
60
30
0
0.00
0.25
0.50
0.75
1.00
distance

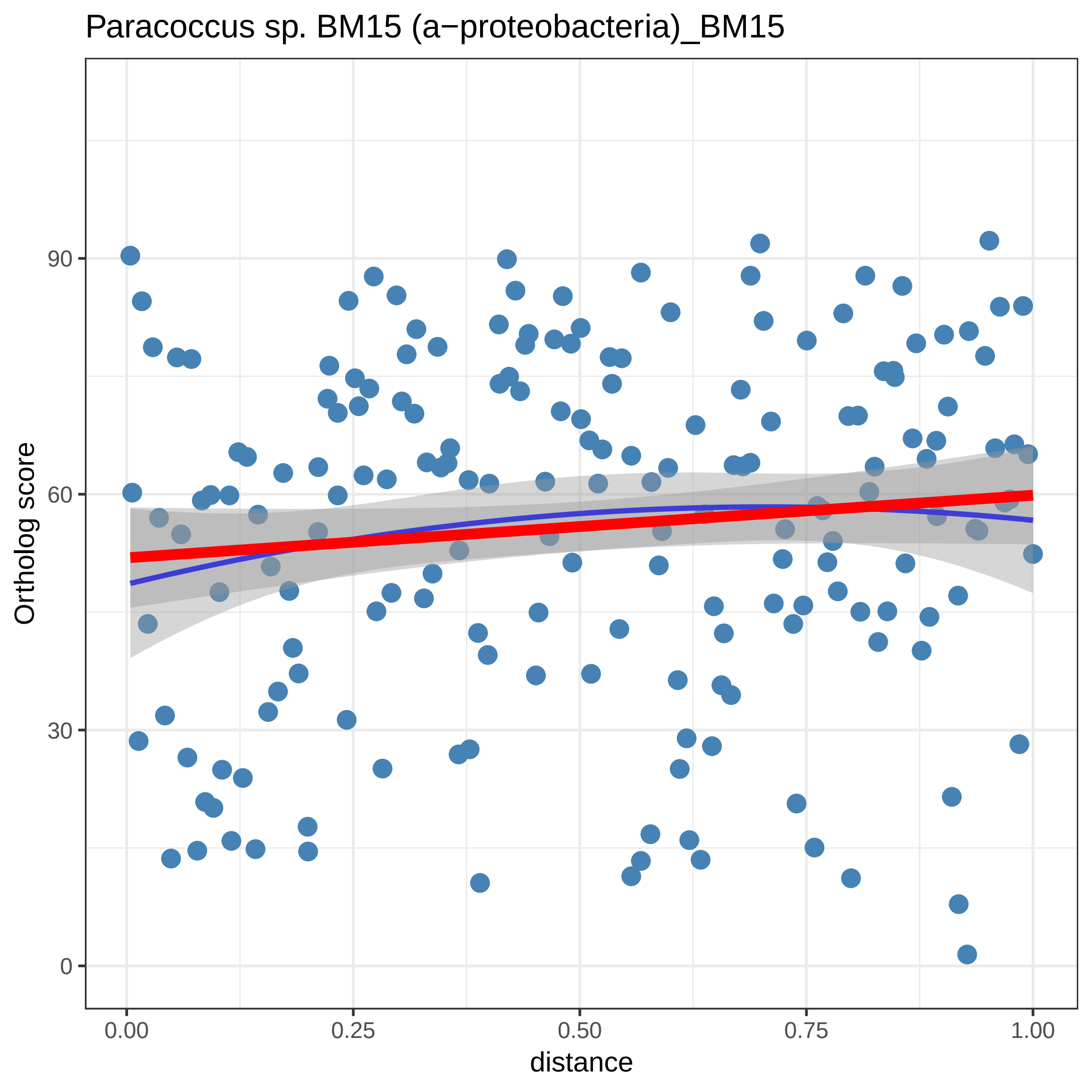

Paracoccus sp. BM15 (a−proteobacteria)_BM15
Ortholog score
90
60
30
0
0.00
0.25
0.50
0.75
1.00
distance

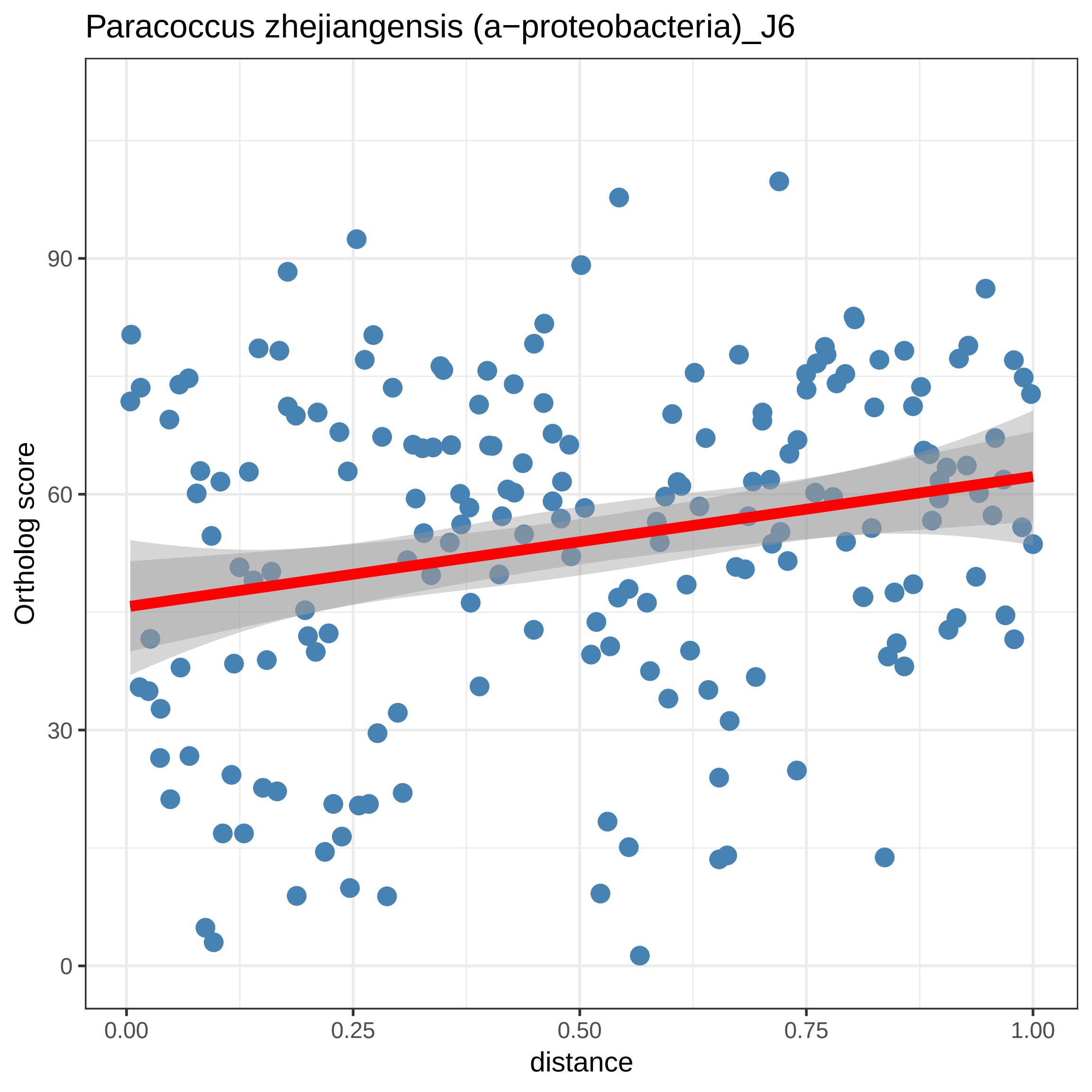

Paracoccus zhejiangensis (a−proteobacteria)_J6
Ortholog score
0
30
60
90
0.00
0.25
0.50
0.75
1.00
distance

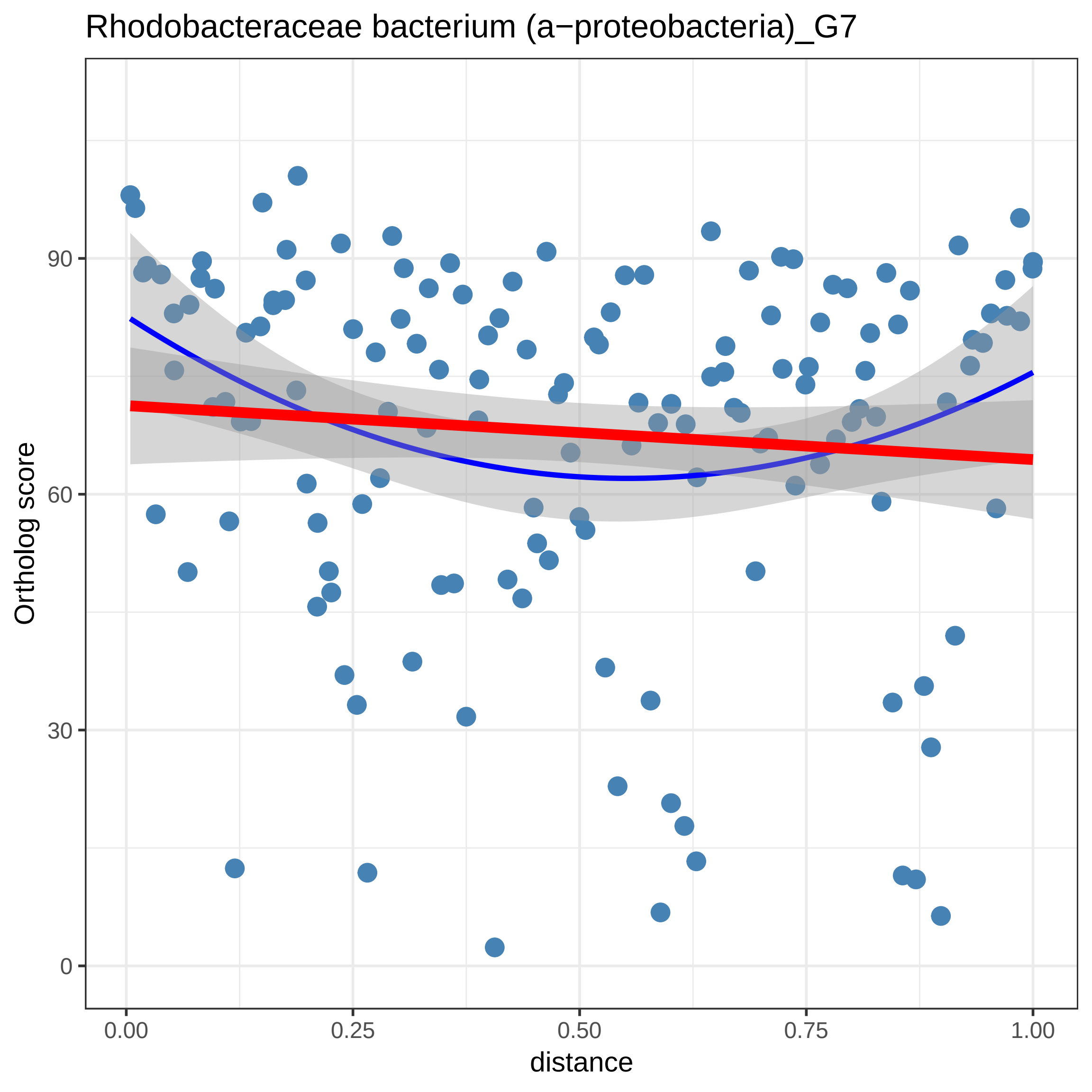

Rhodobacteraceae bacterium (a−proteobacteria)_G7
Ortholog score
distance
0
30
60
90
0.00
0.25
0.50
0.75
1.00

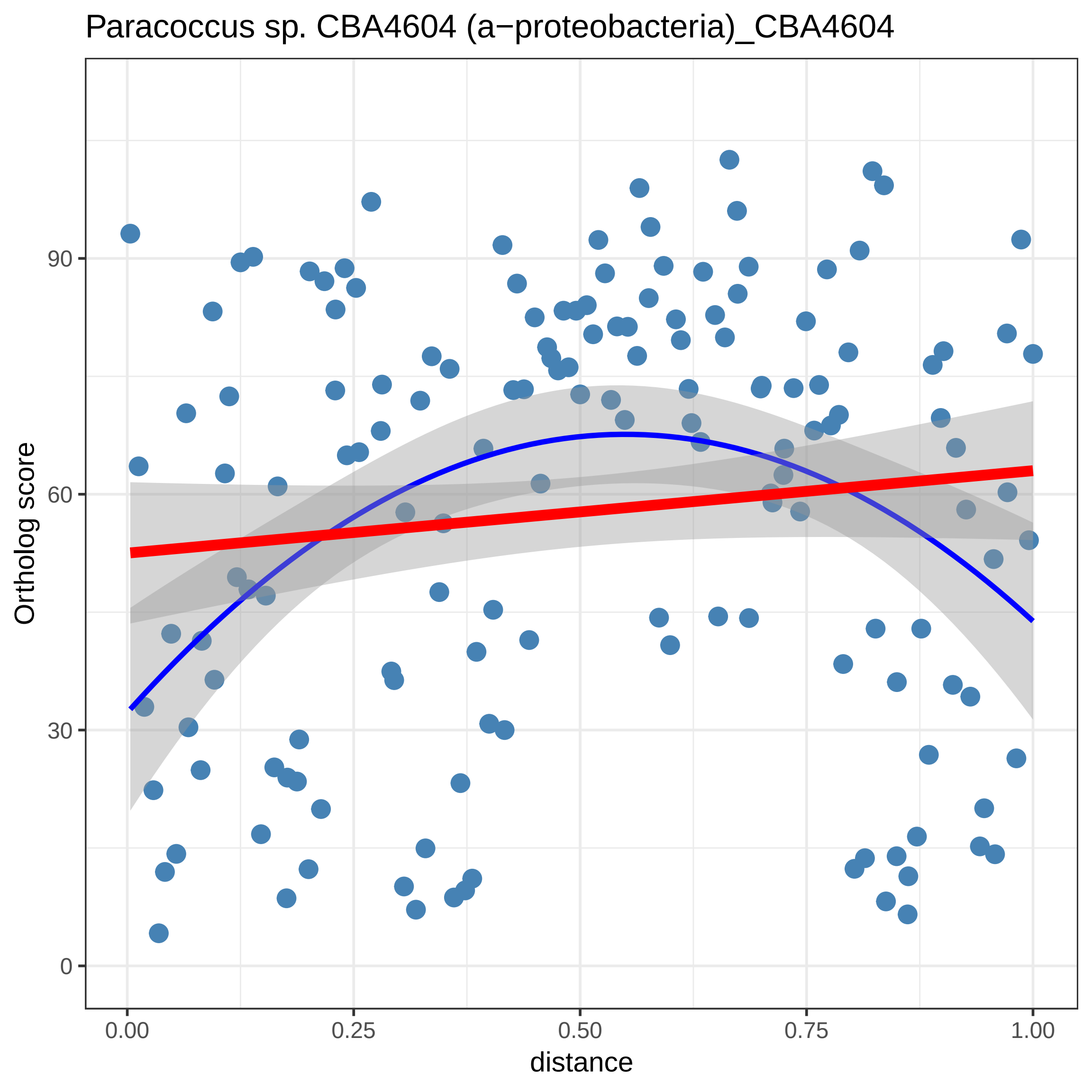

Paracoccus sp. CBA4604 (a−proteobacteria)_CBA4604
Ortholog score
0
30
60
90
0.00
0.25
0.50
0.75
1.00
distance

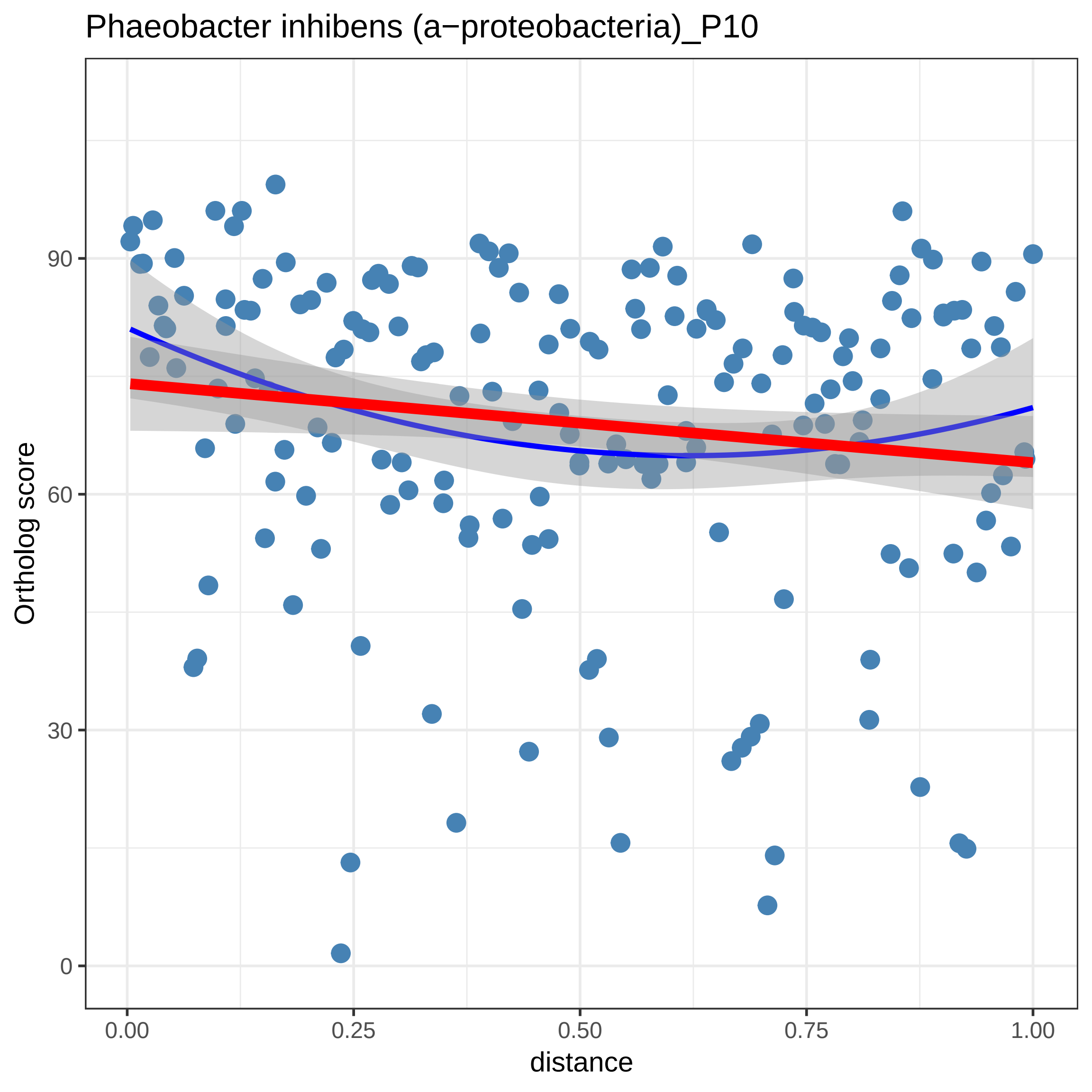

Phaeobacter inhibens (a−proteobacteria)_P10
Ortholog score
0
30
60
90
distance
0.00
0.25
0.50
0.75
1.00

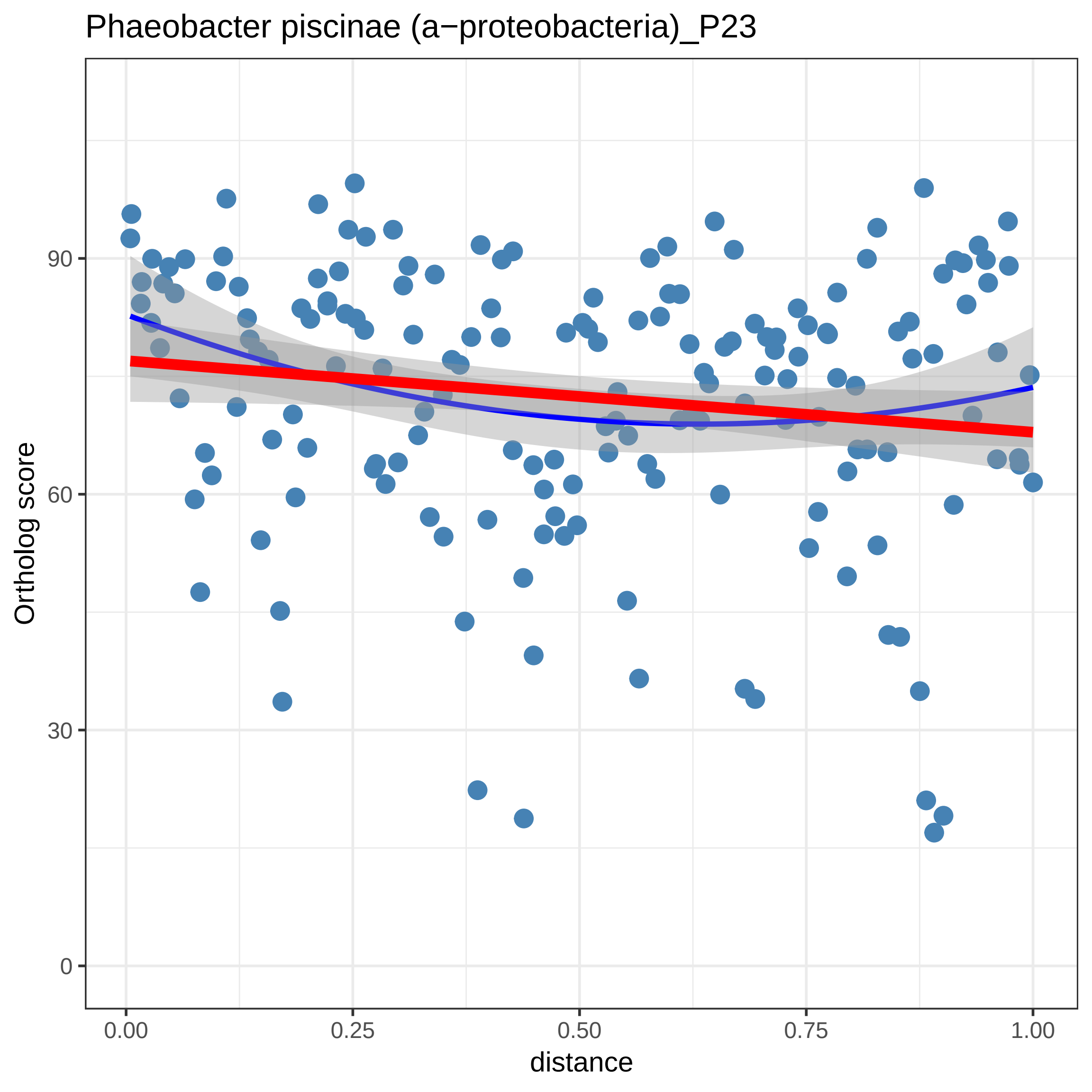

Phaeobacter piscinae (a−proteobacteria)_P23
Ortholog score
90
60
30
0
0.00
0.25
0.50
0.75
1.00
distance

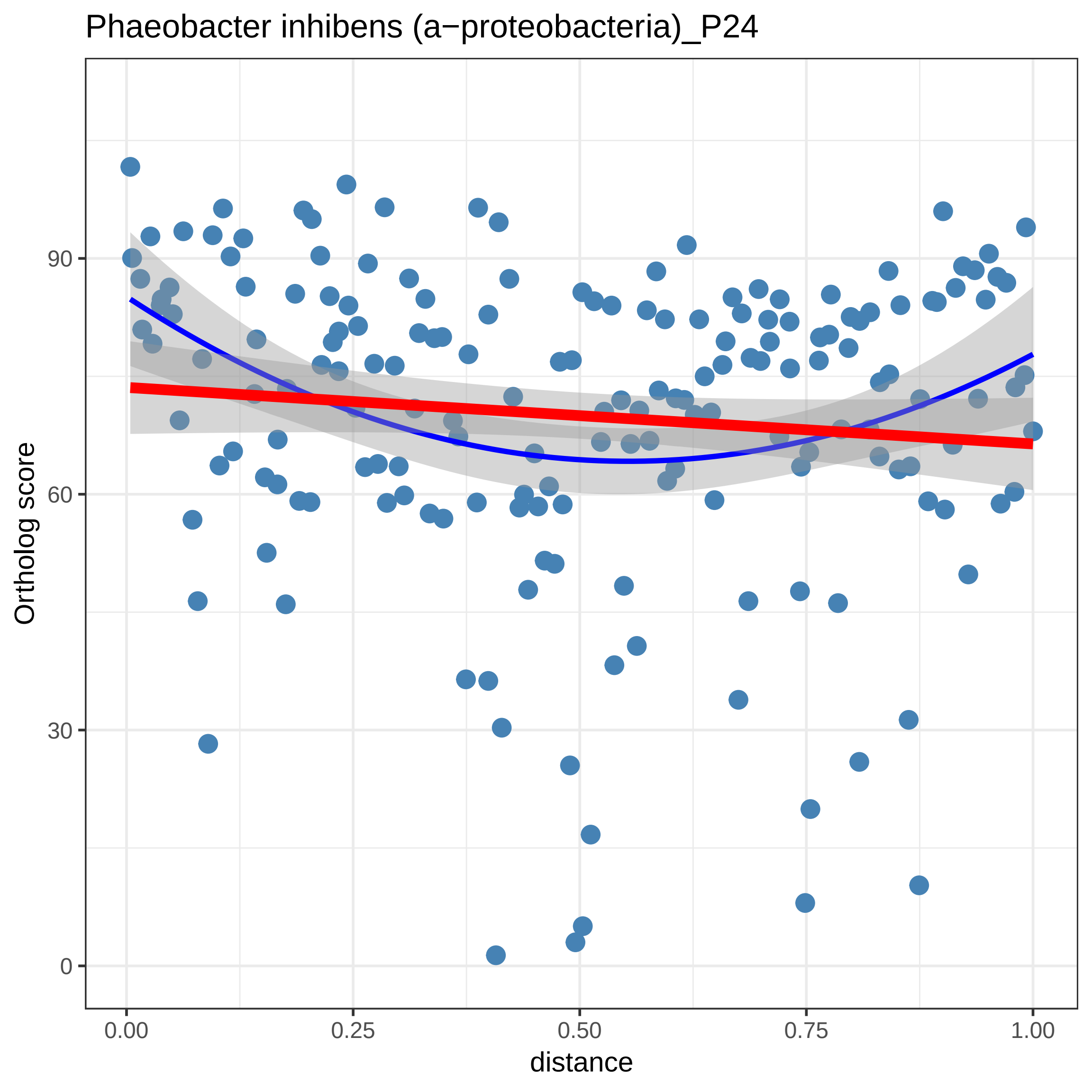
Phaeobacter inhibens (a−proteobacteria)_P24
Ortholog score
distance
0
30
60
90
0.00
0.25
0.50
0.75
1.00

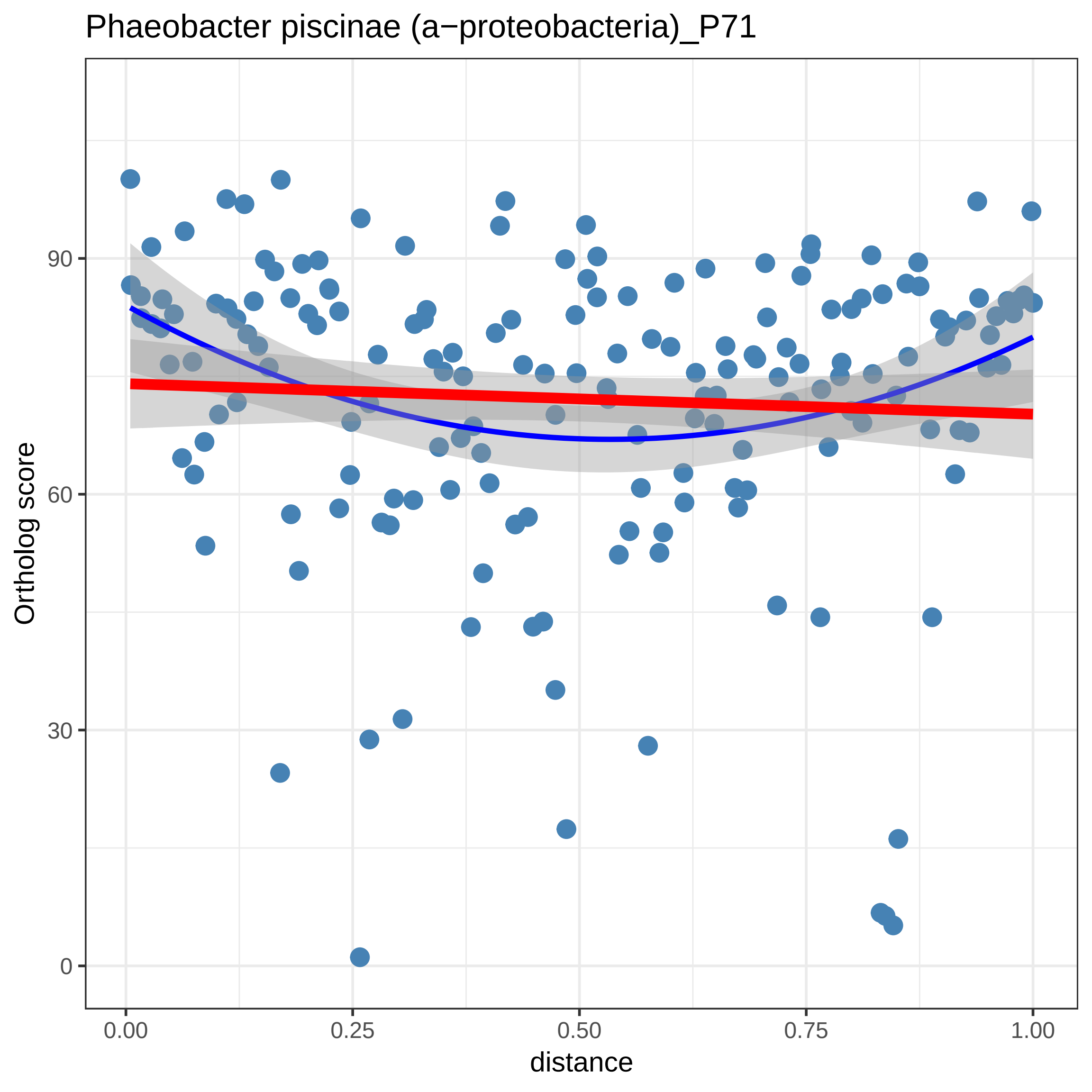
Phaeobacter piscinae (a−proteobacteria)_P71
Ortholog score
90
60
30
0
0.00
0.25
0.50
0.75
1.00
distance

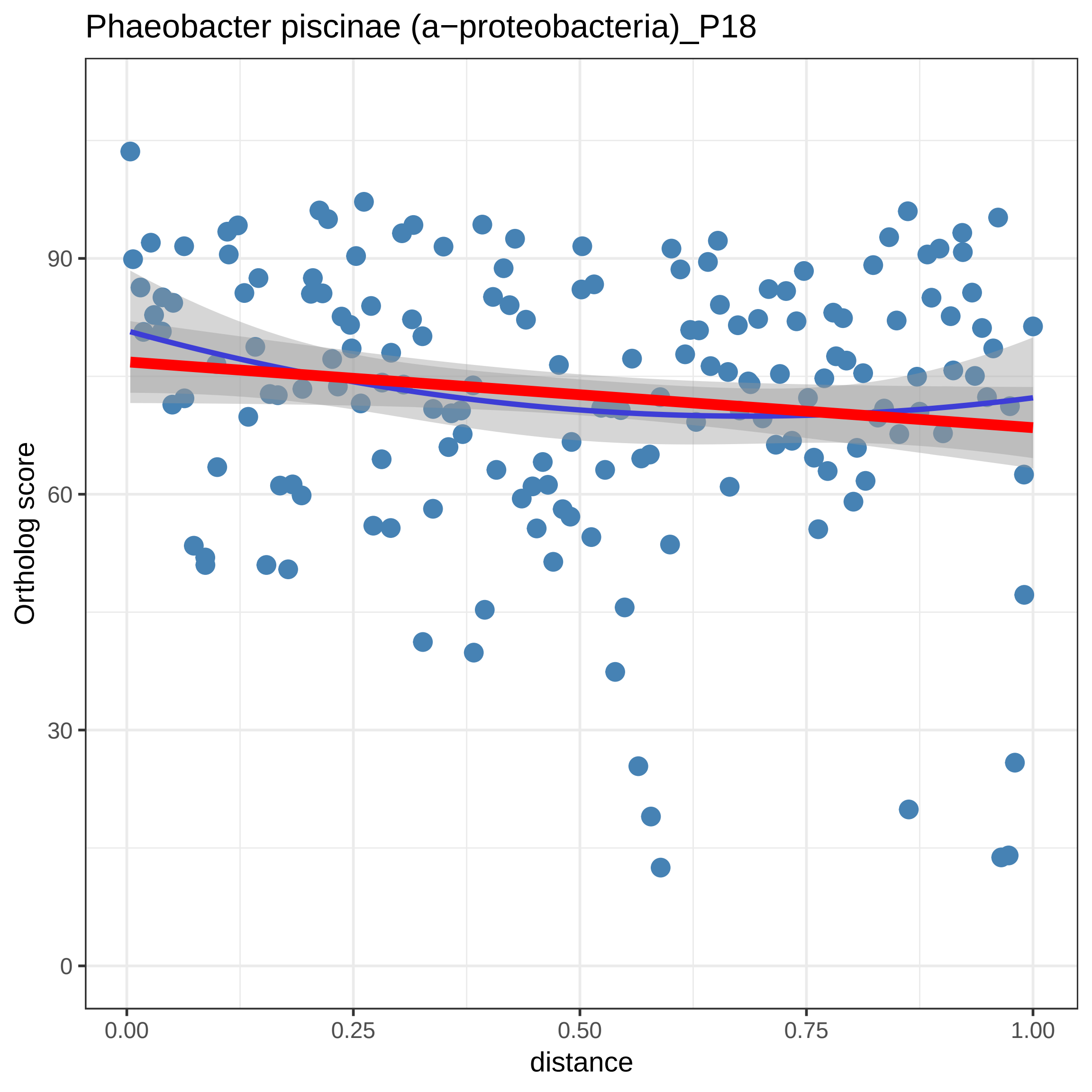

Phaeobacter piscinae (a−proteobacteria)_P18
Ortholog score
90
60
30
0
0.00
0.25
0.50
0.75
1.00
distance

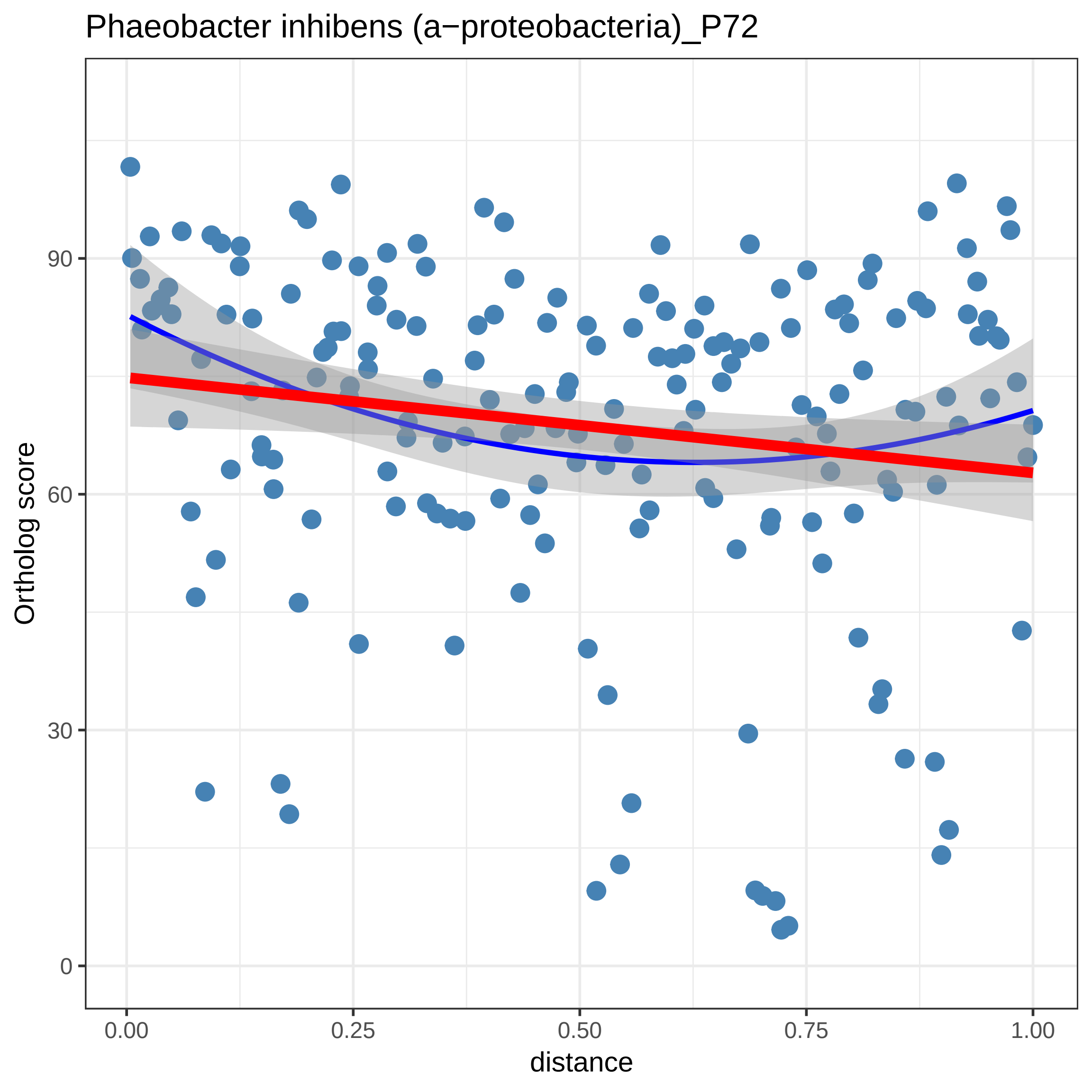

Phaeobacter inhibens (a−proteobacteria)_P72
Ortholog score
distance
0
30
60
90
0.00
0.25
0.50
0.75
1.00

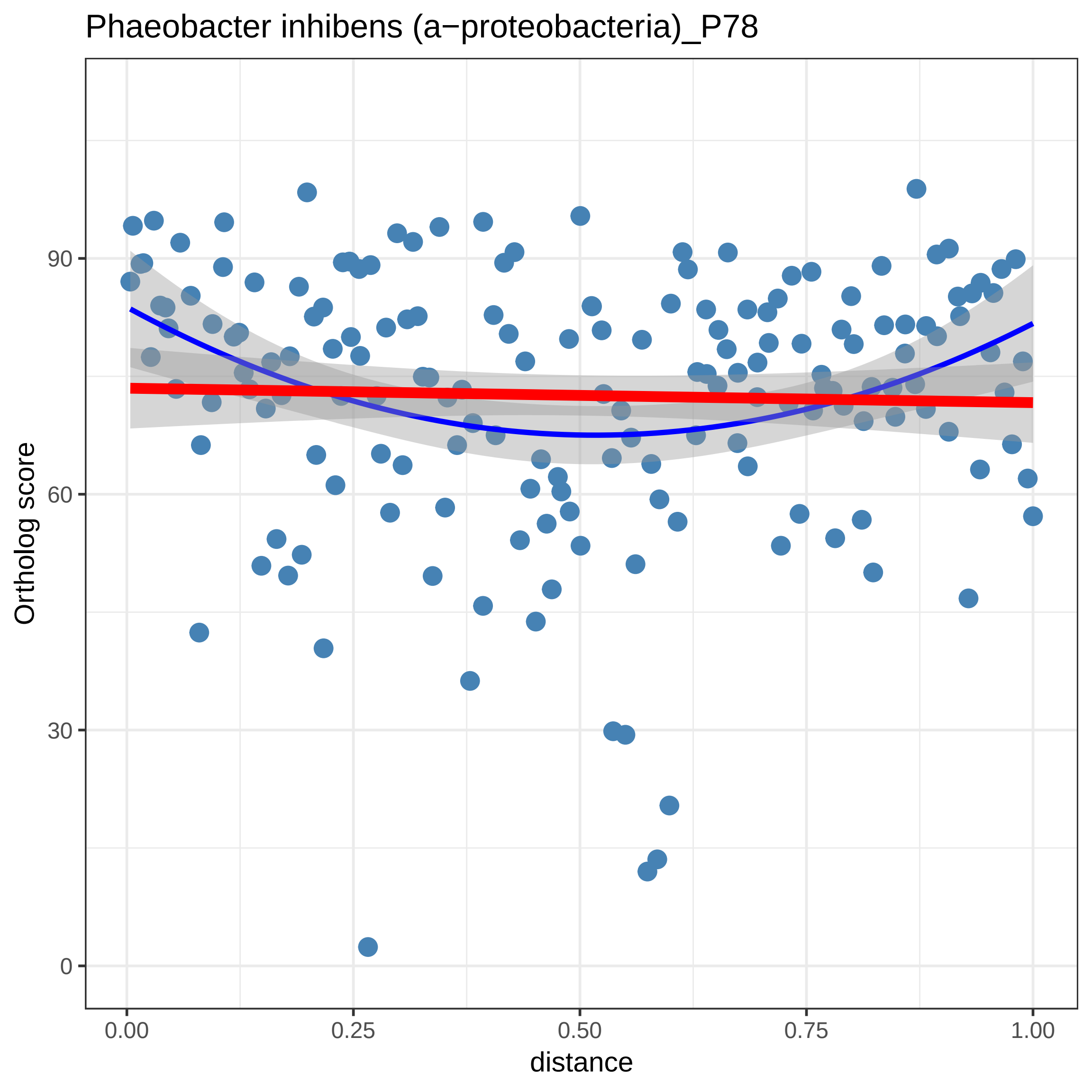

Phaeobacter inhibens (a−proteobacteria)_P78
Ortholog score
90
60
30
0
0.00
0.25
0.50
0.75
1.00
distance

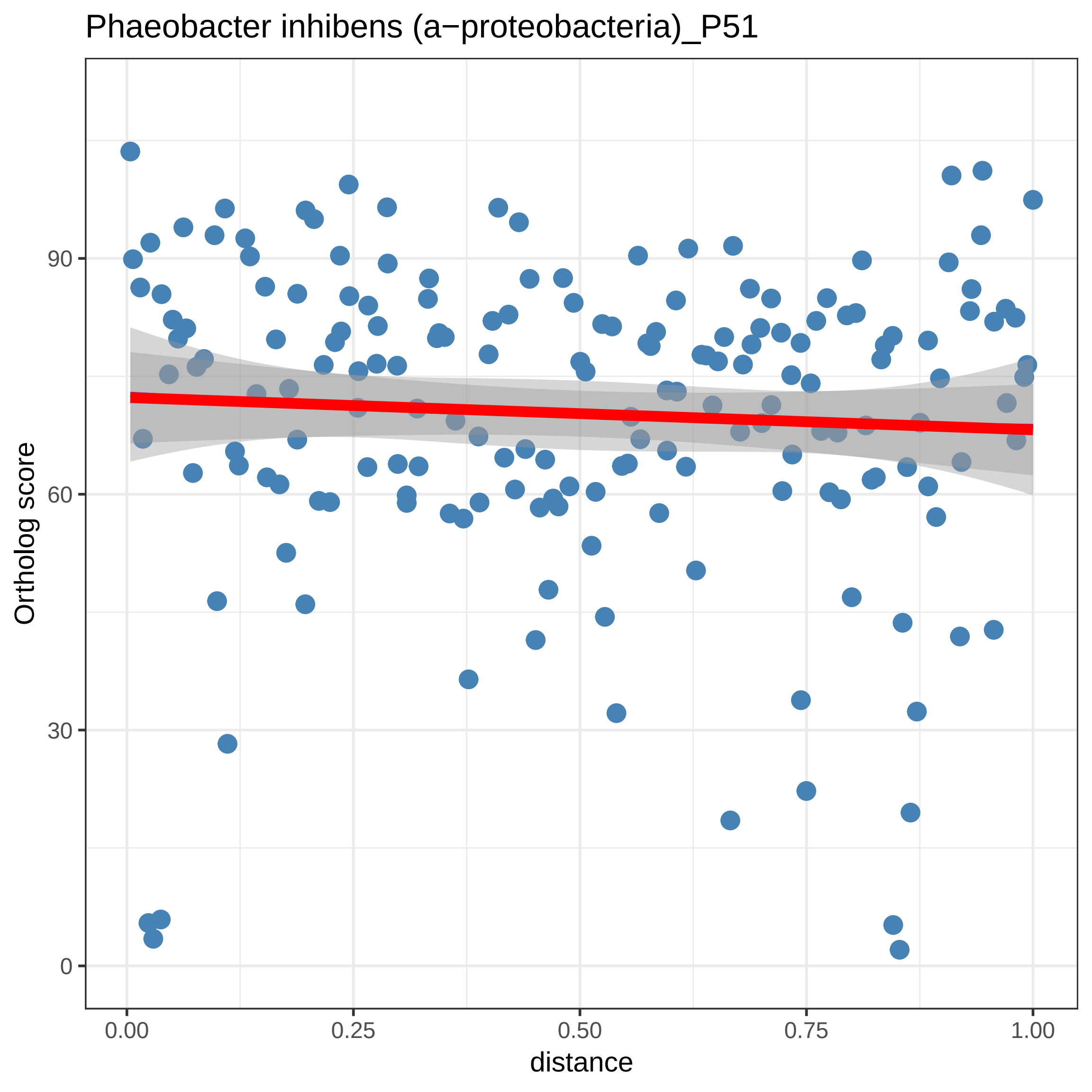
Phaeobacter inhibens (a−proteobacteria)_P51
Ortholog score
distance
0
30
60
90
0.00
0.25
0.50
0.75
1.00

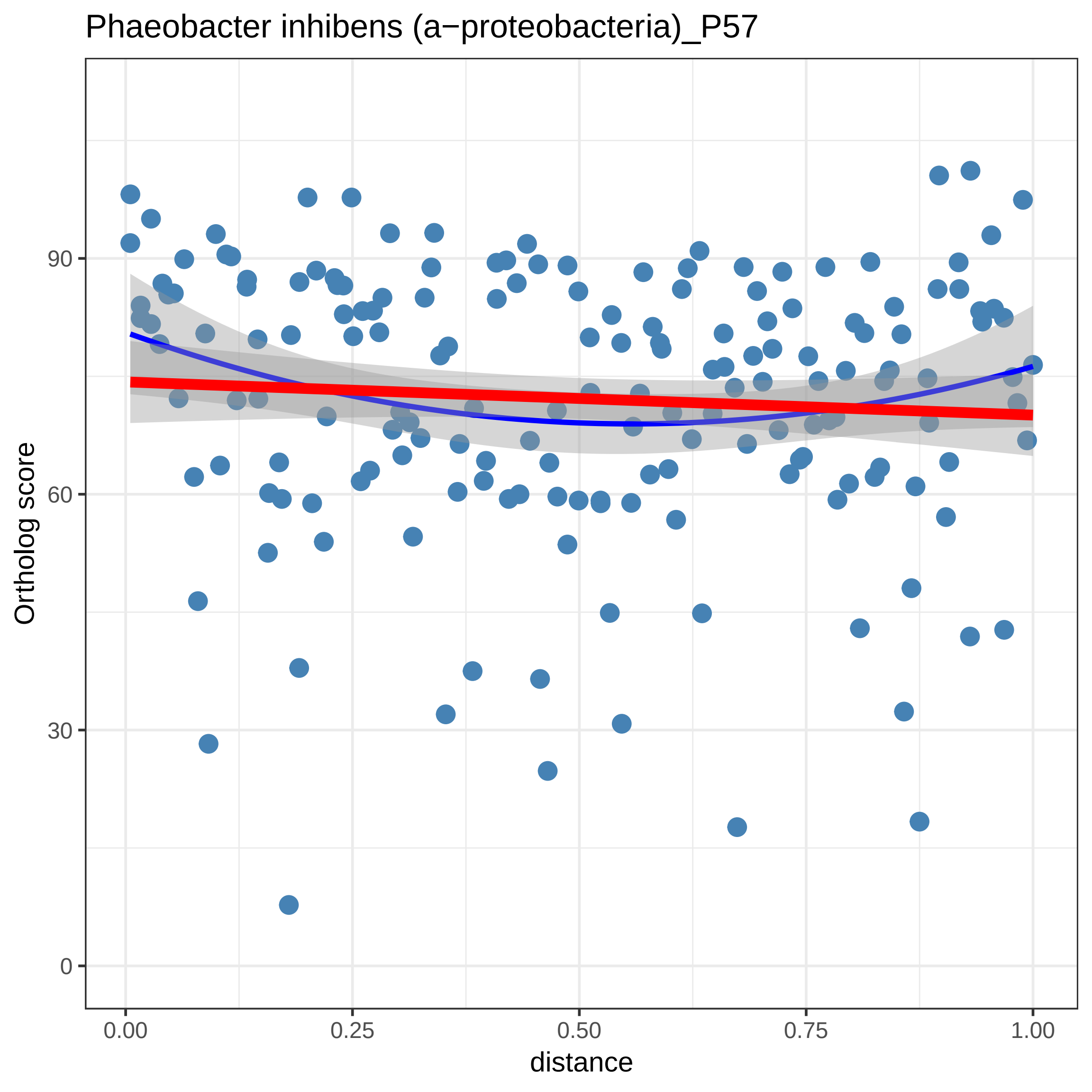

Phaeobacter inhibens (a−proteobacteria)_P57
Ortholog score
0
30
60
90
distance
0.00
0.25
0.50
0.75
1.00

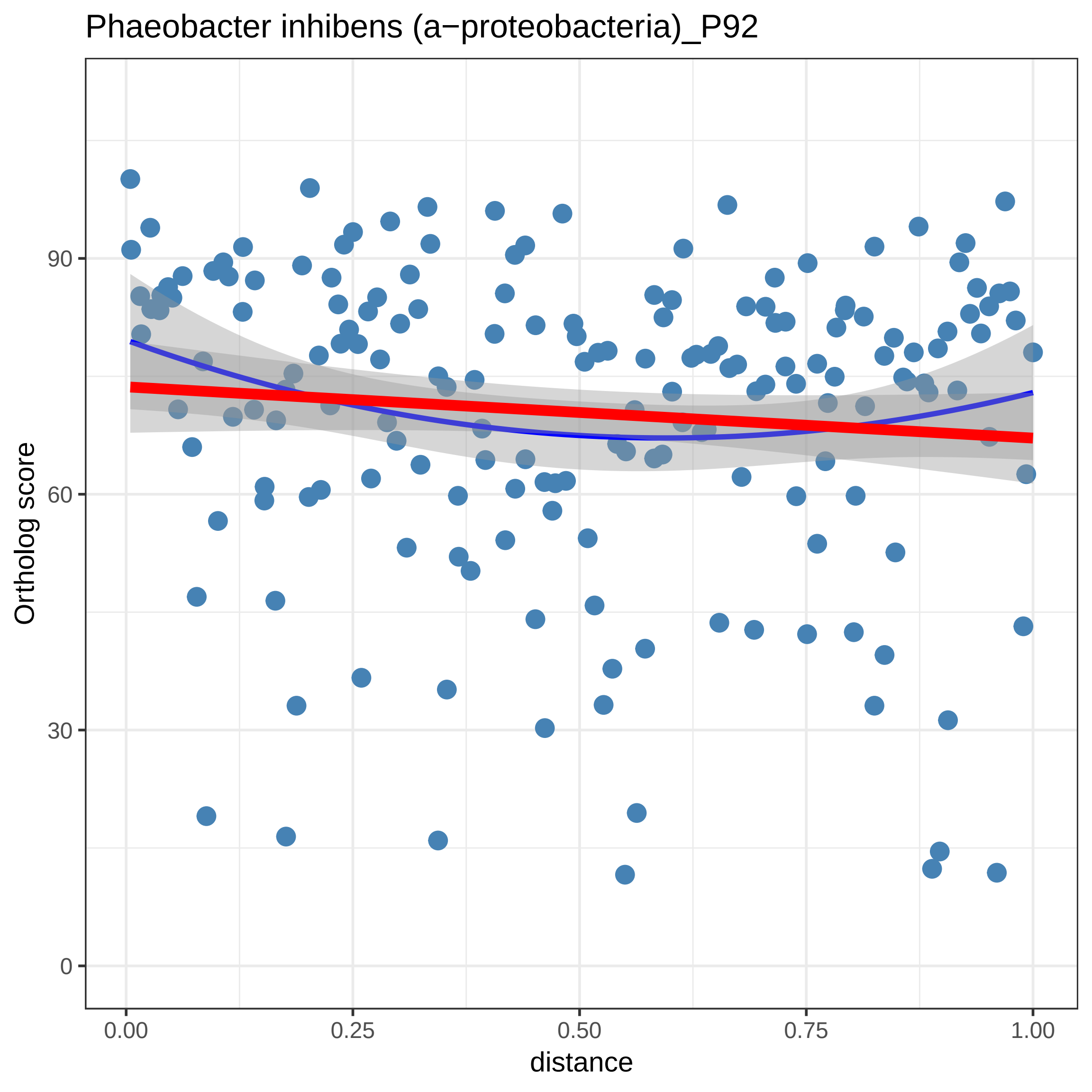

Phaeobacter inhibens (a−proteobacteria)_P92
Ortholog score
0
30
60
90
distance
0.00
0.25
0.50
0.75
1.00

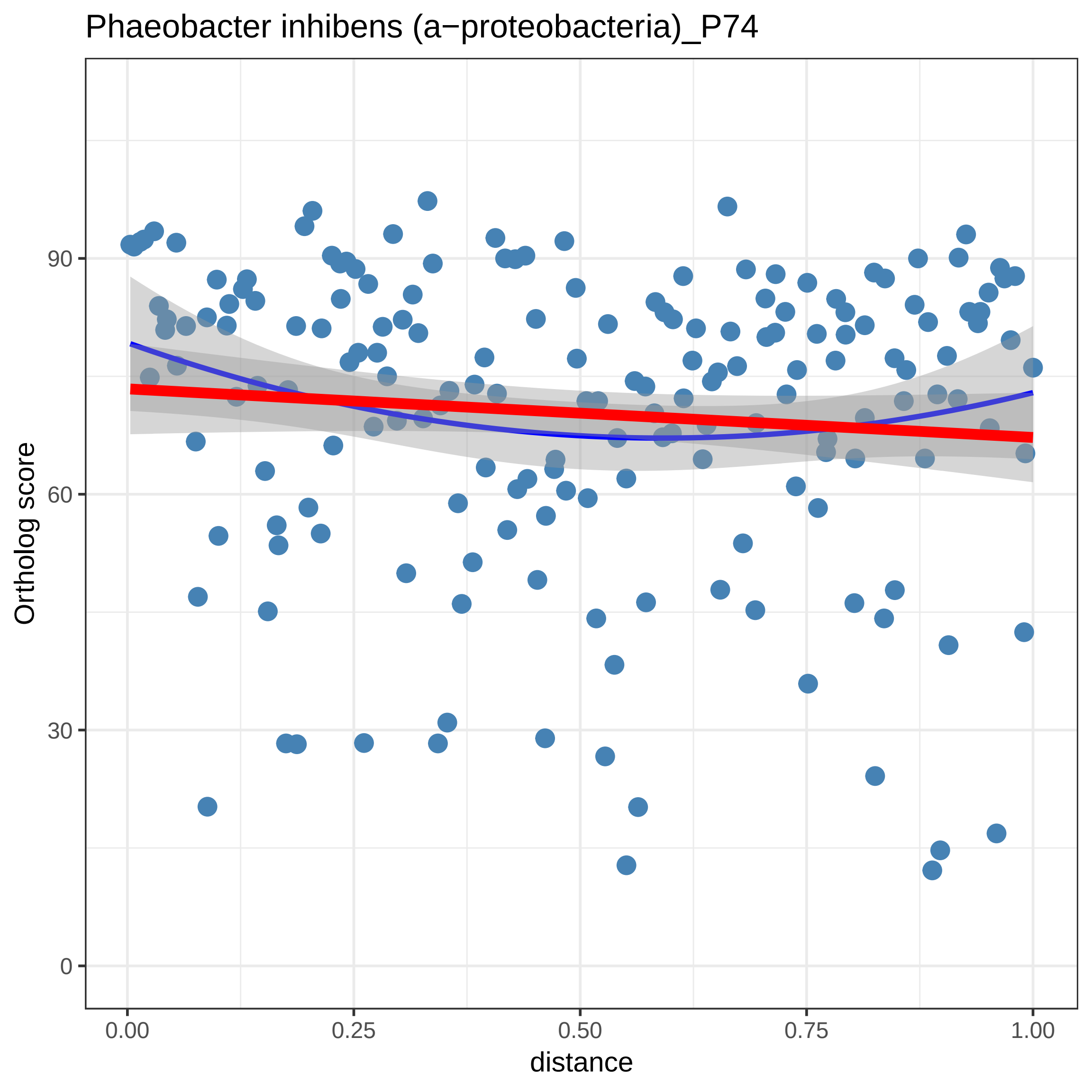

Phaeobacter inhibens (a−proteobacteria)_P74
Ortholog score
90
60
30
0
0.00
0.25
0.50
0.75
1.00
distance

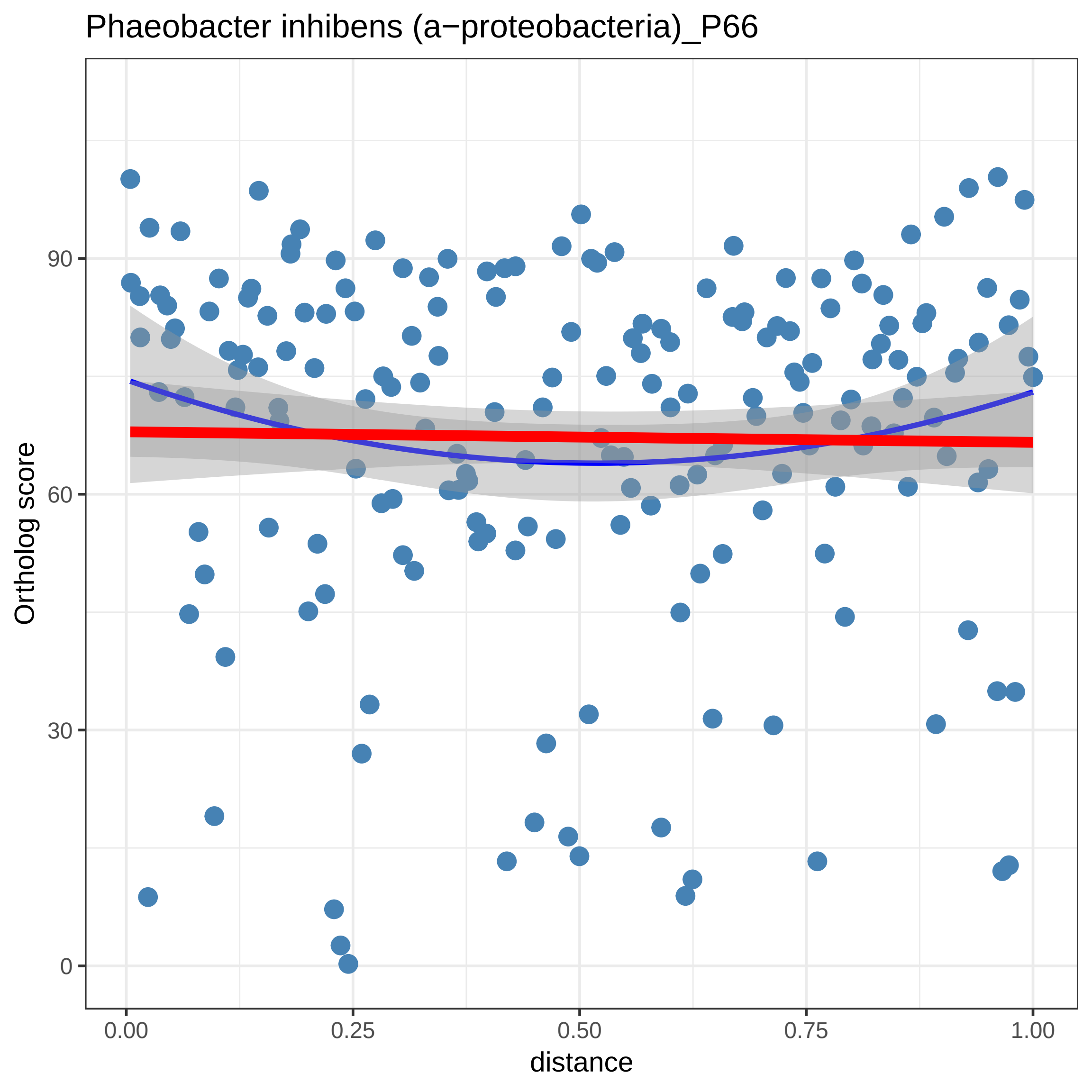

Phaeobacter inhibens (a−proteobacteria)_P66
Ortholog score
distance
0
30
60
90
0.00
0.25
0.50
0.75
1.00

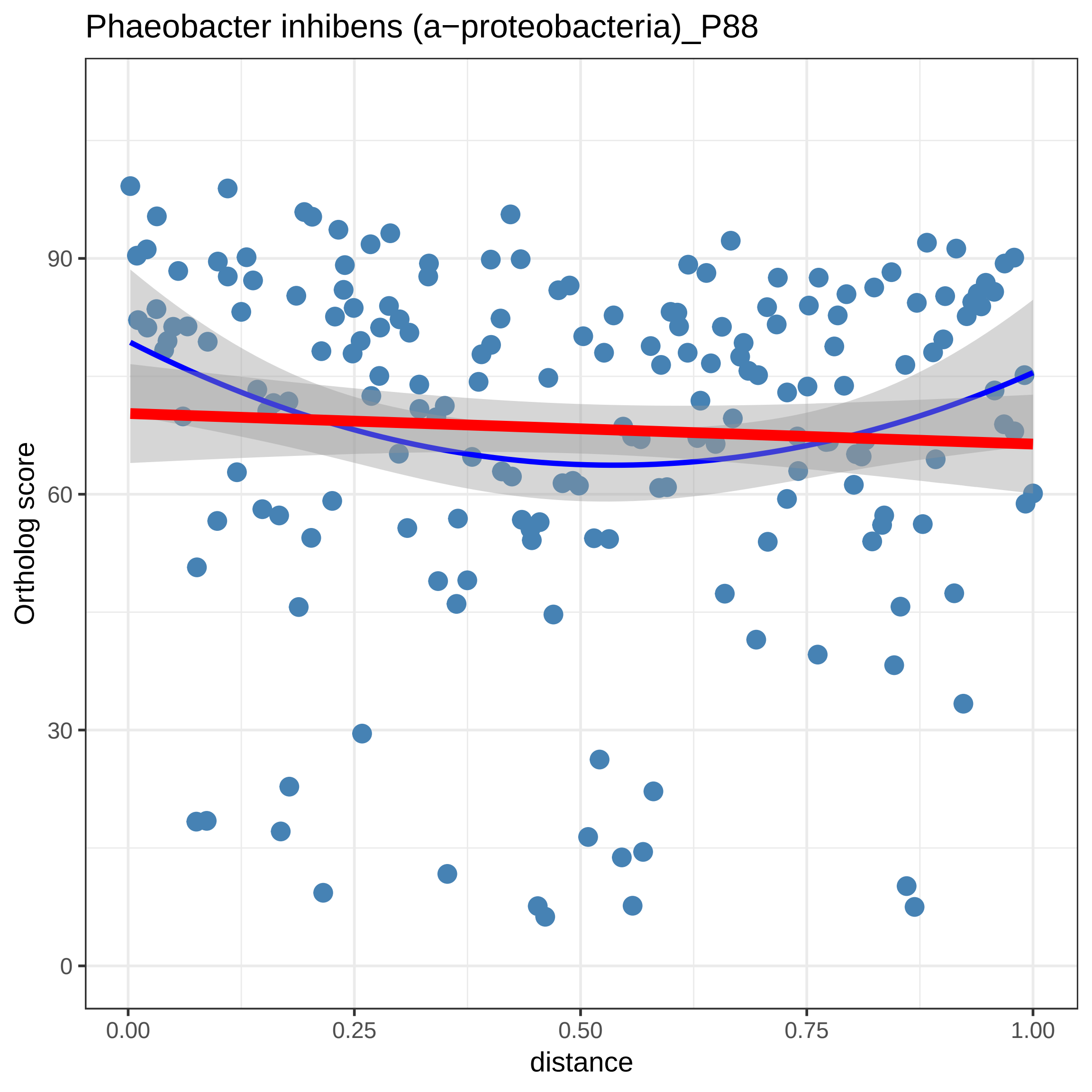

Phaeobacter inhibens (a−proteobacteria)_P88
Ortholog score
distance
0
30
60
90
0.00
0.25
0.50
0.75
1.00

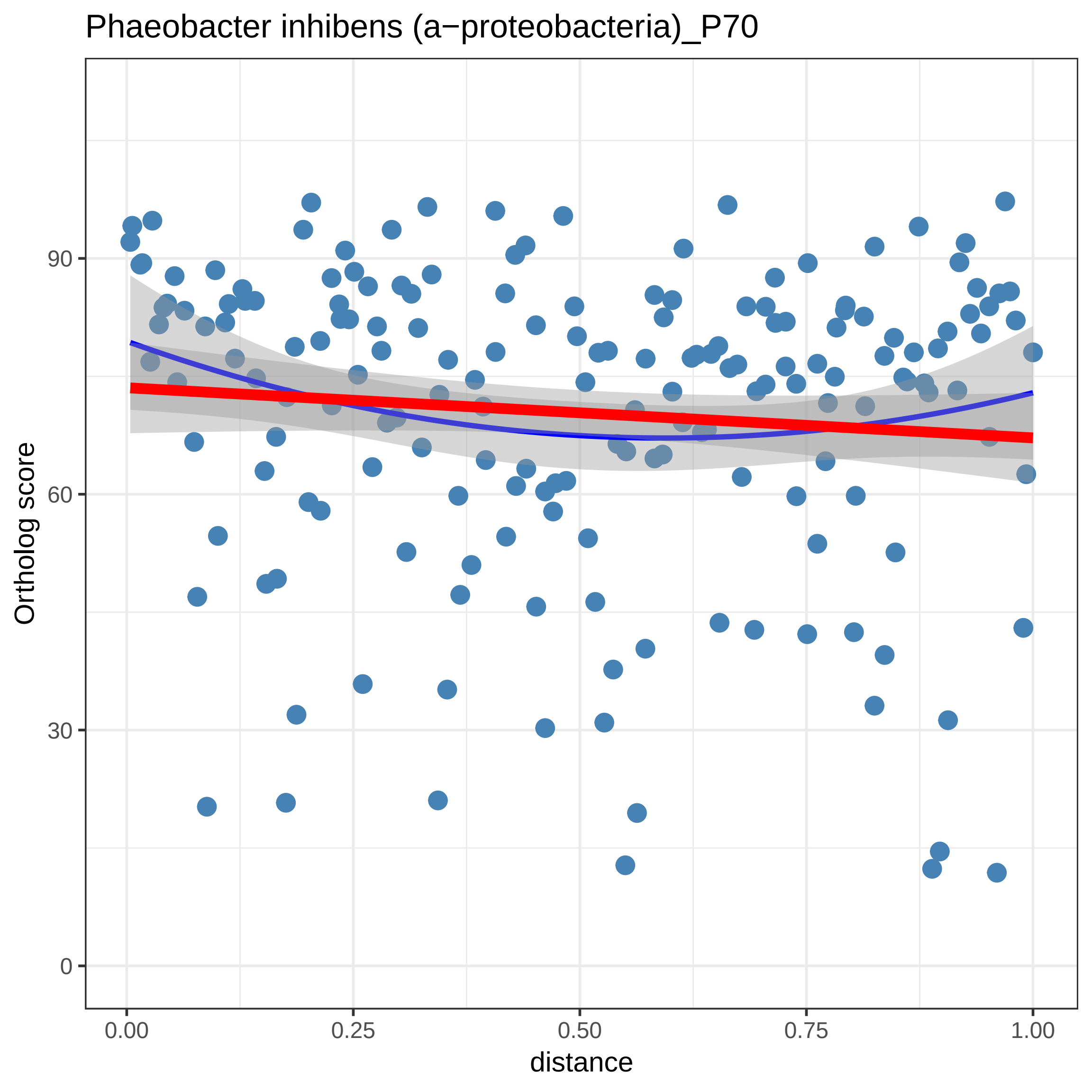

Phaeobacter inhibens (a−proteobacteria)_P70
Ortholog score
90
60
30
0
0.00
0.25
0.50
0.75
1.00
distance

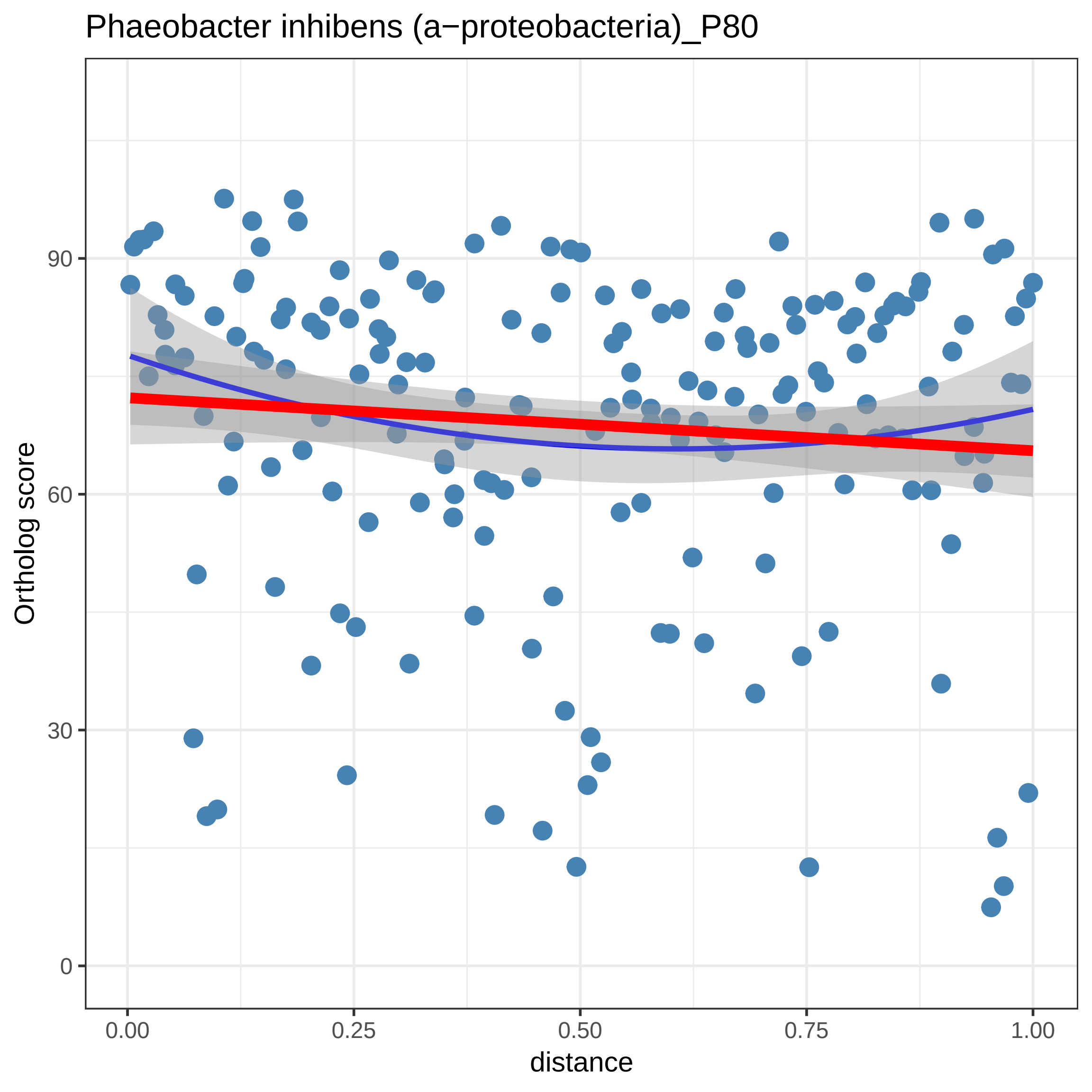
Phaeobacter inhibens (a−proteobacteria)_P80
Ortholog score
distance
0
30
60
90
0.00
0.25
0.50
0.75
1.00

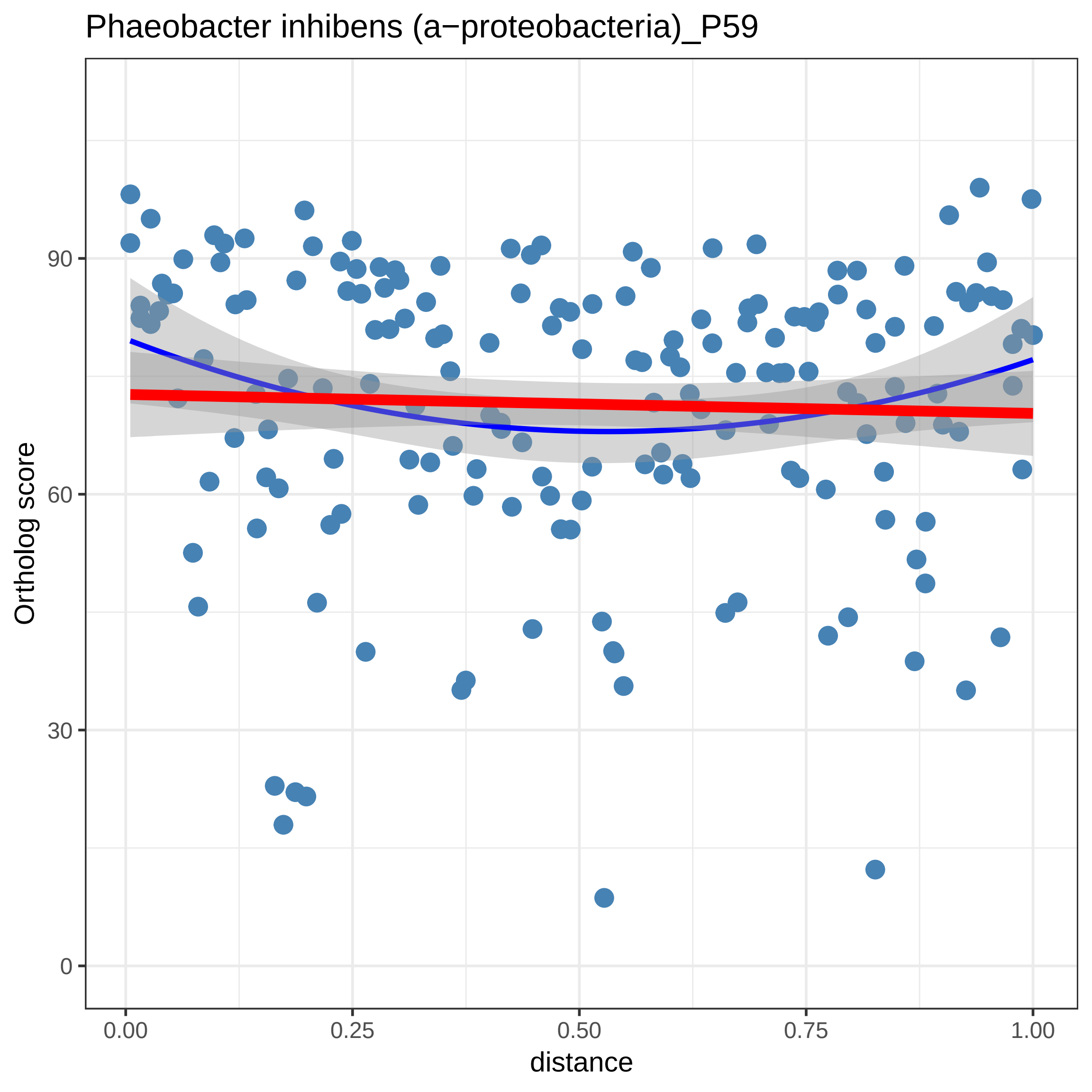
Phaeobacter inhibens (a−proteobacteria)_P59
Ortholog score
90
60
30
0
0.00
0.25
0.50
0.75
1.00
distance

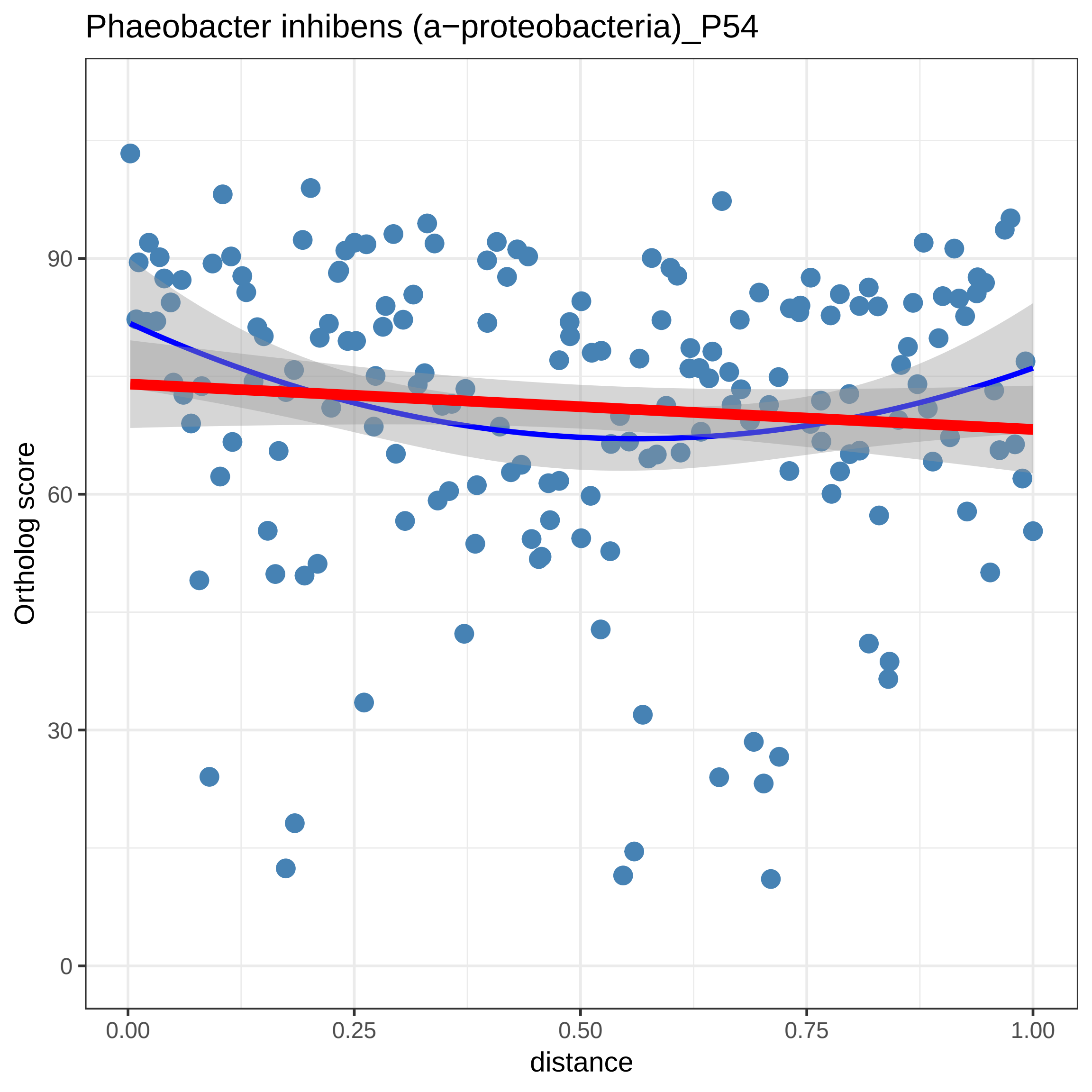
Phaeobacter inhibens (a−proteobacteria)_P54
Ortholog score
distance
0
30
60
90
0.00
0.25
0.50
0.75
1.00

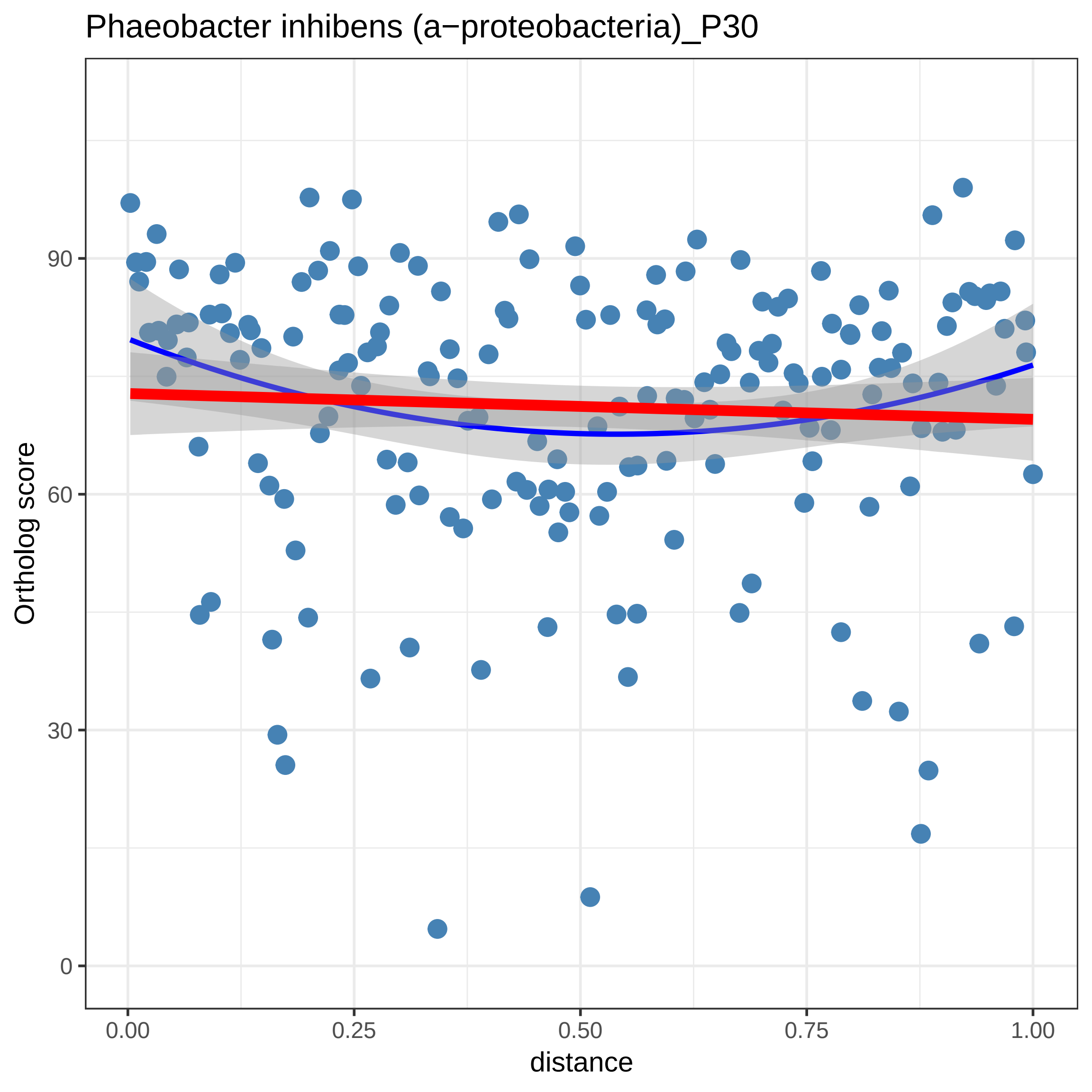
Phaeobacter inhibens (a−proteobacteria)_P30
Ortholog score
distance
0
30
60
90
0.00
0.25
0.50
0.75
1.00

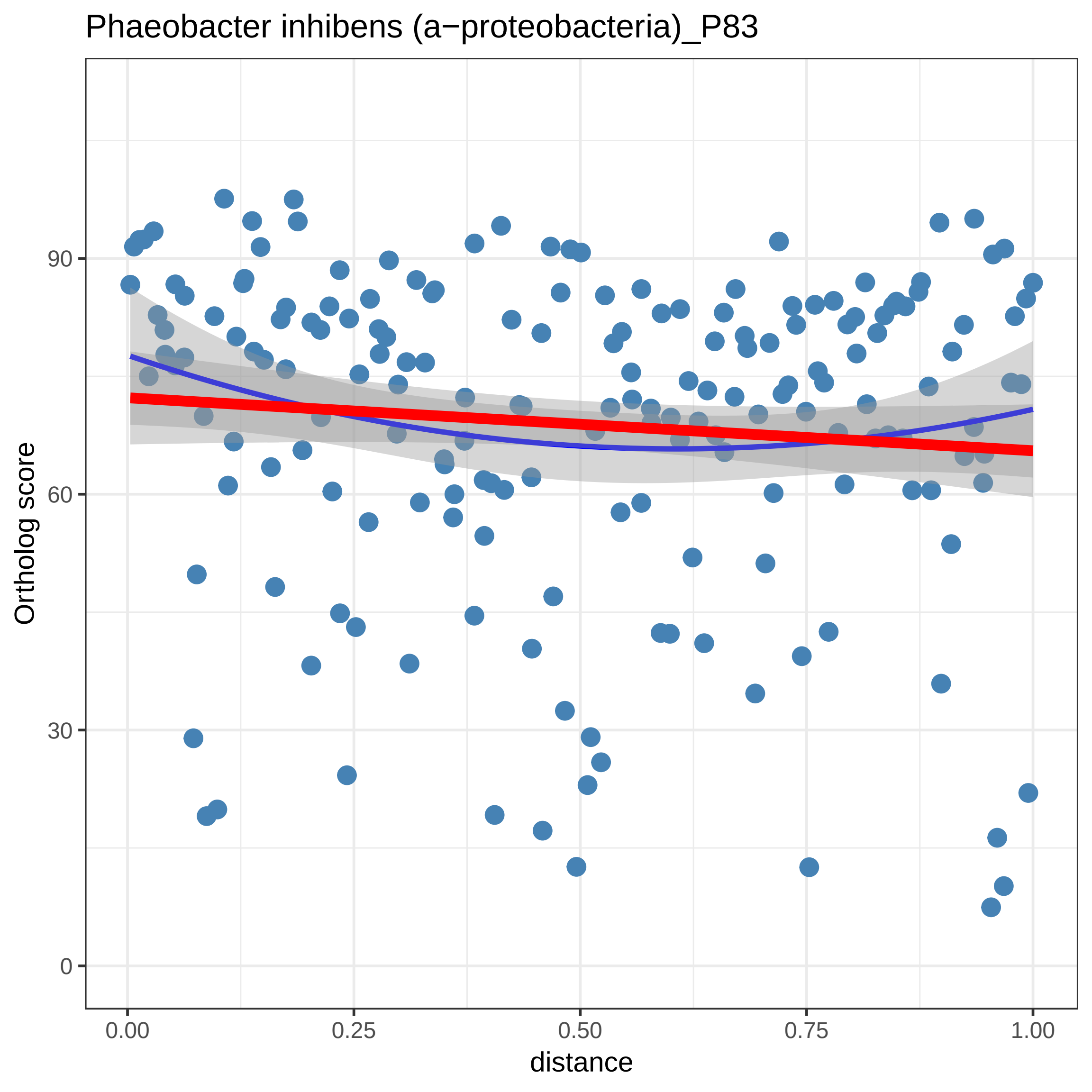

Phaeobacter inhibens (a−proteobacteria)_P83
Ortholog score
distance
0
30
60
90
0.00
0.25
0.50
0.75
1.00

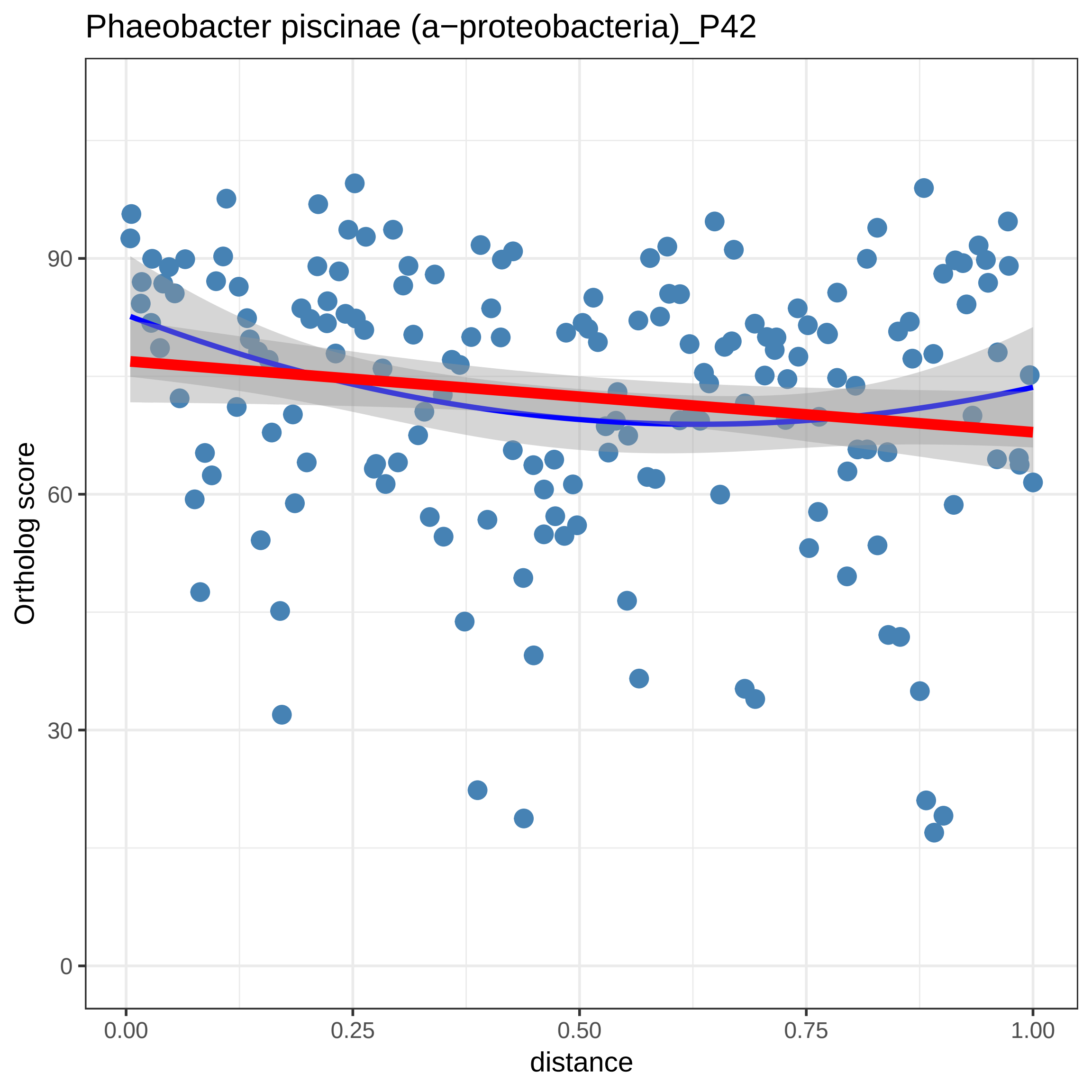
Phaeobacter piscinae (a−proteobacteria)_P42
Ortholog score
90
60
30
0
0.00
0.25
0.50
0.75
1.00
distance

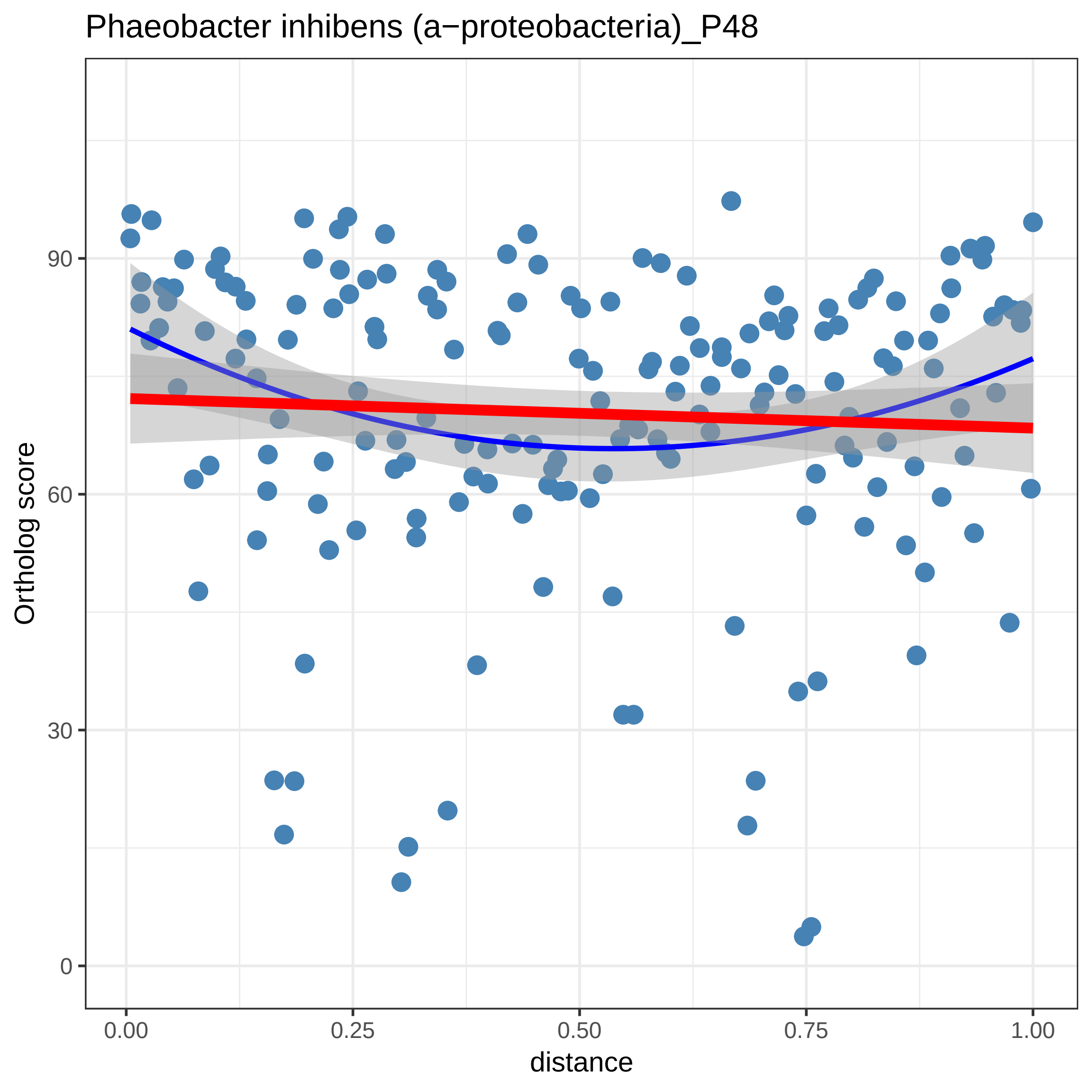

Phaeobacter inhibens (a−proteobacteria)_P48
Ortholog score
distance
0
30
60
90
0.00
0.25
0.50
0.75
1.00

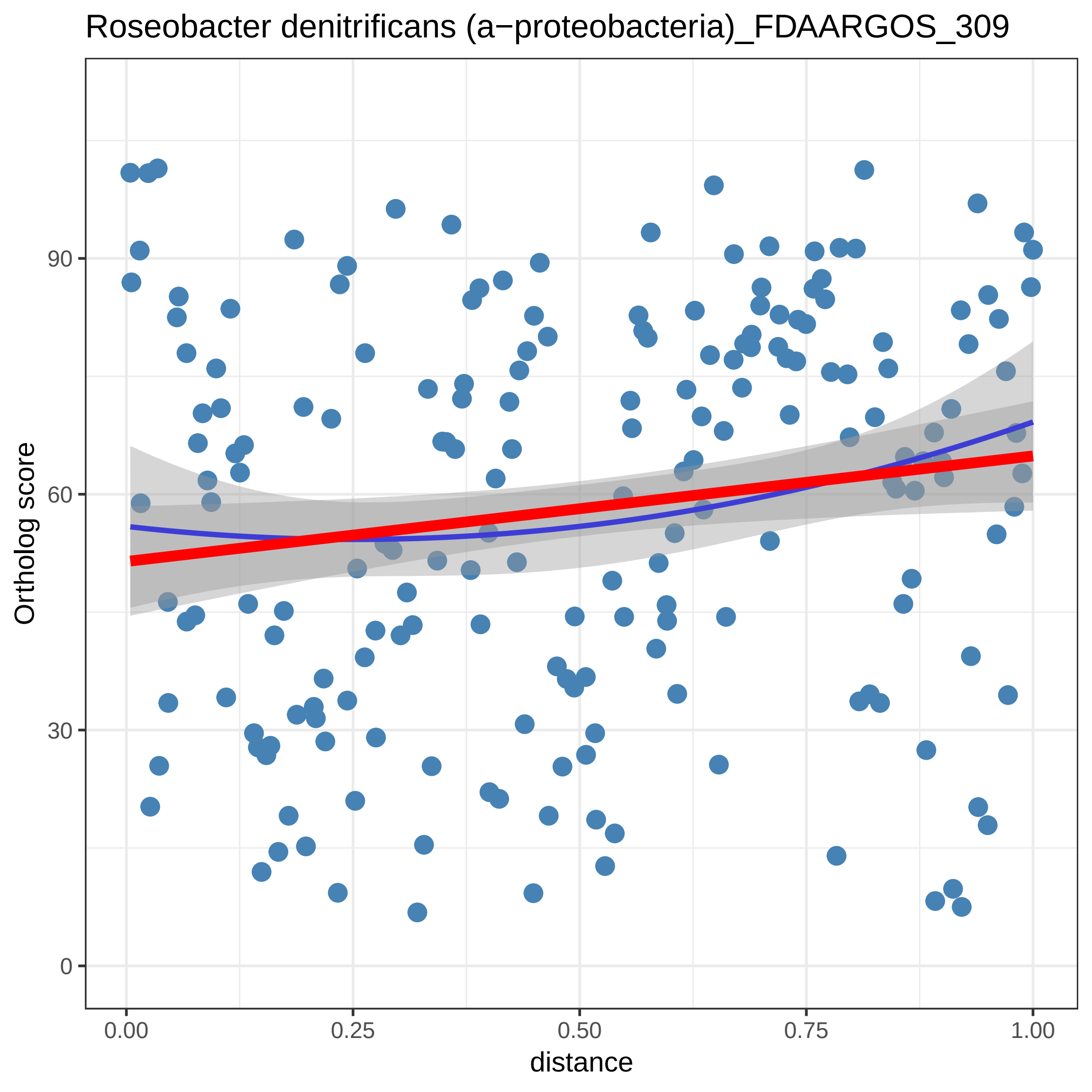

Roseobacter denitrificans (a−proteobacteria)_FDAARGOS_309
Ortholog score
distance
0
30
60
90
0.00
0.25
0.50
0.75
1.00

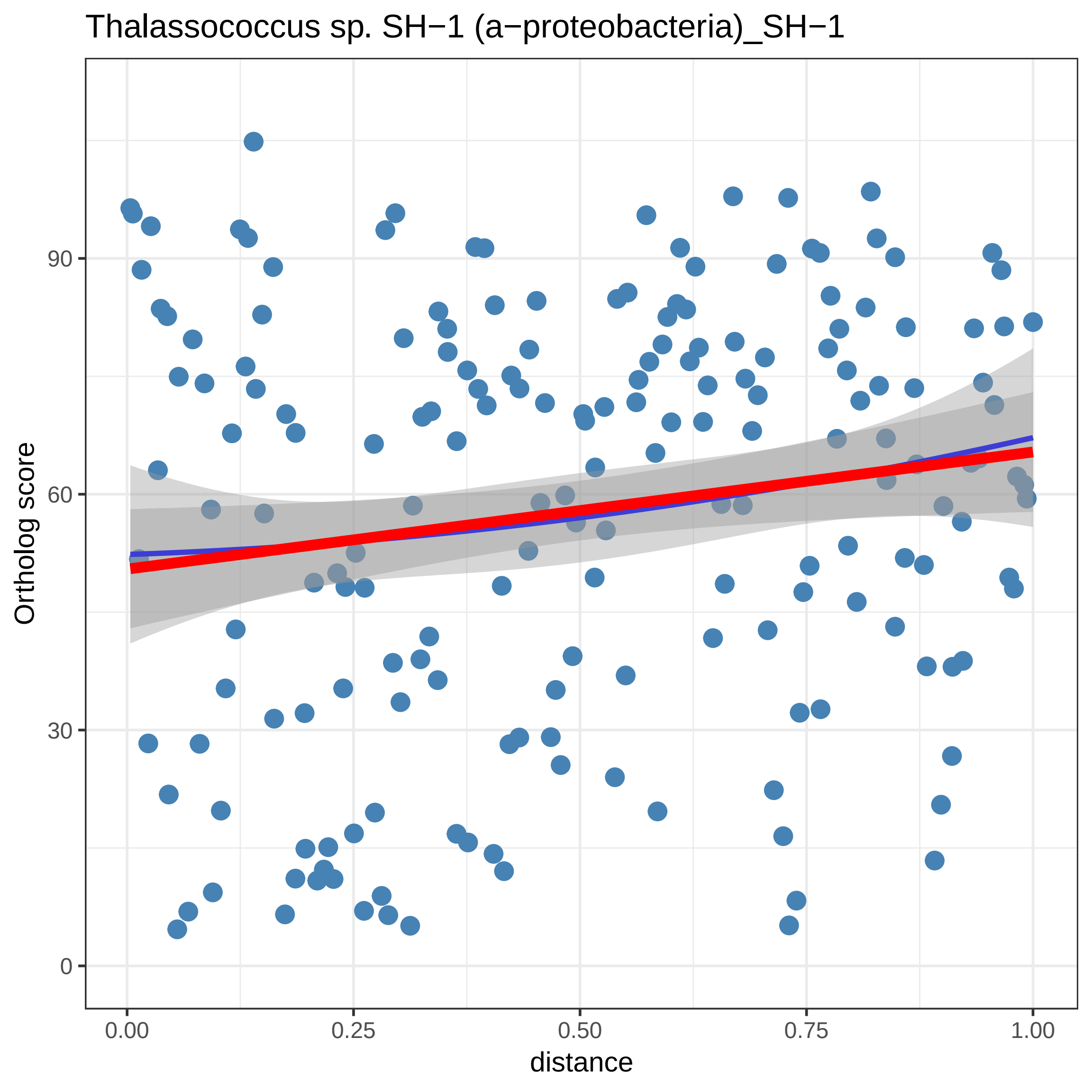
Thalassococcus sp. SH−1 (a−proteobacteria)_SH−1
Ortholog score
90
60
30
0
0.00
0.25
0.50
0.75
1.00
distance

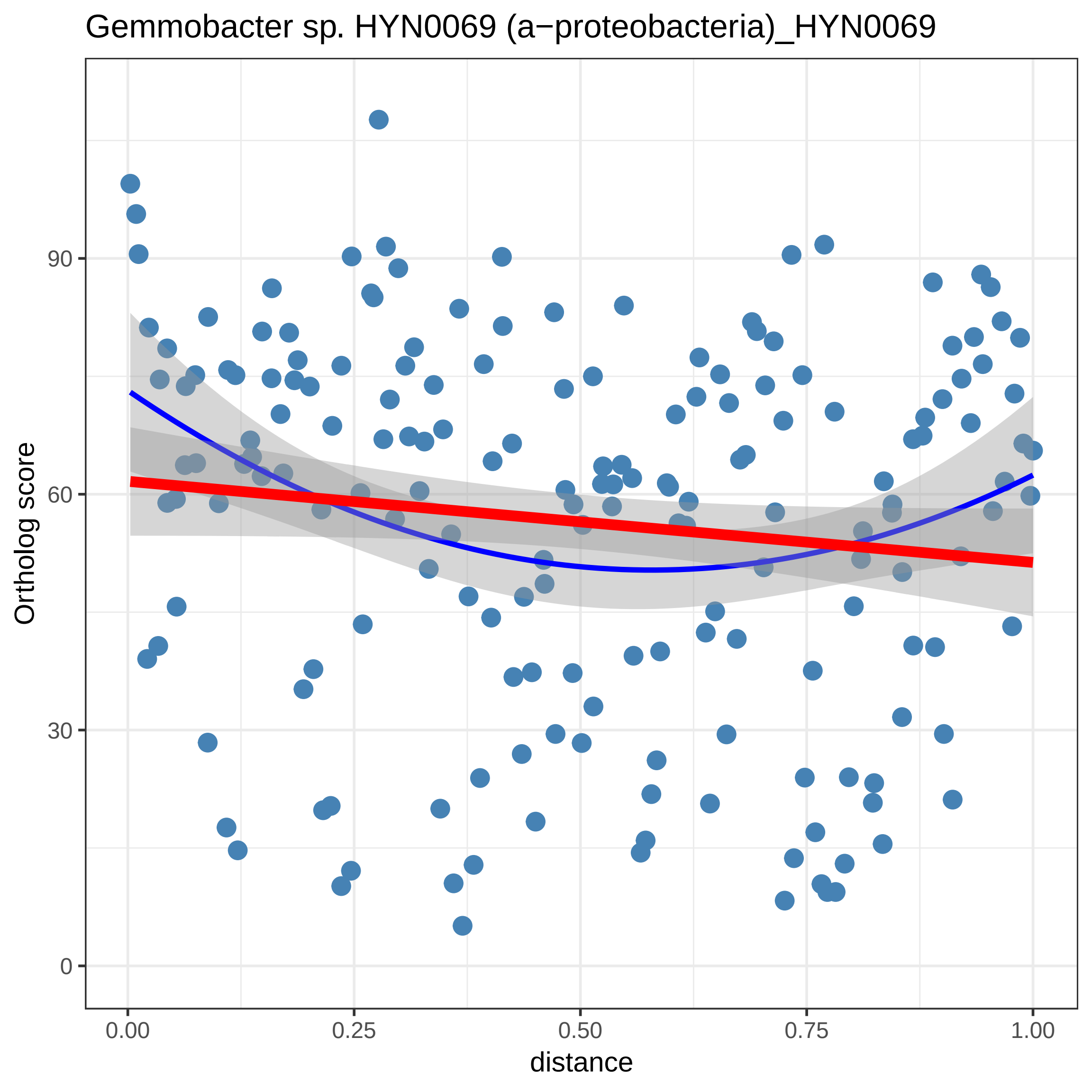

Gemmobacter sp. HYN0069 (a−proteobacteria)_HYN0069
Ortholog score
90
60
30
0
0.00
0.25
0.50
0.75
1.00
distance

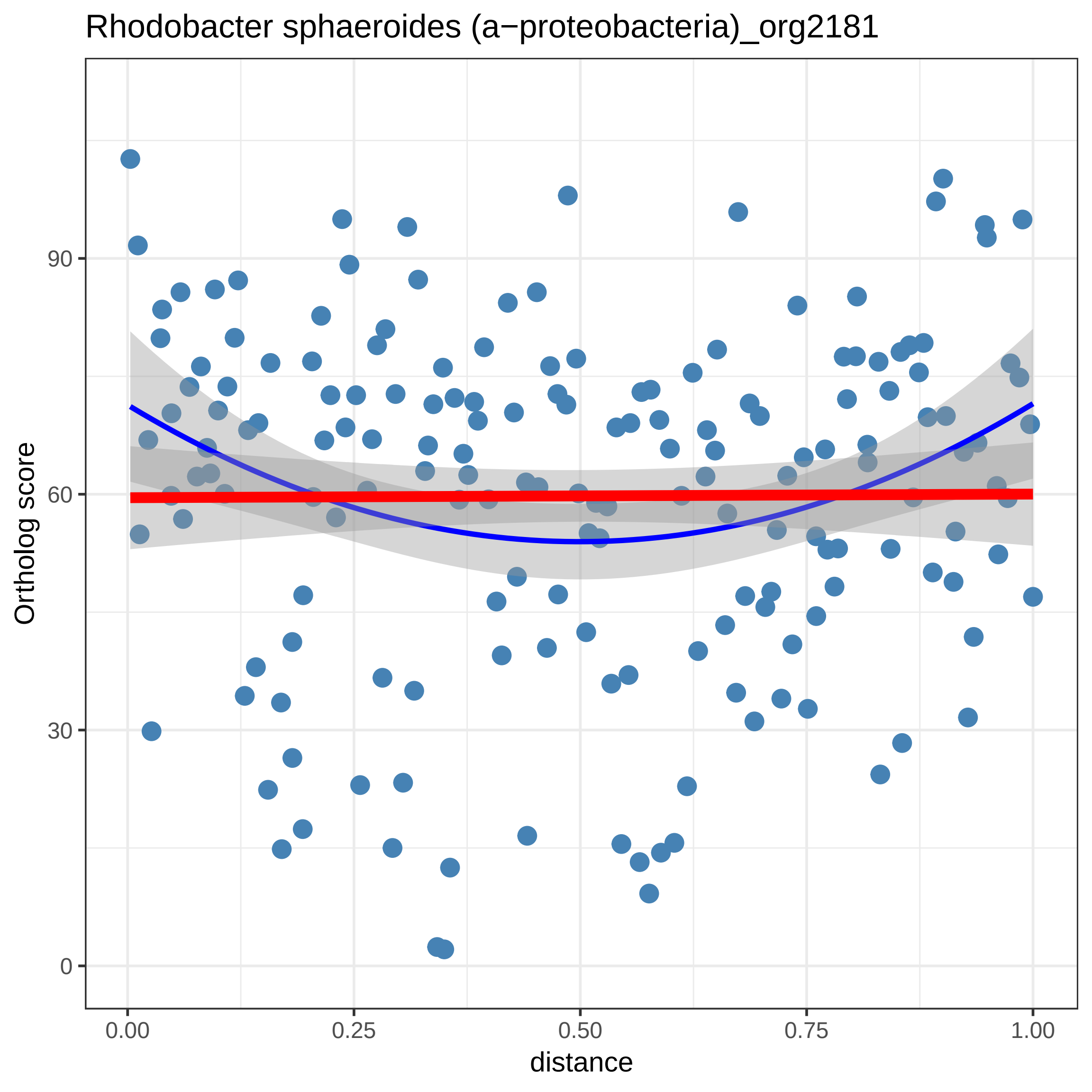
Rhodobacter sphaeroides (a−proteobacteria)_org2181
Ortholog score
90
60
30
0
0.00
0.25
0.50
0.75
1.00
distance

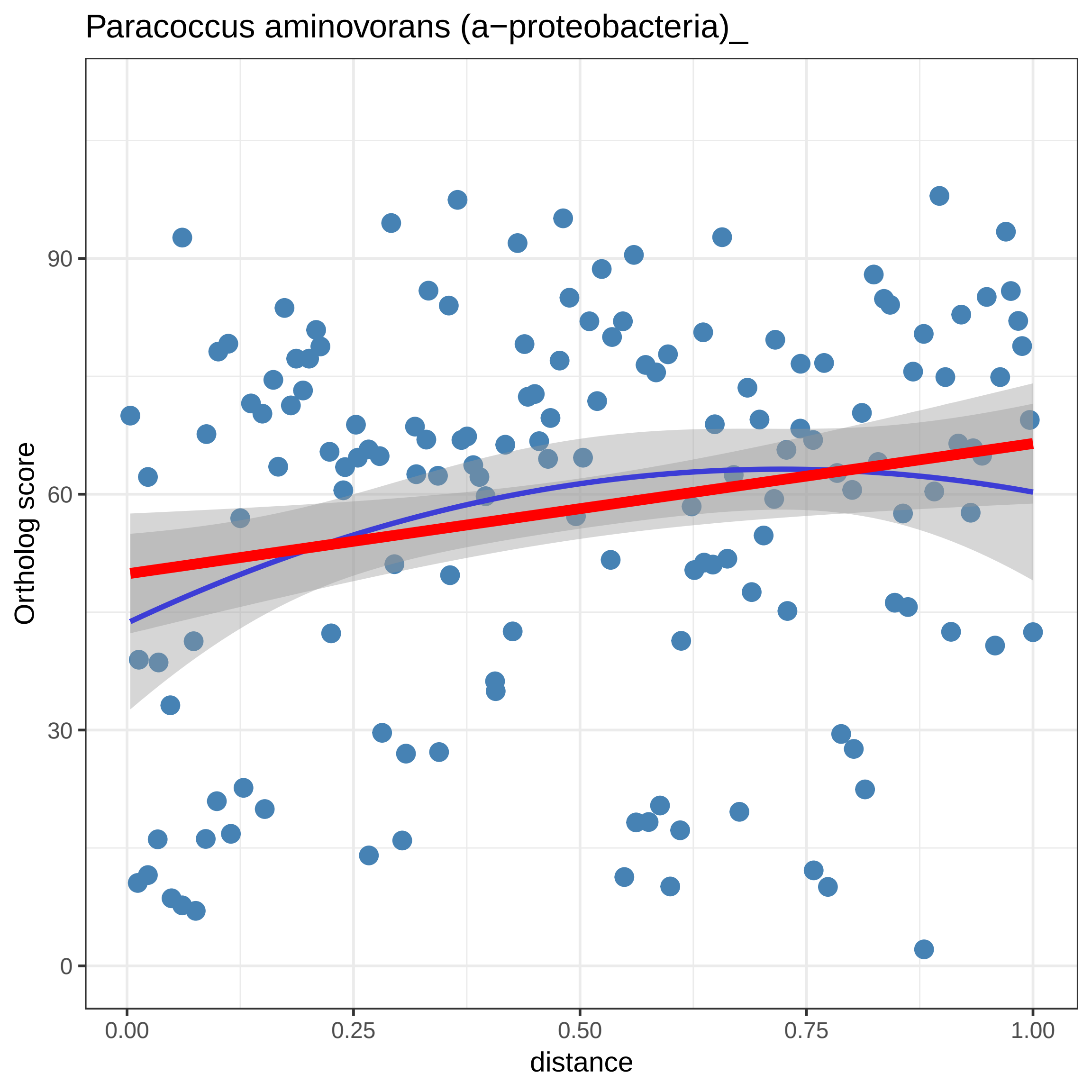
Paracoccus aminovorans (a−proteobacteria)_
Ortholog score
0
30
60
90
0.00
0.25
0.50
0.75
1.00
distance
