# Supplementary material for: Clustered Core- and Pan-Genome Content on Rhodobacteraceae Chromosomes
Source: Genome Biol Evol. 2019 Jul 3;11(8):2208–17. doi: 10.1093/gbe/evz138 (PMC6699656; doi:10.1093/gbe/evz138)
Supplement: evz138_Supplementary_Data [file evz138_supplementary_data.zip › Supplementary_Figure_S7_circular_chromosomes.pdf]

Antarctobacter heliothermus_SMS3

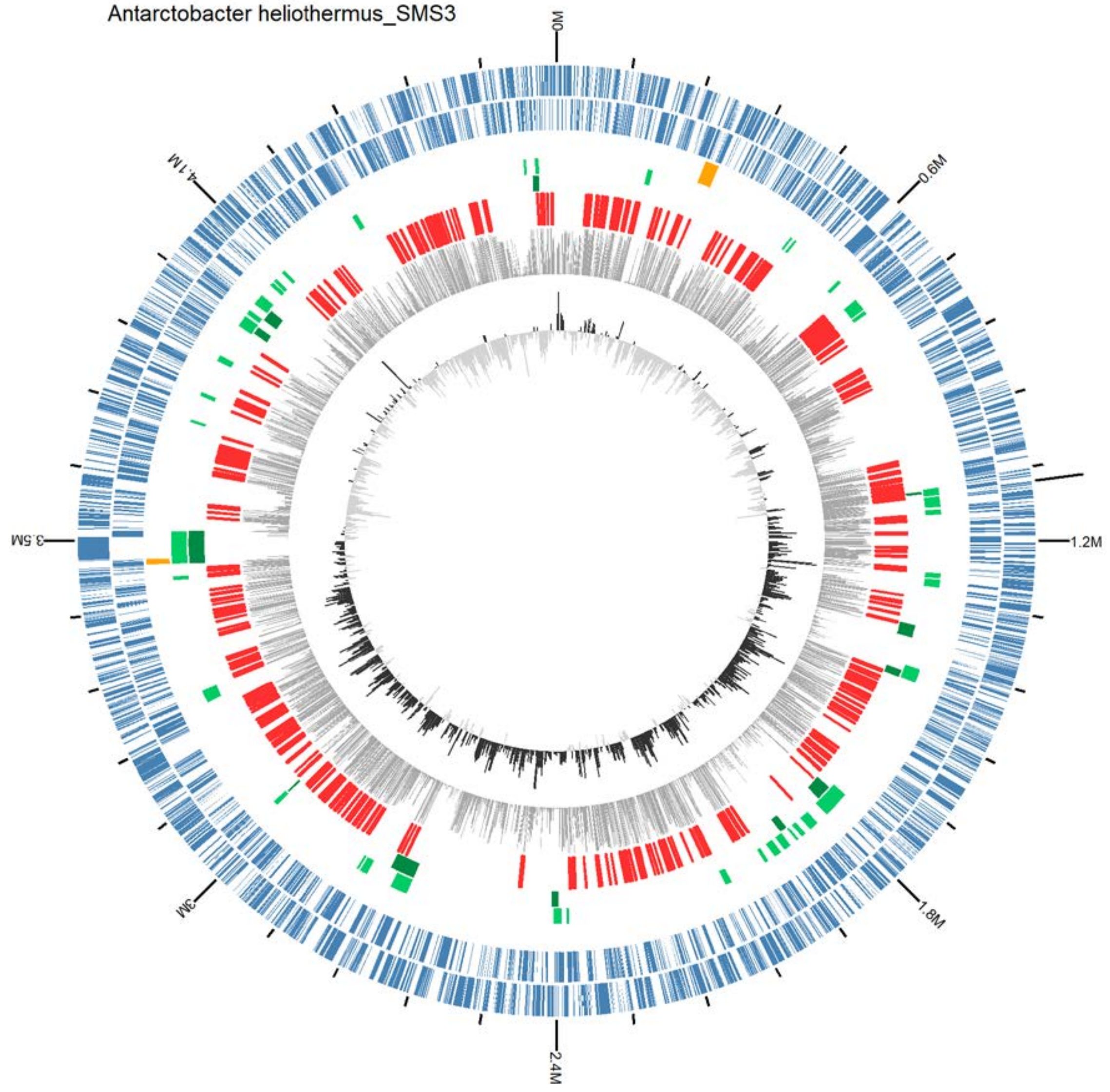

Celeribacter ethanolicus_TSPH2

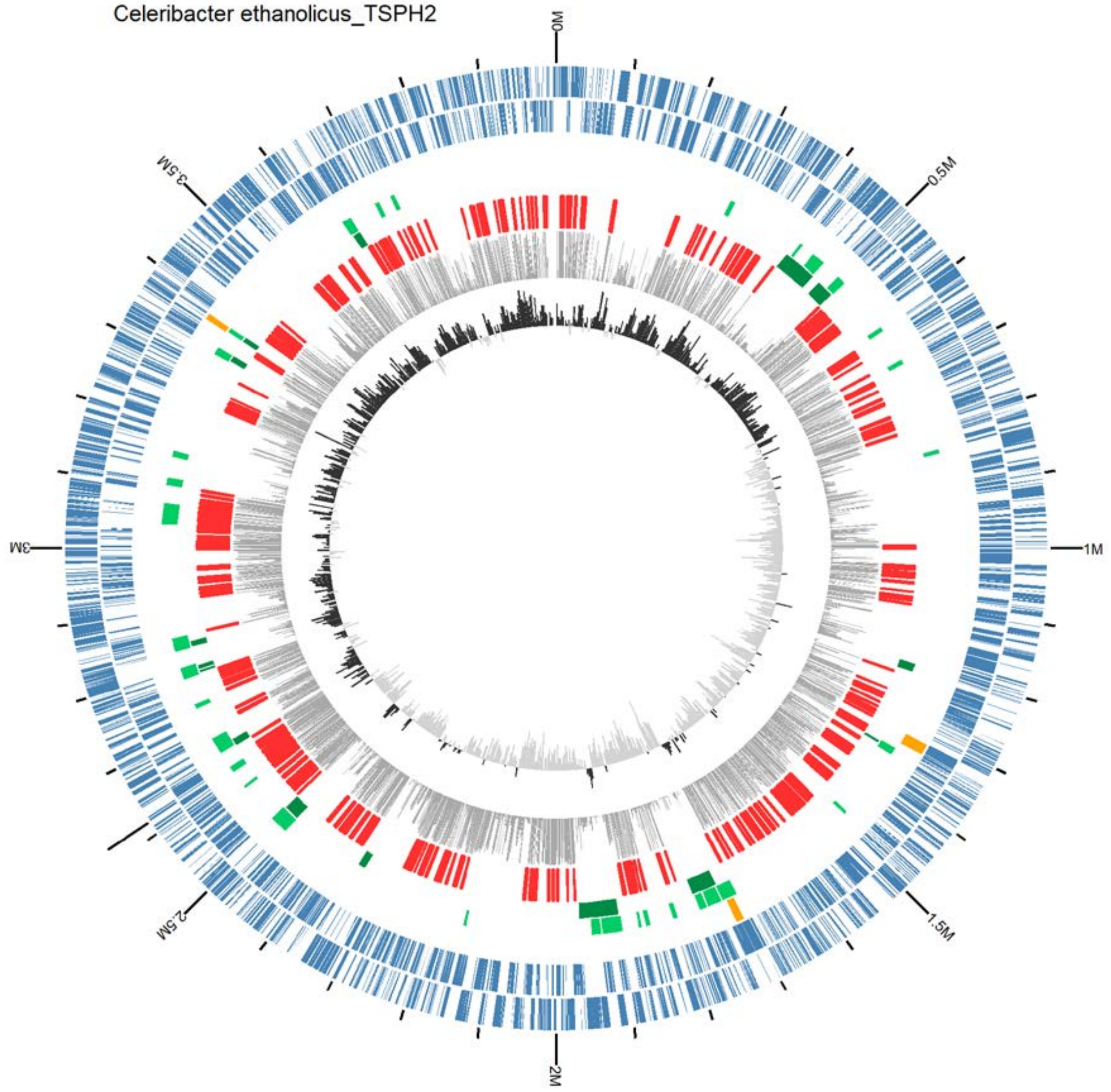

Celeribacter indicus_P73

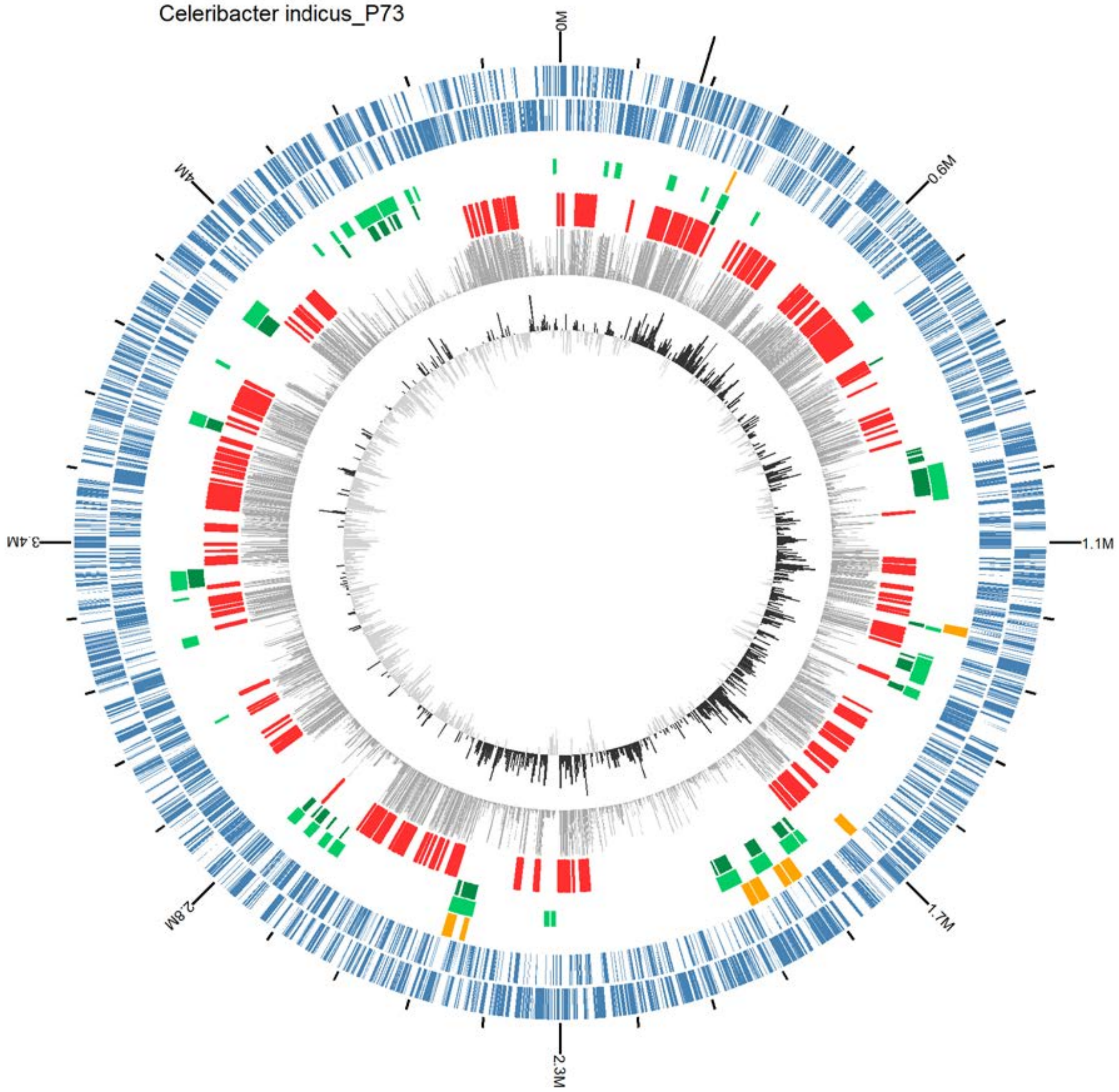

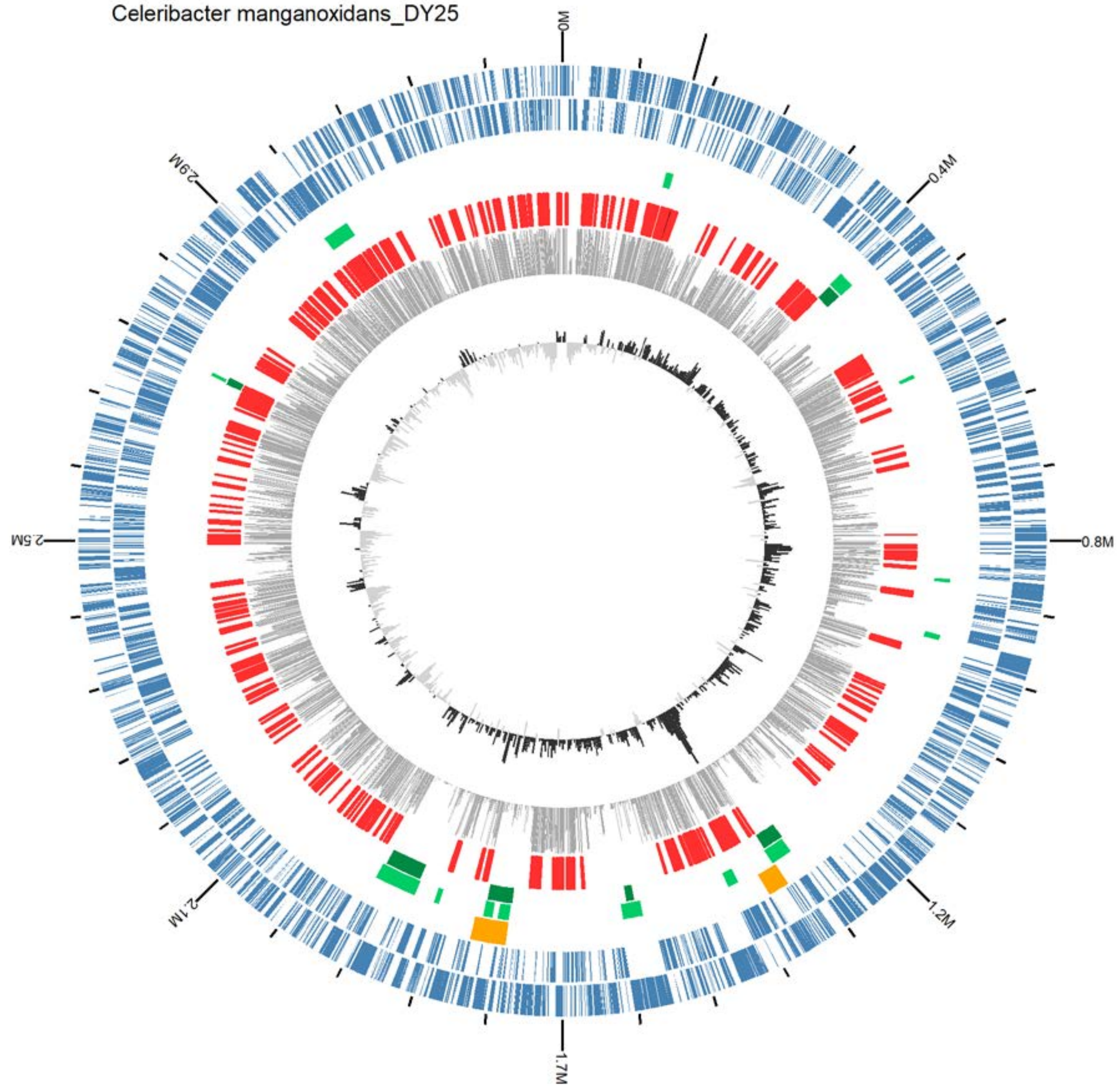

Celeribacter manganoxidans_DY25
0M
0.4M
0.8M
1.2M
1.7M
2.1M
2.5M
2.9M

Celeribacter marinus_IMCC 12053

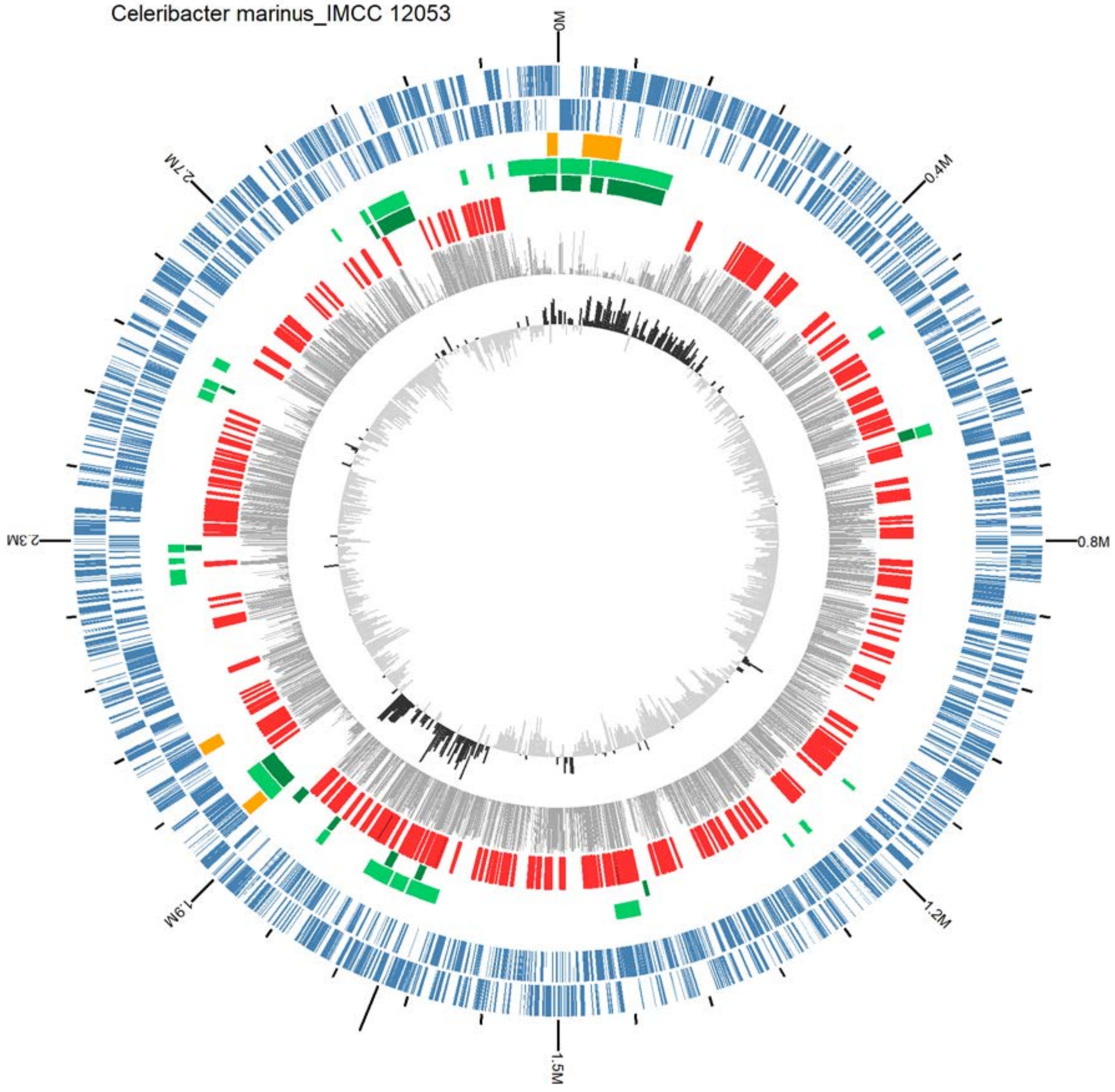

Confluentimicrobium sp. EMB200-NS6_EMB200-NS6

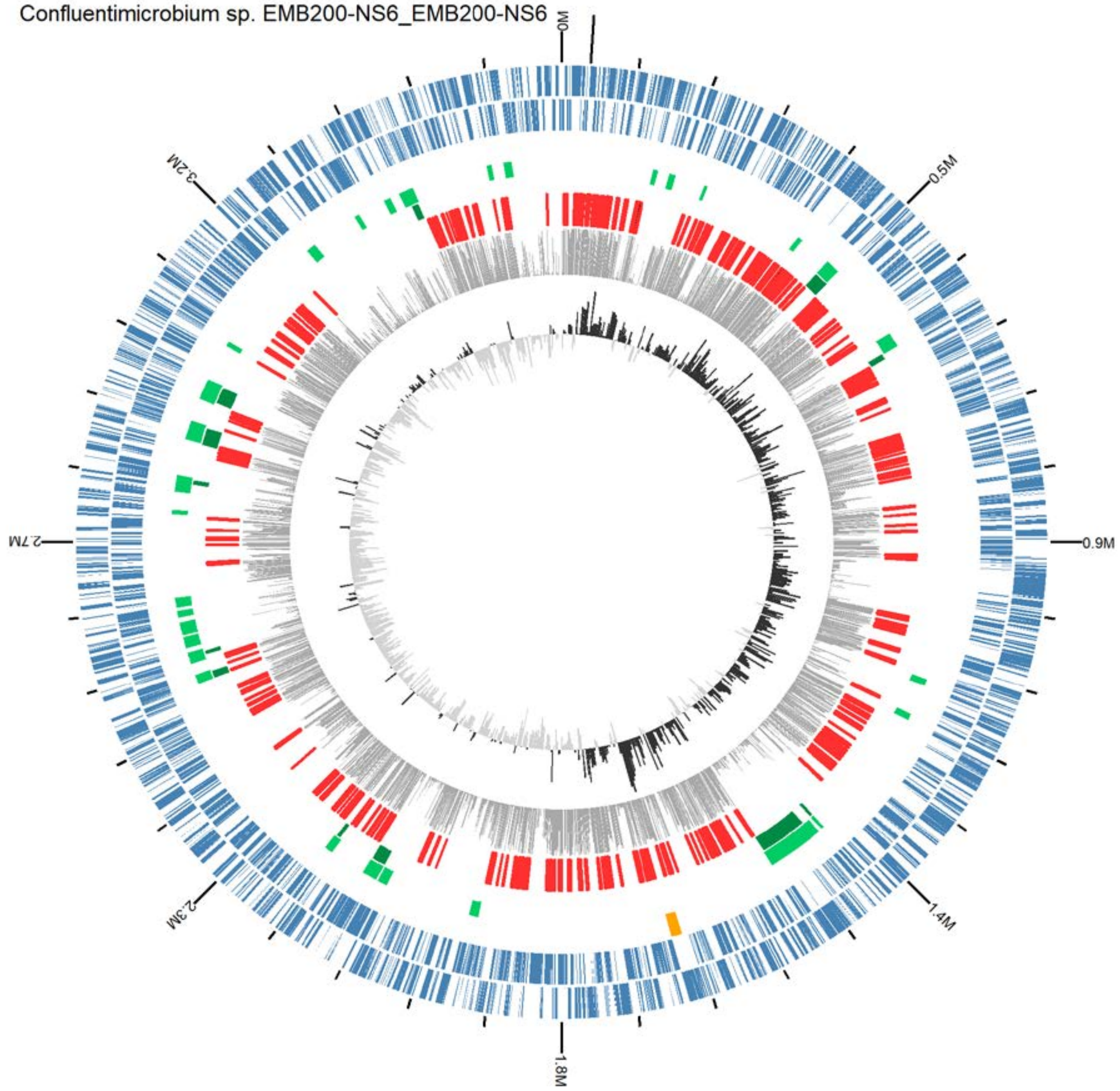

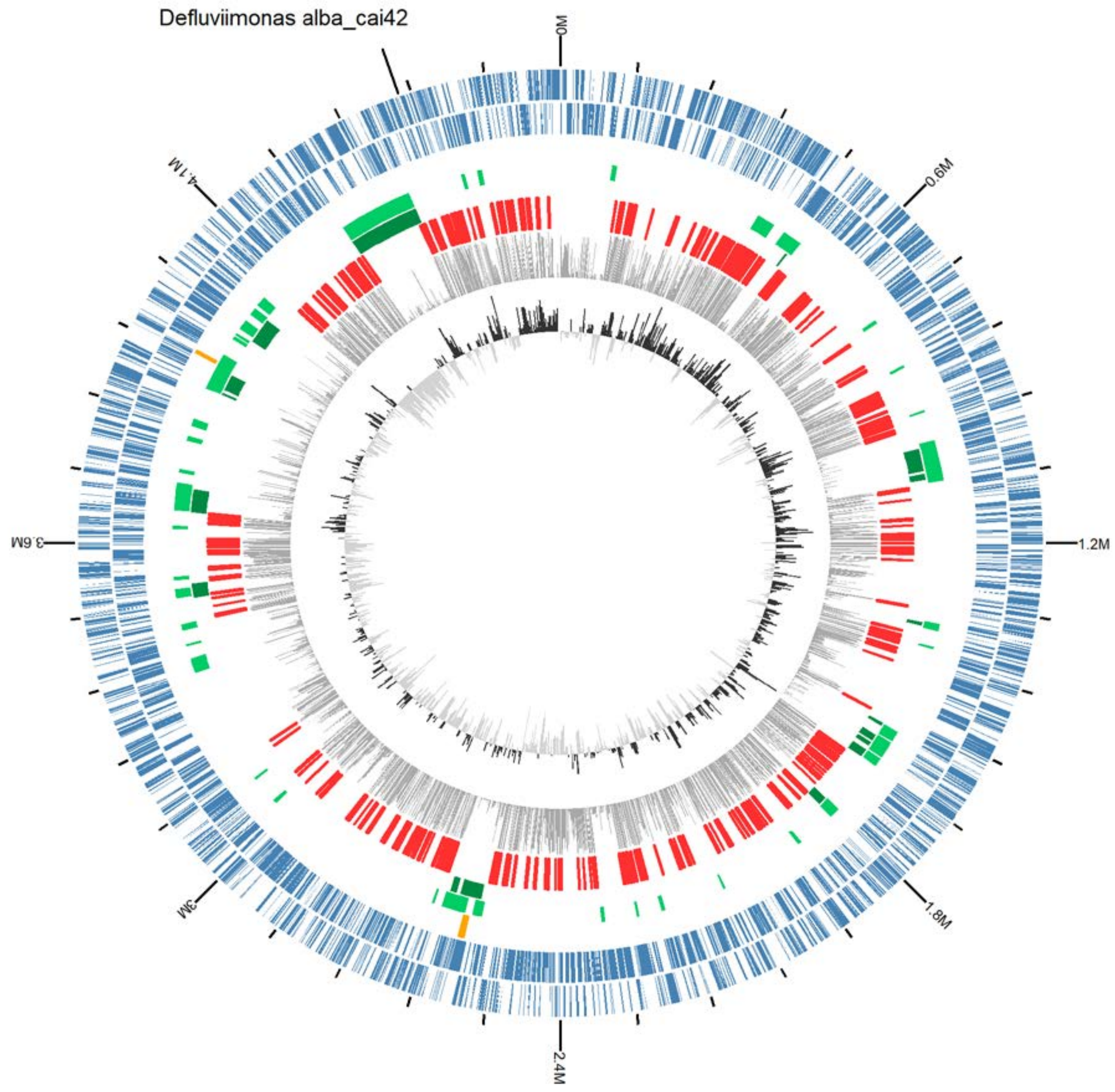

Defluviimonas alba_cai42
0M
0.6M
1.2M
1.8M
2.4M
3M
3.6M
4.1M

Dinoroseobacter shibae DFL 12 = DSM 16493_DFL 12

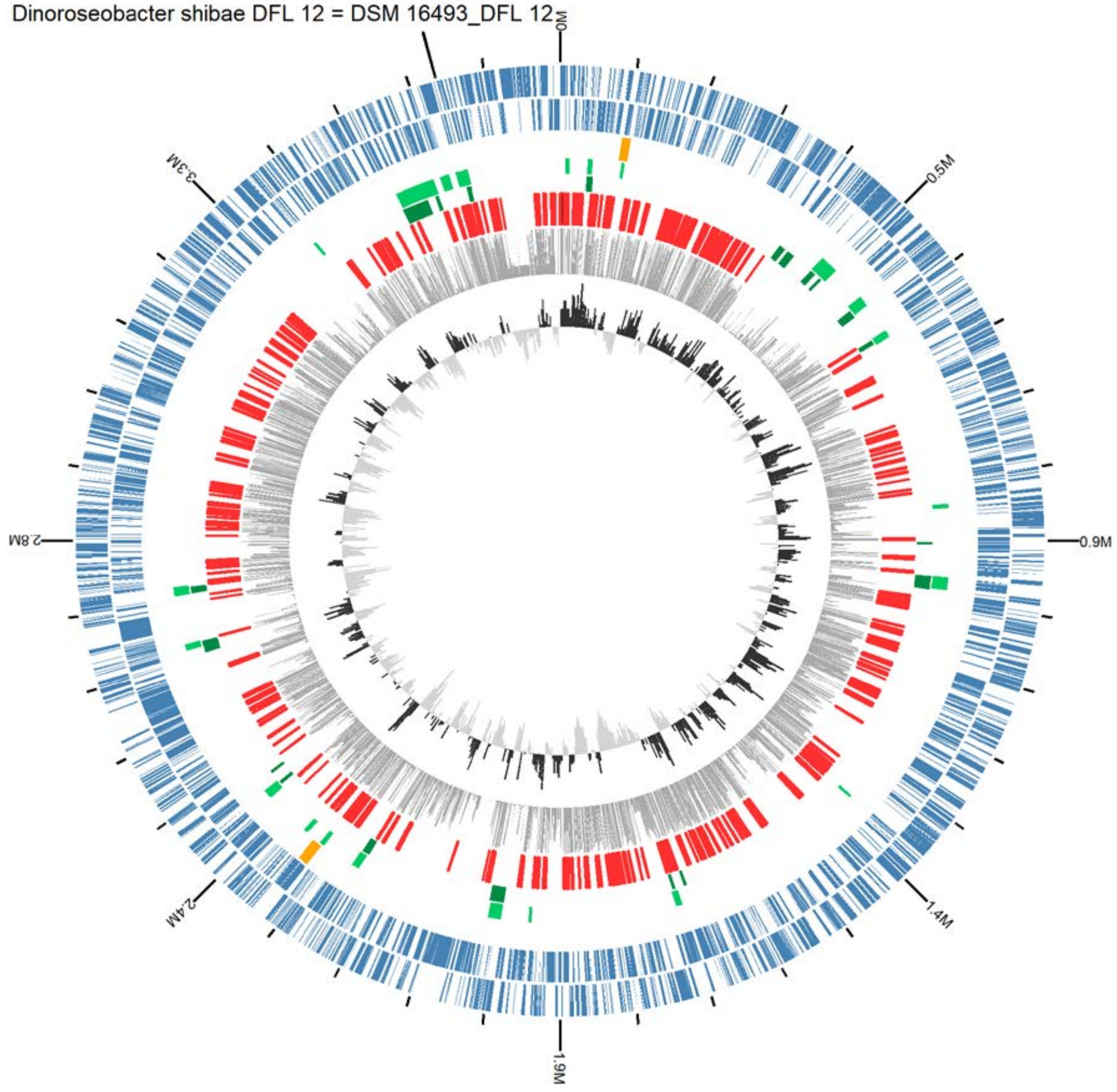

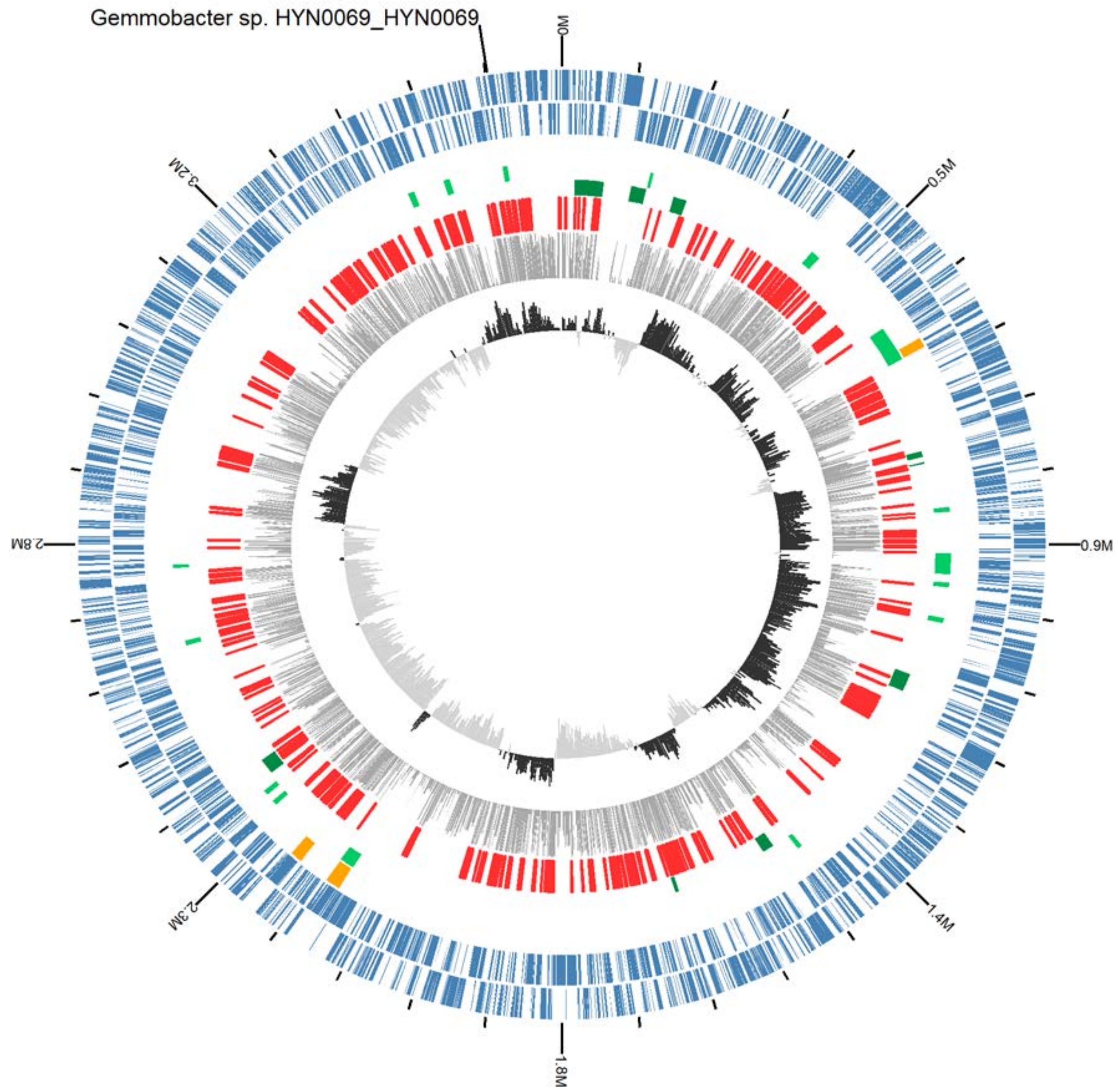
Gemmobacter sp. HYN0069_HYN0069
0M
0.5M
0.9M
1.4M
1.8M
2.3M
2.8M
3.2M

Halocynthiibacter arcticus_PAMC 20958

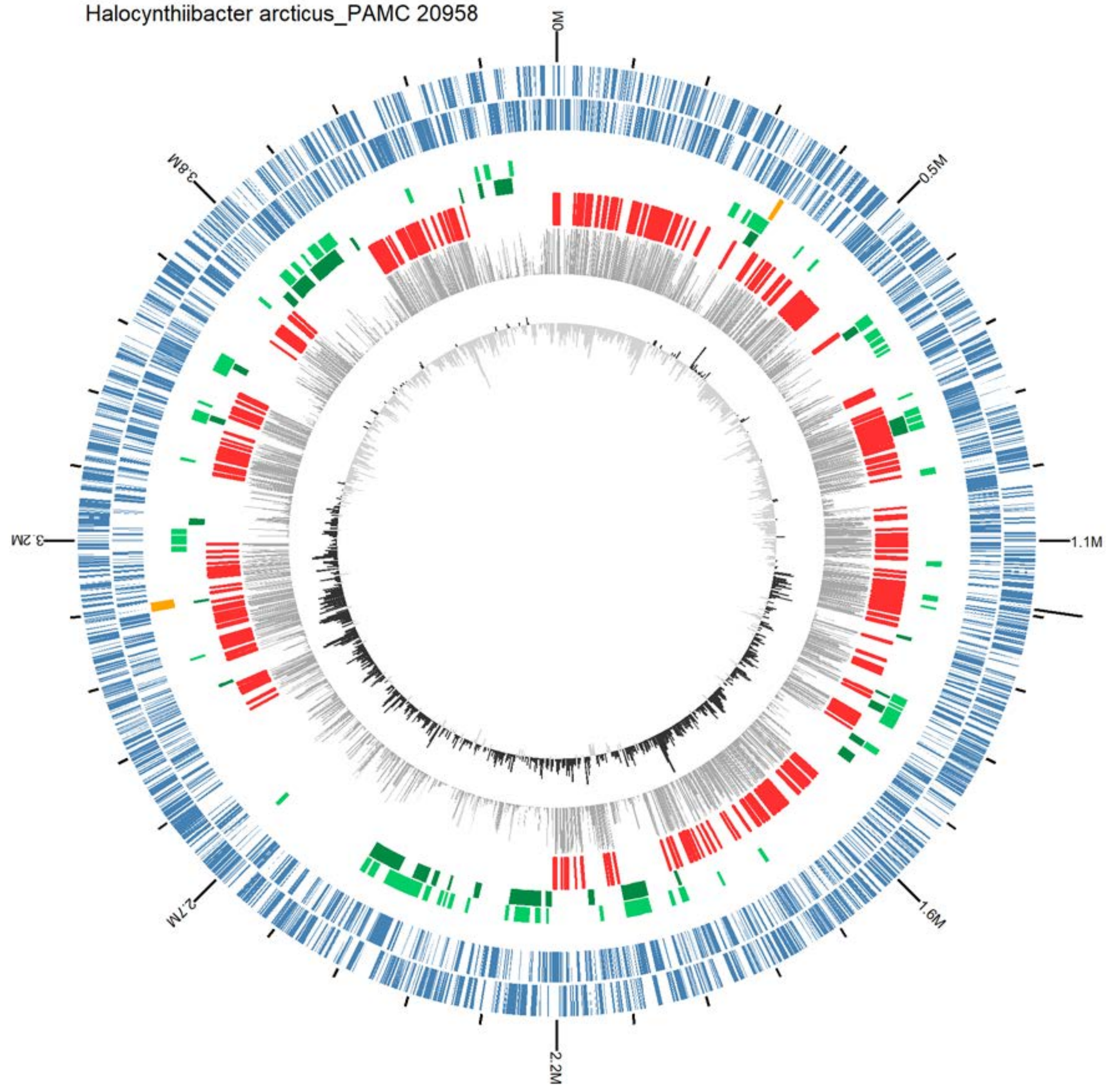

Jannaschia sp. CCS1_CCS1

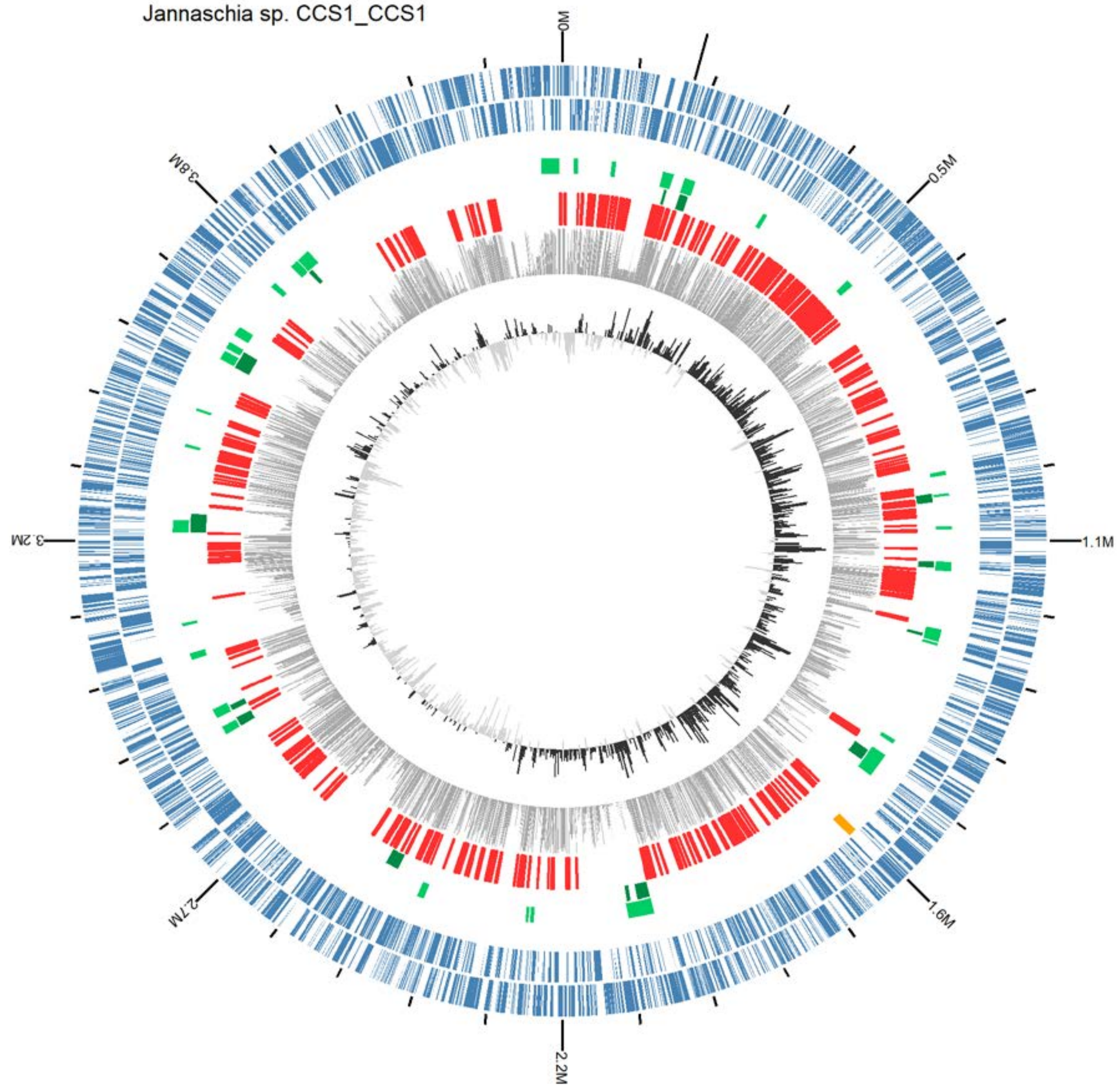

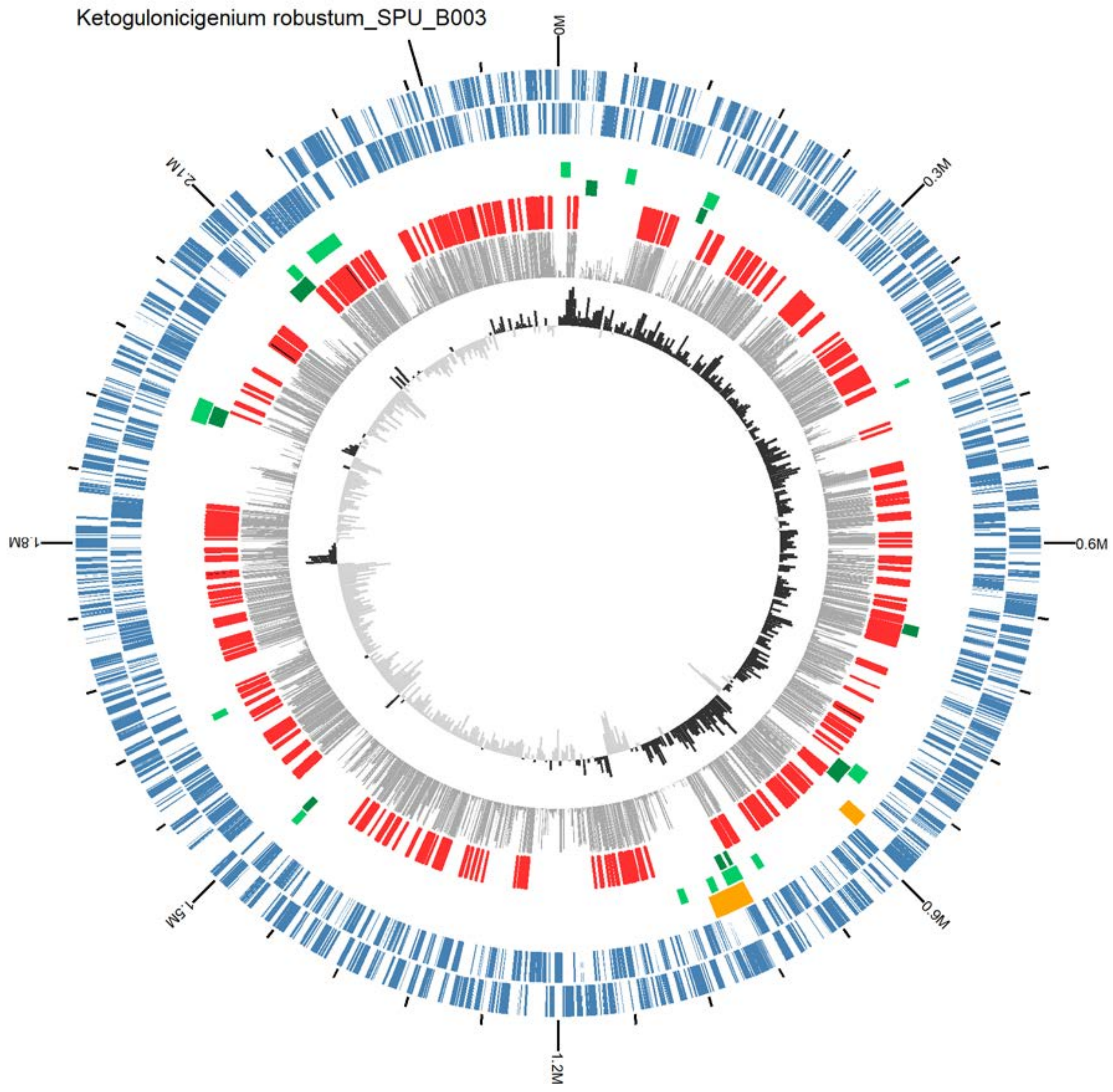

Ketogulonicigenium robustum_SPU_B003
0M
0.3M
0.6M
0.9M
1.2M
1.5M
1.8M
2.1M

Ketogulonicigenium vulgare WSH-001_WSH-001

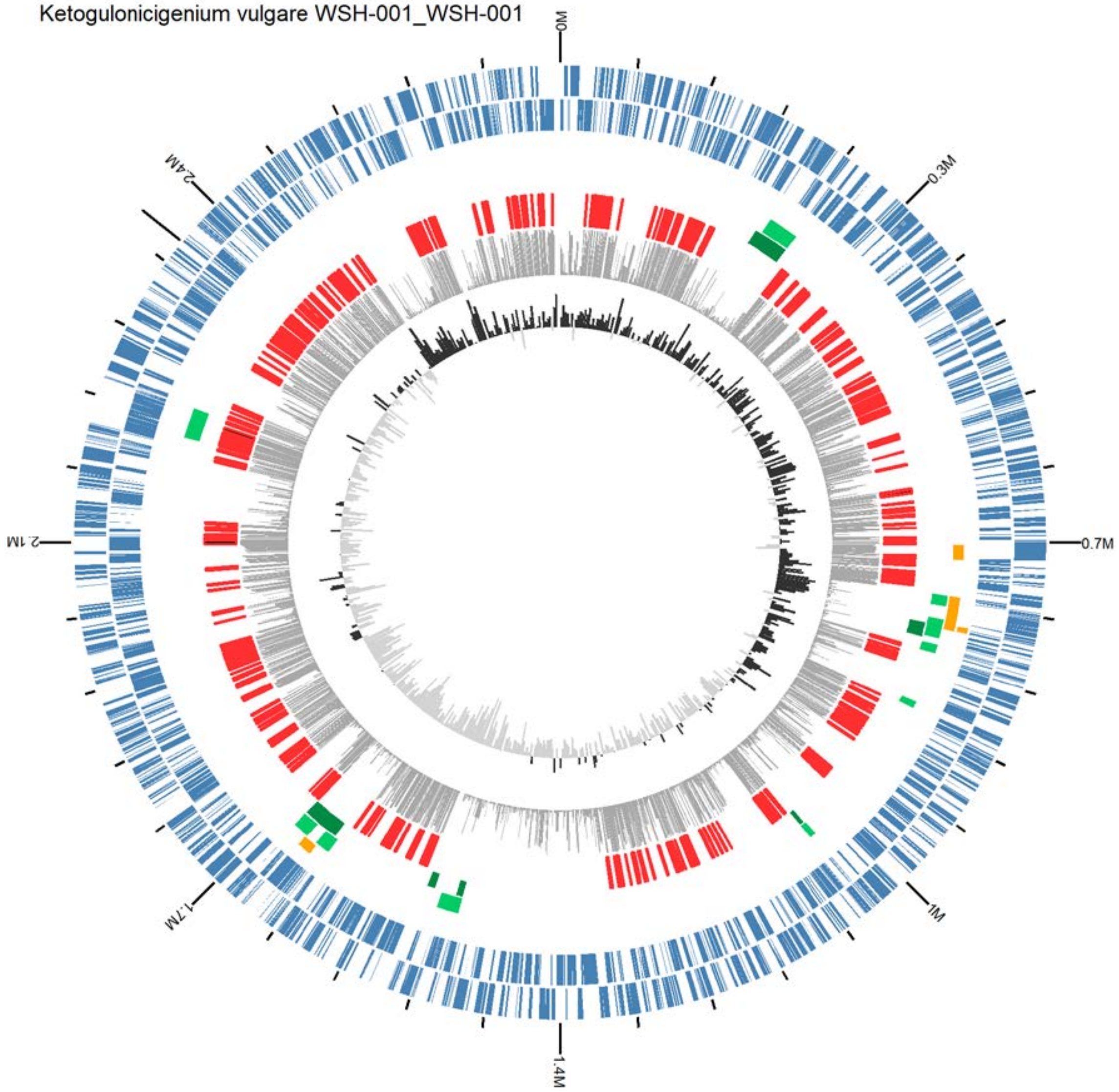

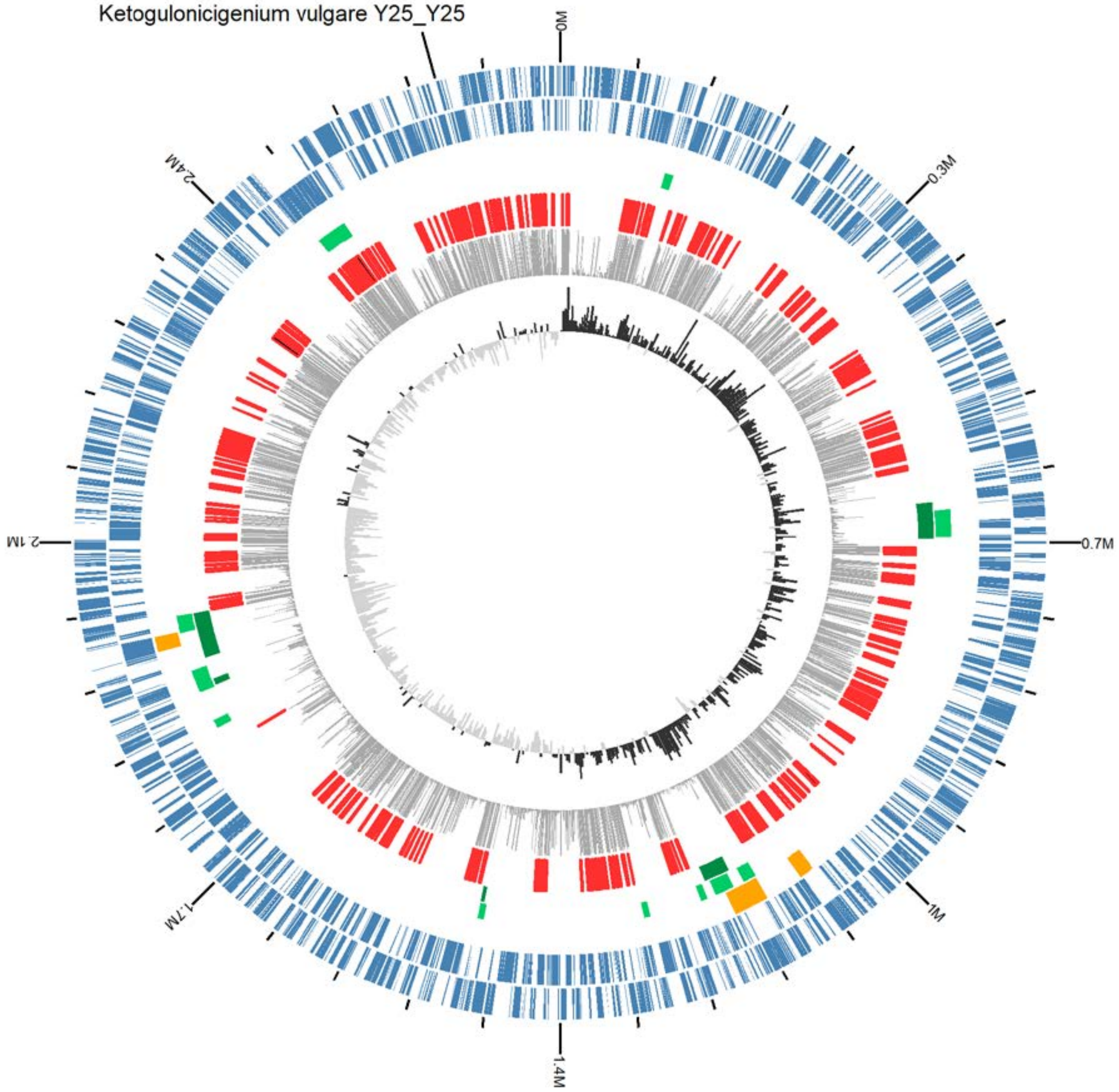

Ketogulonicigenium vulgare Y25_Y25
0M
0.3M
0.7M
1M
1.4M
1.7M
2.1M
2.4M

Ketogulonicigenium vulgare_Hbe602

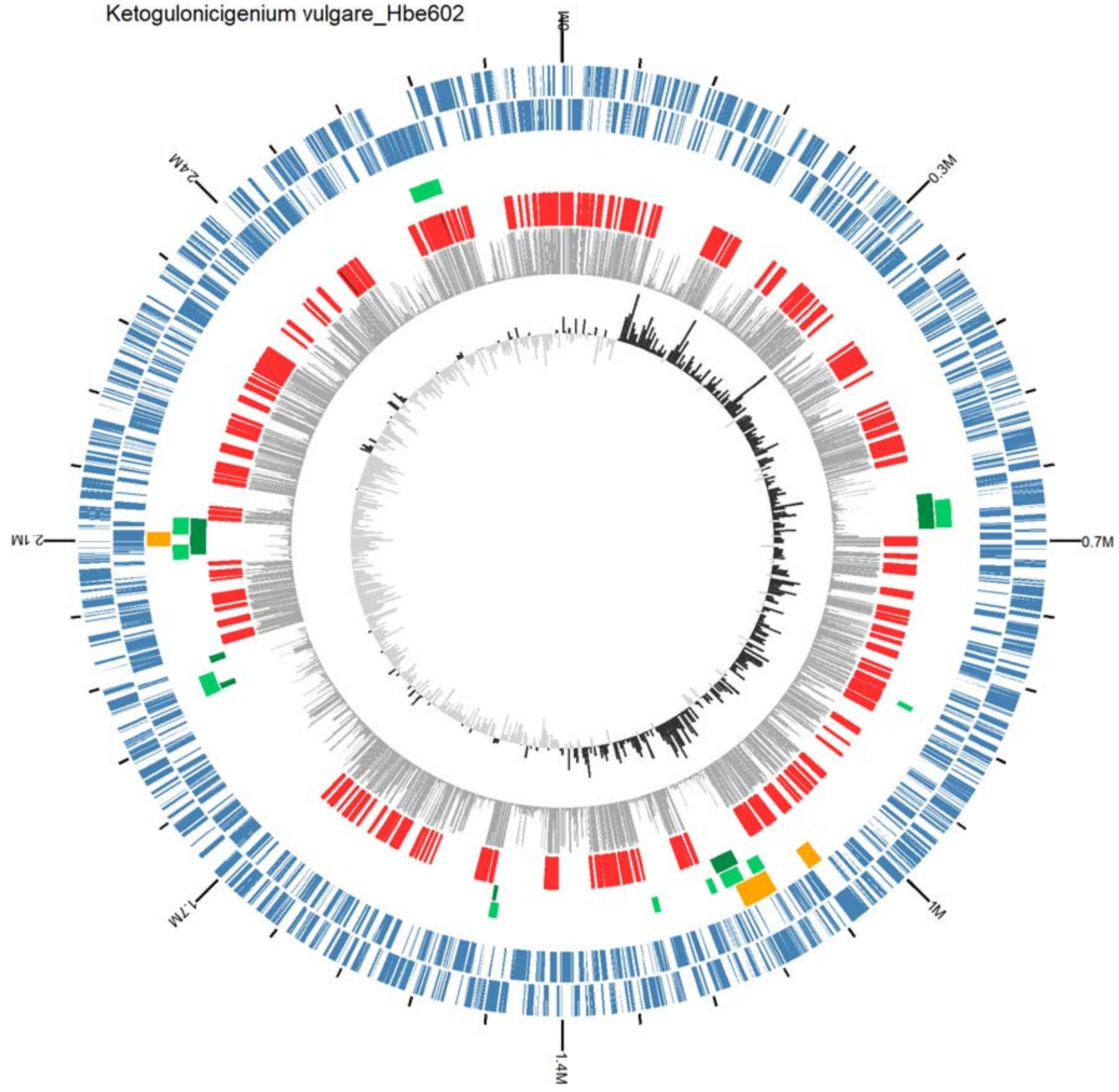

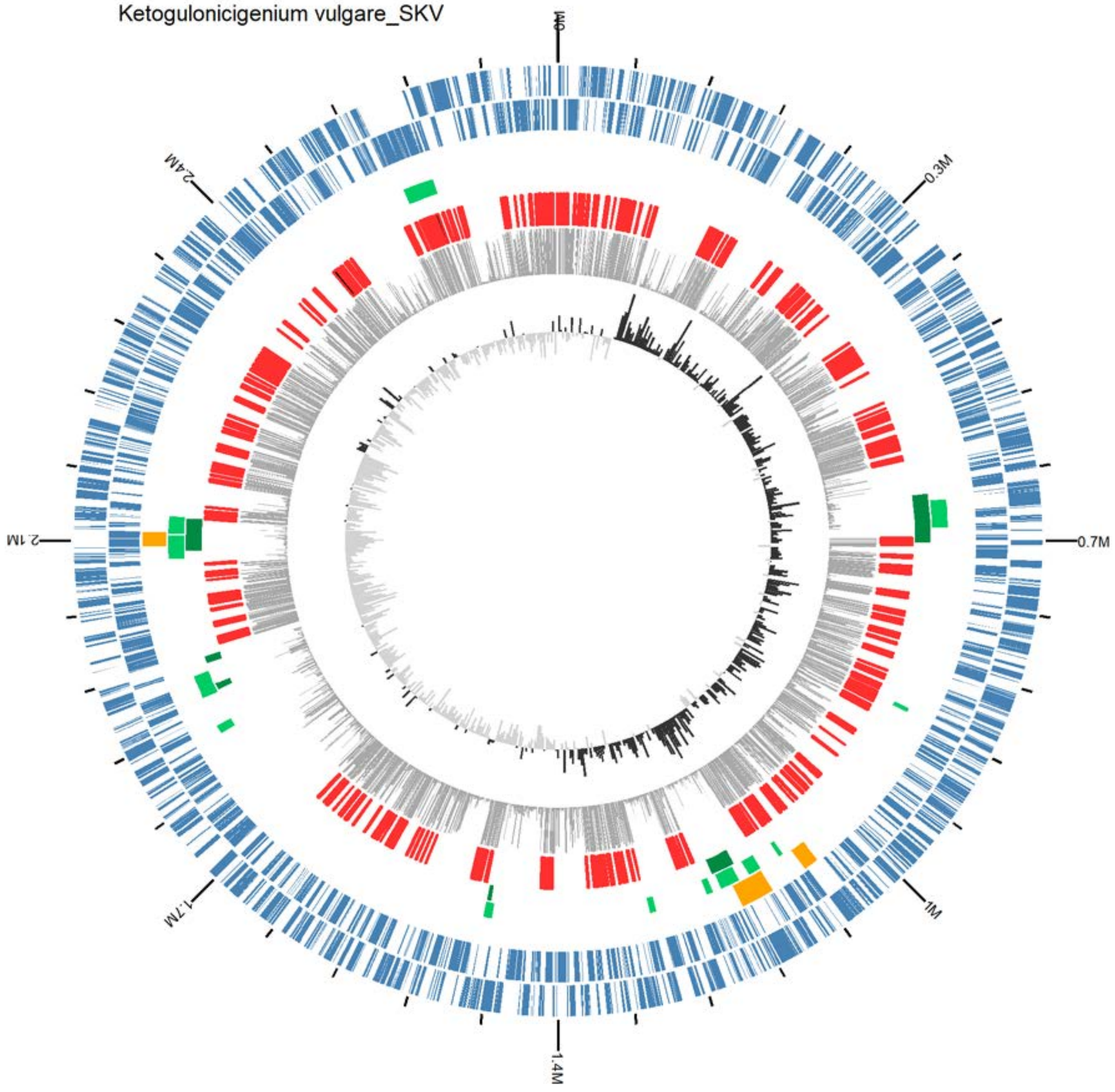

Ketogulonicigenium vulgare_SKV
0M
0.3M
0.7M
1M
1.4M
1.7M
2.1M
2.4M

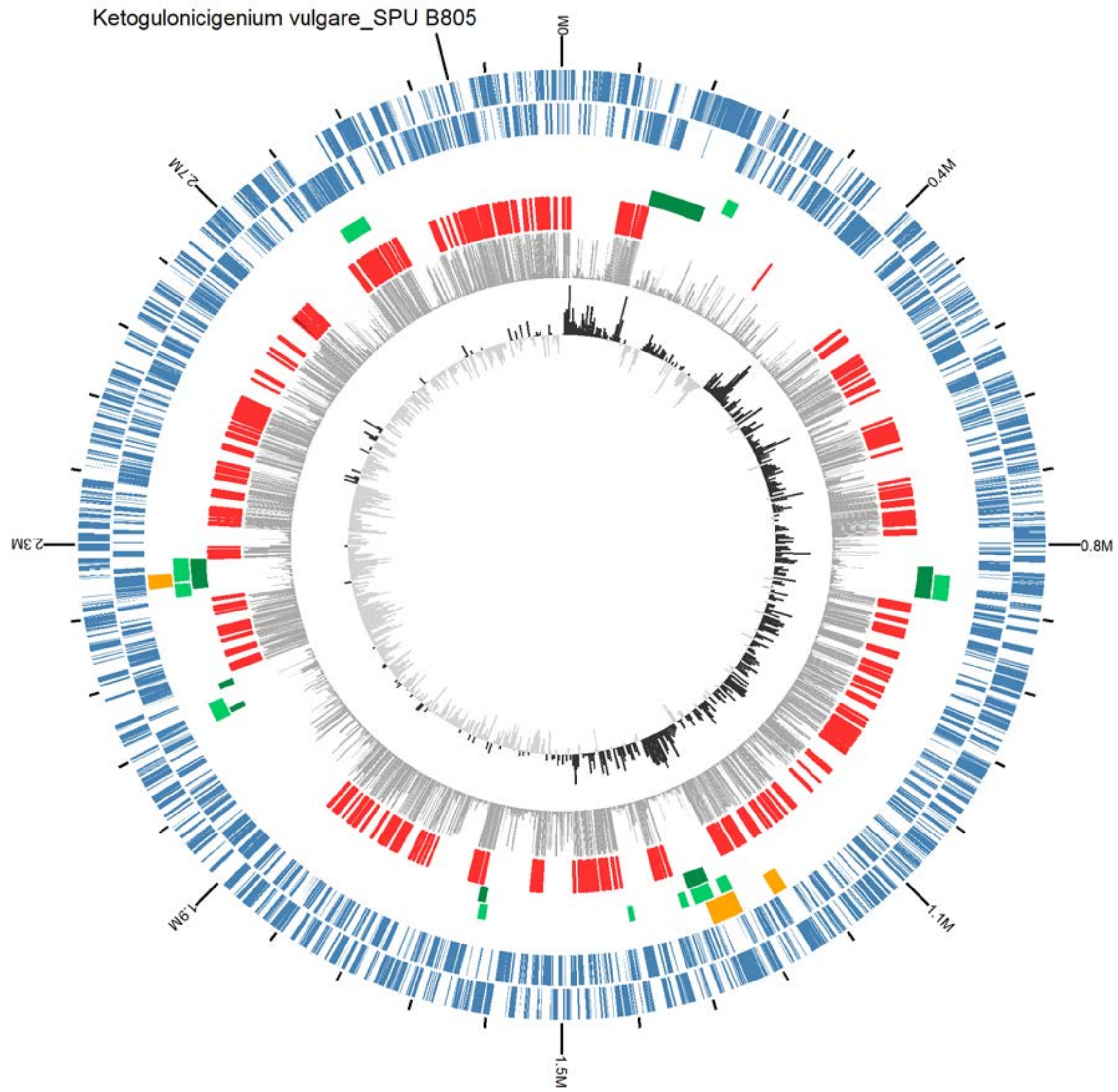

Ketogulonicigenium vulgare_SPU B805
0M
0.4M
0.8M
1.1M
1.5M
1.9M
2.3M
2.7M

eisingera methylohalidivorans DSM 14336_DSM 14336; MB2

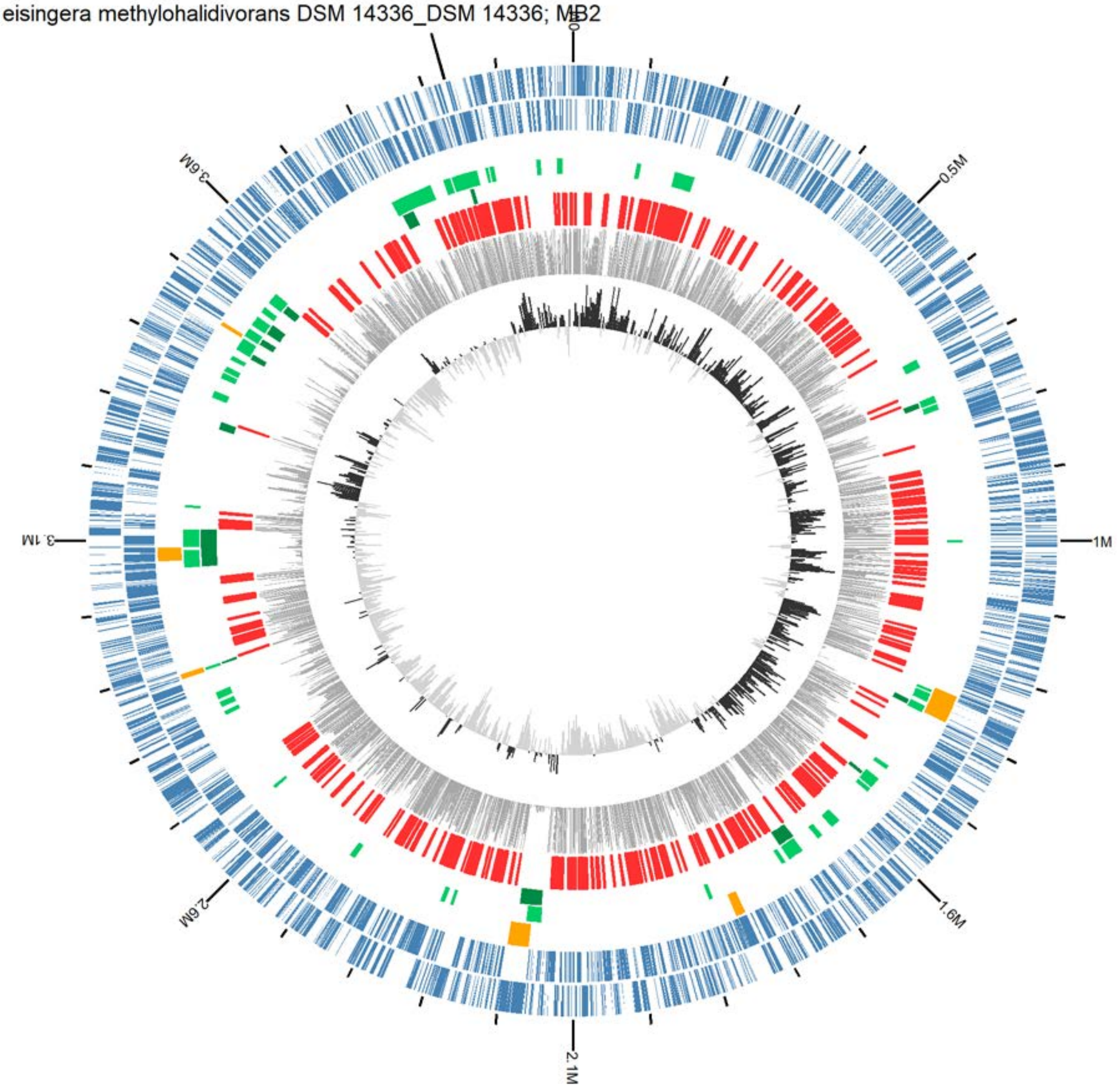

Loktanella vestfoldensis_SMR4r

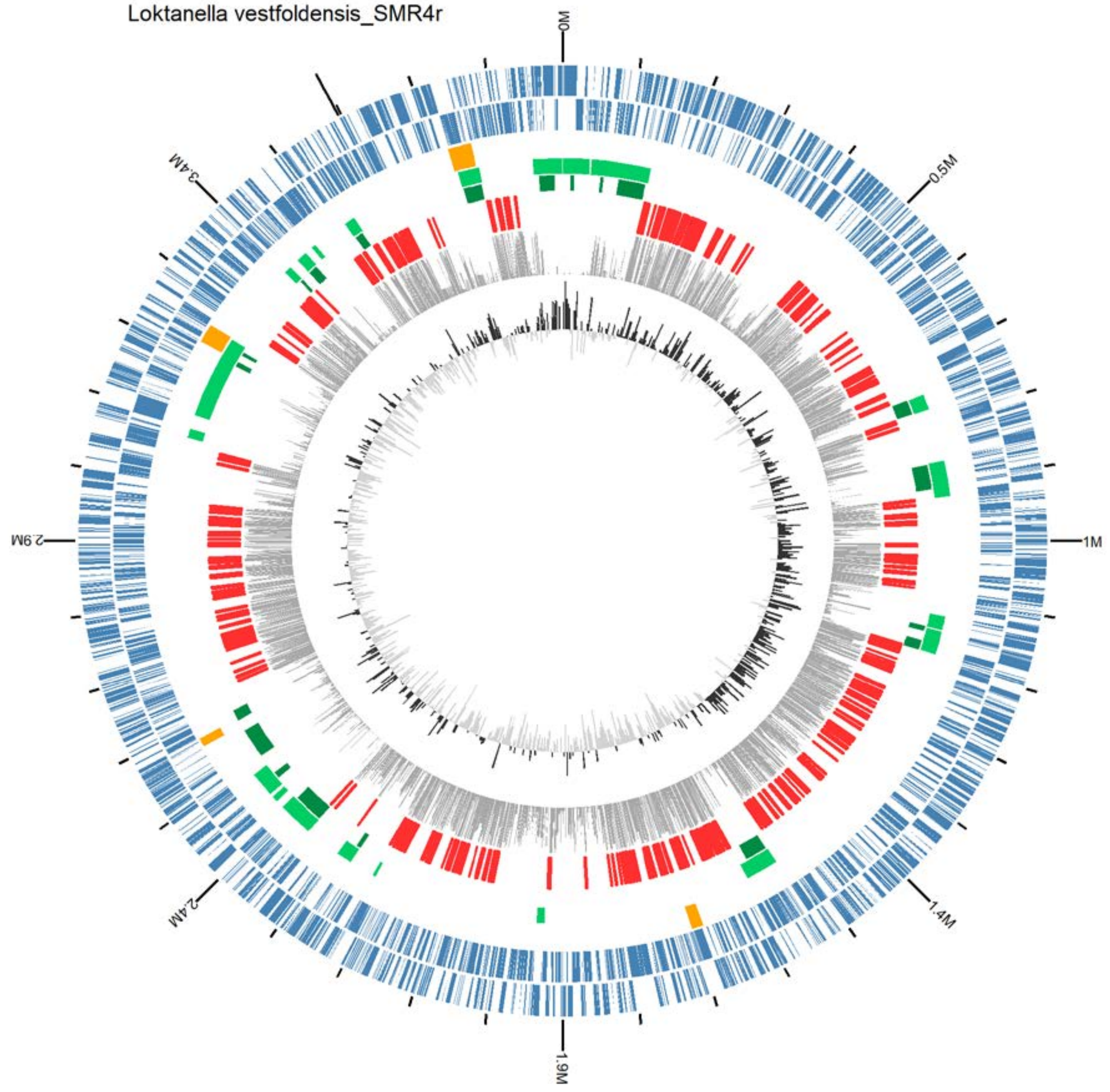

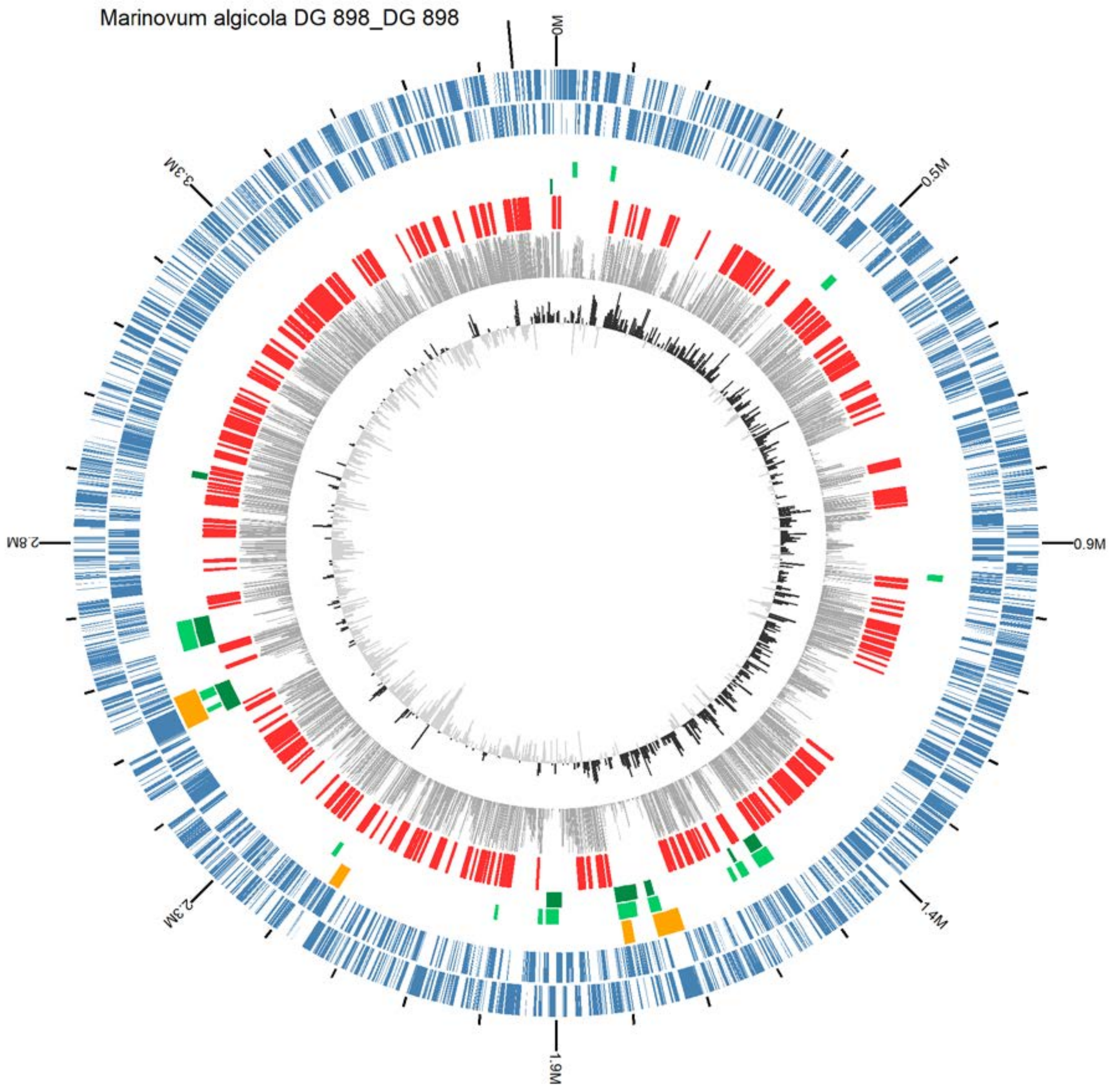
Marinovum algicola DG 898_DG 898
0M
0.5M
0.9M
1.4M
1.9M
2.3M
2.8M
3.3M

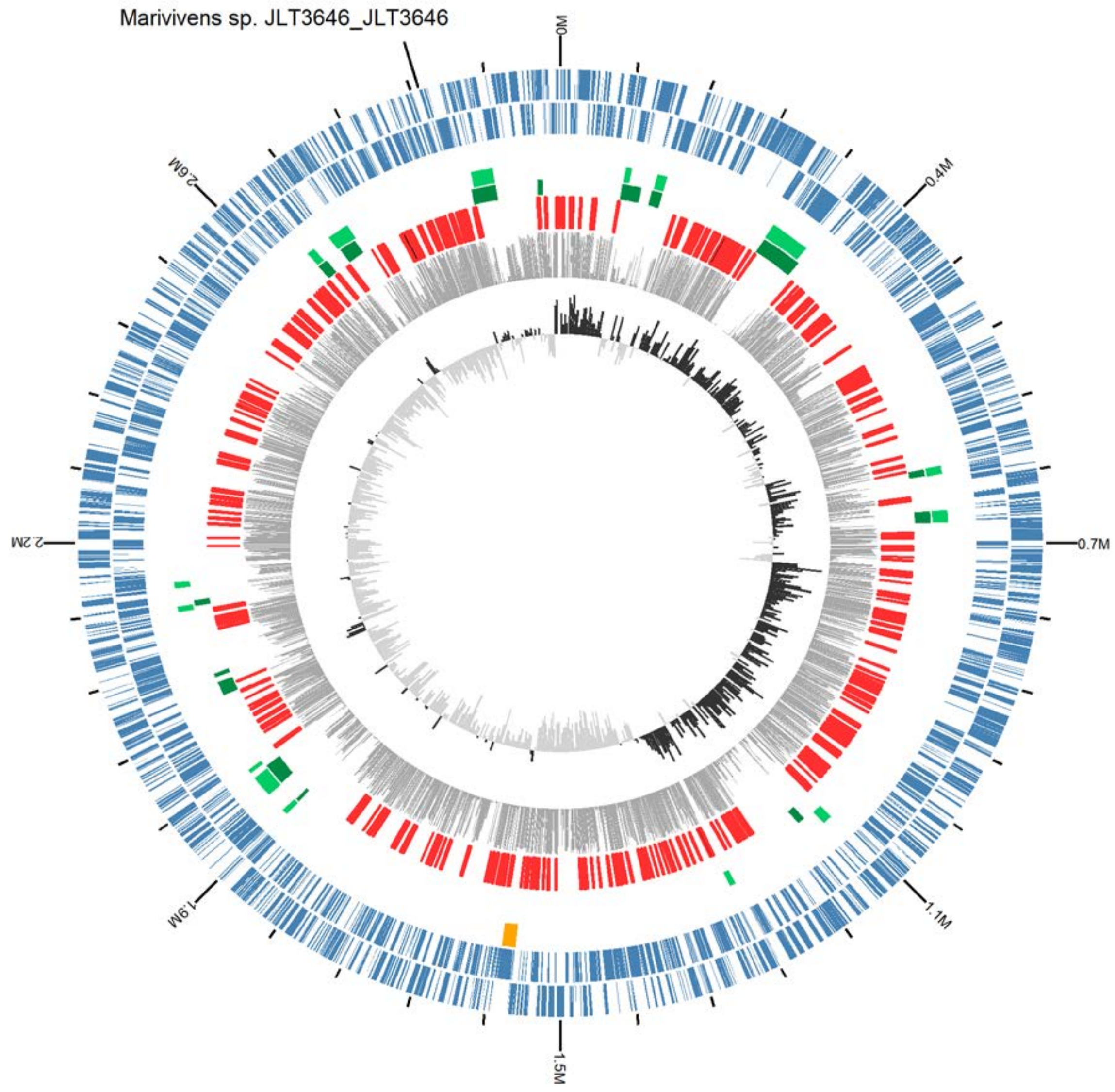

Marivivens sp. JLT3646_JLT3646
0M
0.4M
0.7M
1.1M
1.5M
1.9M
2.2M
2.6M

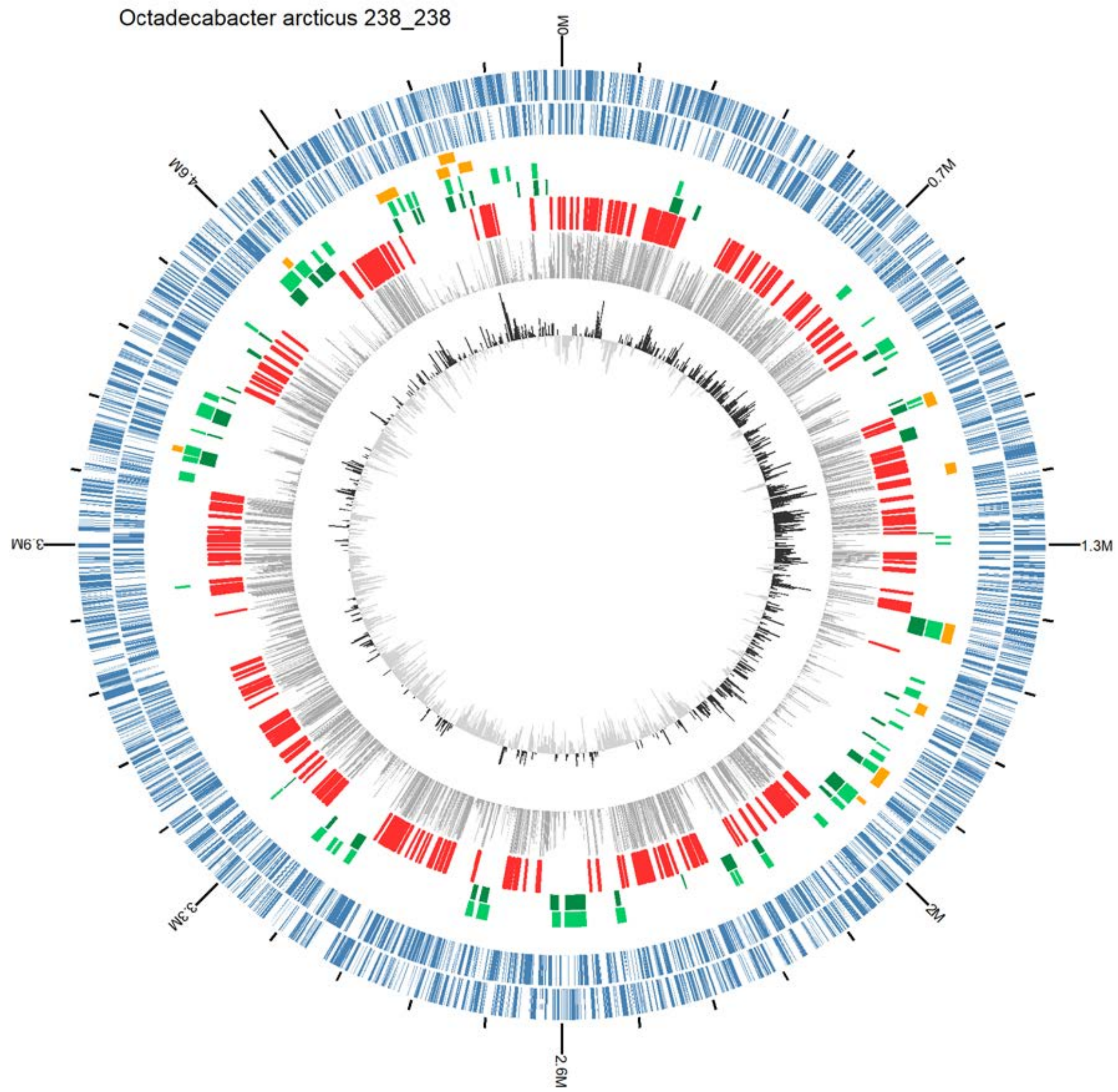
Octadecabacter arcticus 238_238
0M
0.7M
1.3M
2M
2.6M
3.3M
3.9M
4.6M

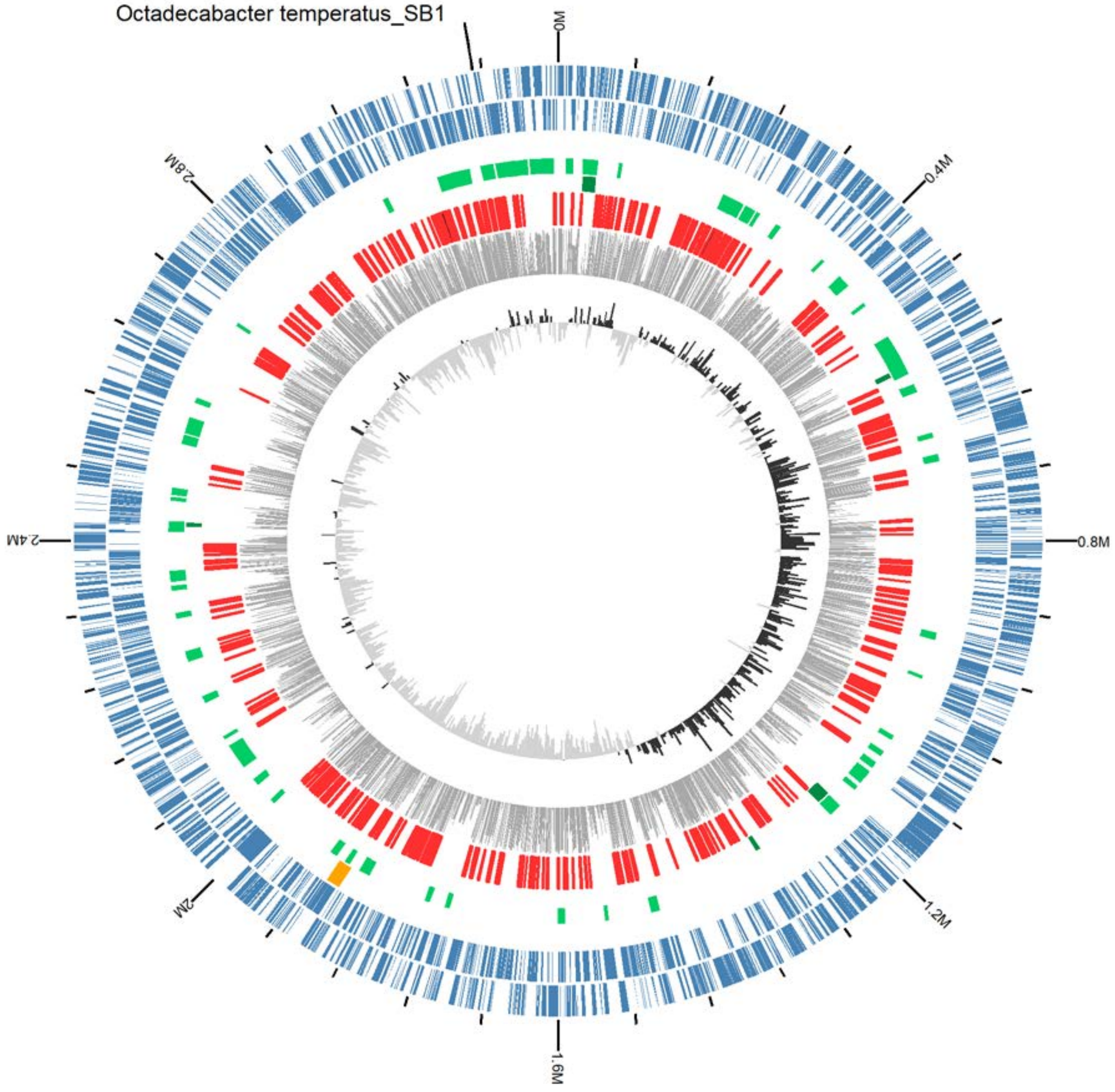
Octadecabacter temperatus_SB1
0M
0.4M
0.8M
1.2M
1.6M
2M
2.4M
2.8M

Pannonibacter phragmitetus_31801

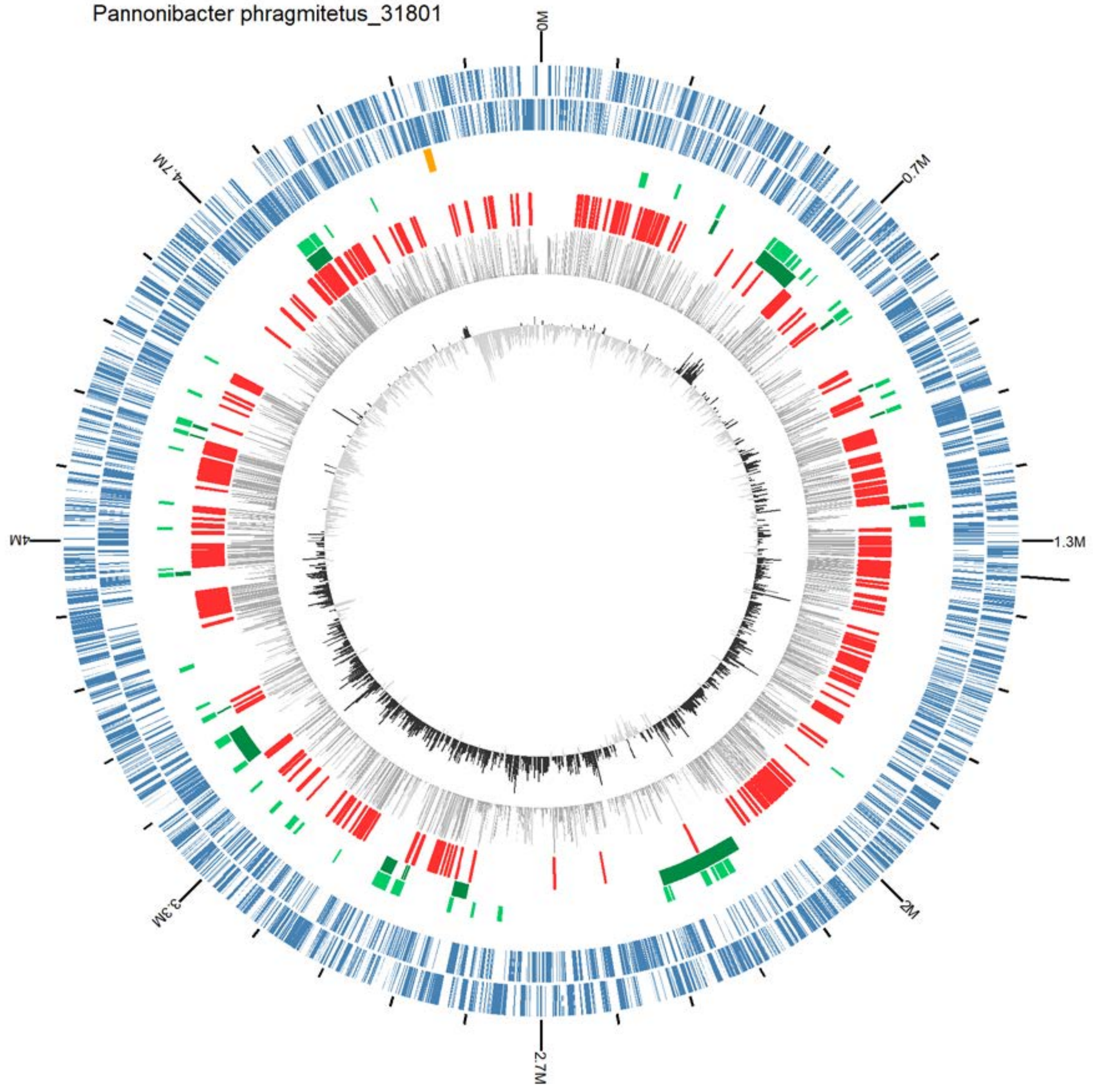

Paracoccus aminovorans_

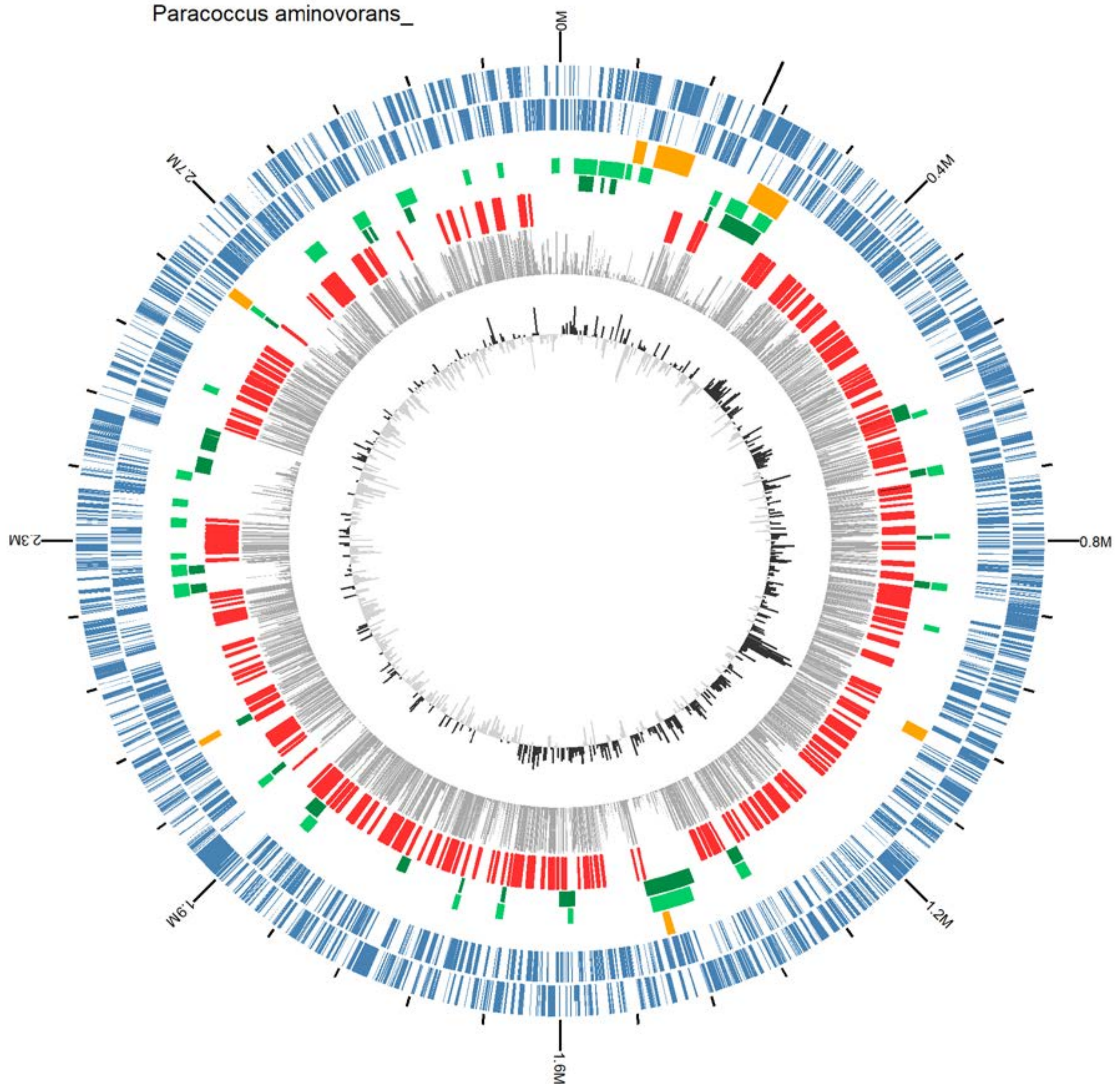

Paracoccus denitrificans PD1222_PD1222

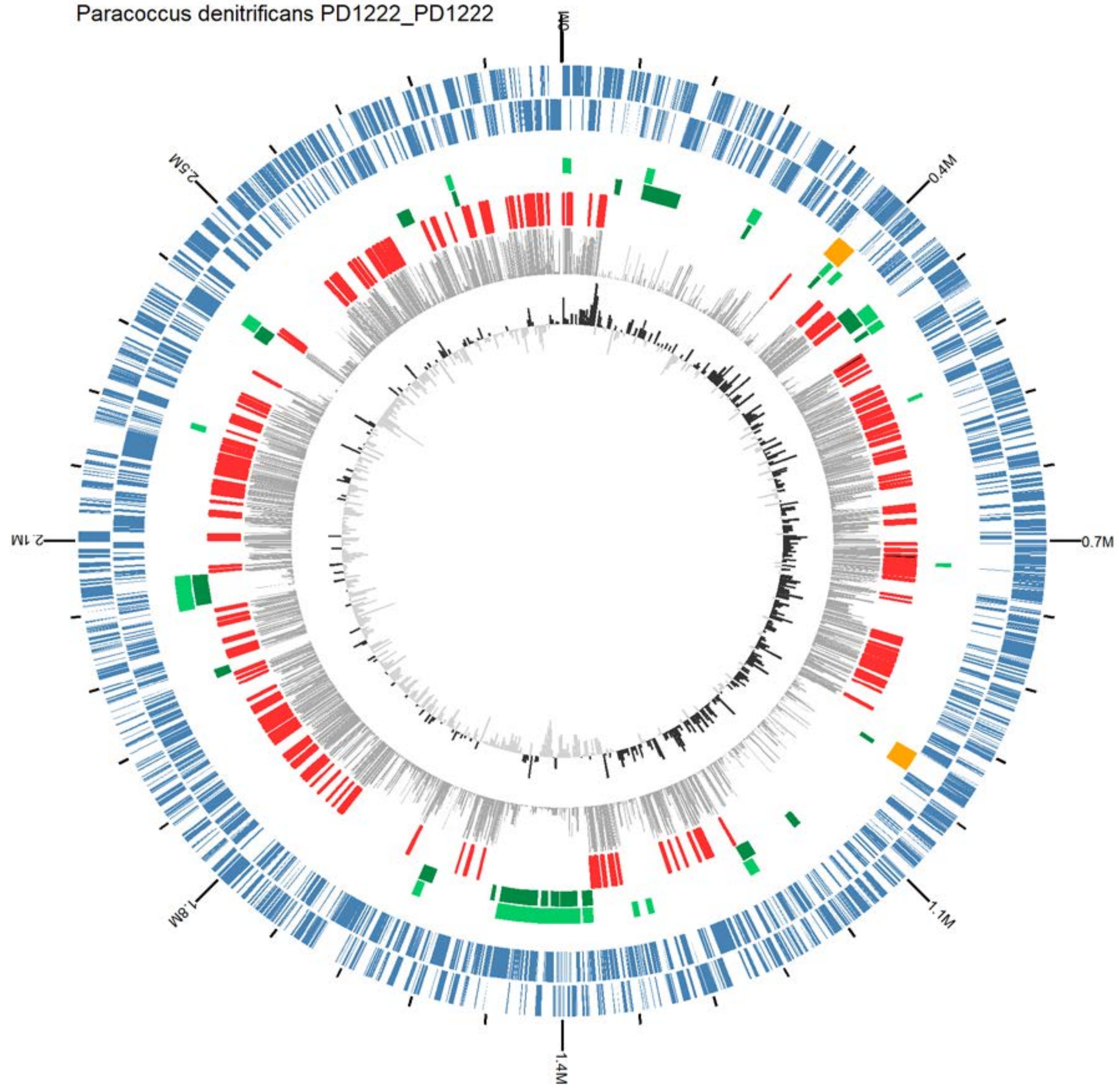

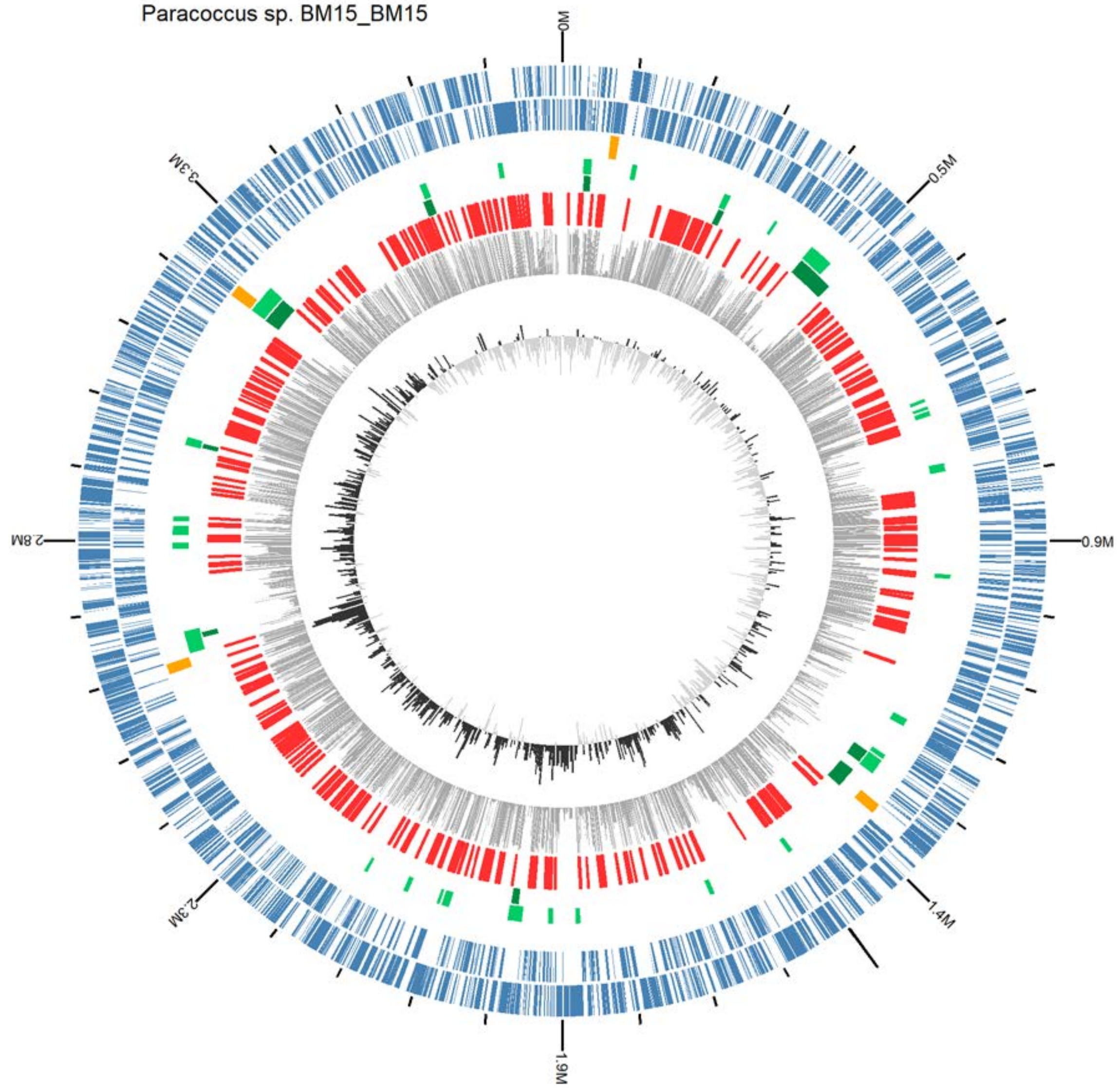
Paracoccus sp. BM15_BM15
0M
0.5M
0.9M
1.4M
1.9M
2.3M
2.8M
3.3M

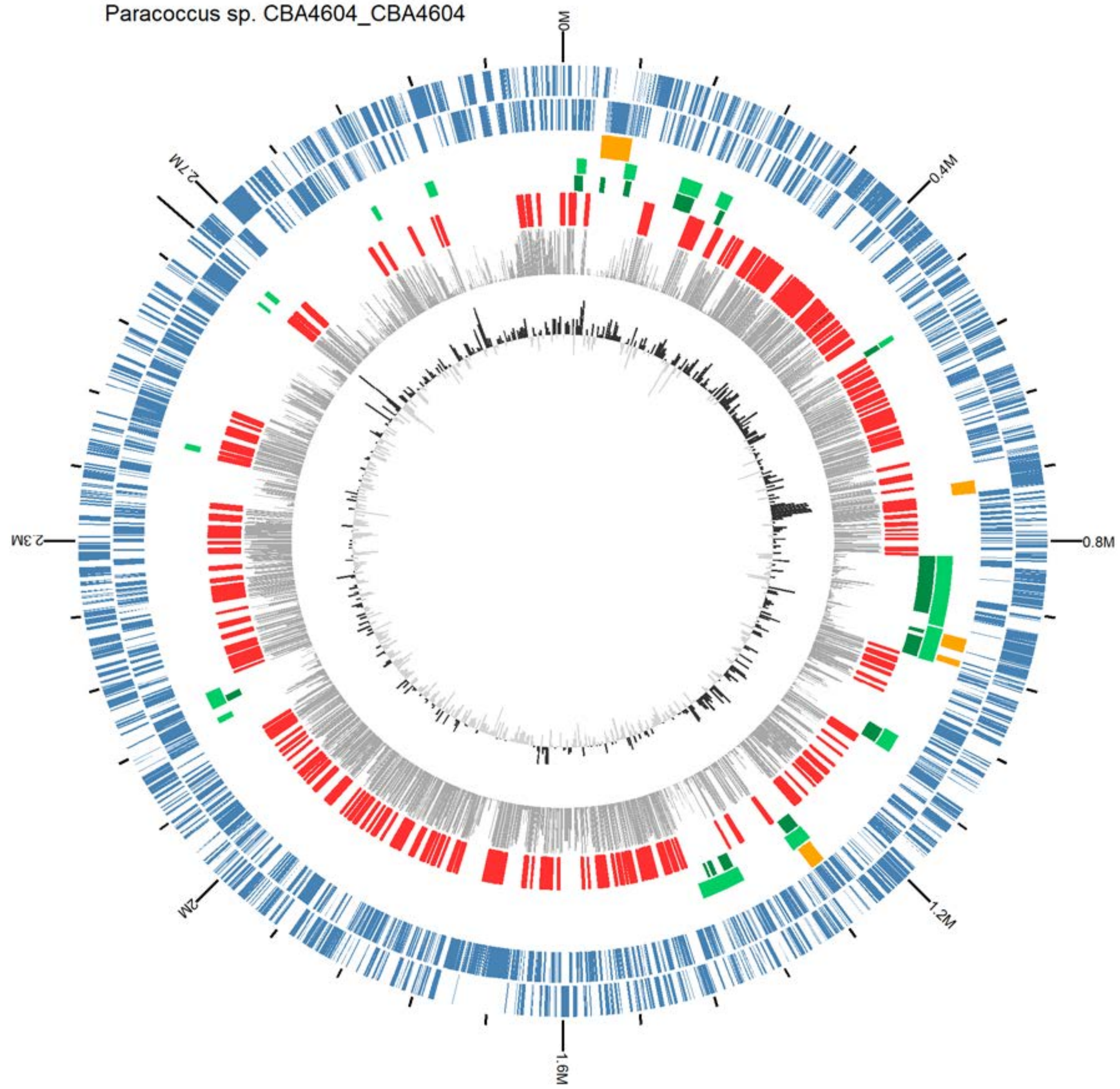
Paracoccus sp. CBA4604_CBA4604
0M
0.4M
0.8M
1.2M
1.6M
2M
2.3M
2.7M

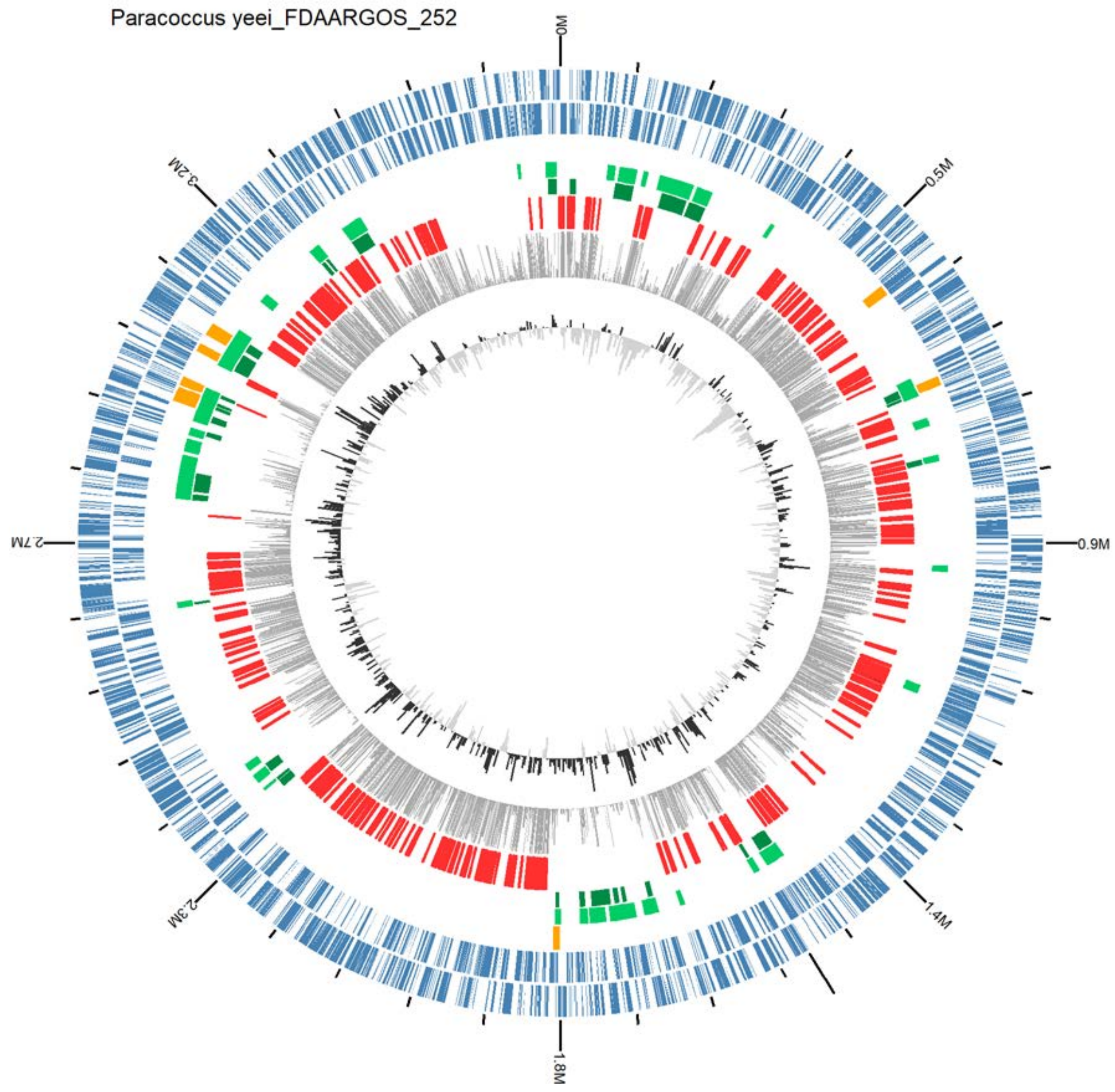
Paracoccus yeei_FDAARGOS_252
0M
0.5M
0.9M
1.4M
1.8M
2.3M
2.7M
3.2M

Paracoccus yeei_TT13

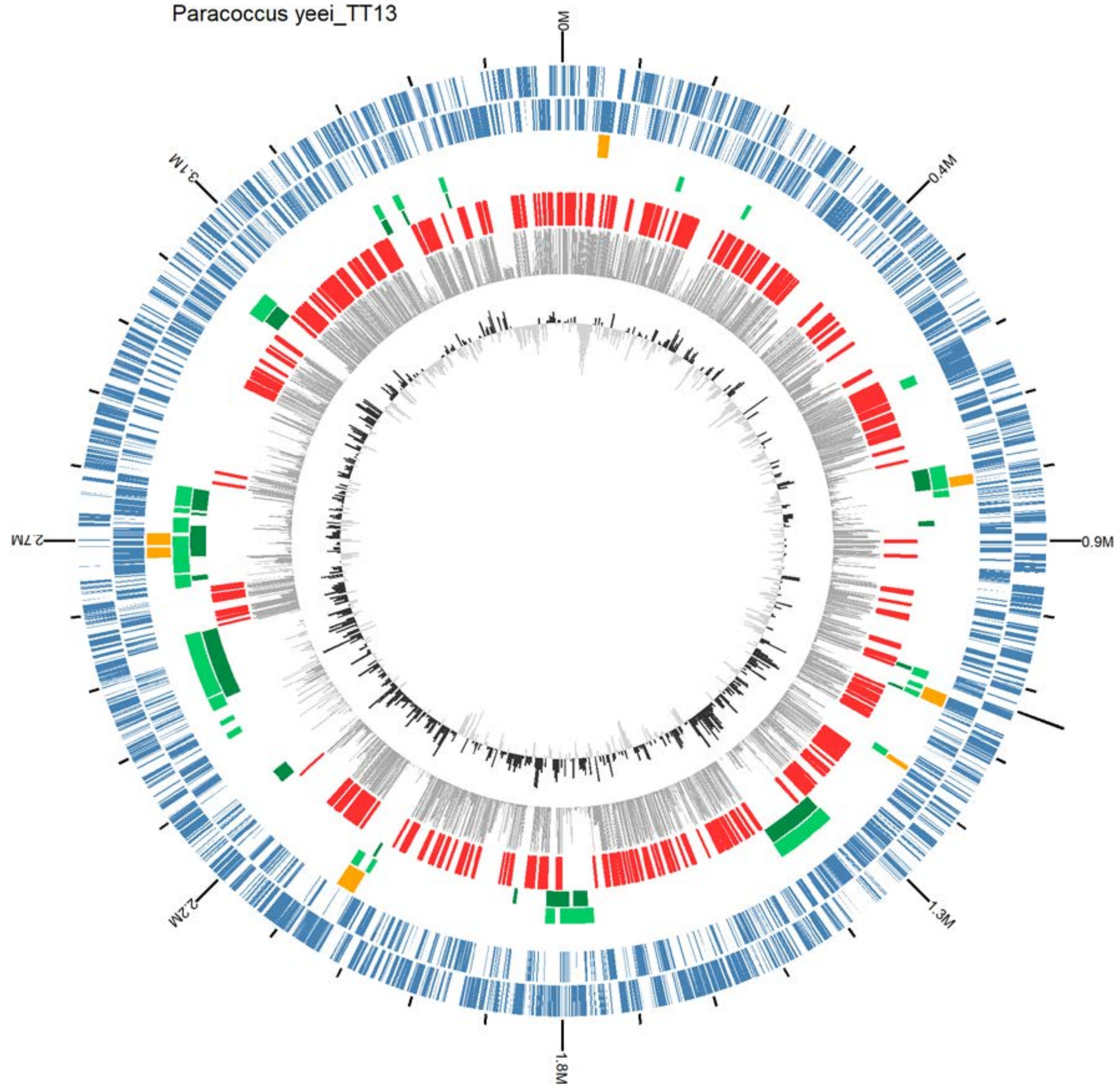

Paracoccus zhejiangensis_J6

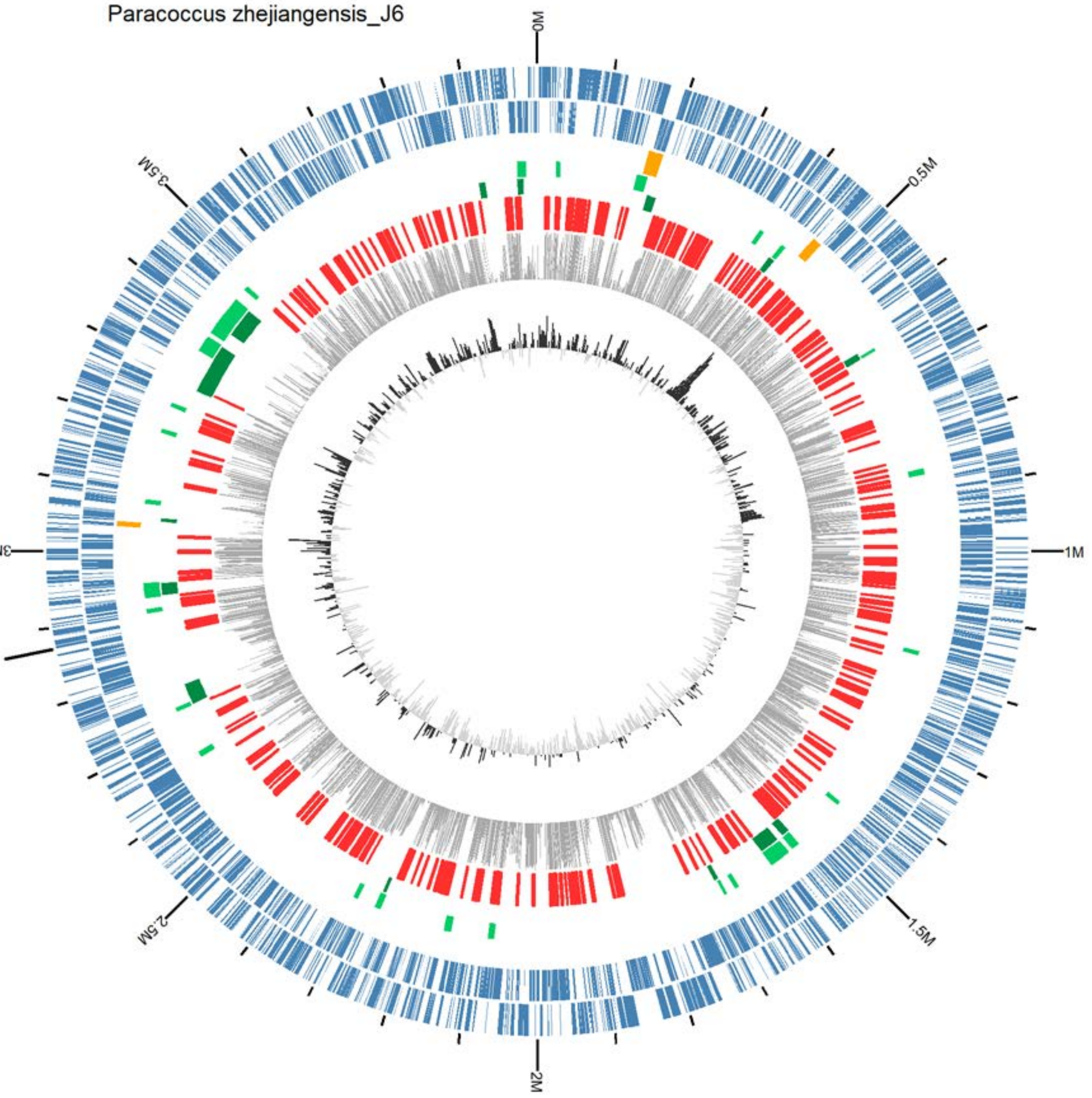

Pelagibaca abyssi_JLT2014

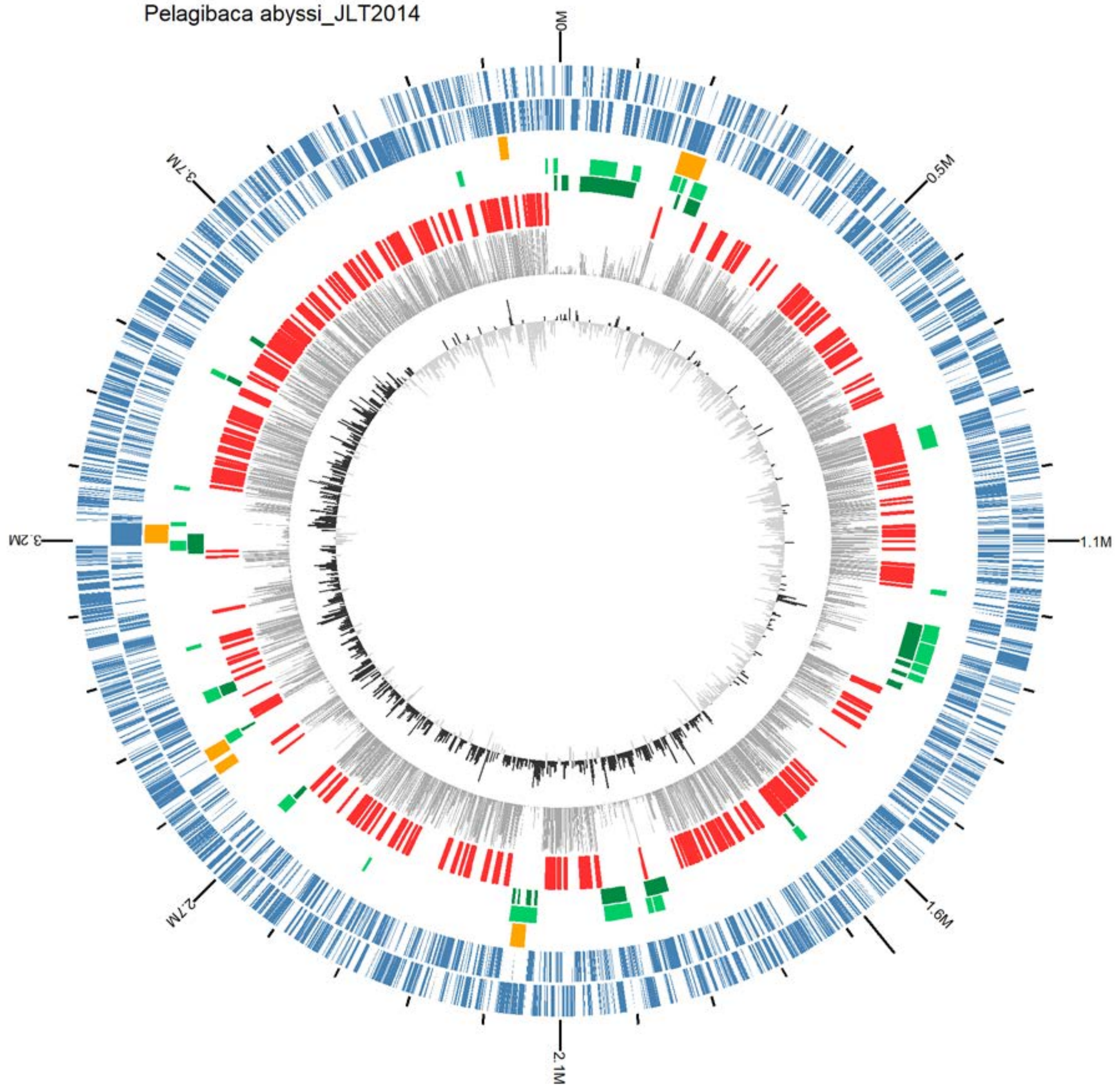

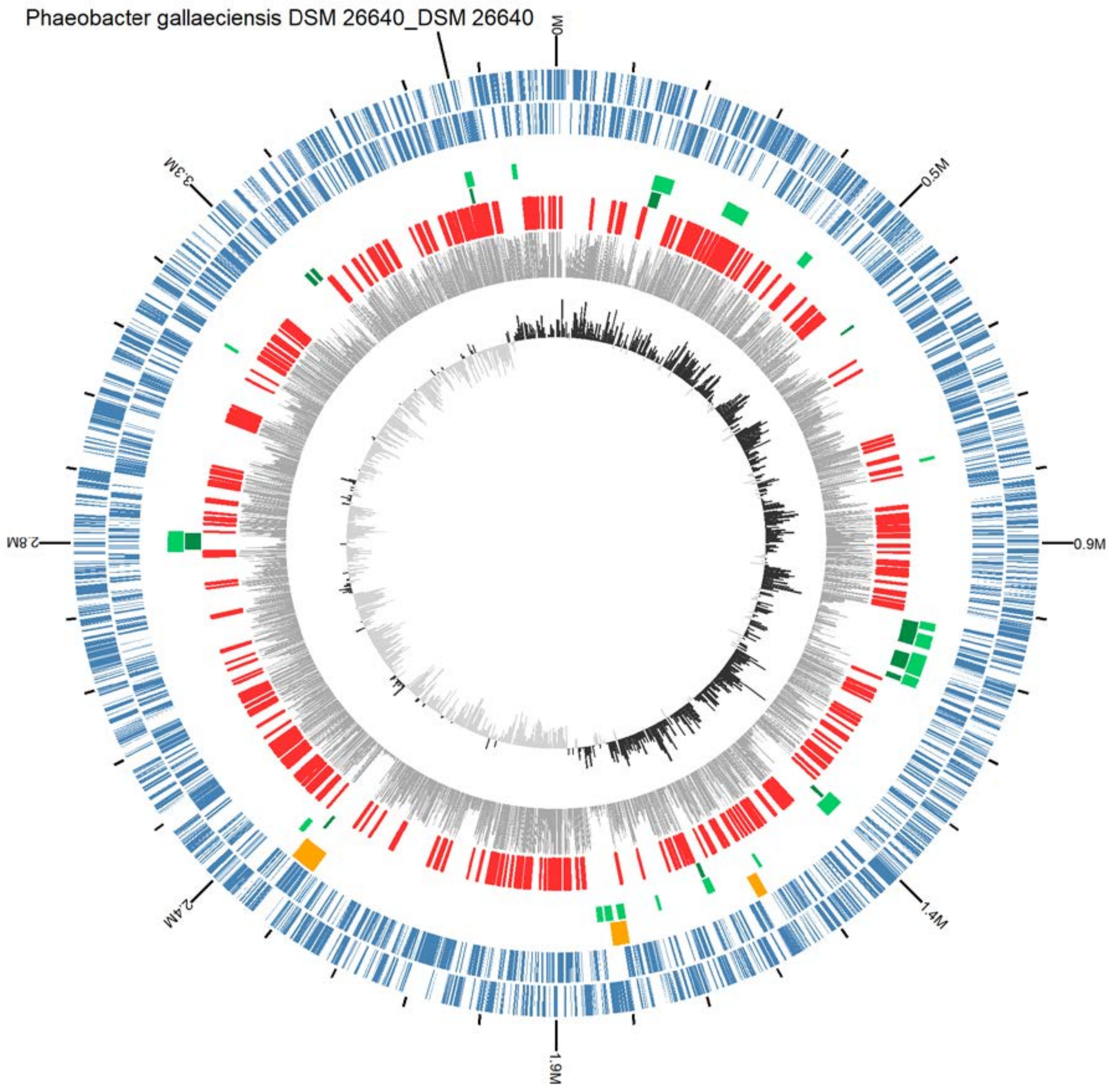
Phaeobacter gallaeciensis DSM 26640_DSM 26640
0M
0.5M
0.9M
1.4M
1.9M
2.4M
2.8M
3.3M

Phaeobacter gallaeciensis_JL2886

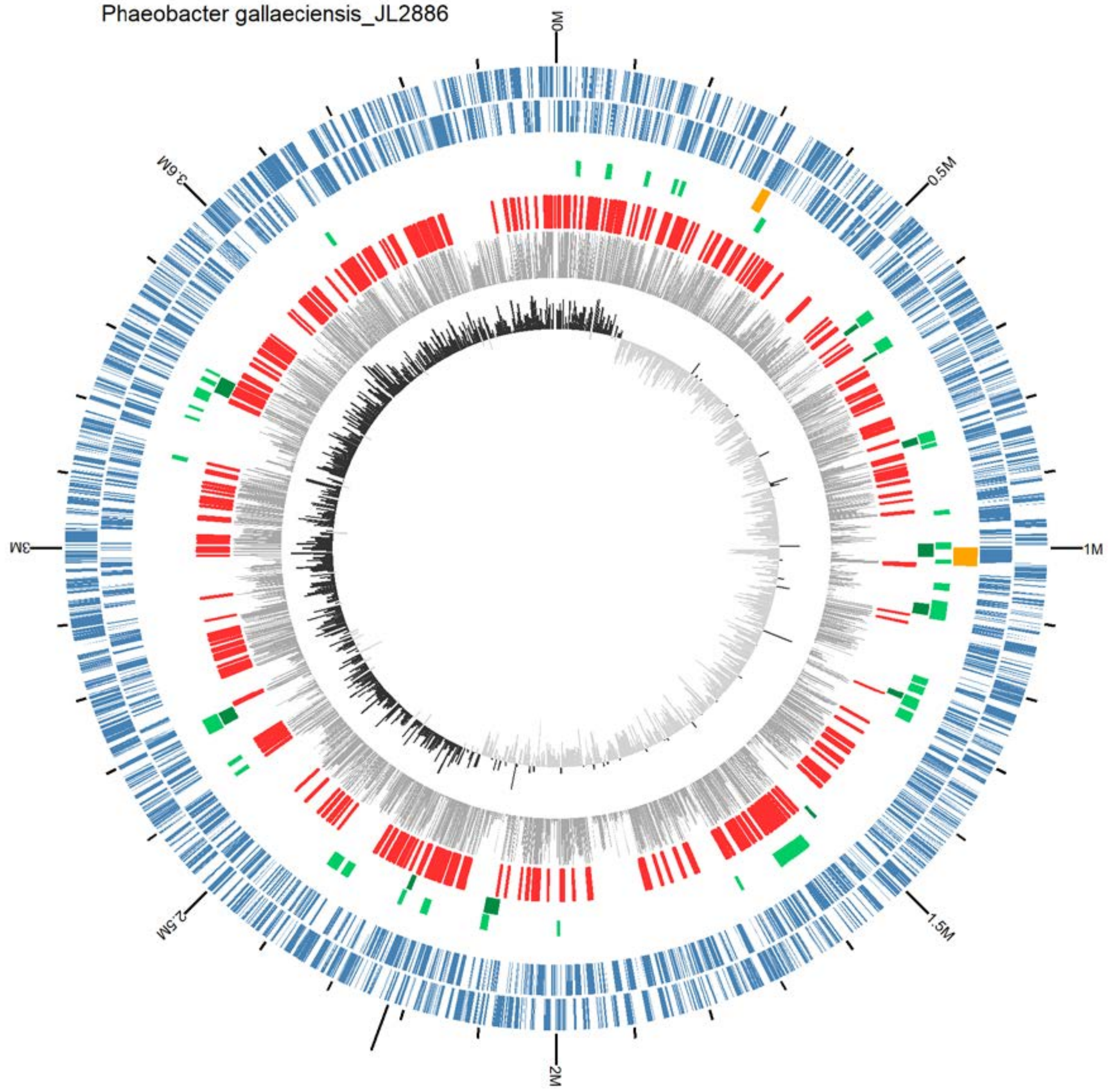

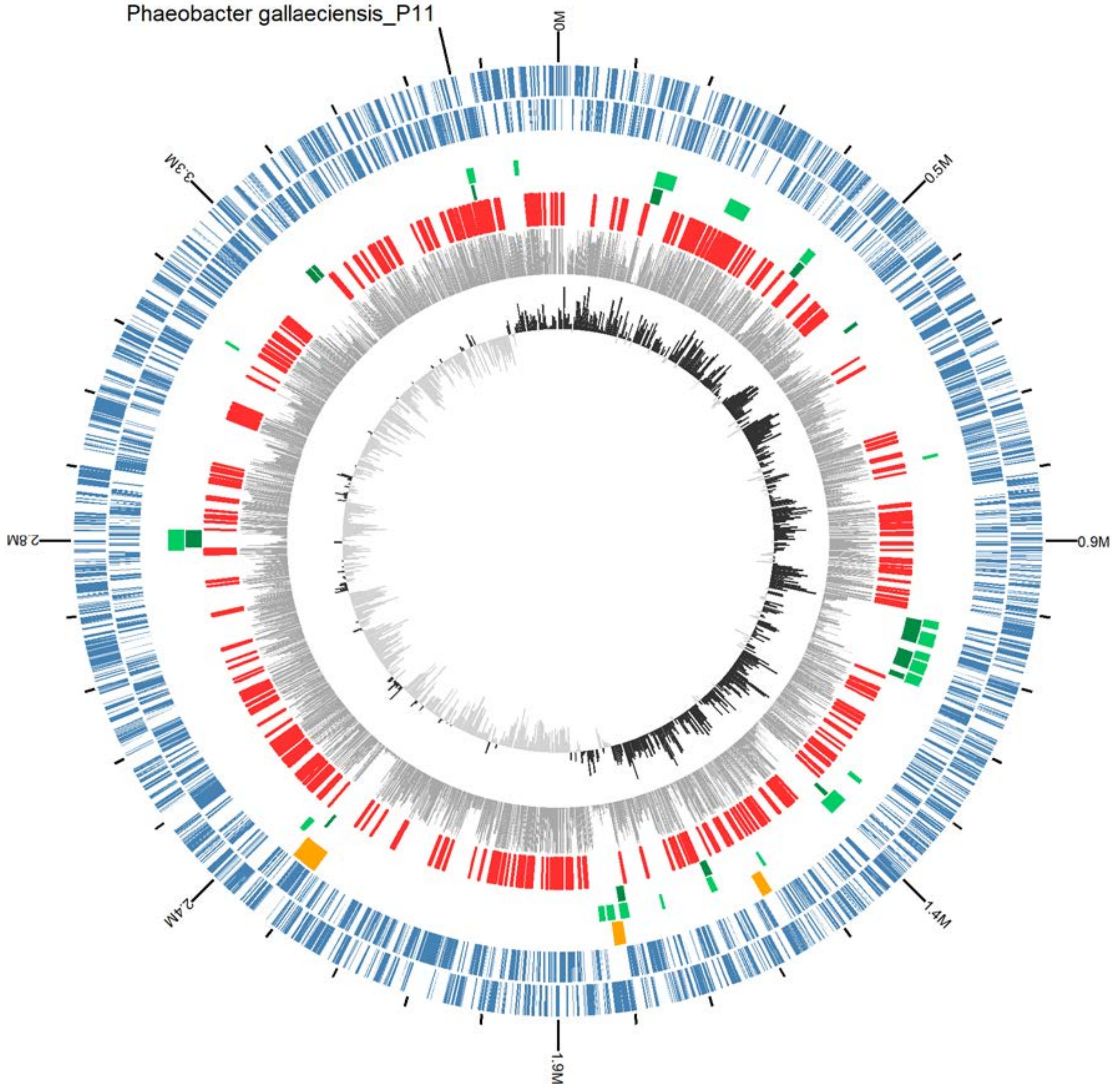

Phaeobacter gallaeciensis_P11
0M
0.5M
0.9M
1.4M
1.9M
2.4M
2.8M
3.3M

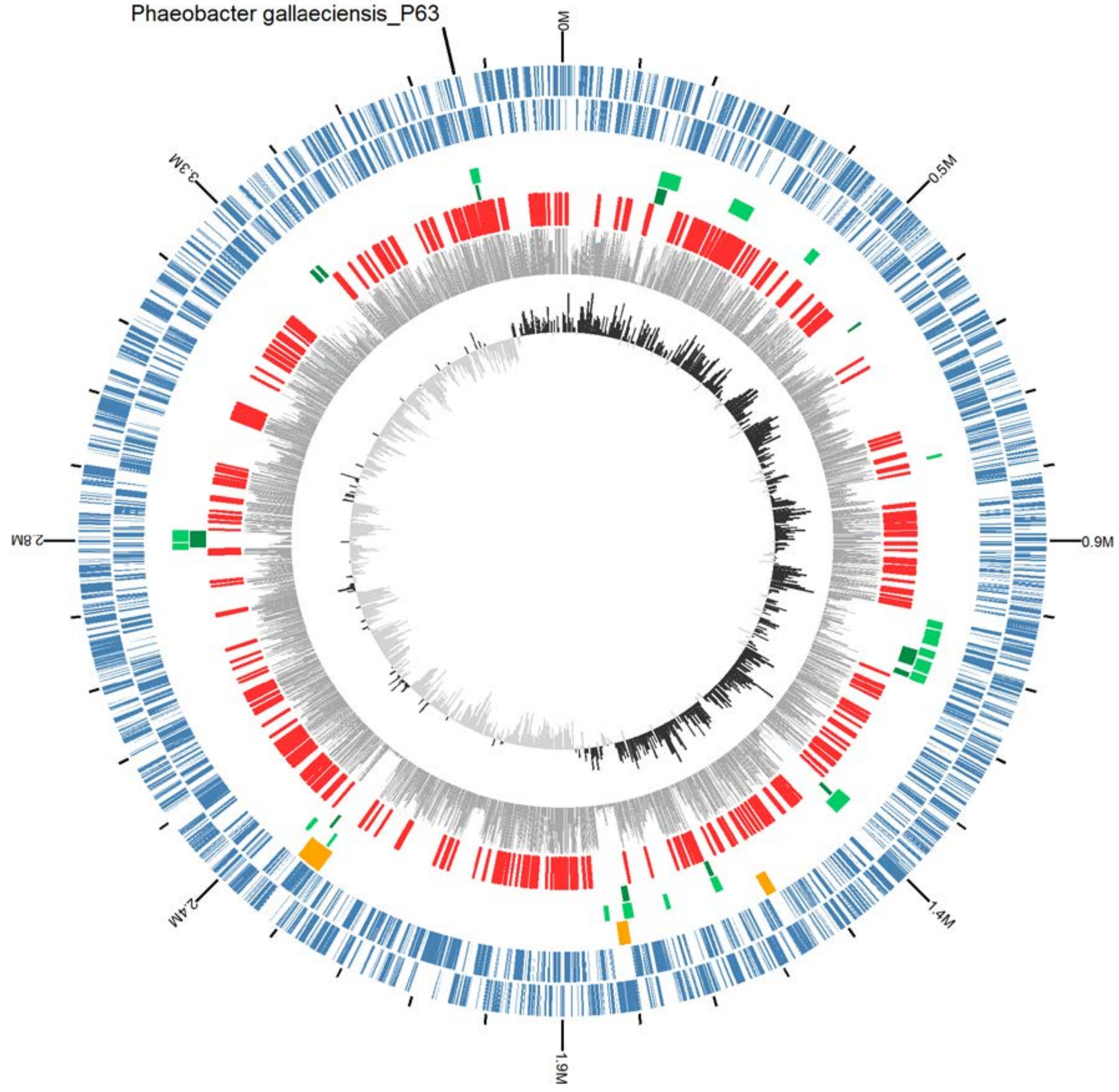

Phaeobacter gallaeciensis_P63
0M
0.5M
0.9M
1.4M
1.9M
2.4M
2.8M
3.3M

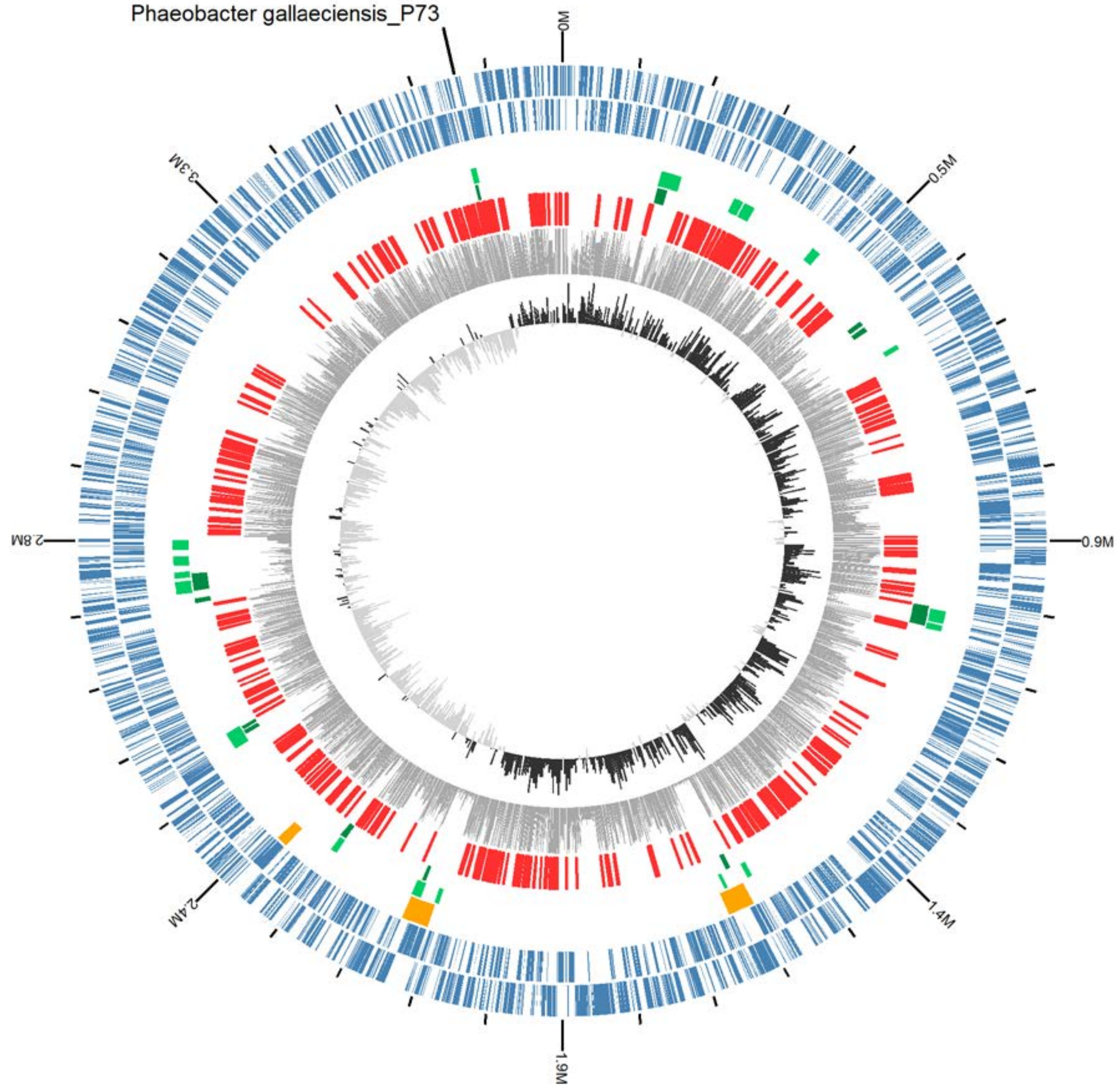

Phaeobacter gallaeciensis_P73
0M
0.5M
0.9M
1.4M
1.9M
2.4M
2.8M
3.3M

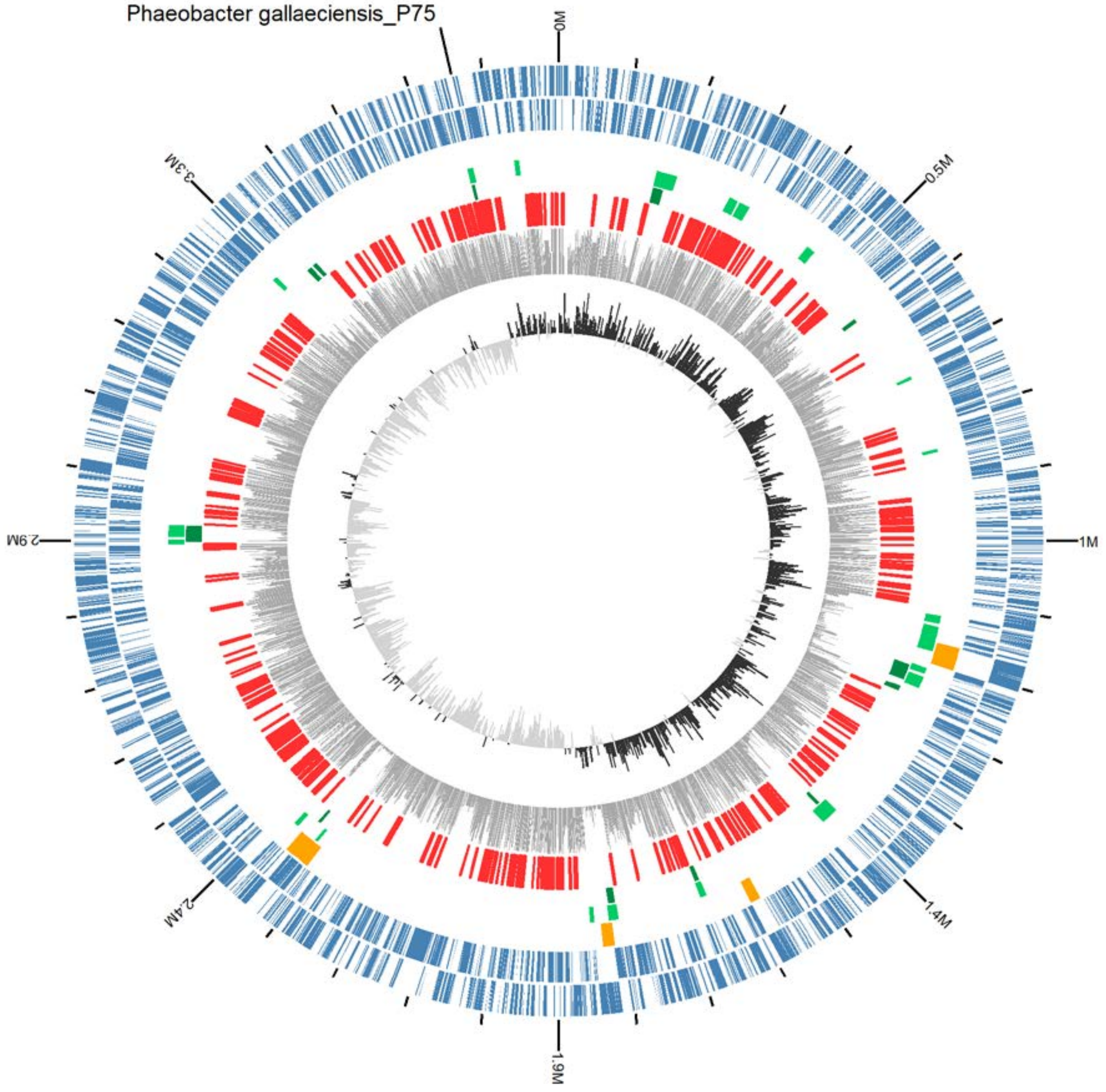

Phaeobacter gallaeciensis_P75
0M
0.5M
1M
1.4M
1.9M
2.4M
2.9M
3.3M

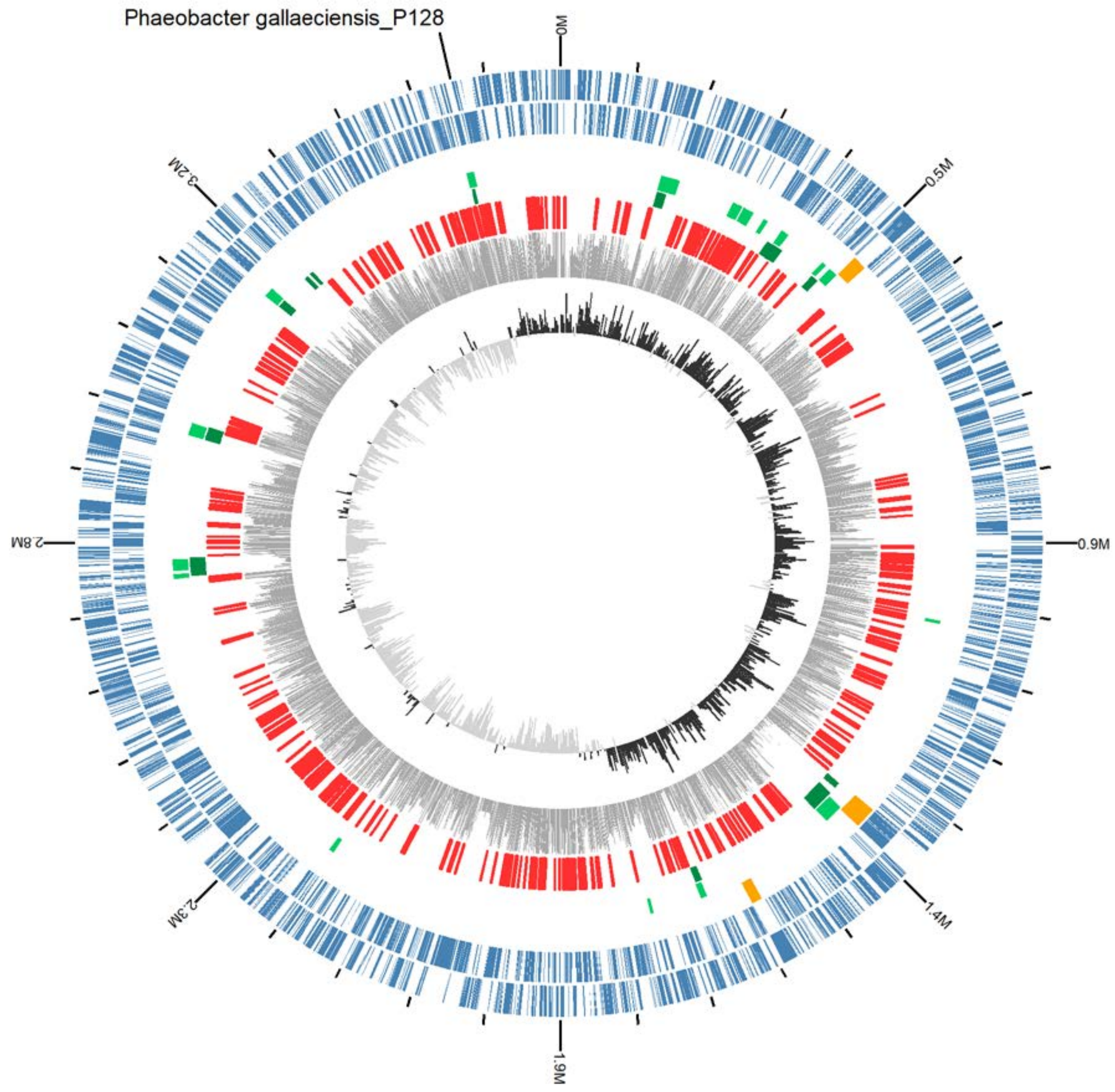
Phaeobacter gallaeciensis_P128
0M
0.5M
0.9M
1.4M
1.9M
2.3M
2.8M
3.2M

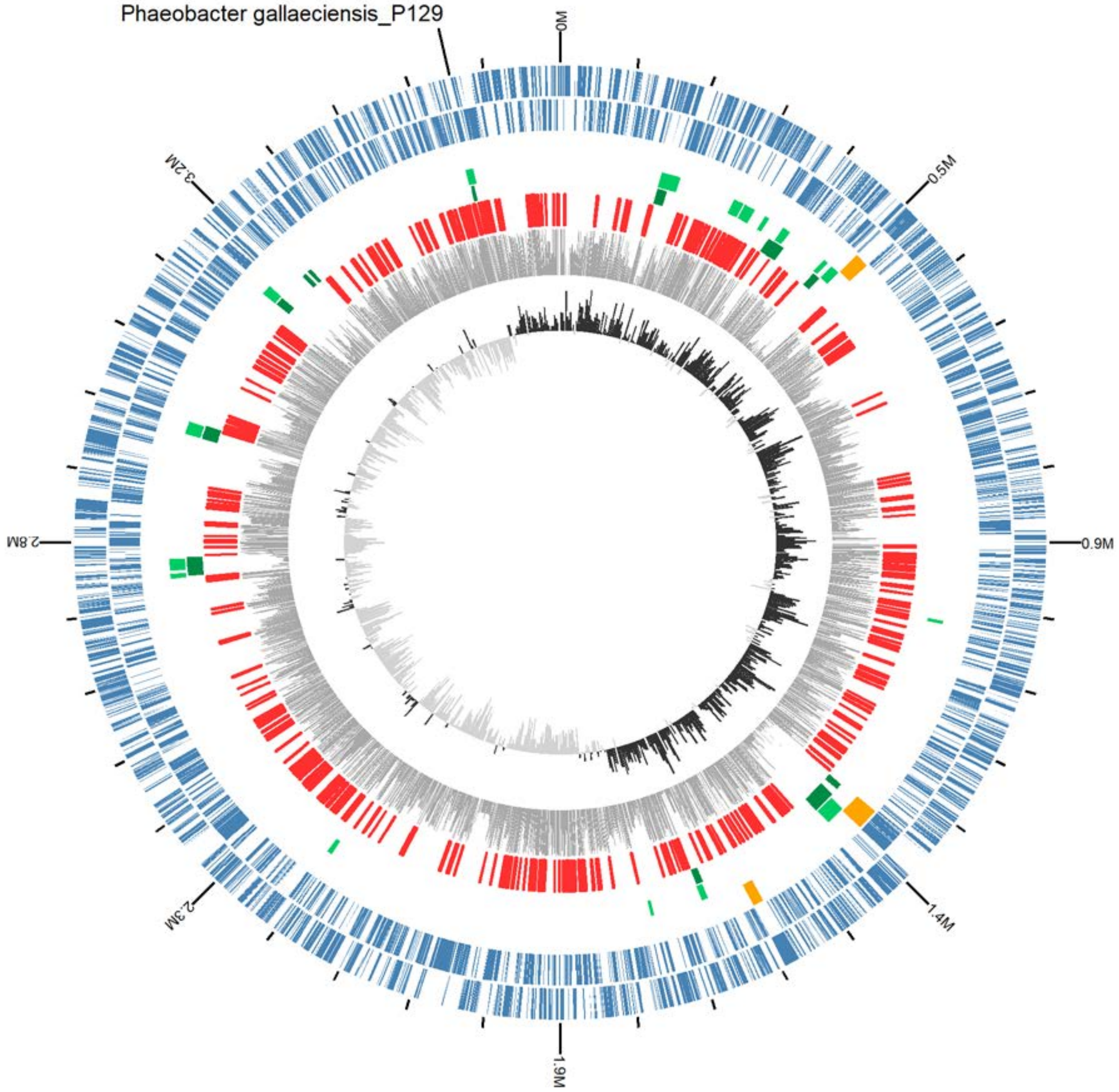
Phaeobacter gallaeciensis_P129
0M
0.5M
0.9M
1.4M
1.9M
2.3M
2.8M
3.2M

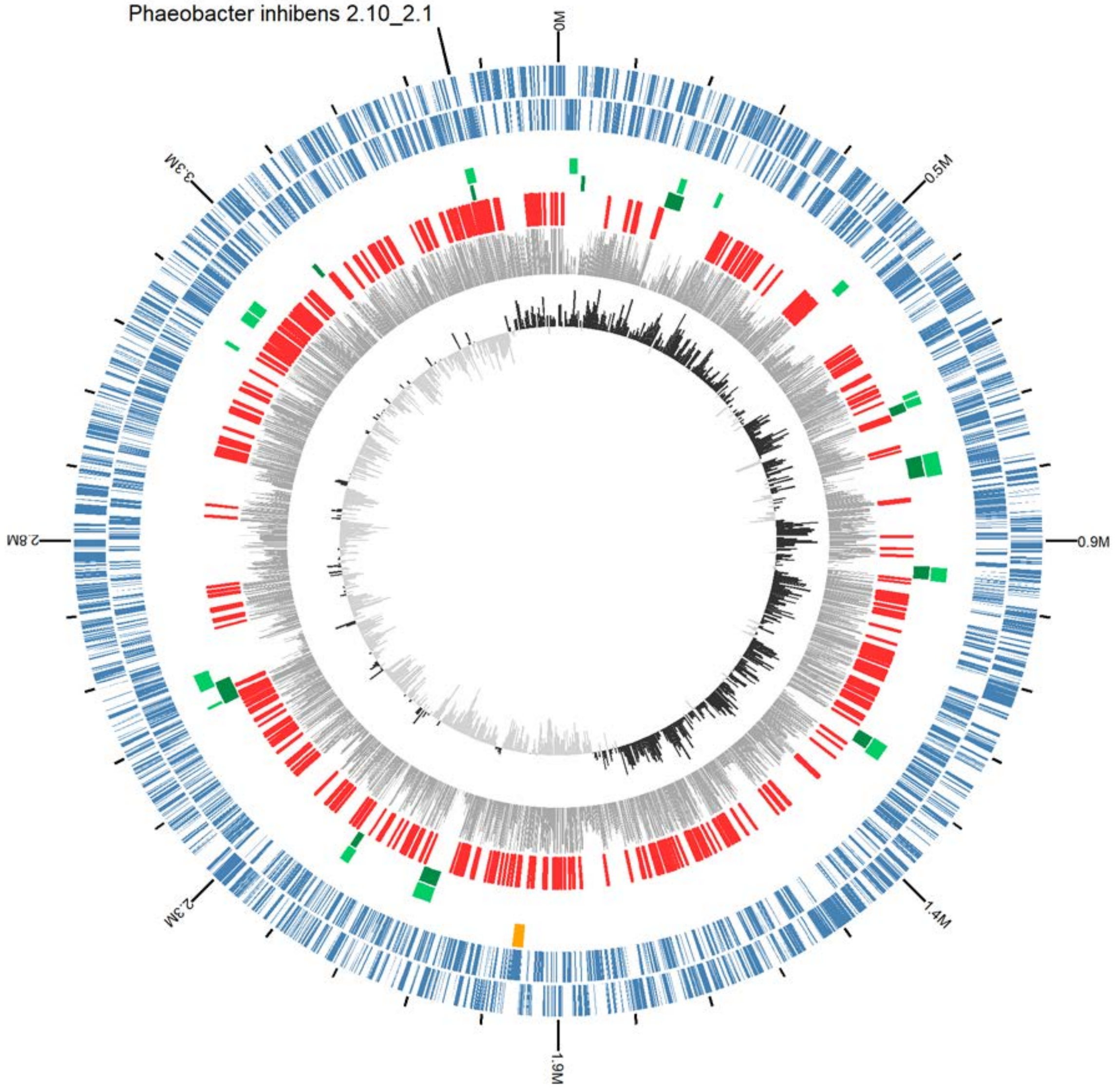
Phaeobacter inhibens 2.10_2.1
0M
0.5M
0.9M
1.4M
1.9M
2.3M
2.8M
3.3M

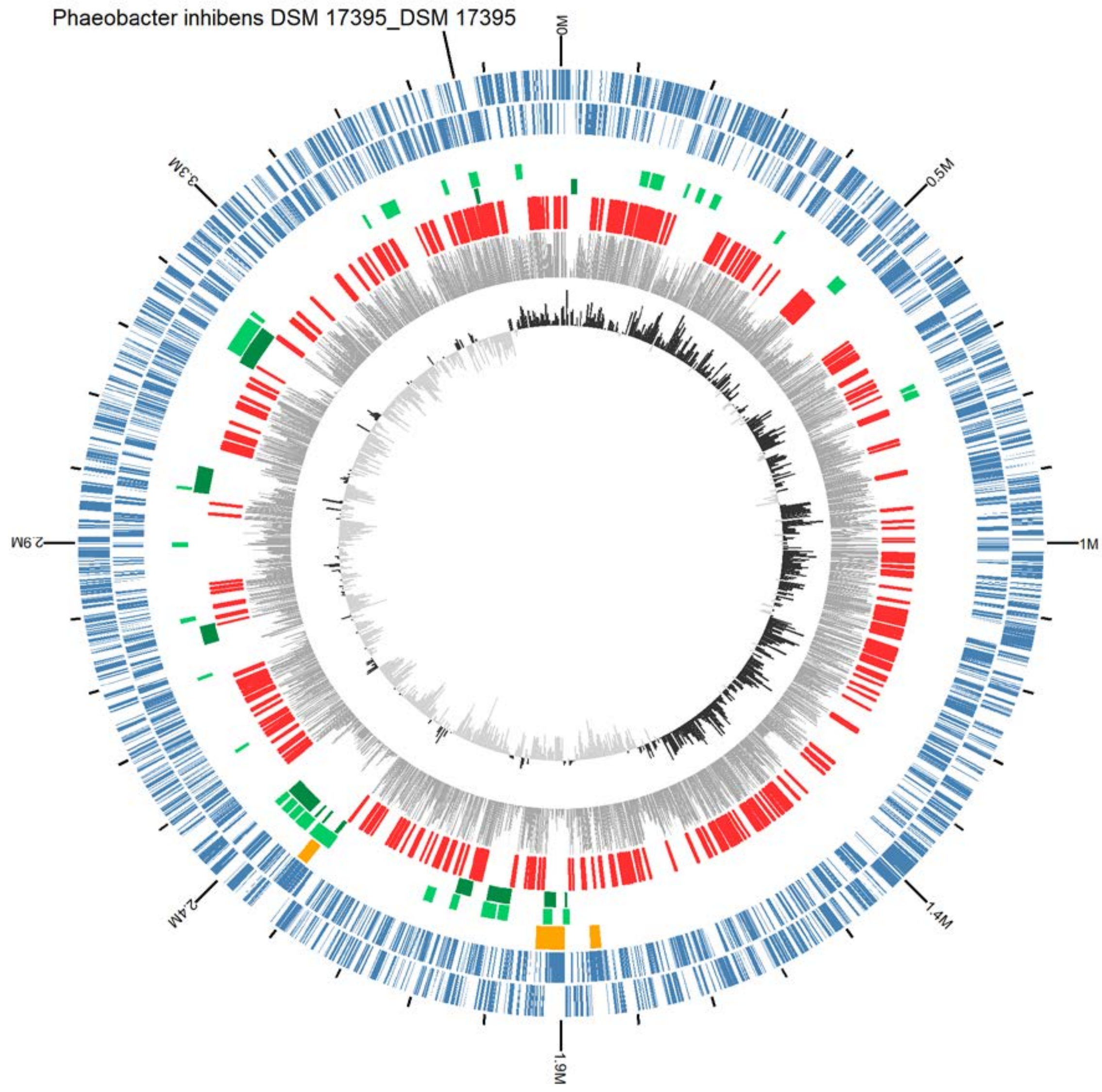

Phaeobacter inhibens DSM 17395_DSM 17395
0M
0.5M
1M
1.4M
1.9M
2.4M
2.9M
3.3M

Phaeobacter inhibens_DOK1-1

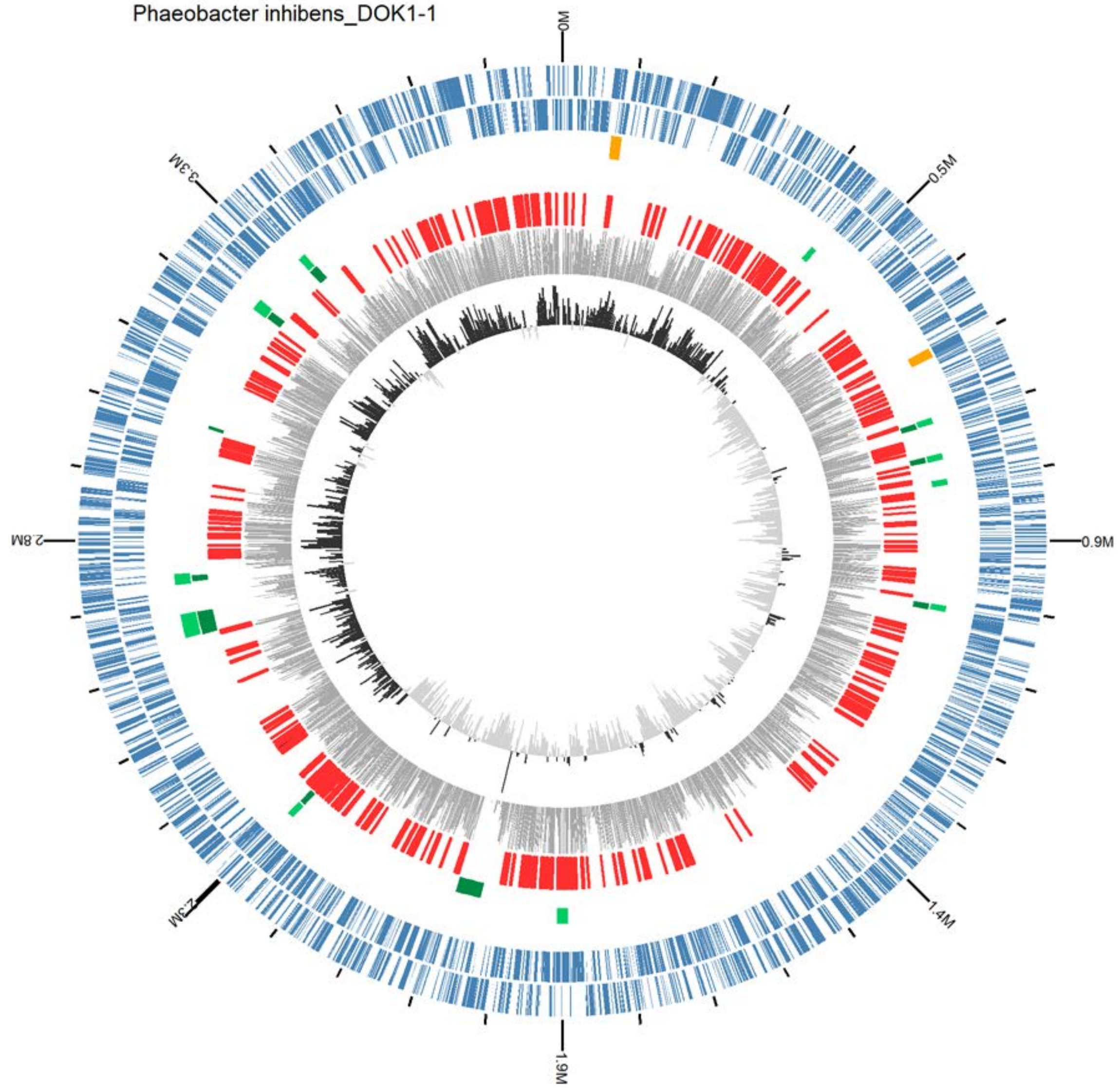

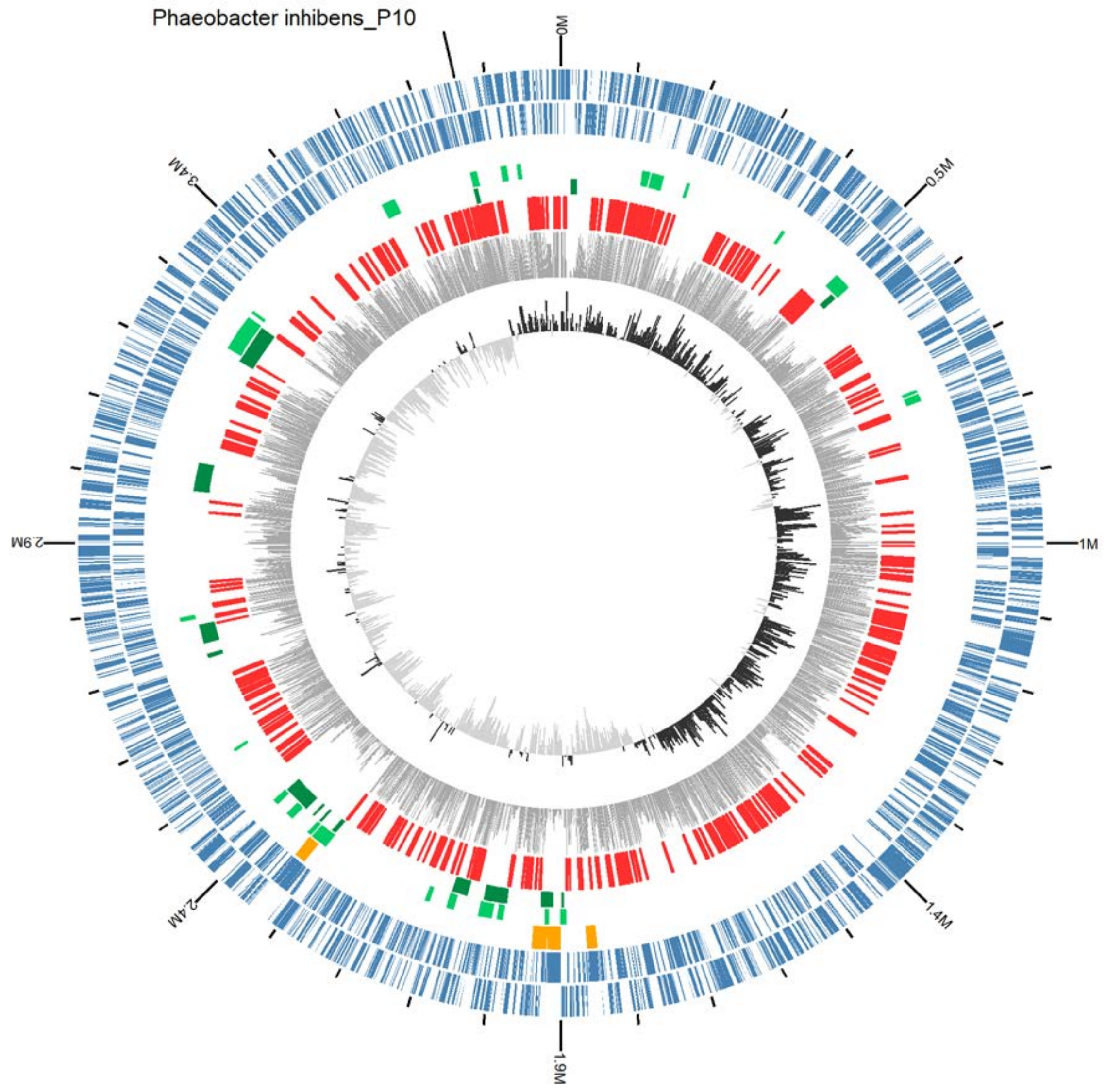
Phaeobacter inhibens_P10
0M
0.5M
1M
1.4M
1.9M
2.4M
2.9M
3.4M

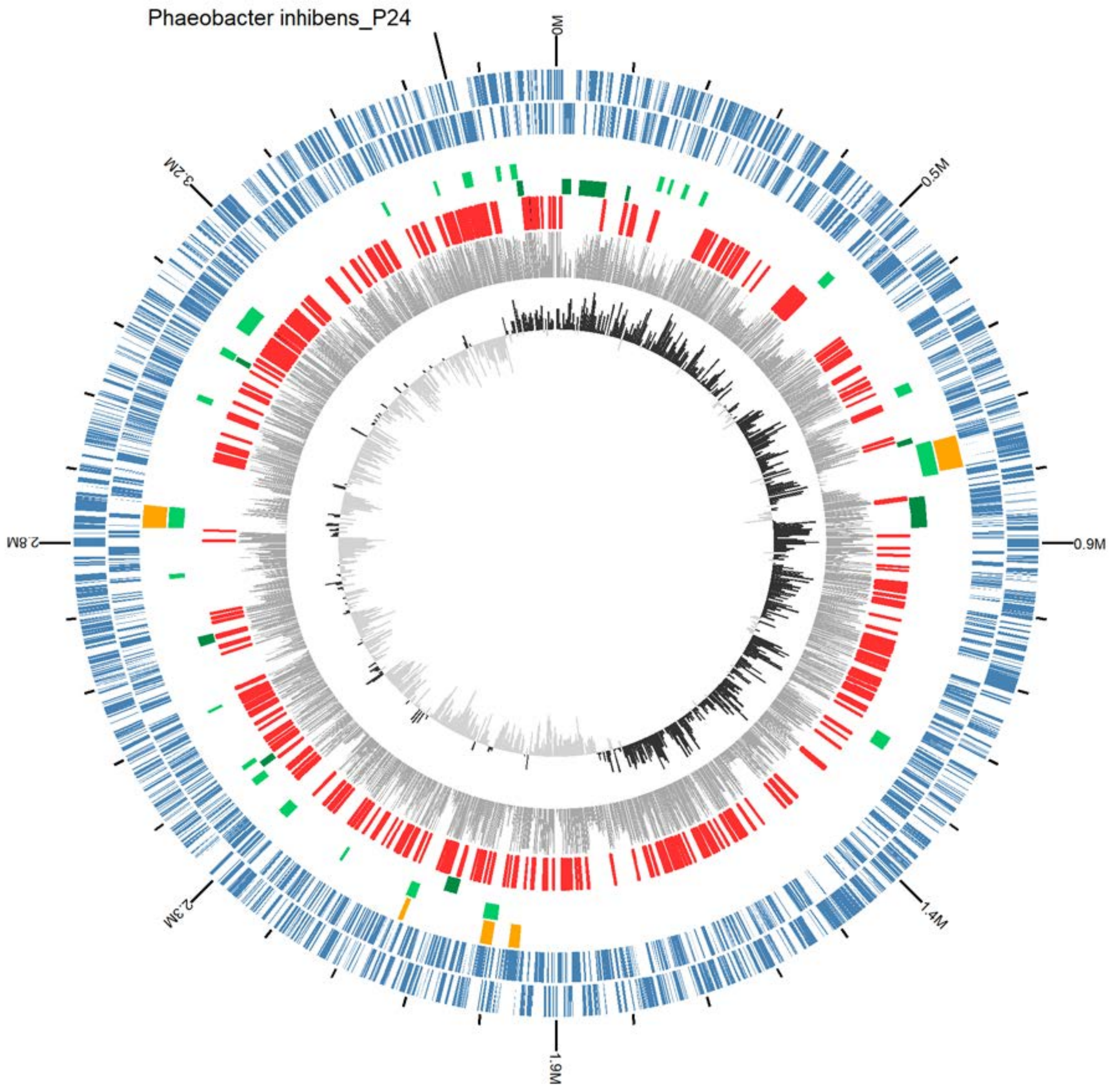
Phaeobacter inhibens_P24
0M
0.5M
0.9M
1.4M
1.9M
2.3M
2.8M
3.2M

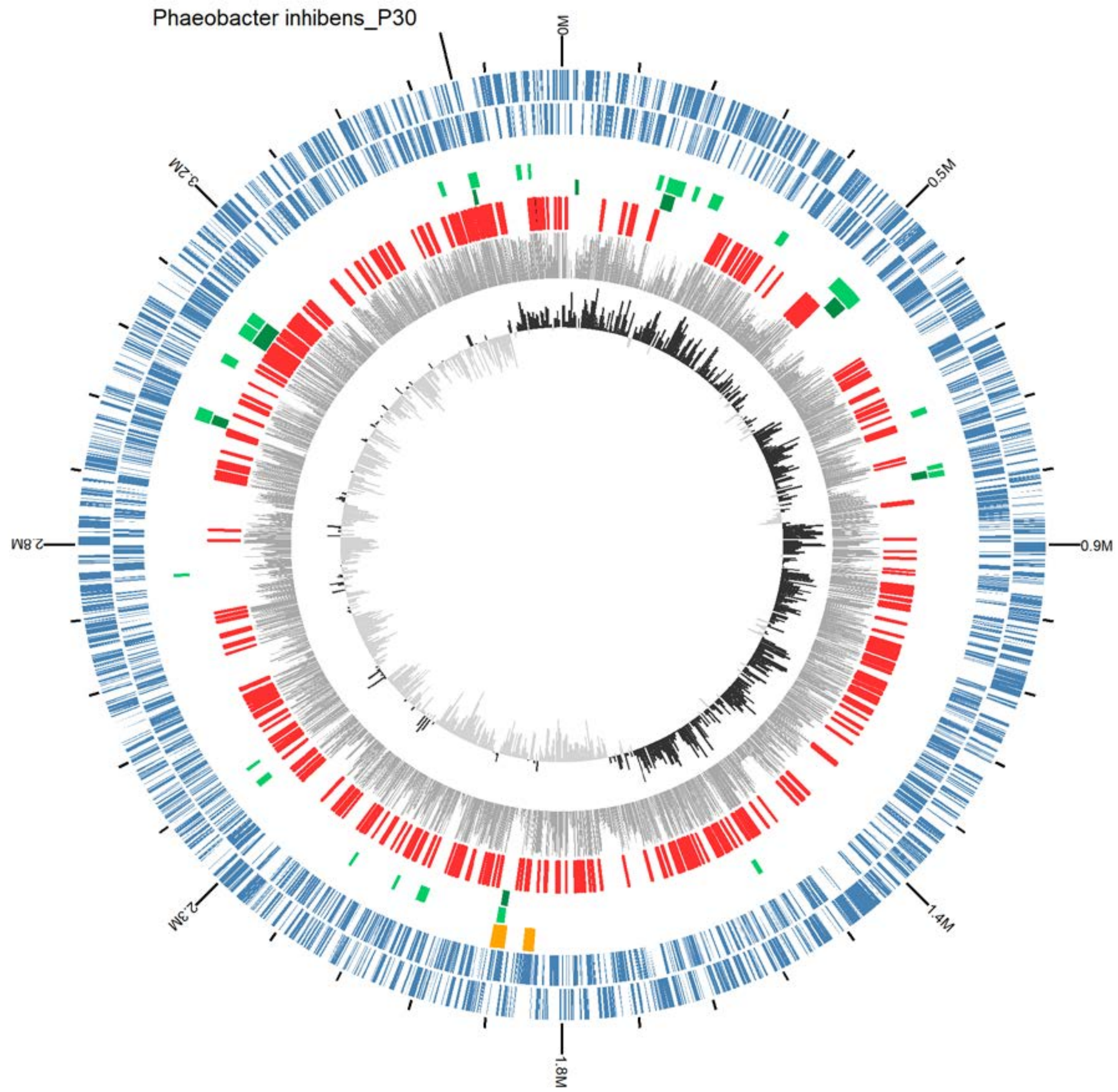
Phaeobacter inhibens_P30
0M
0.5M
0.9M
1.4M
1.8M
2.3M
2.8M
3.2M

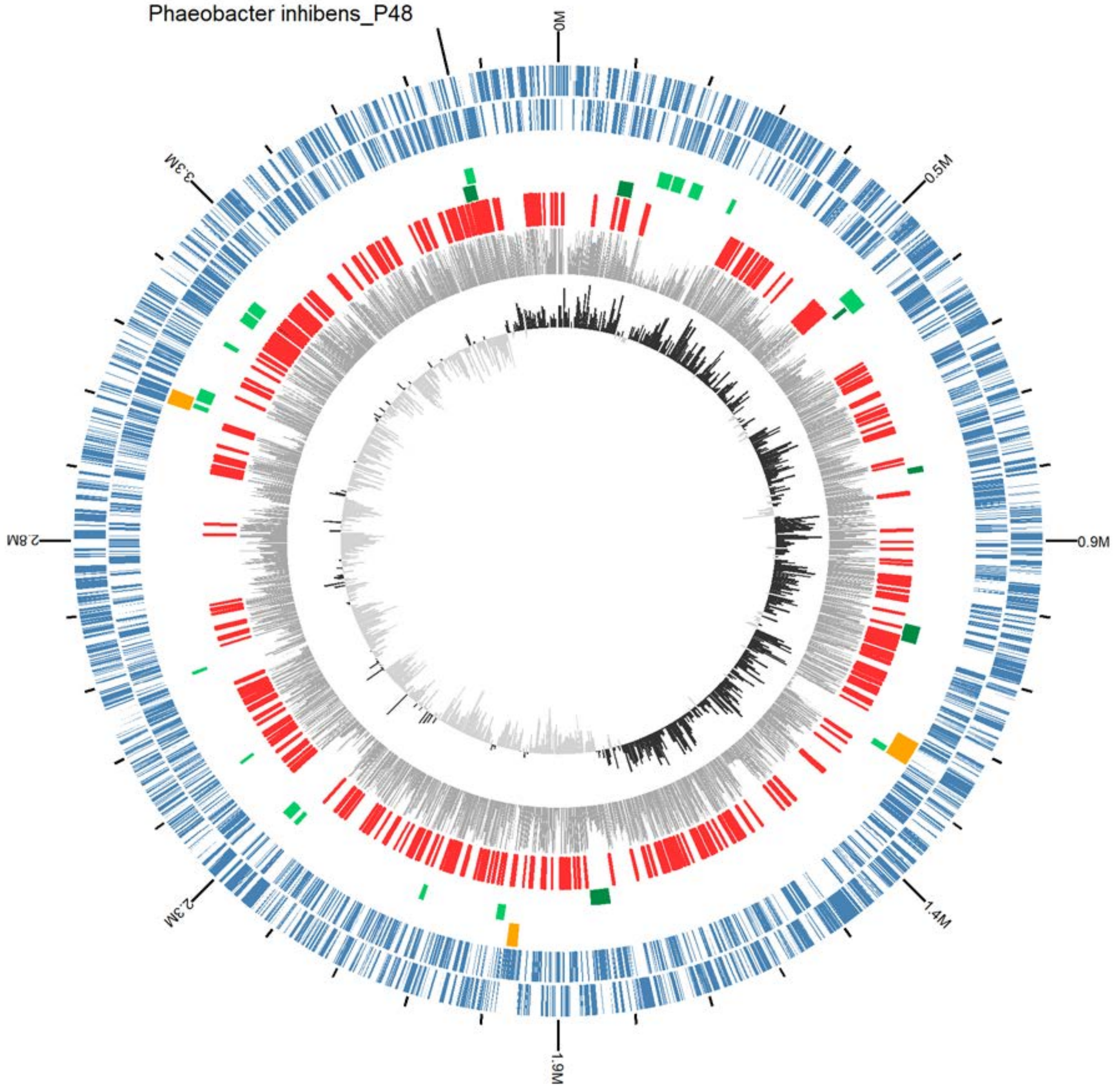
Phaeobacter inhibens_P48
0M
0.5M
0.9M
1.4M
1.9M
2.3M
2.8M
3.3M

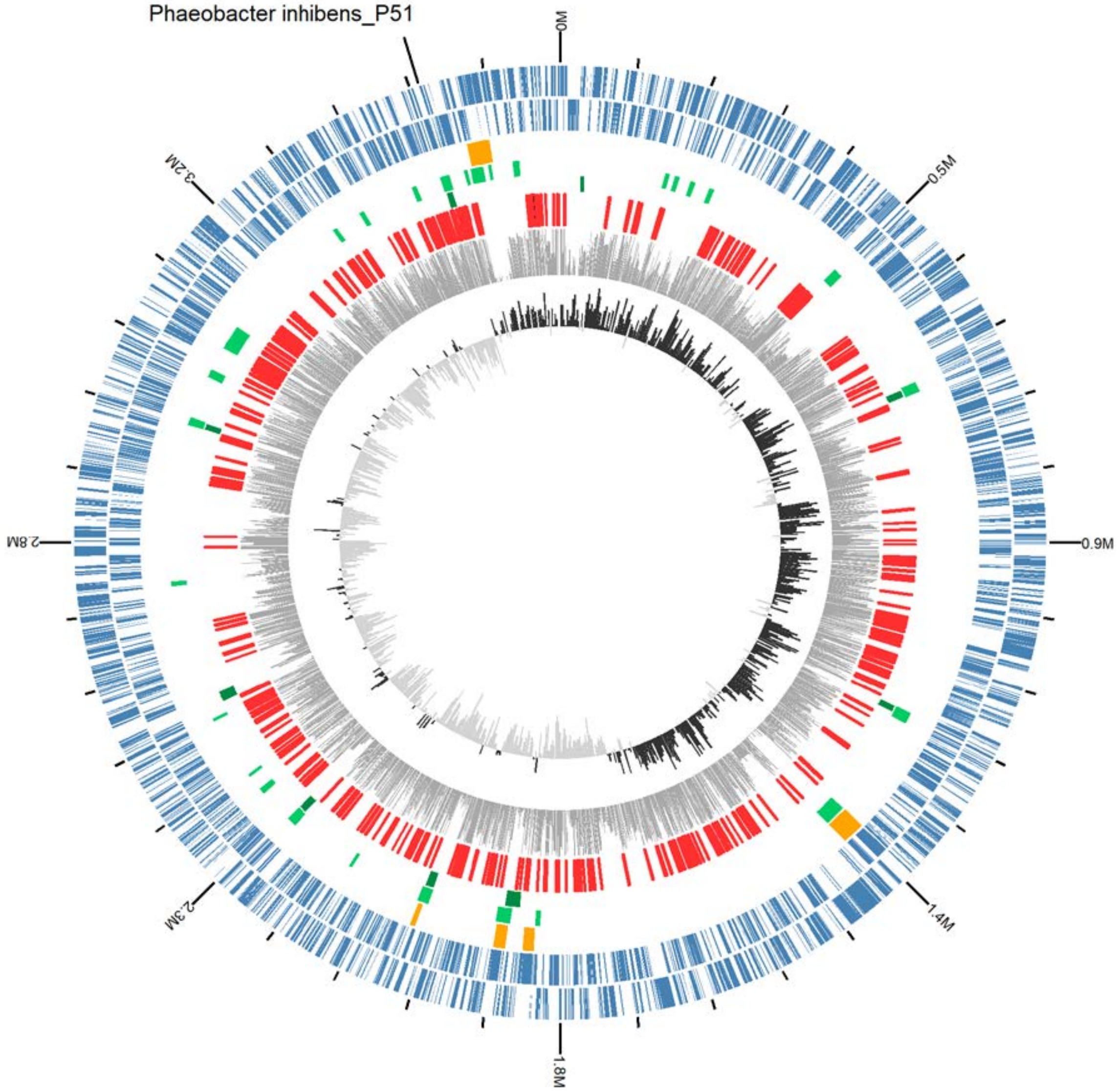
Phaeobacter inhibens_P51
0M
0.5M
0.9M
1.4M
1.8M
2.3M
2.8M
3.2M

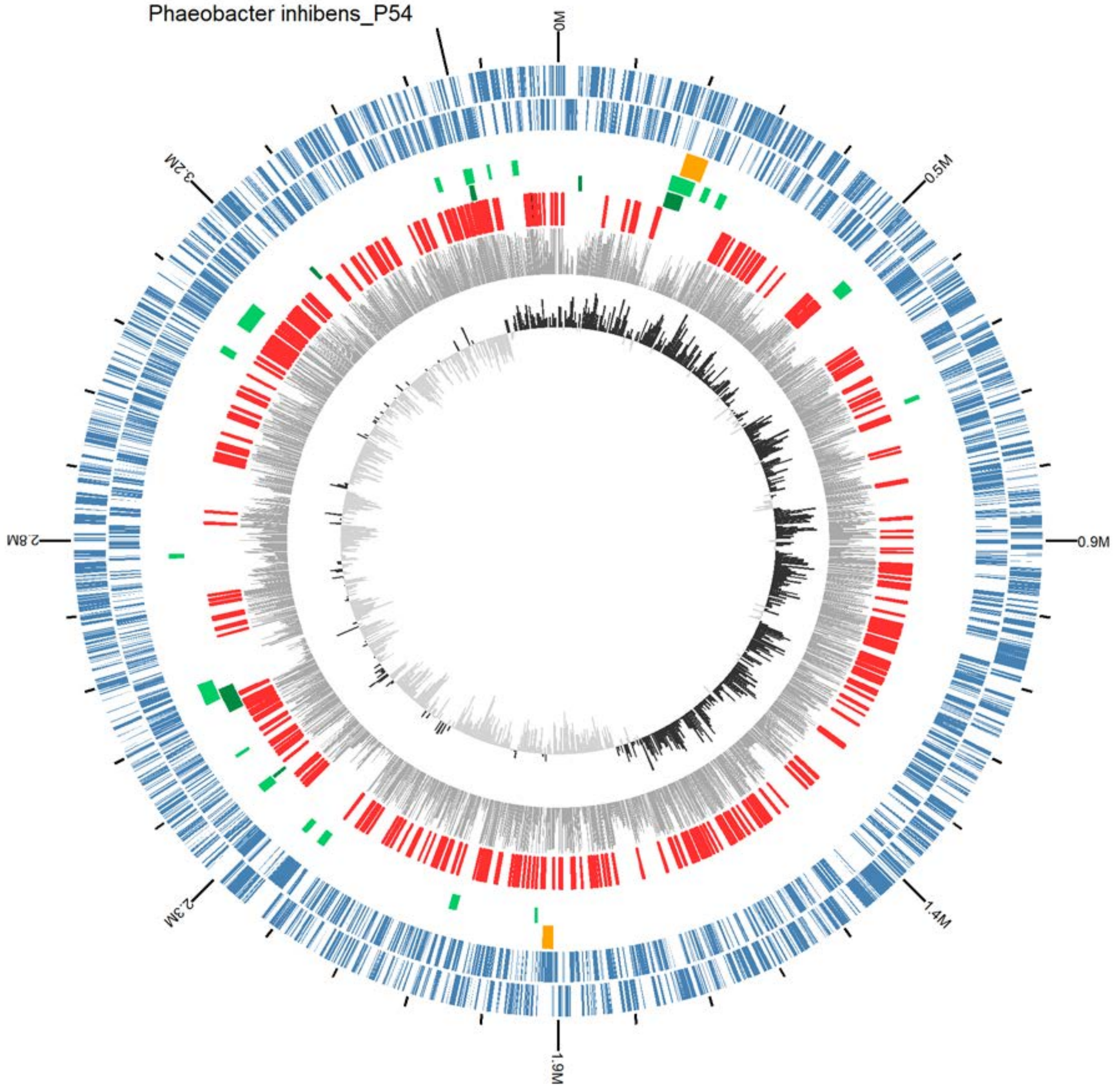
Phaeobacter inhibens_P54
0M
0.5M
0.9M
1.4M
1.9M
2.3M
2.8M
3.2M

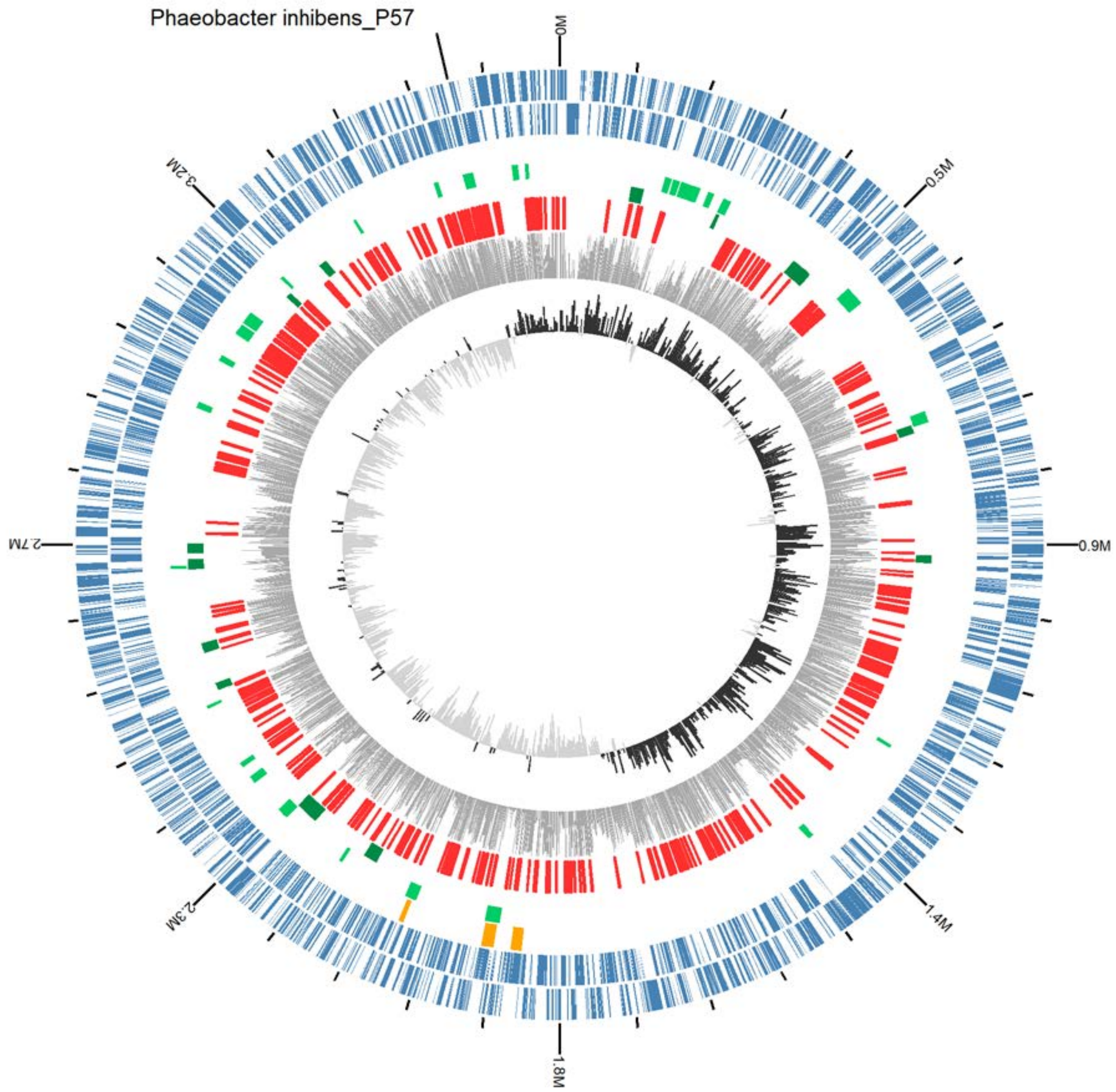

Phaeobacter inhibens_P57
0M
0.5M
0.9M
1.4M
1.8M
2.3M
2.7M
3.2M

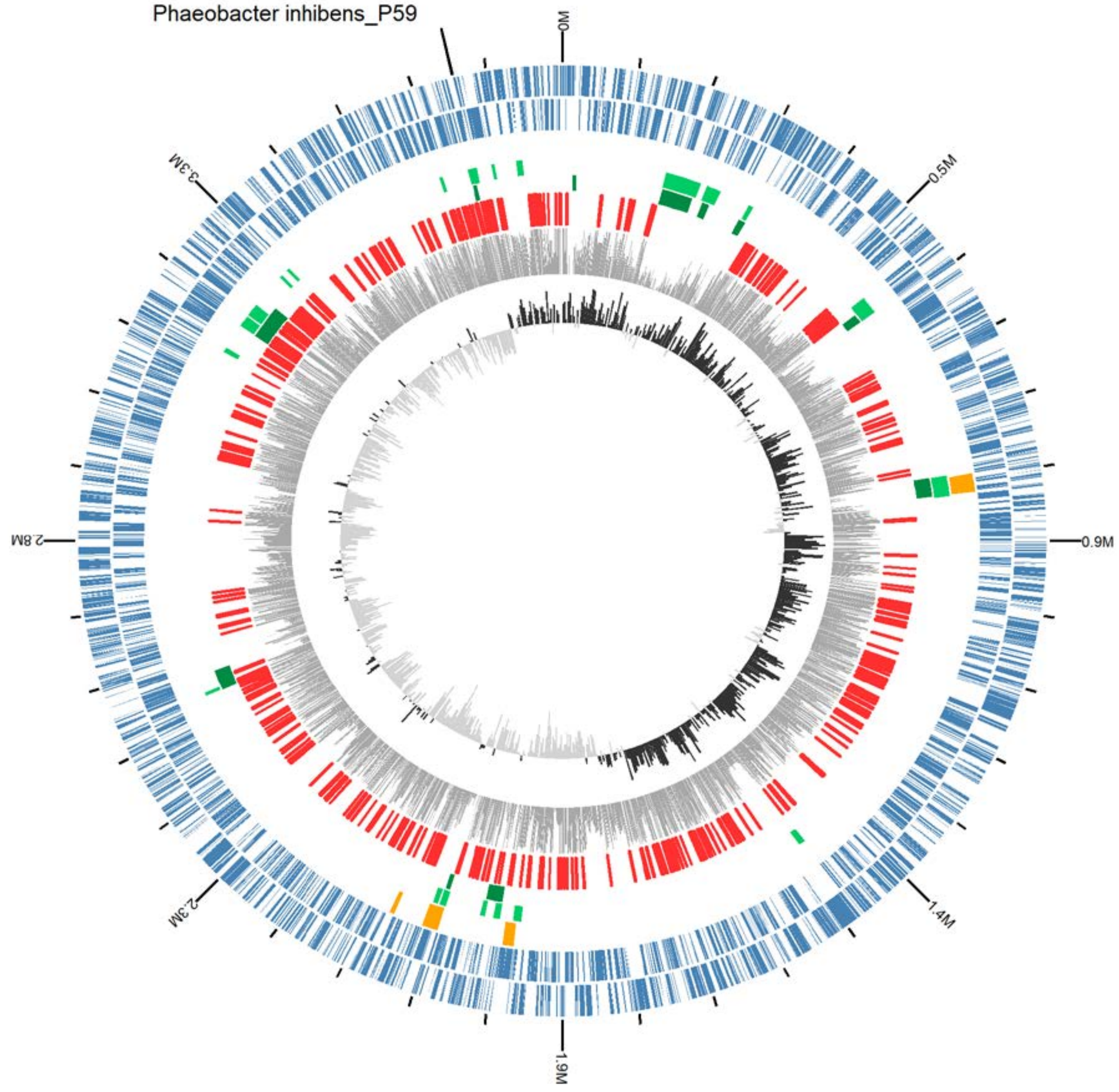

Phaeobacter inhibens_P59
0M
0.5M
0.9M
1.4M
1.9M
2.3M
2.8M
3.3M

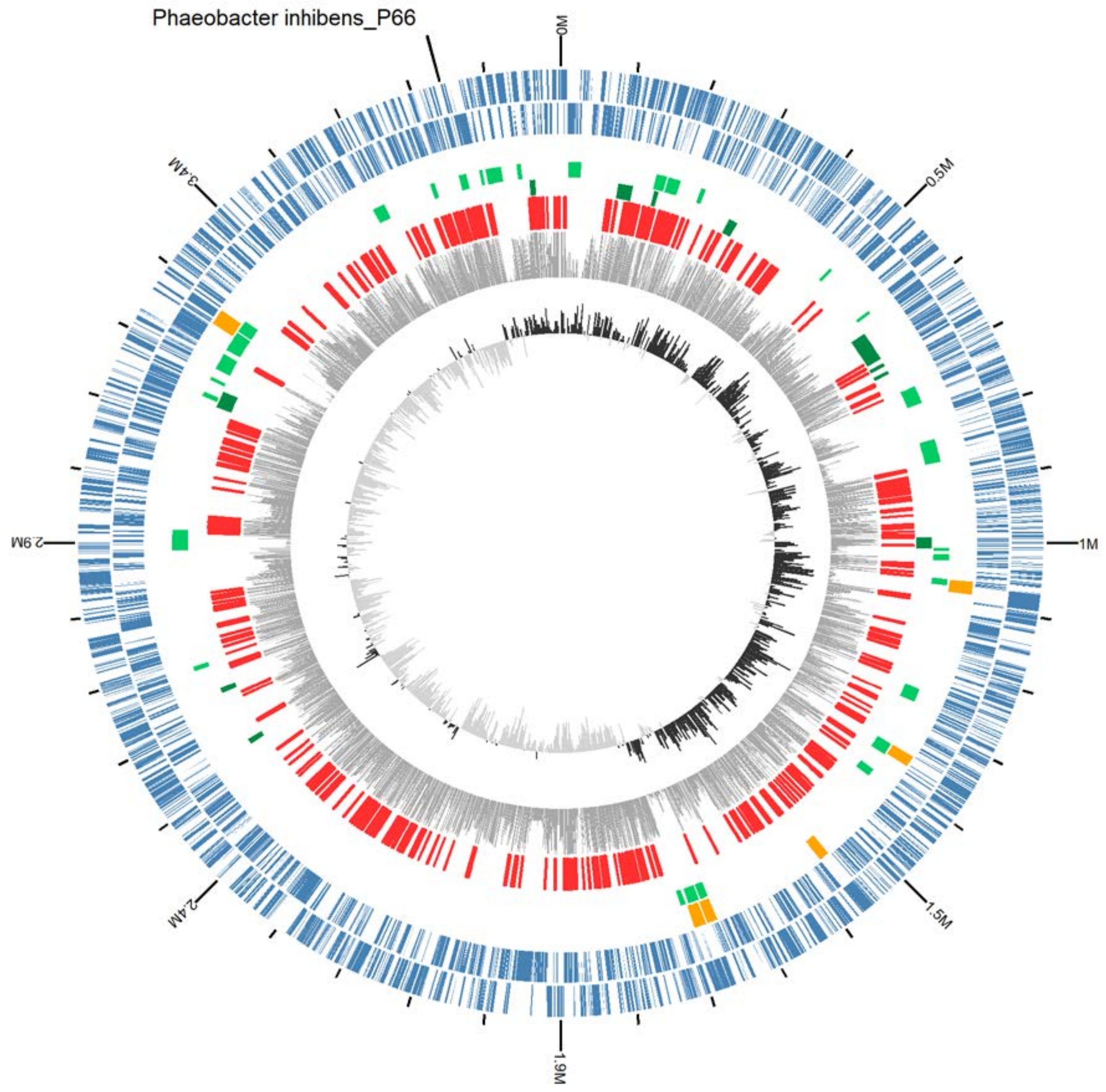
Phaeobacter inhibens_P66
0M
0.5M
1M
1.5M
1.9M
2.4M
2.9M
3.4M

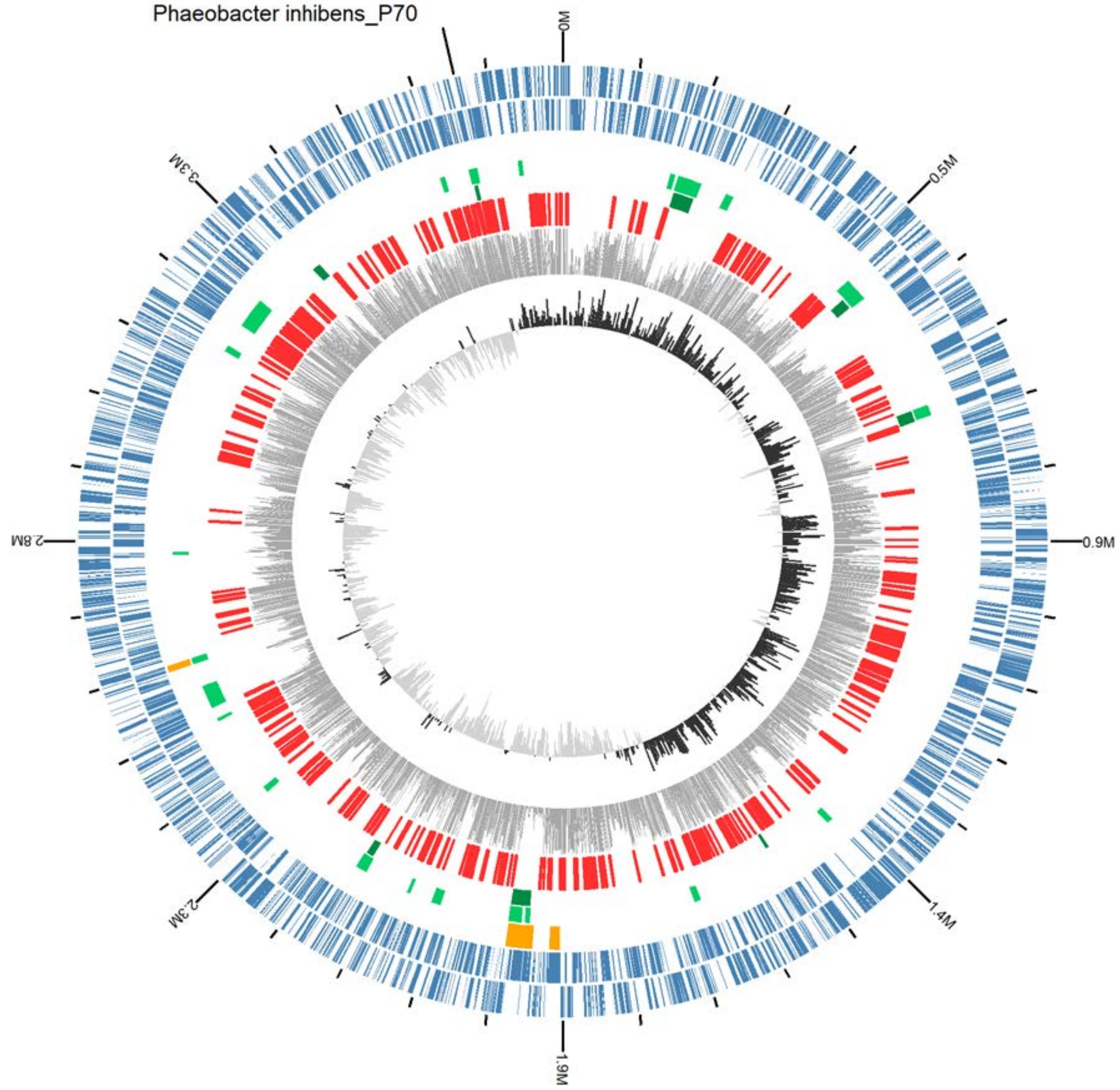

Phaeobacter inhibens_P70
0M
0.5M
0.9M
1.4M
1.9M
2.3M
2.8M
3.3M

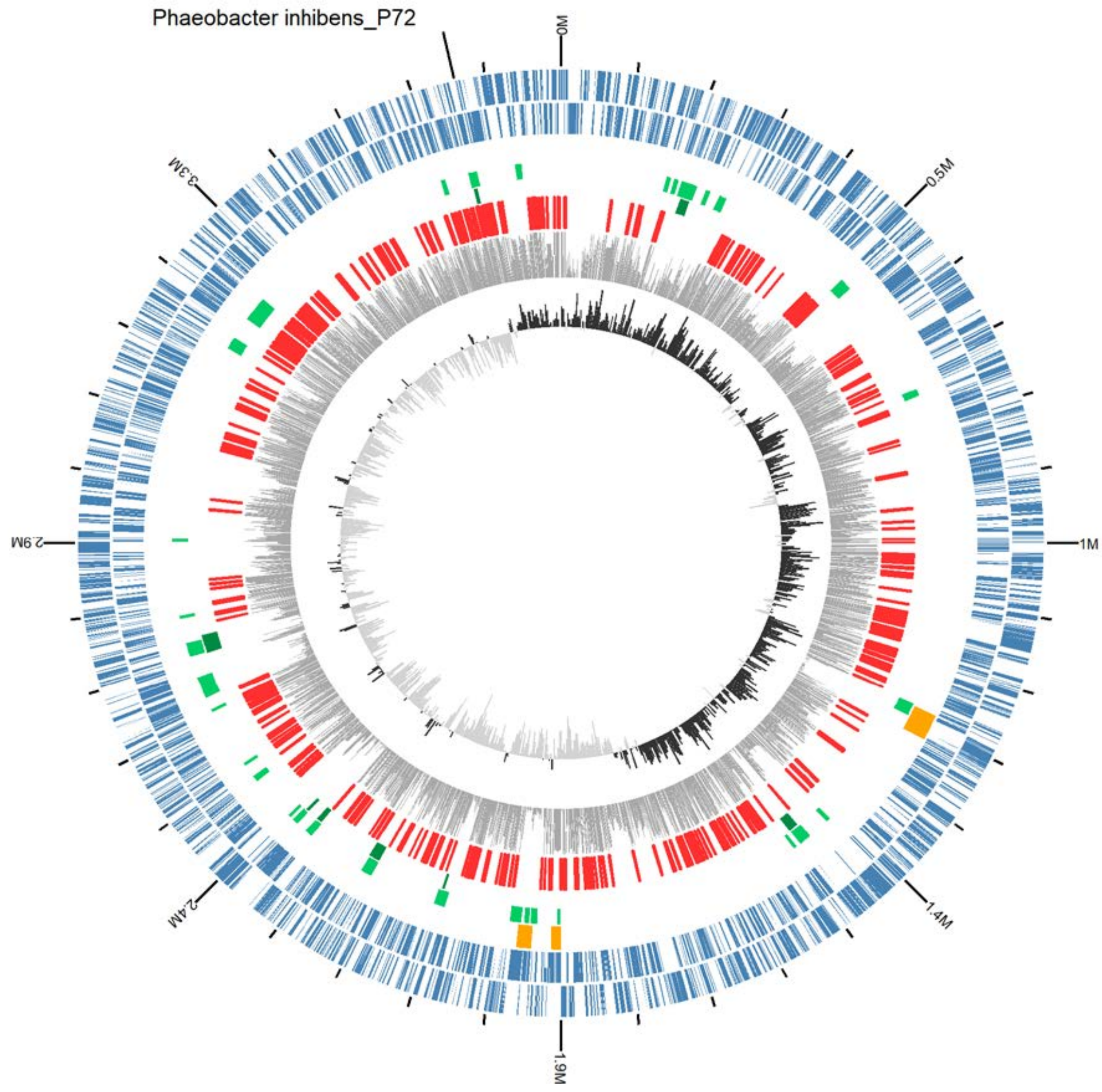

Phaeobacter inhibens_P72
0M
0.5M
1M
1.4M
1.9M
2.4M
2.9M
3.3M

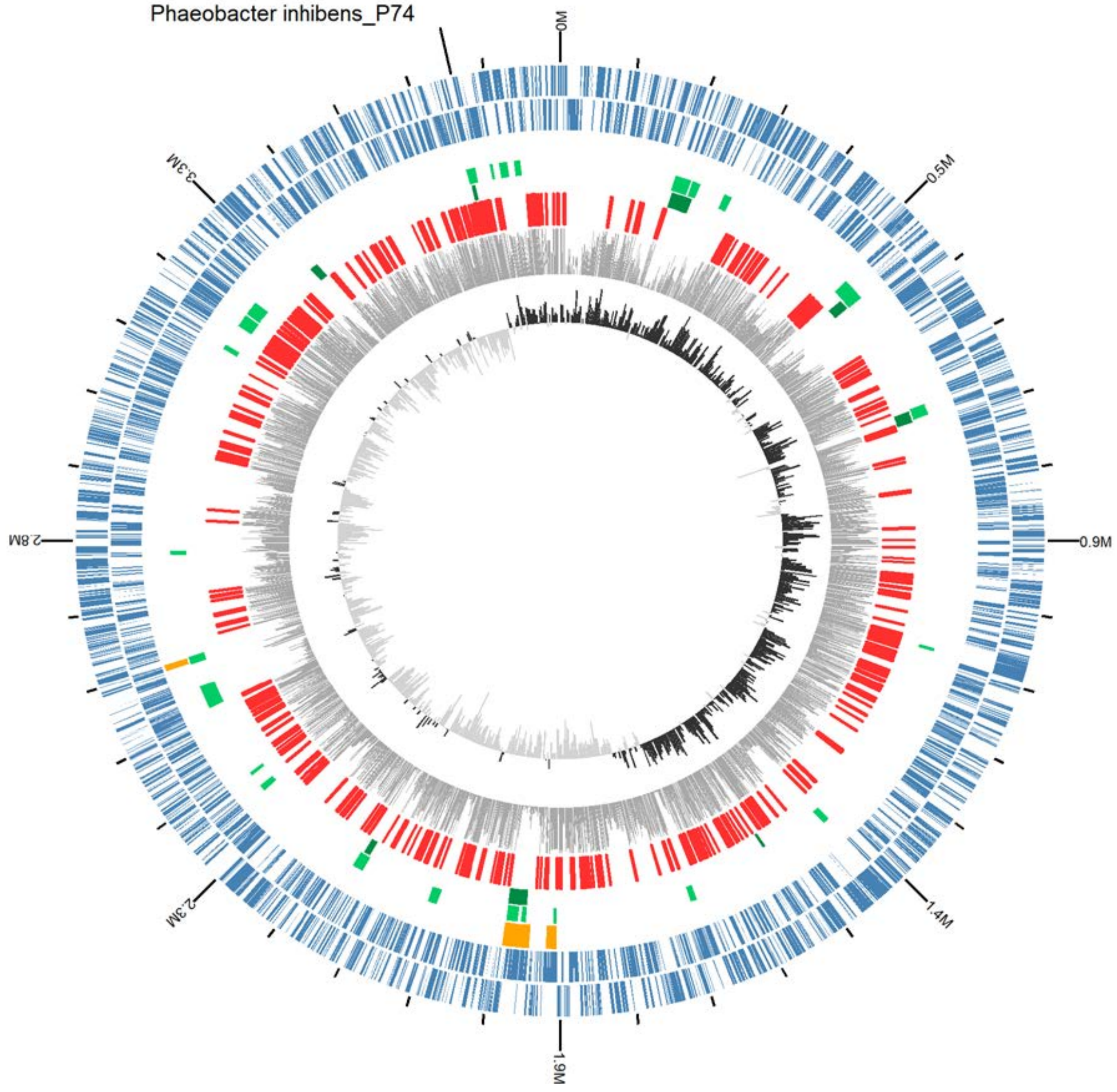

Phaeobacter inhibens_P74
0M
0.5M
0.9M
1.4M
1.9M
2.3M
2.8M
3.3M

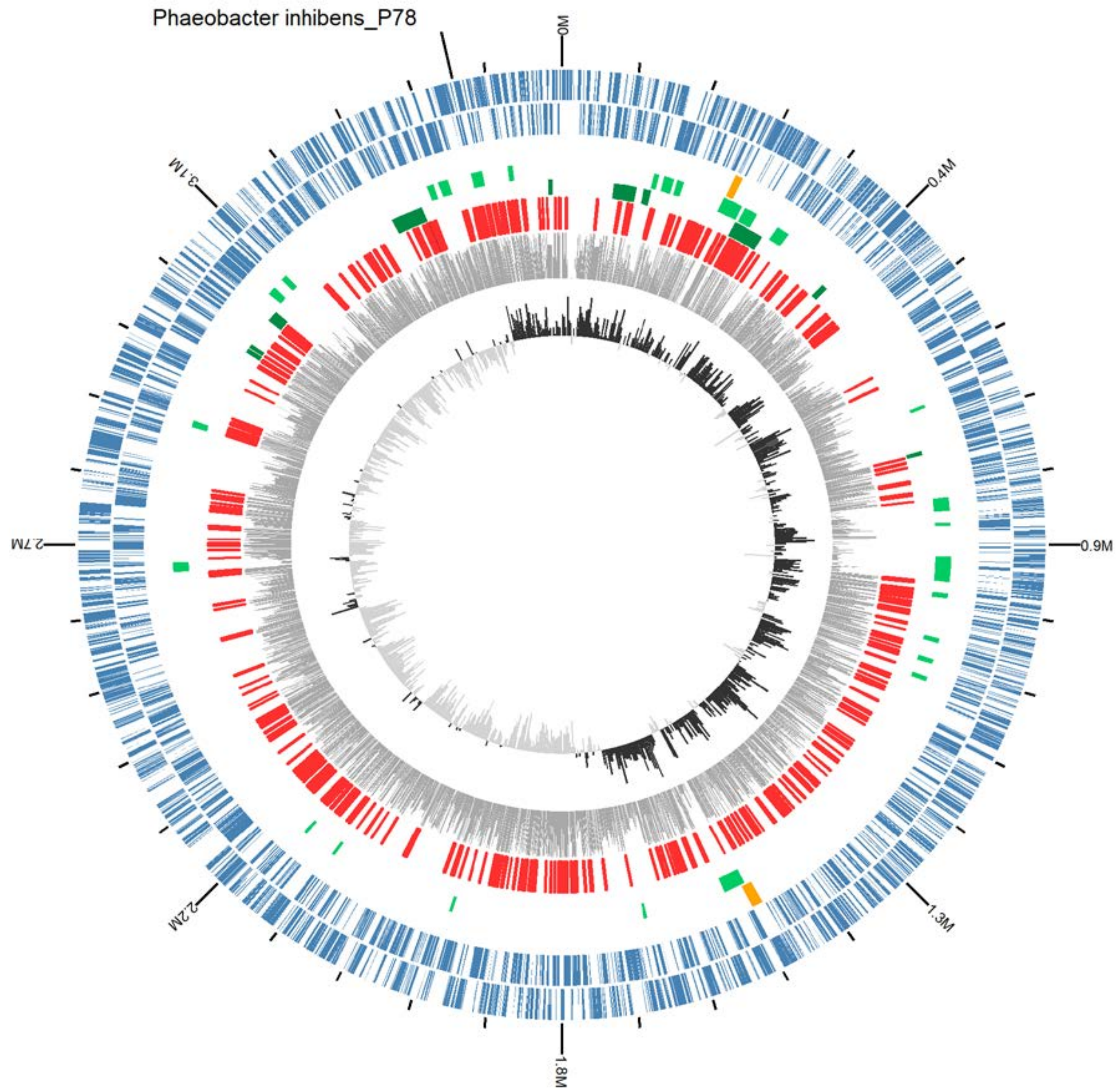

Phaeobacter inhibens_P78
0M
0.4M
0.9M
1.3M
1.8M
2.2M
2.7M
3.1M

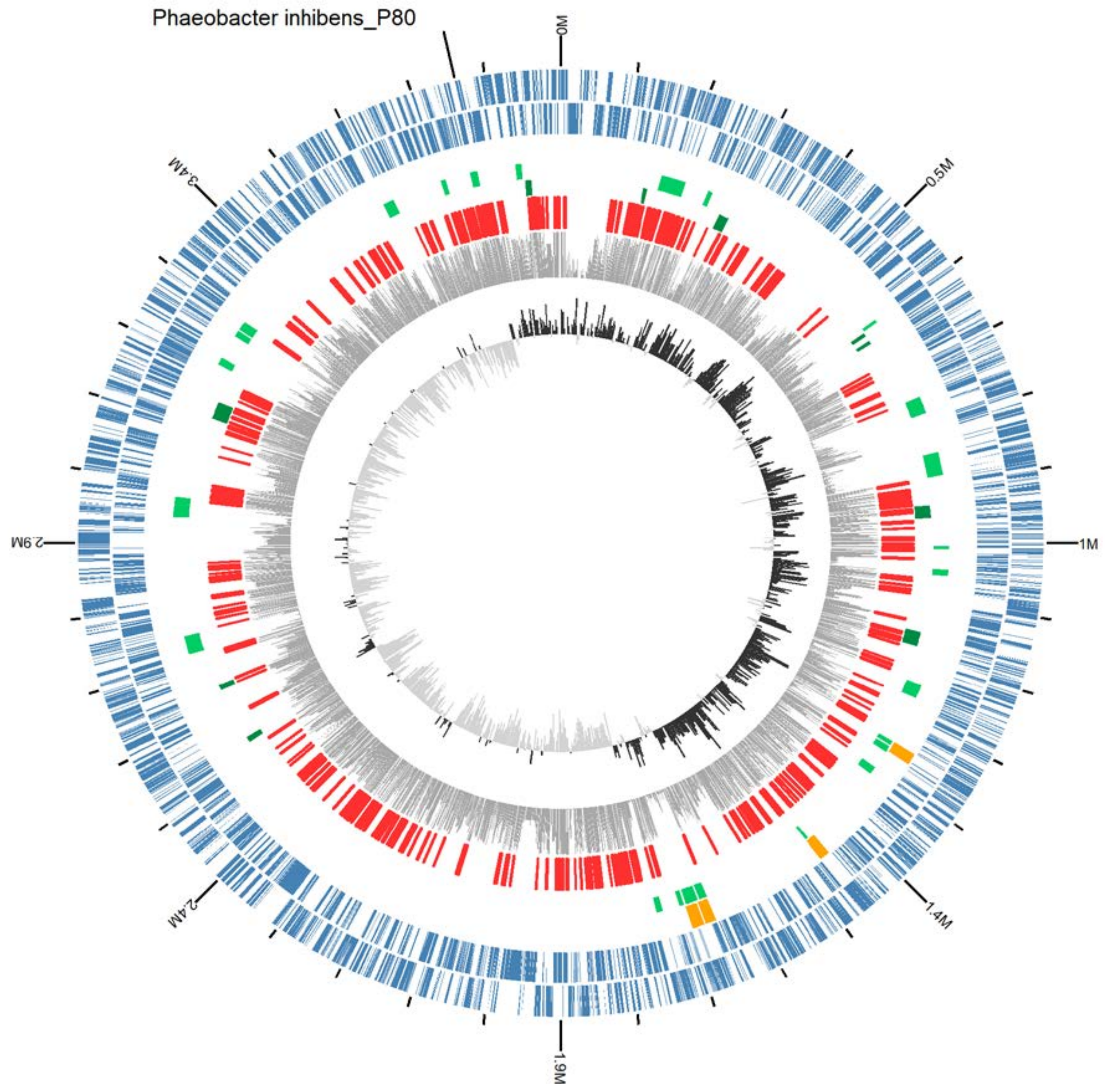

Phaeobacter inhibens_P80
0M
0.5M
1M
1.4M
1.9M
2.4M
2.9M
3.4M

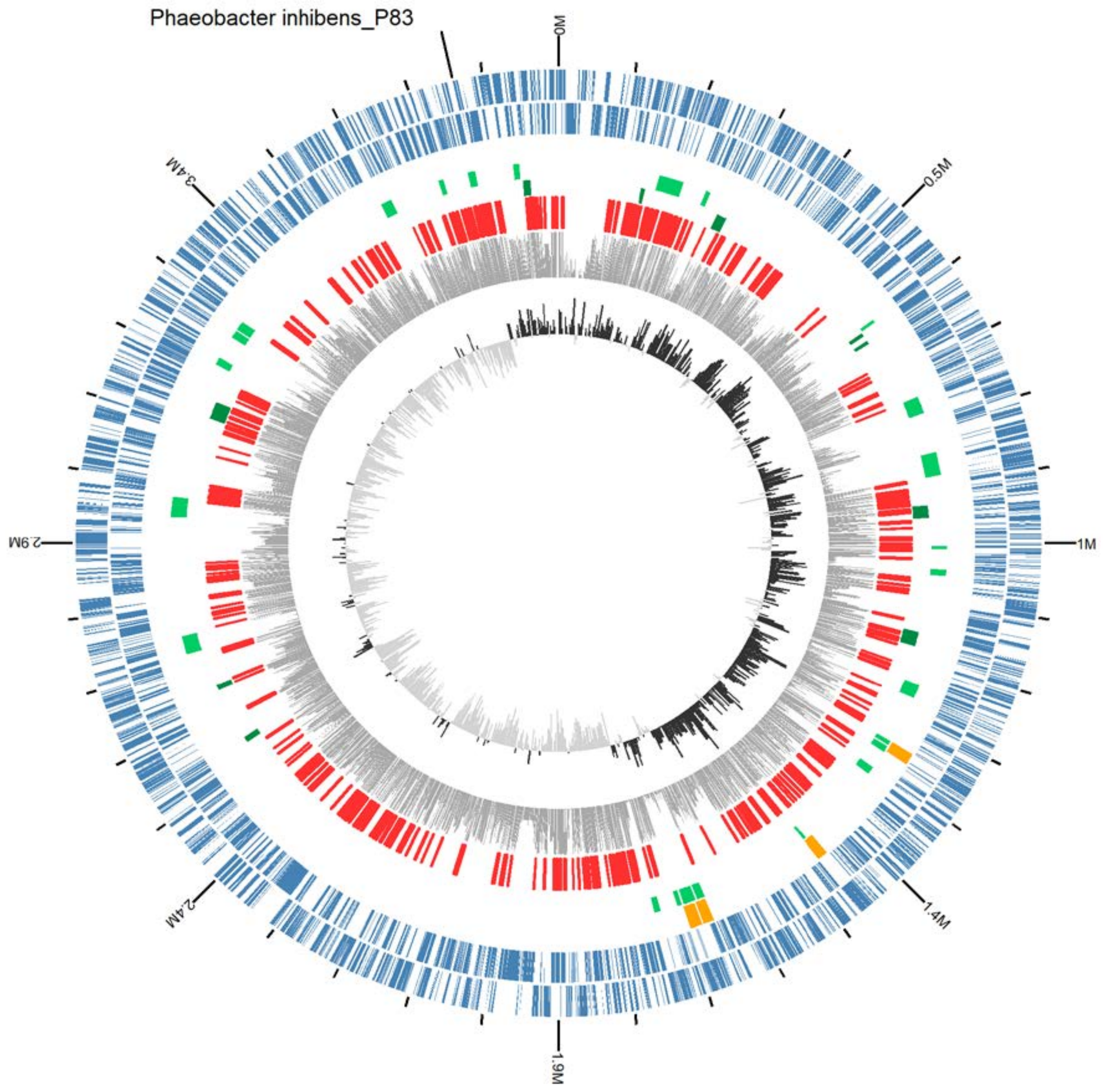
Phaeobacter inhibens_P83
0M
0.5M
1M
1.4M
1.9M
2.4M
2.9M
3.4M

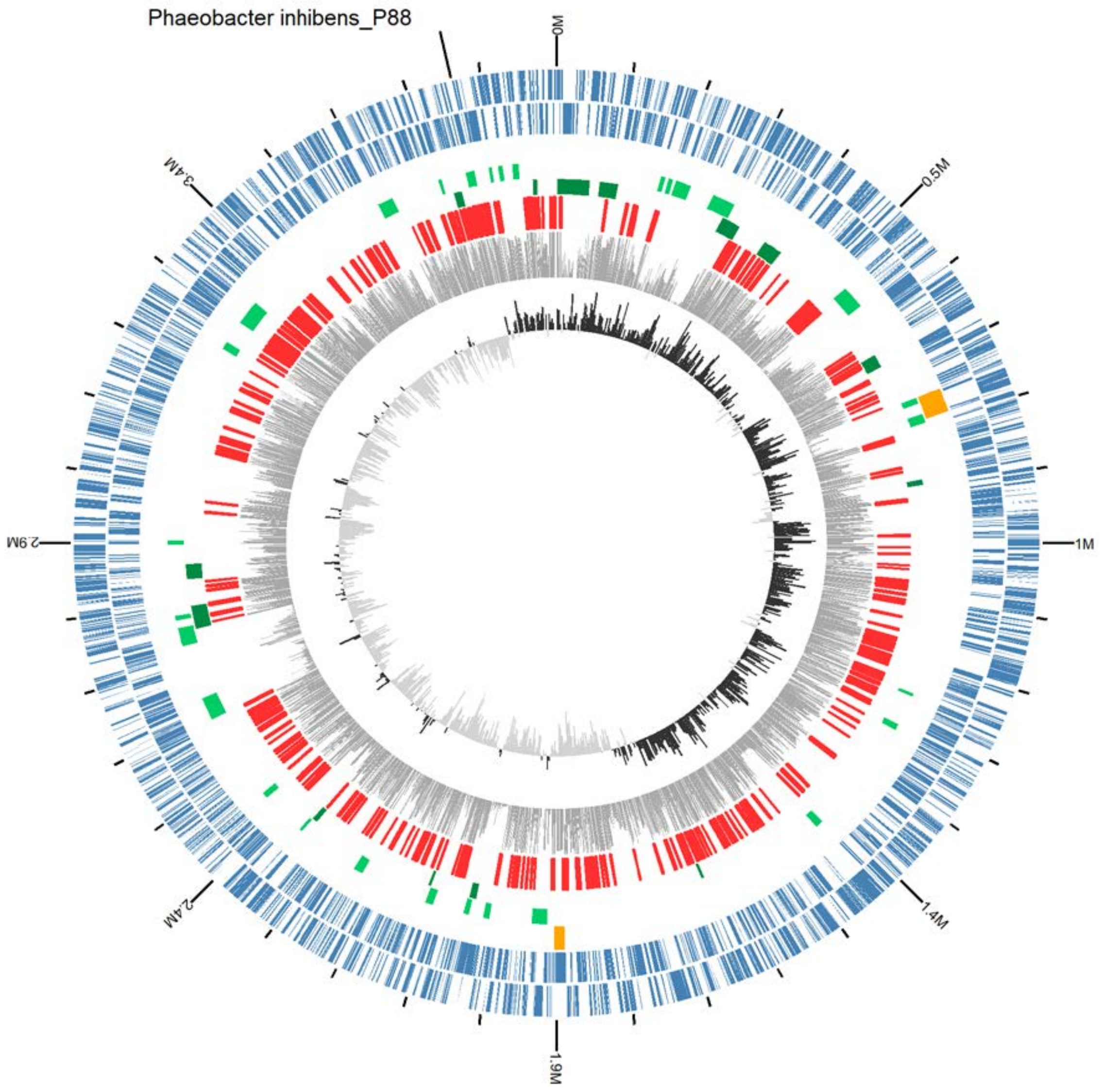
Phaeobacter inhibens_P88
0M
0.5M
1M
1.4M
1.9M
2.4M
2.9M
3.4M

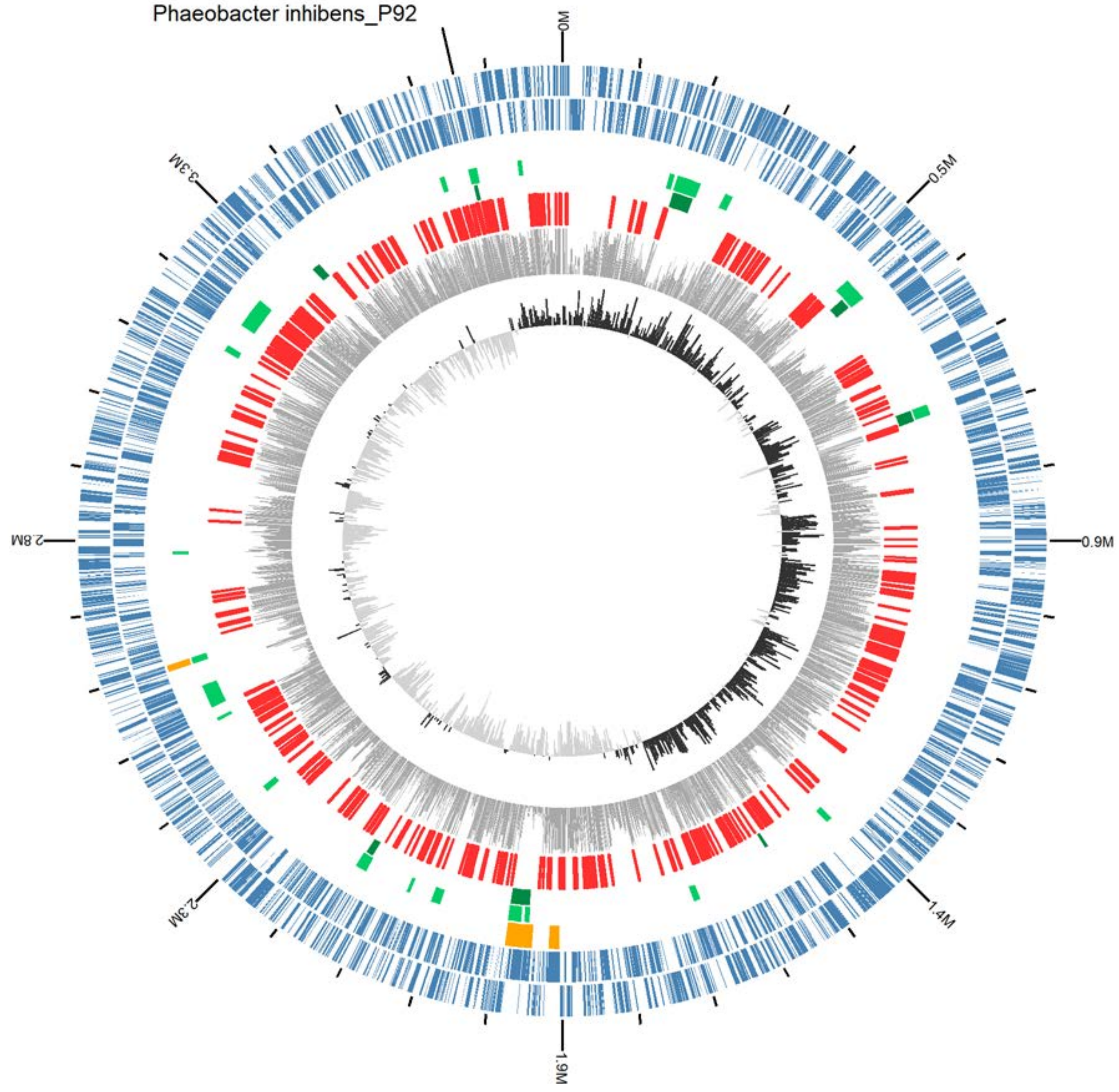
Phaeobacter inhibens_P92
0M
0.5M
0.9M
1.4M
1.9M
2.3M
2.8M
3.3M

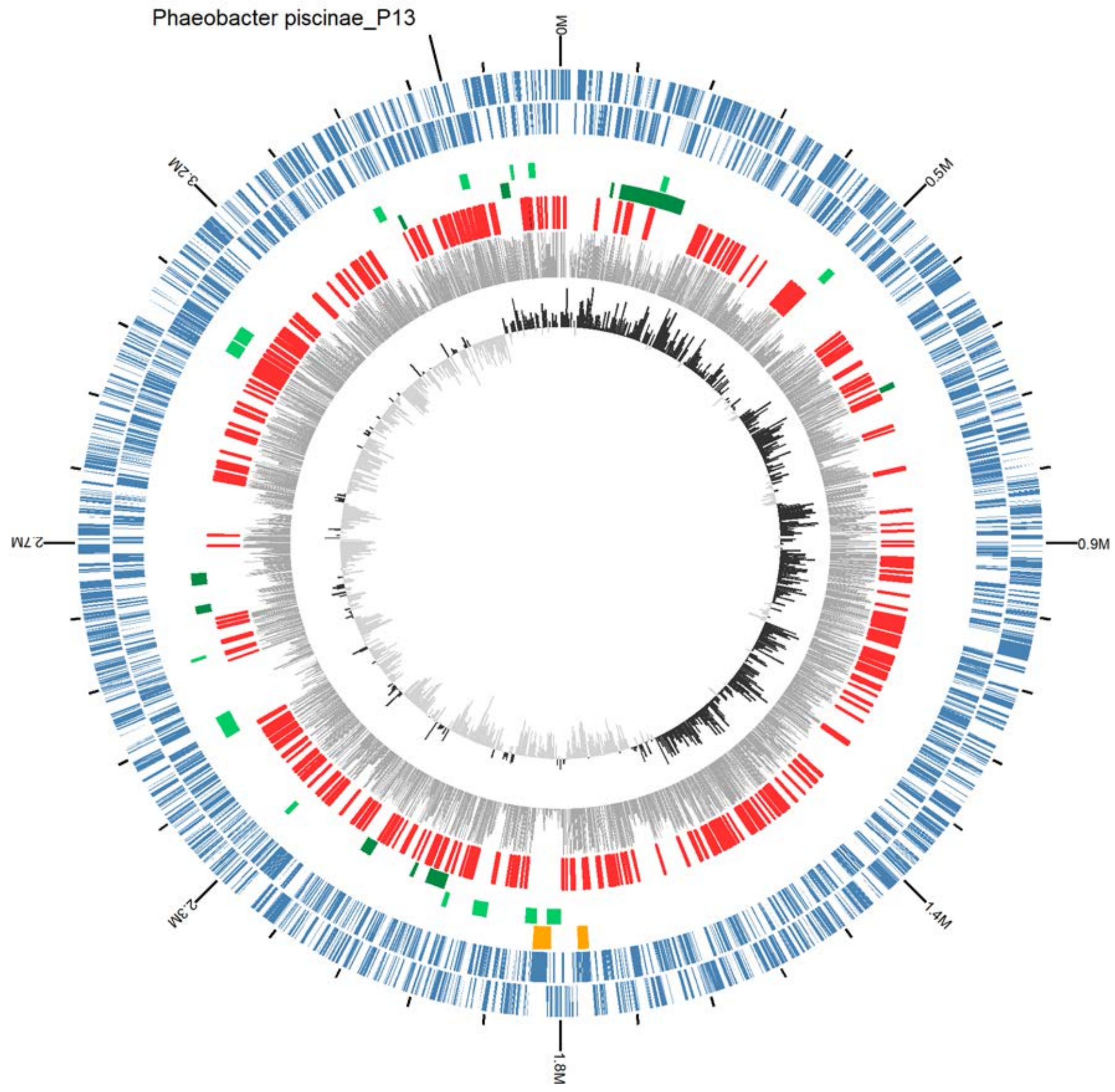

Phaeobacter piscinae_P13
0M
0.5M
0.9M
1.4M
1.8M
2.3M
2.7M
3.2M

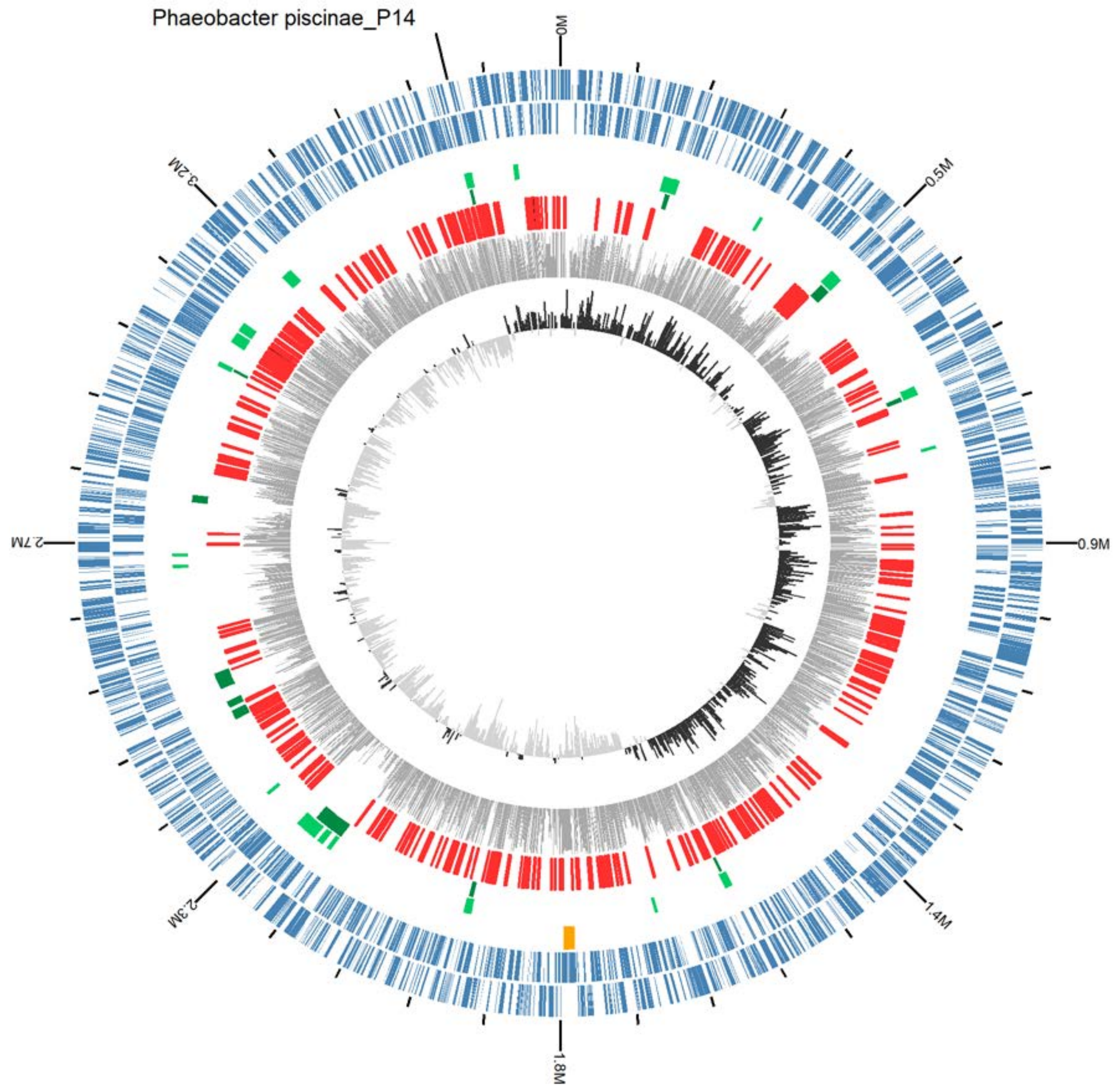

Phaeobacter piscinae_P14
0M
0.5M
0.9M
1.4M
1.8M
2.3M
2.7M
3.2M

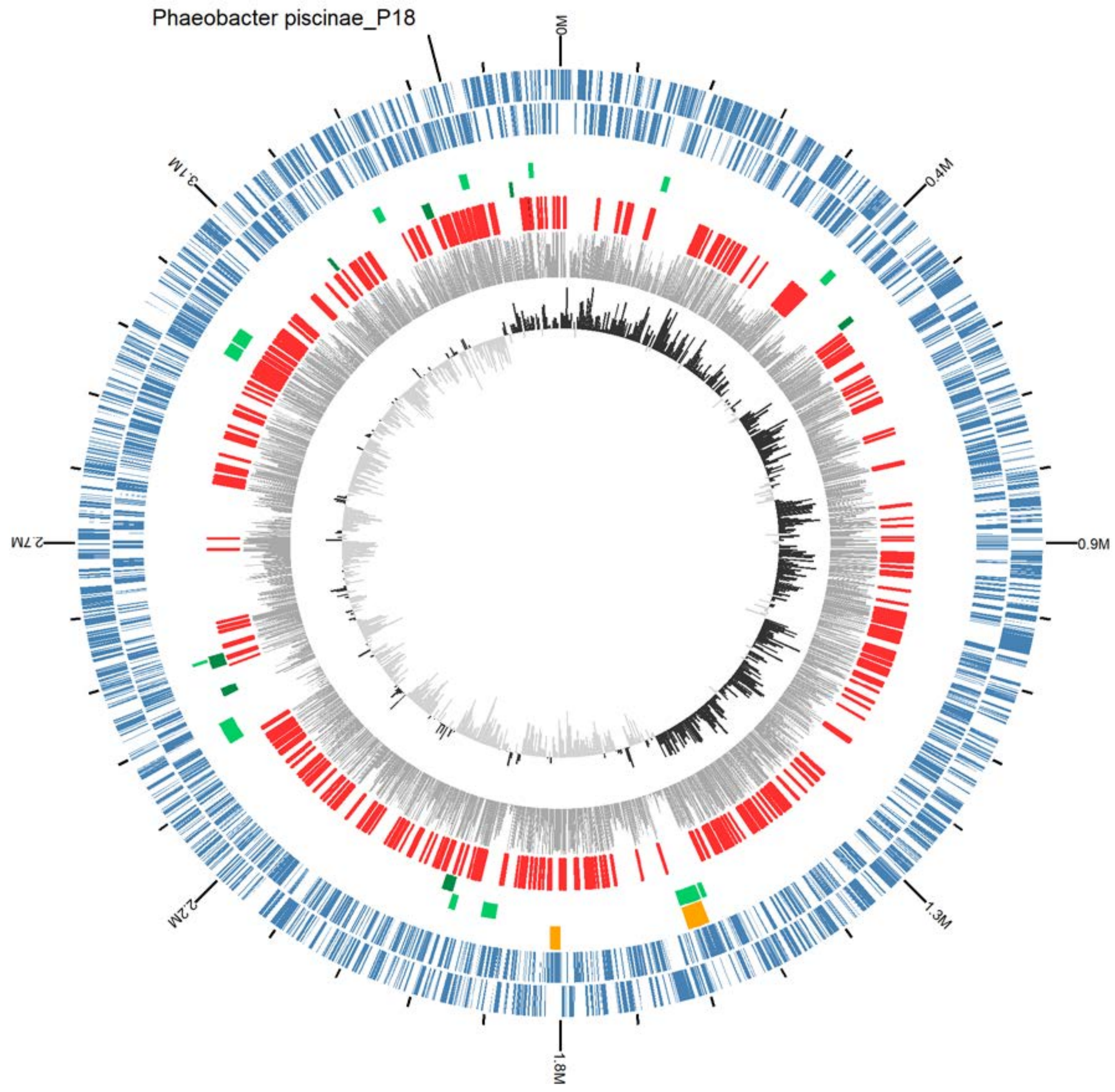

Phaeobacter piscinae_P18
0M
0.4M
0.9M
1.3M
1.8M
2.2M
2.7M
3.1M

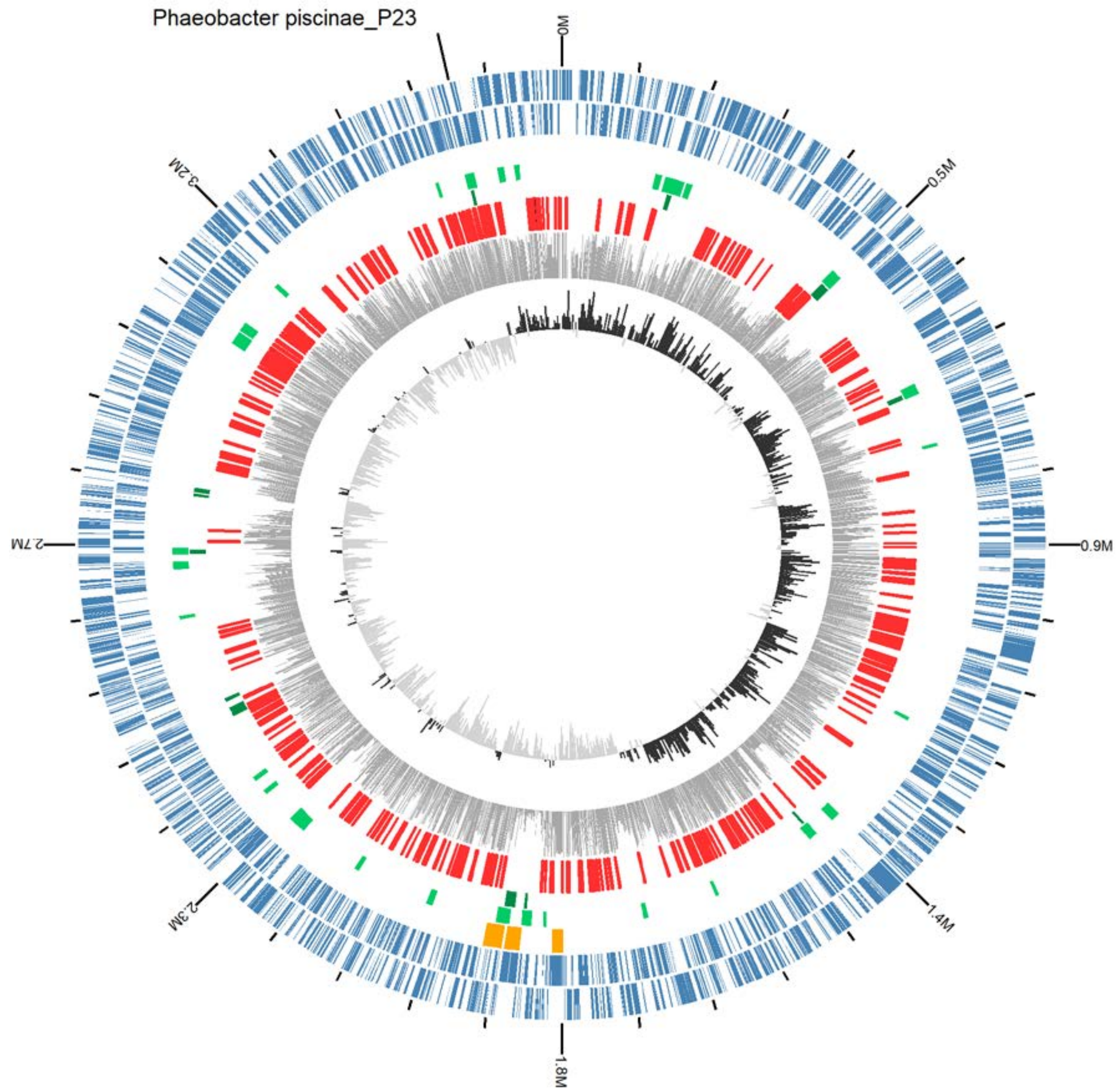

Phaeobacter piscinae_P23
0M
0.5M
0.9M
1.4M
1.8M
2.3M
2.7M
3.2M

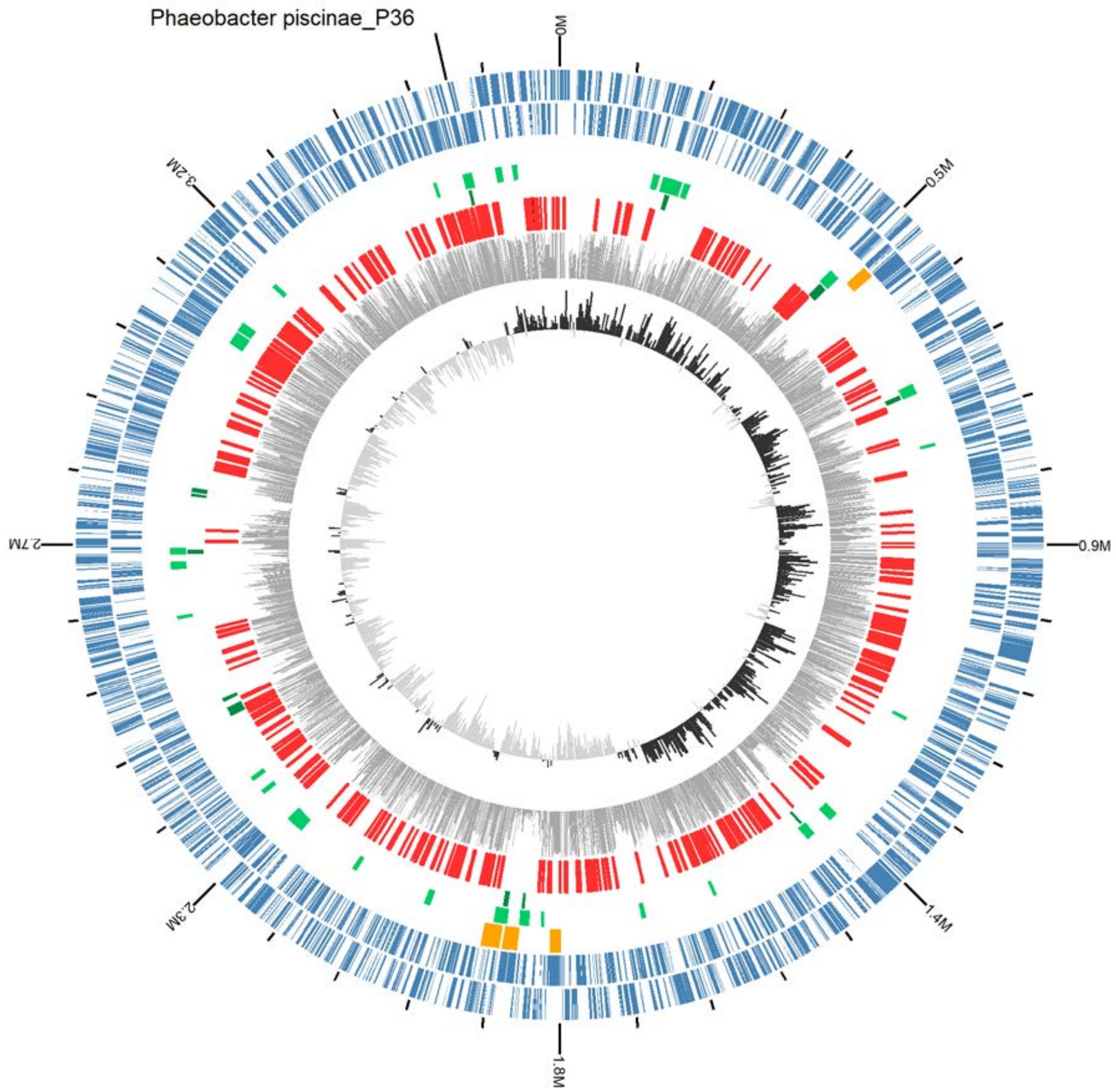

Phaeobacter piscinae_P36
0M
0.5M
0.9M
1.4M
1.8M
2.3M
2.7M
3.2M

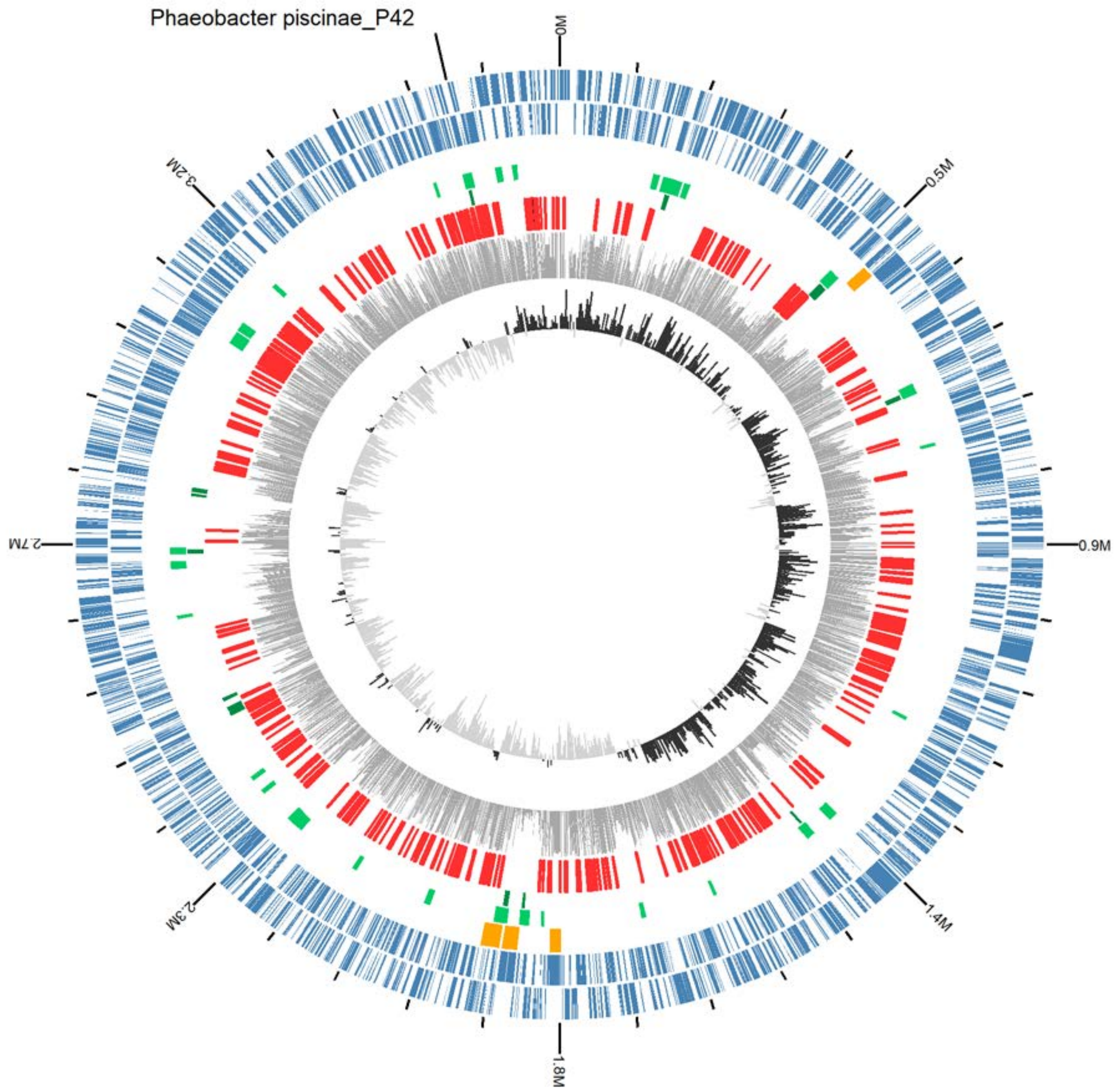

Phaeobacter piscinae_P42
0M
0.5M
0.9M
1.4M
1.8M
2.3M
2.7M
3.2M

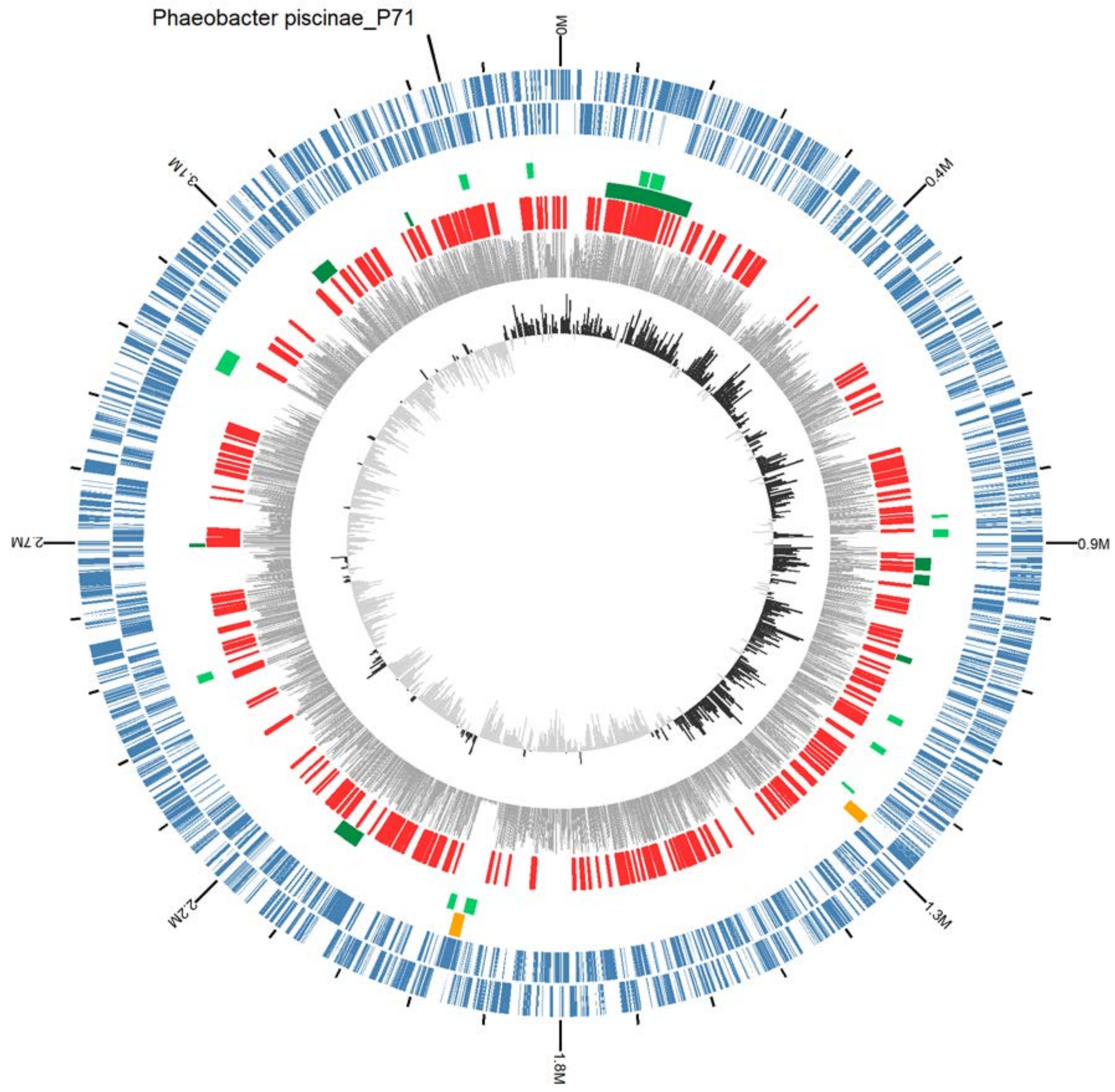

Phaeobacter piscinae_P71
0M
0.4M
0.9M
1.3M
1.8M
2.2M
2.7M
3.1M

Phaeobacter porticola_P97

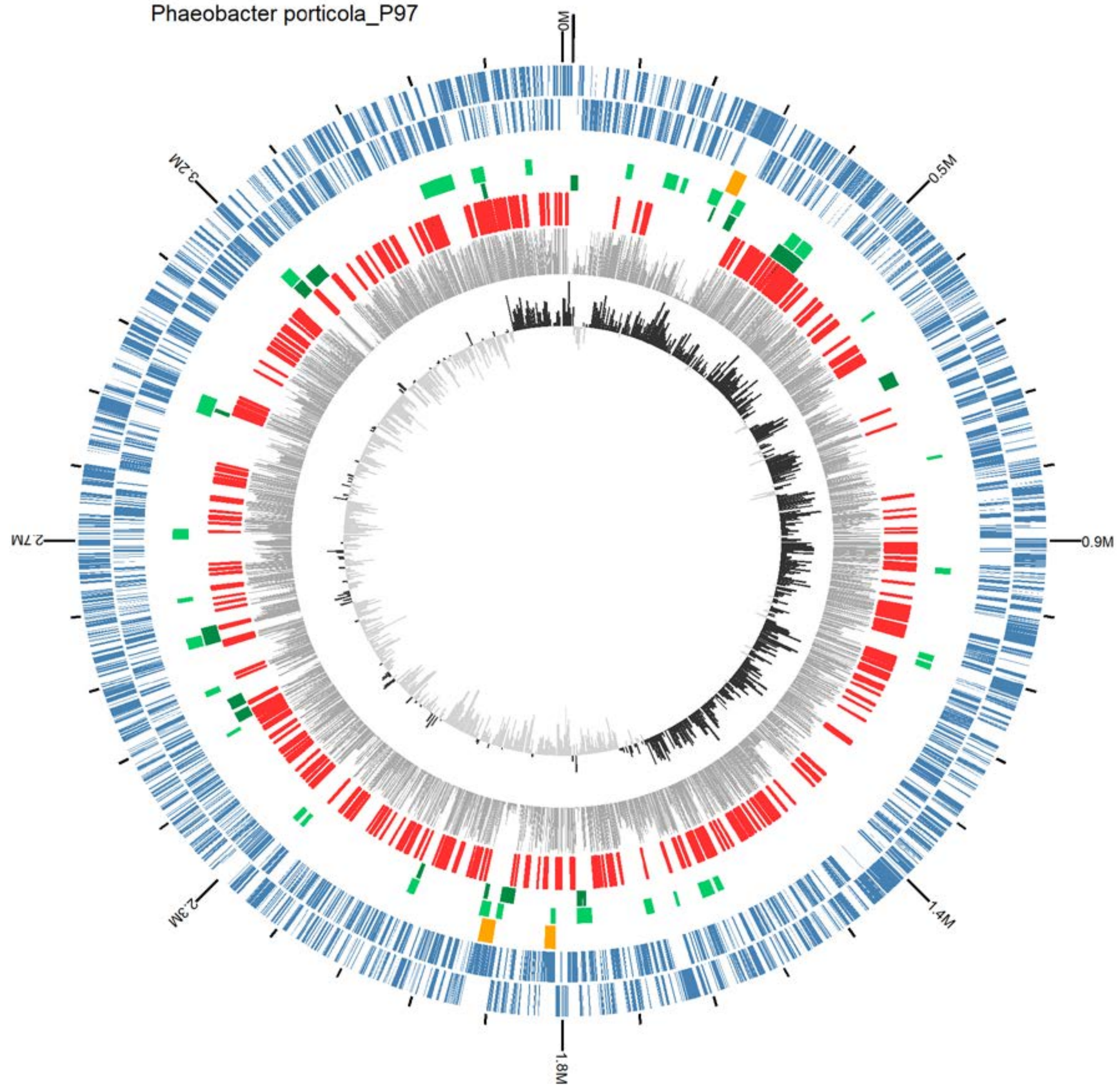

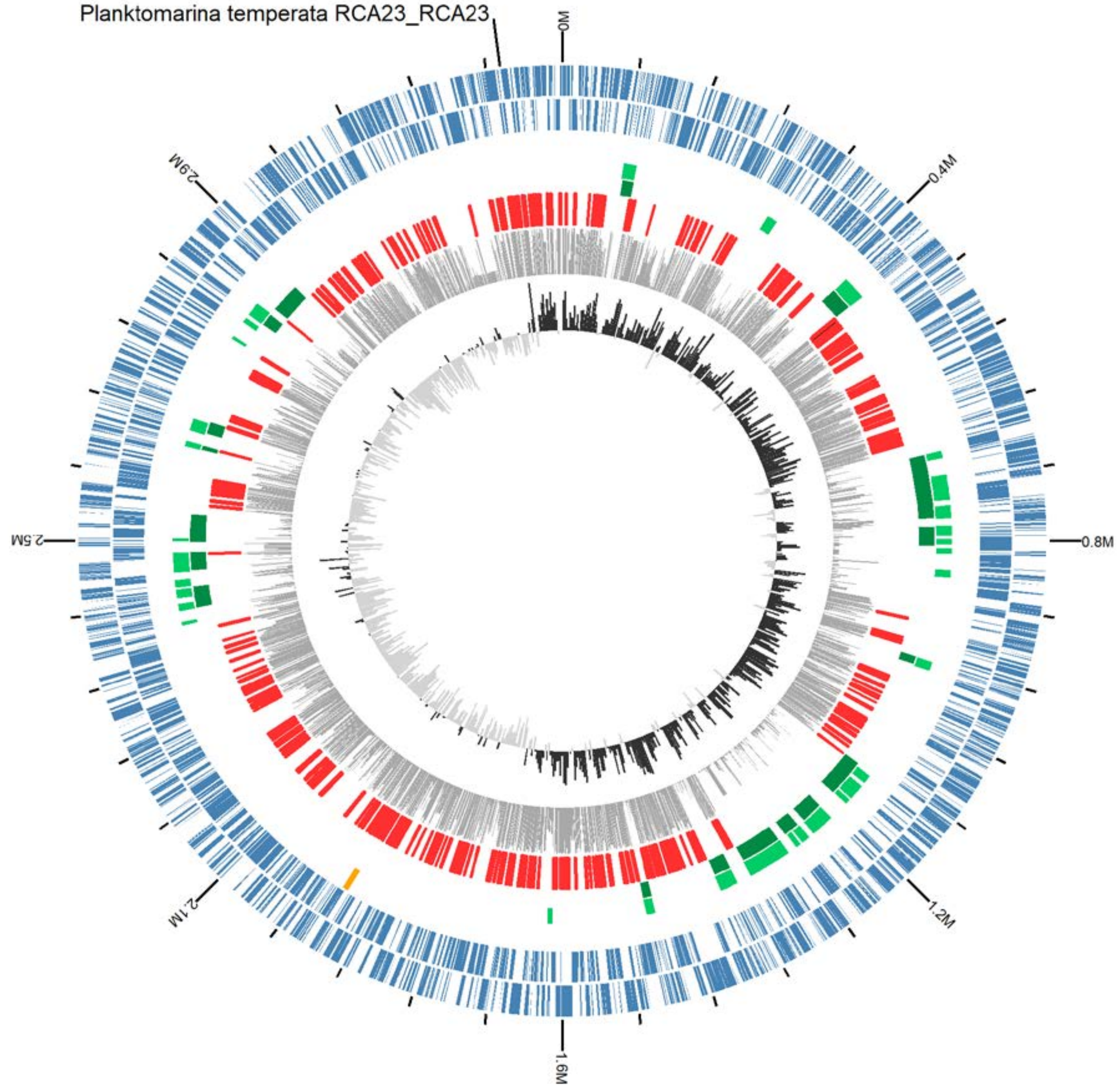
Planktomarina temperata RCA23_RCA23
0M
0.4M
0.8M
1.2M
1.6M
2.1M
2.5M
2.9M

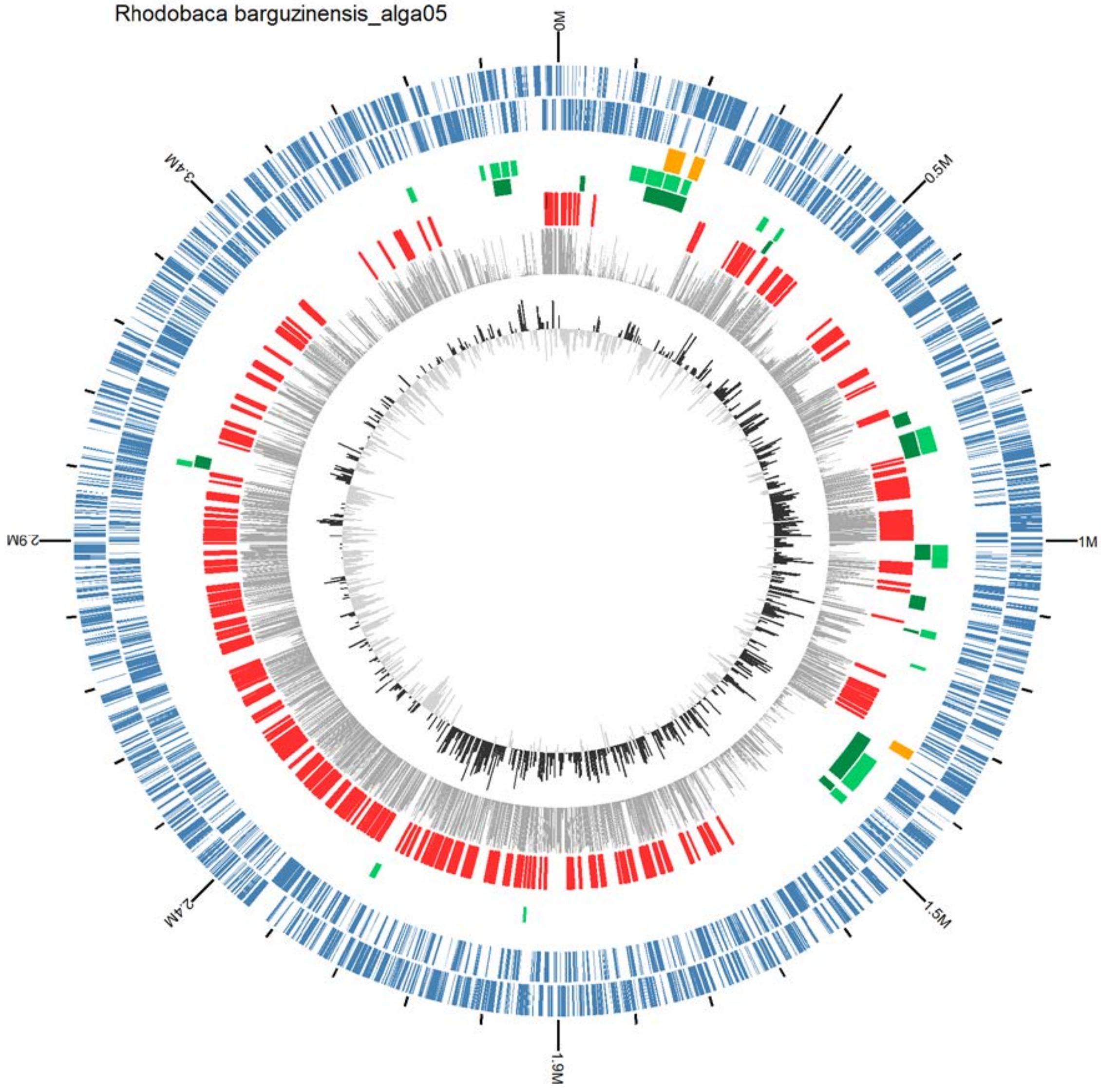
Rhodobaca barguzinensis_alga05
0M
0.5M
1M
1.5M
1.9M
2.4M
2.9M
3.4M

Rhodobacter capsulatus SB 1003_SB 1003

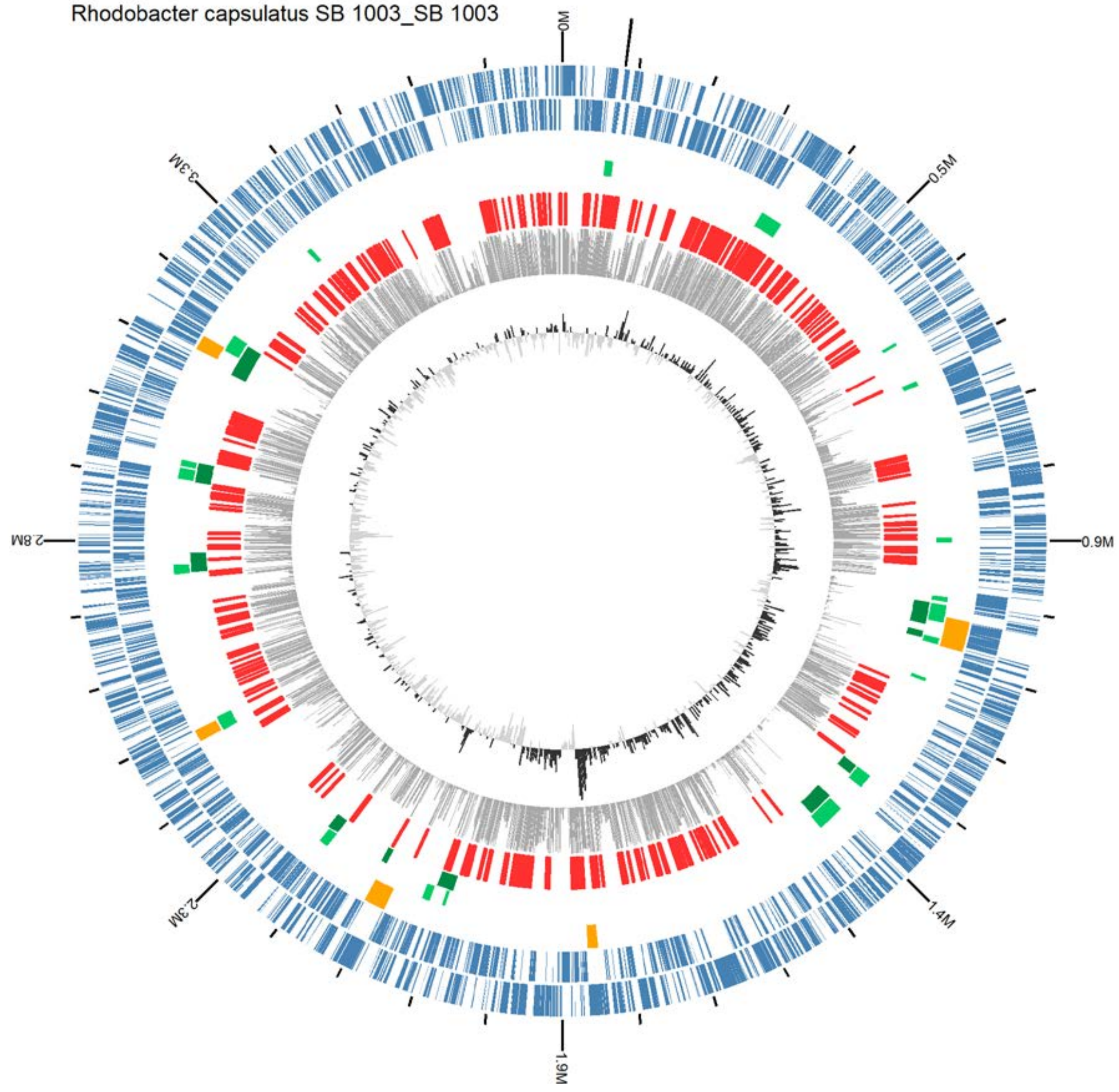

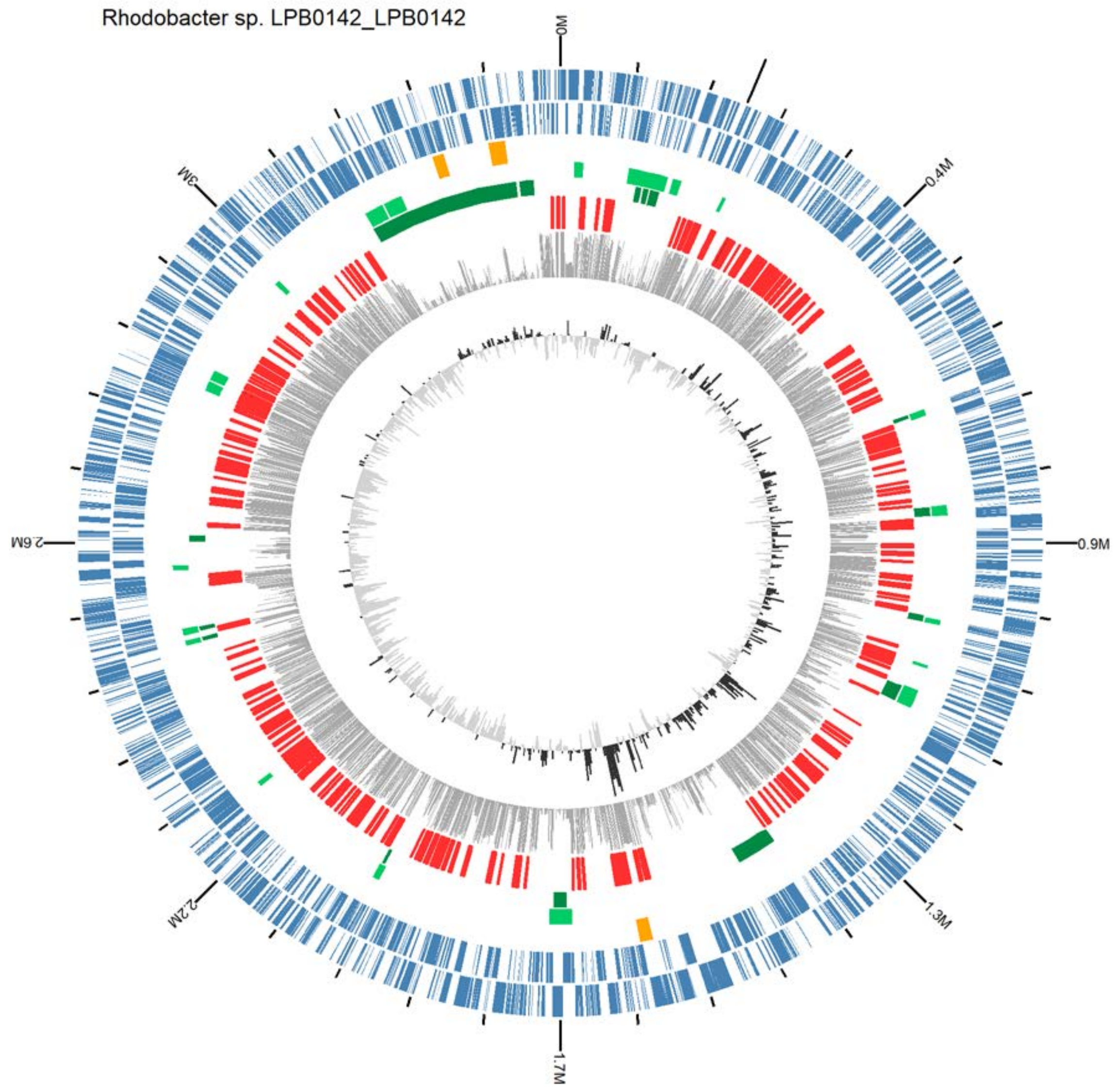
Rhodobacter sp. LPB0142_LPB0142
0M
0.4M
0.9M
1.3M
1.7M
2.2M
2.6M
3M

Rhodobacter sphaeroides ATCC 17025_ATCC 17025

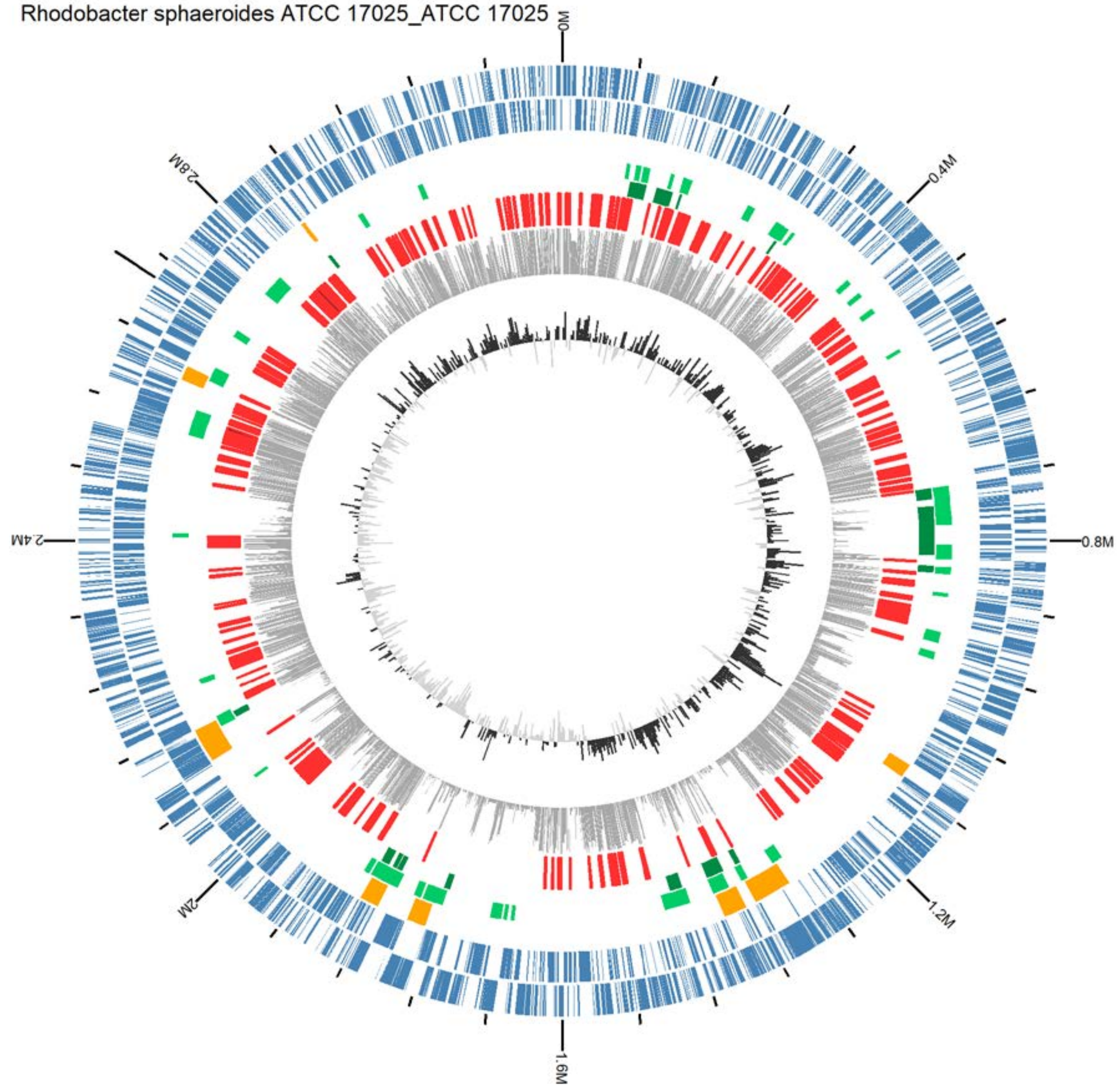

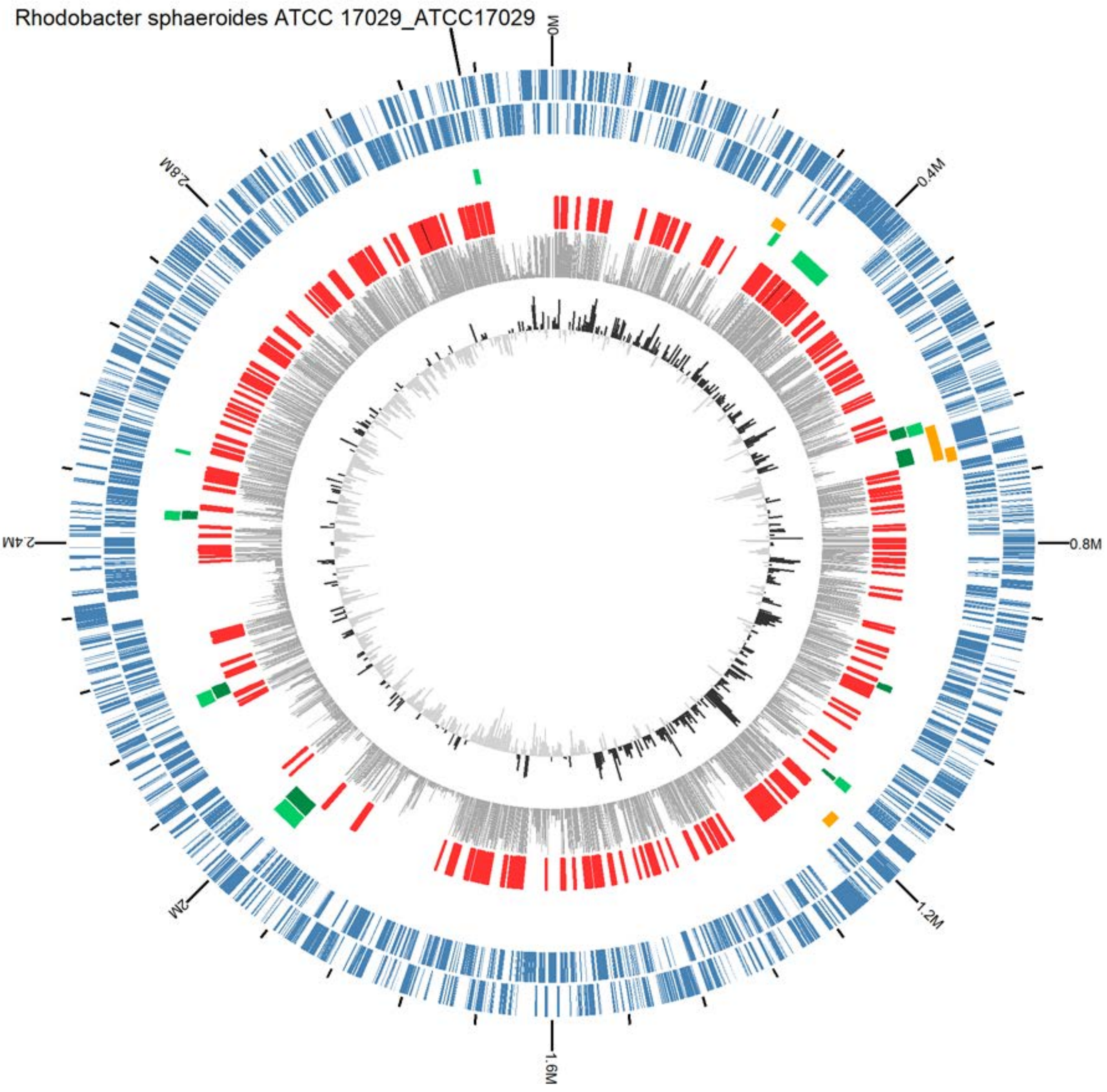

Rhodobacter sphaeroides ATCC 17029_ATCC17029
0M
0.4M
0.8M
1.2M
1.6M
2M
2.4M
2.8M

Rhodobacter sphaeroides KD131_KD131; KCTC 12085

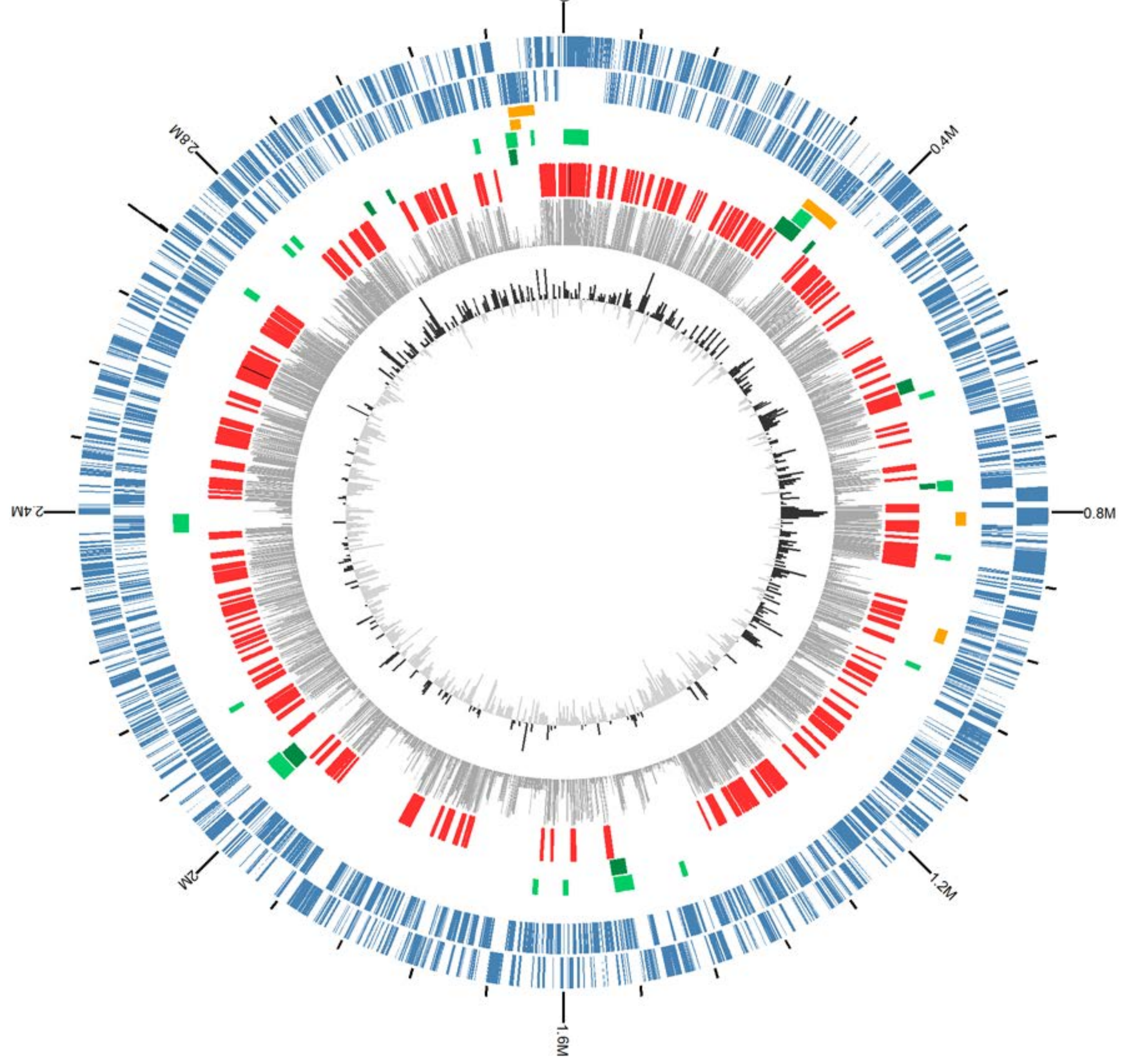

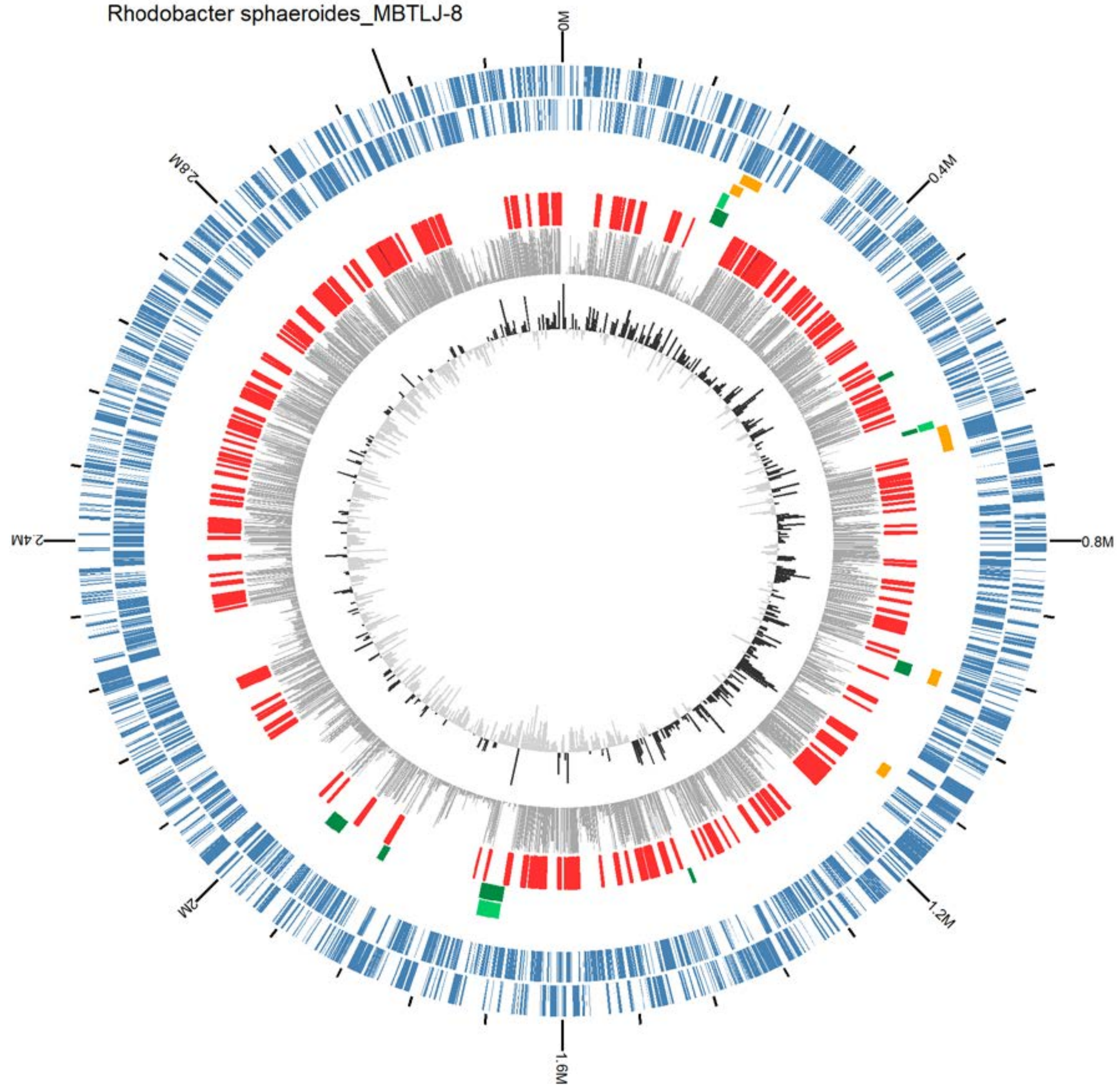
Rhodobacter sphaeroides_MBTLJ-8
0M
0.4M
0.8M
1.2M
1.6M
2M
2.4M
2.8M

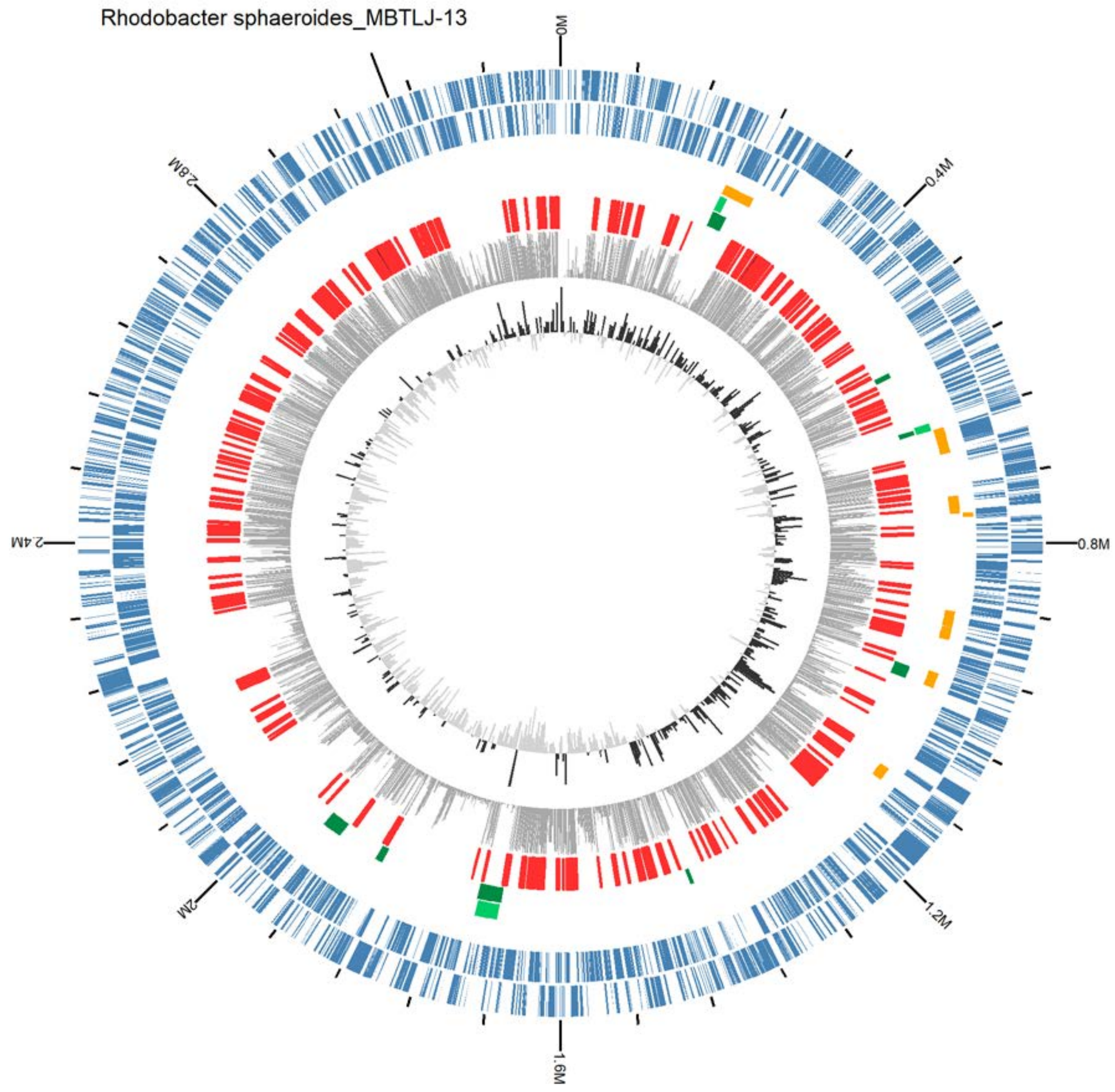
Rhodobacter sphaeroides_MBTLJ-13
0M
0.4M
0.8M
1.2M
1.6M
2M
2.4M
2.8M

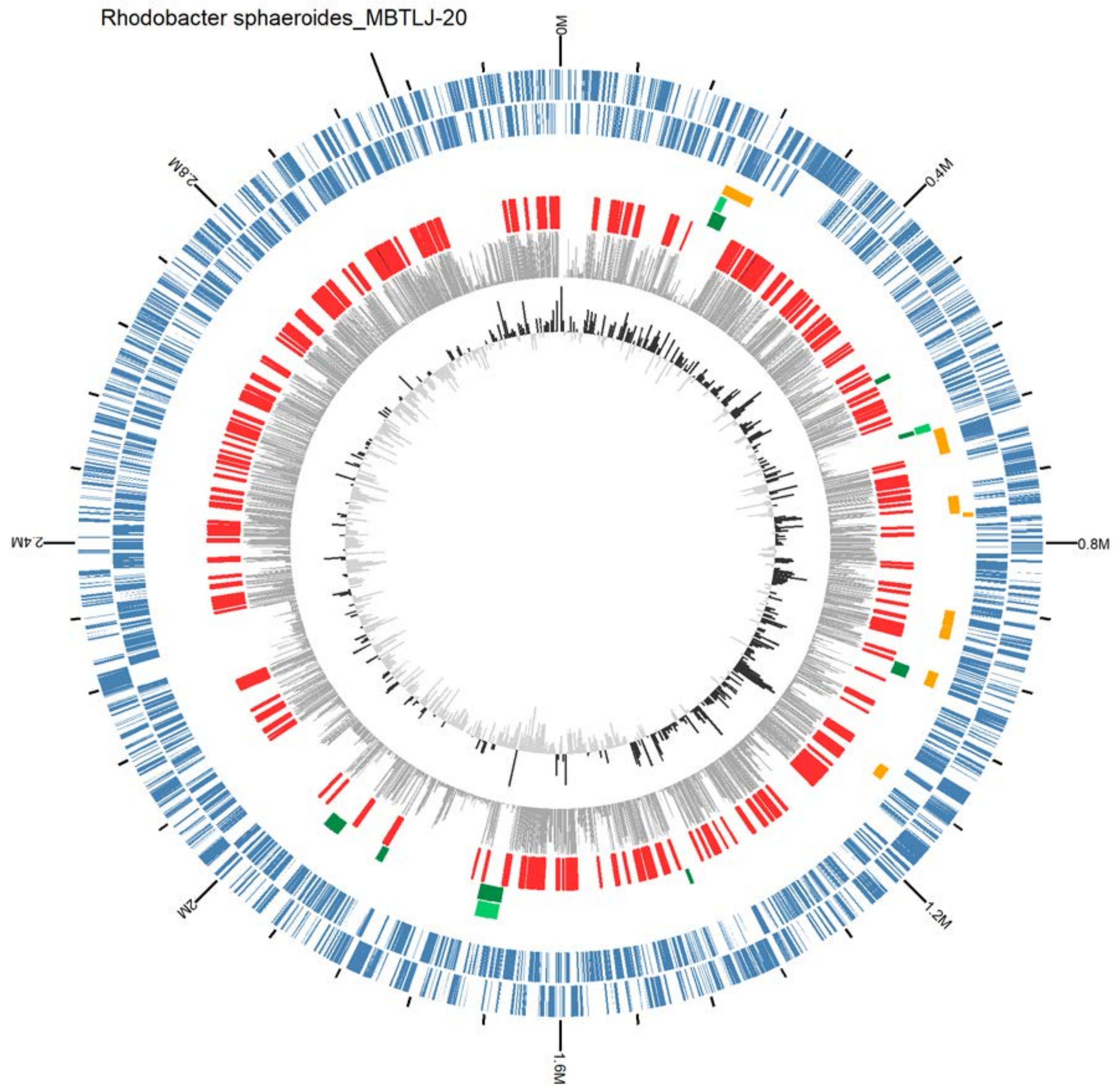
Rhodobacter sphaeroides_MBTLJ-20
0M
0.4M
0.8M
1.2M
1.6M
2M
2.4M
2.8M

Rhodobacter sphaeroides_org2181

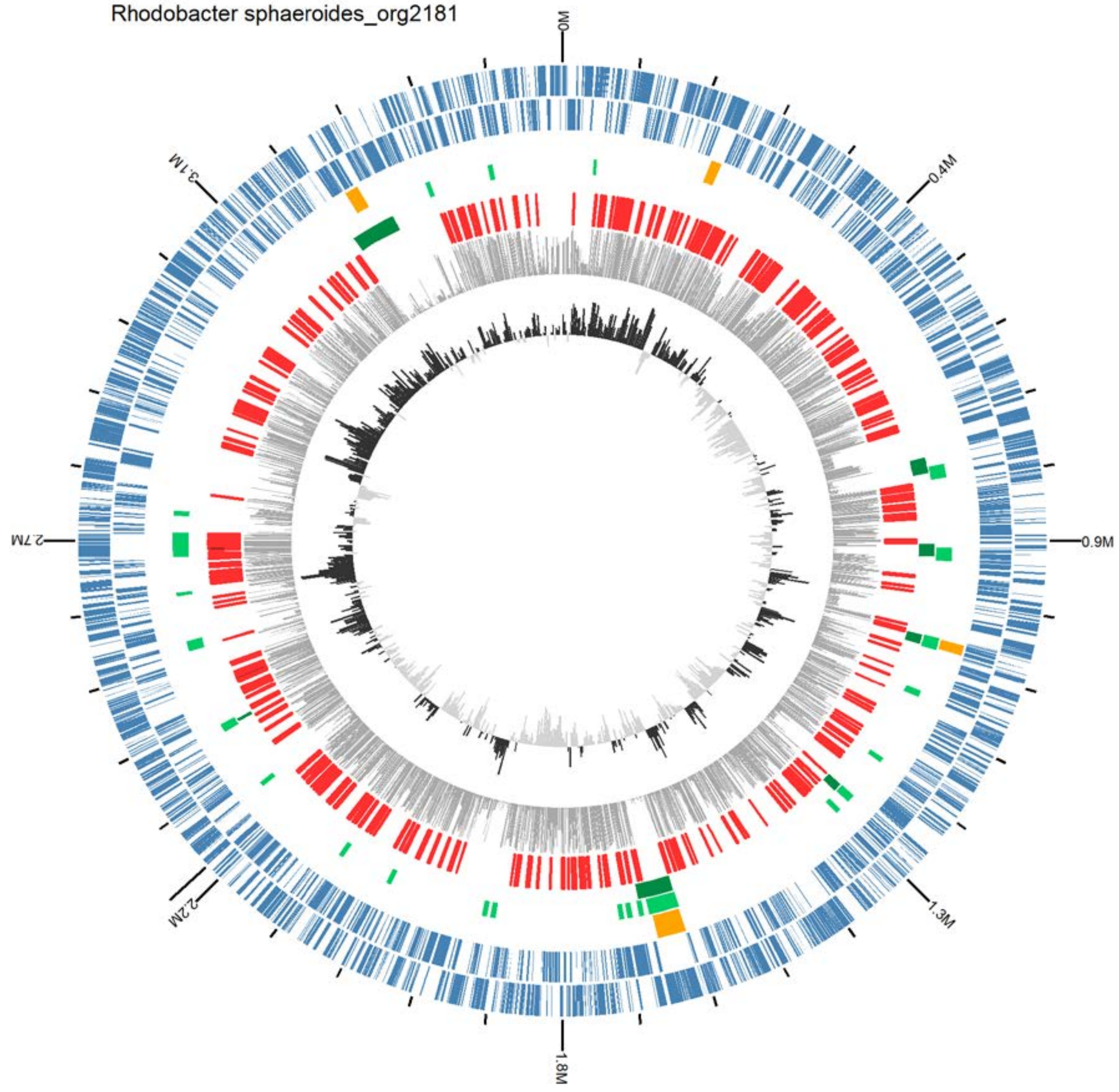

Rhodobacteraceae bacterium_G7

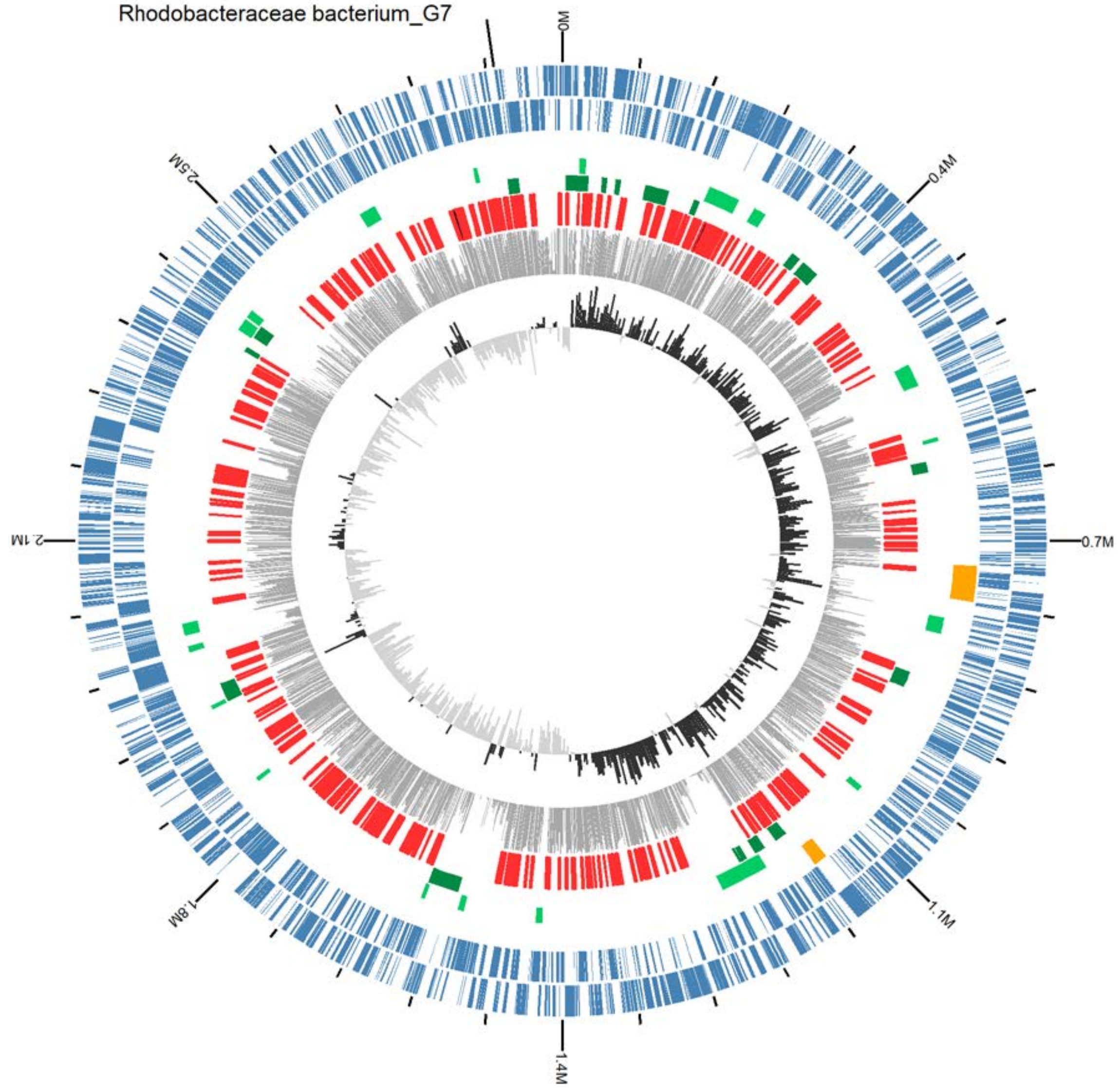

Rhodovulum sp. MB263_MB263

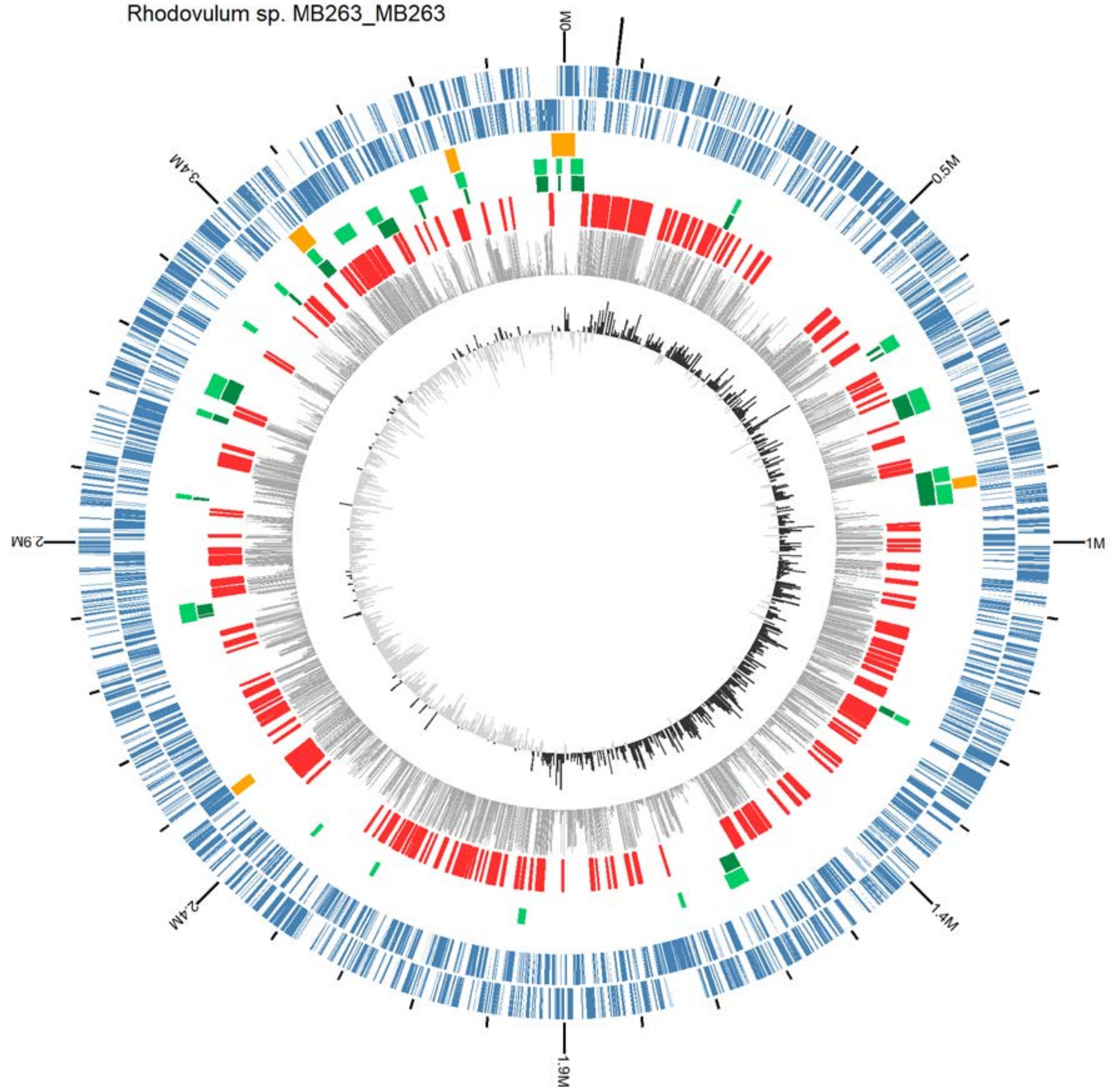

Rhodovulum sp. P5_P5

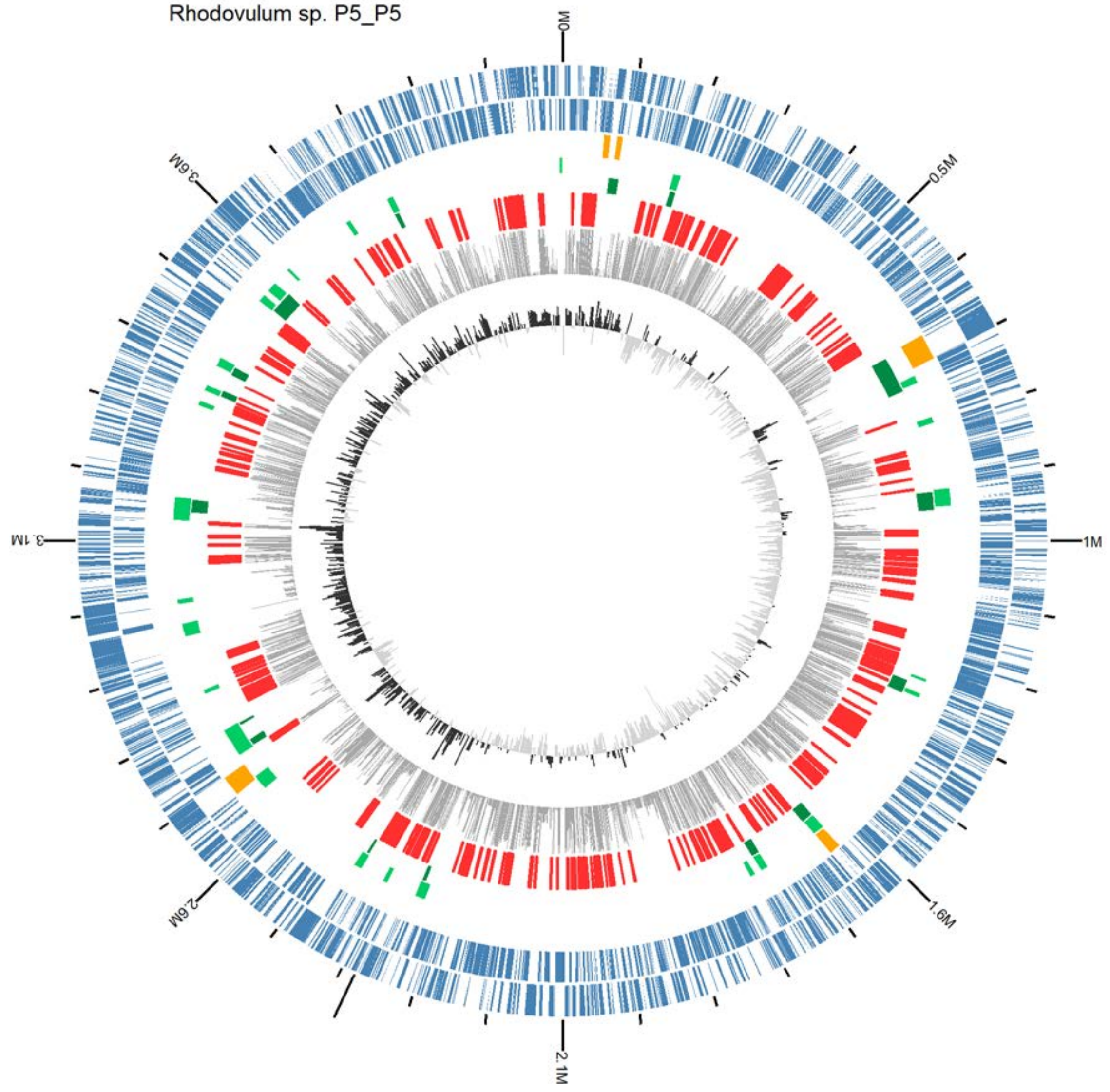

Rhodovulum sulfidophilum DSM 1374_DSM 1374

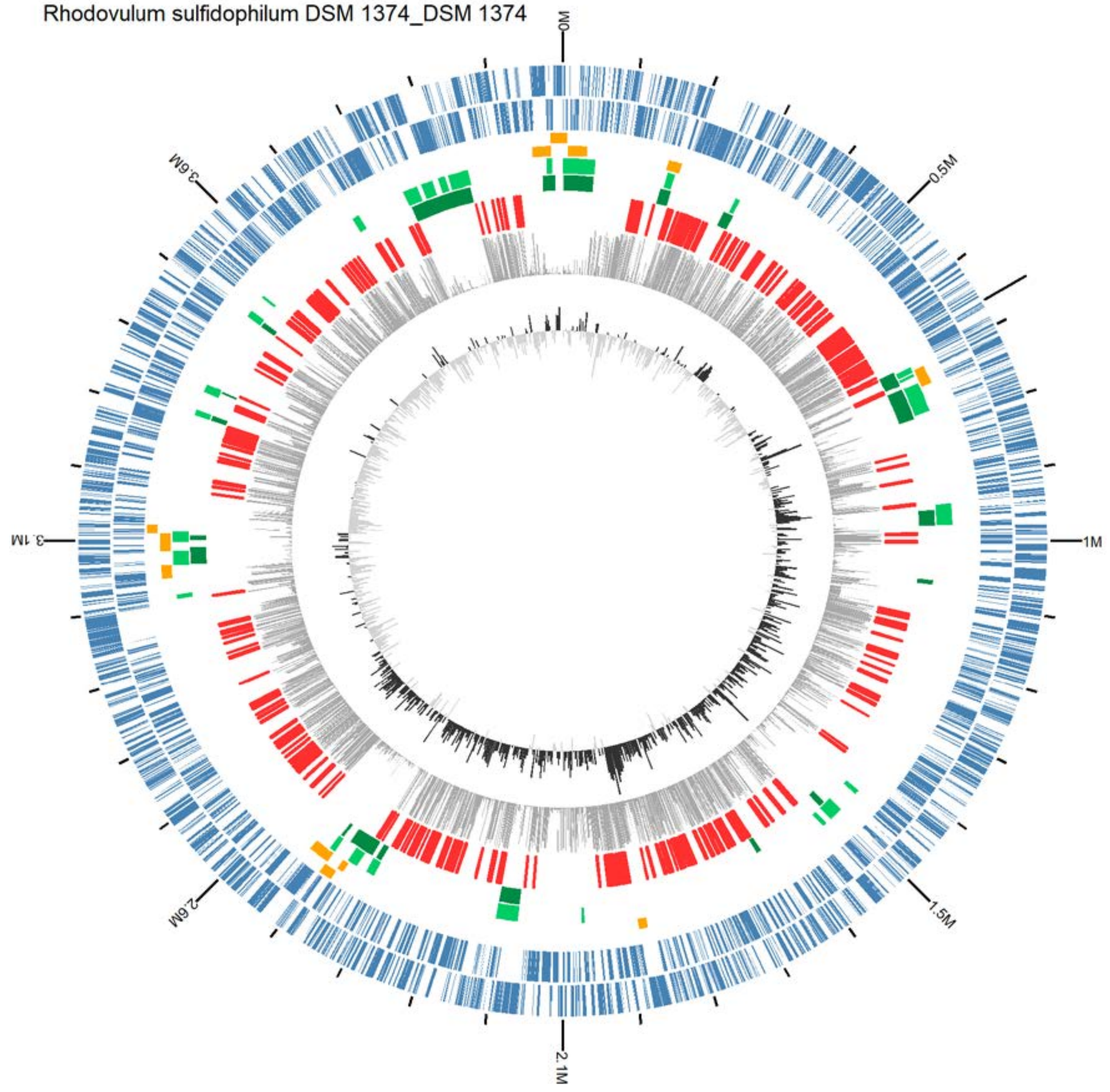

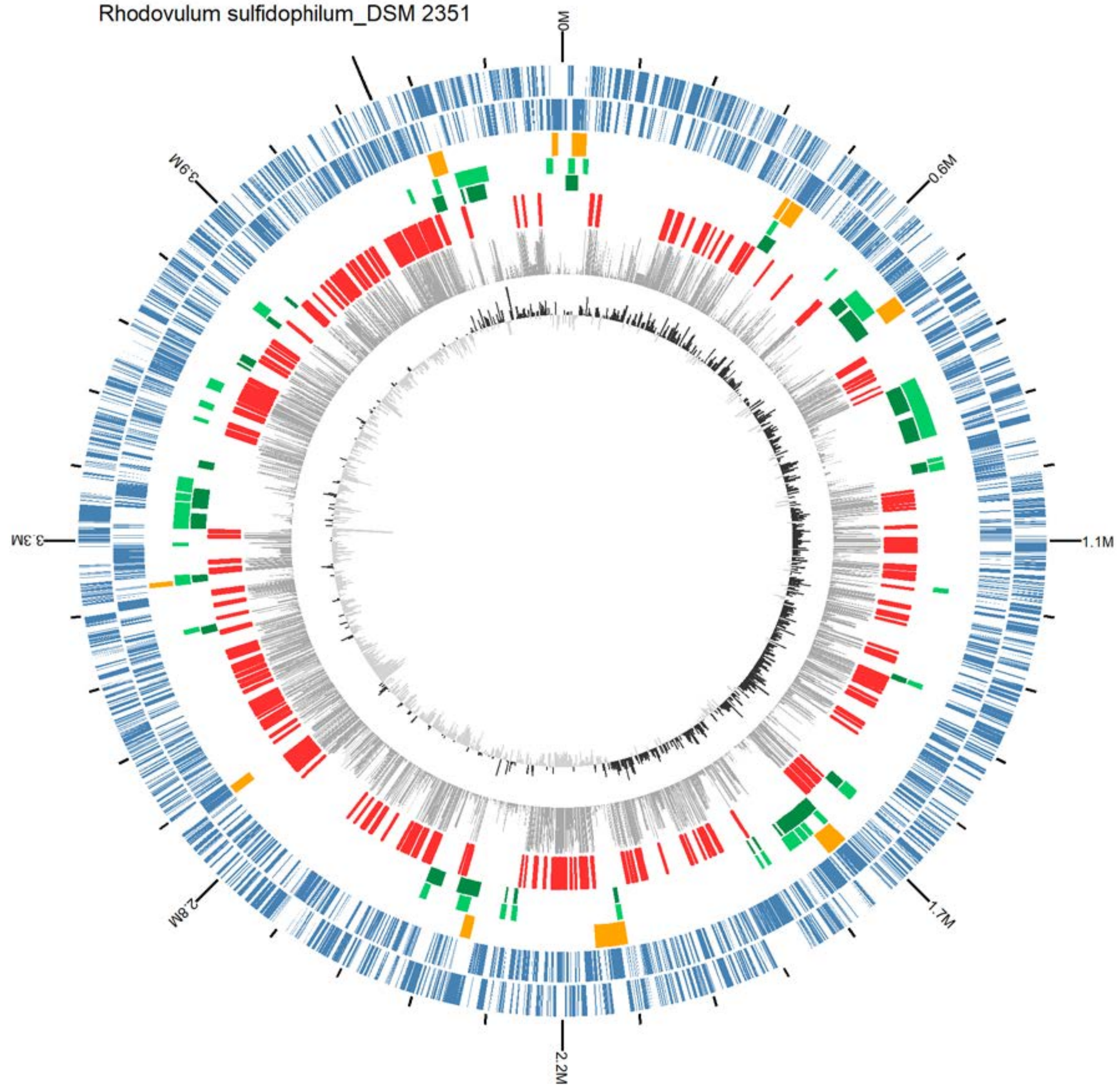

Rhodovulum sulfidophilum_DSM 2351
0M
0.6M
1.1M
1.7M
2.2M
2.8M
3.3M
3.9M

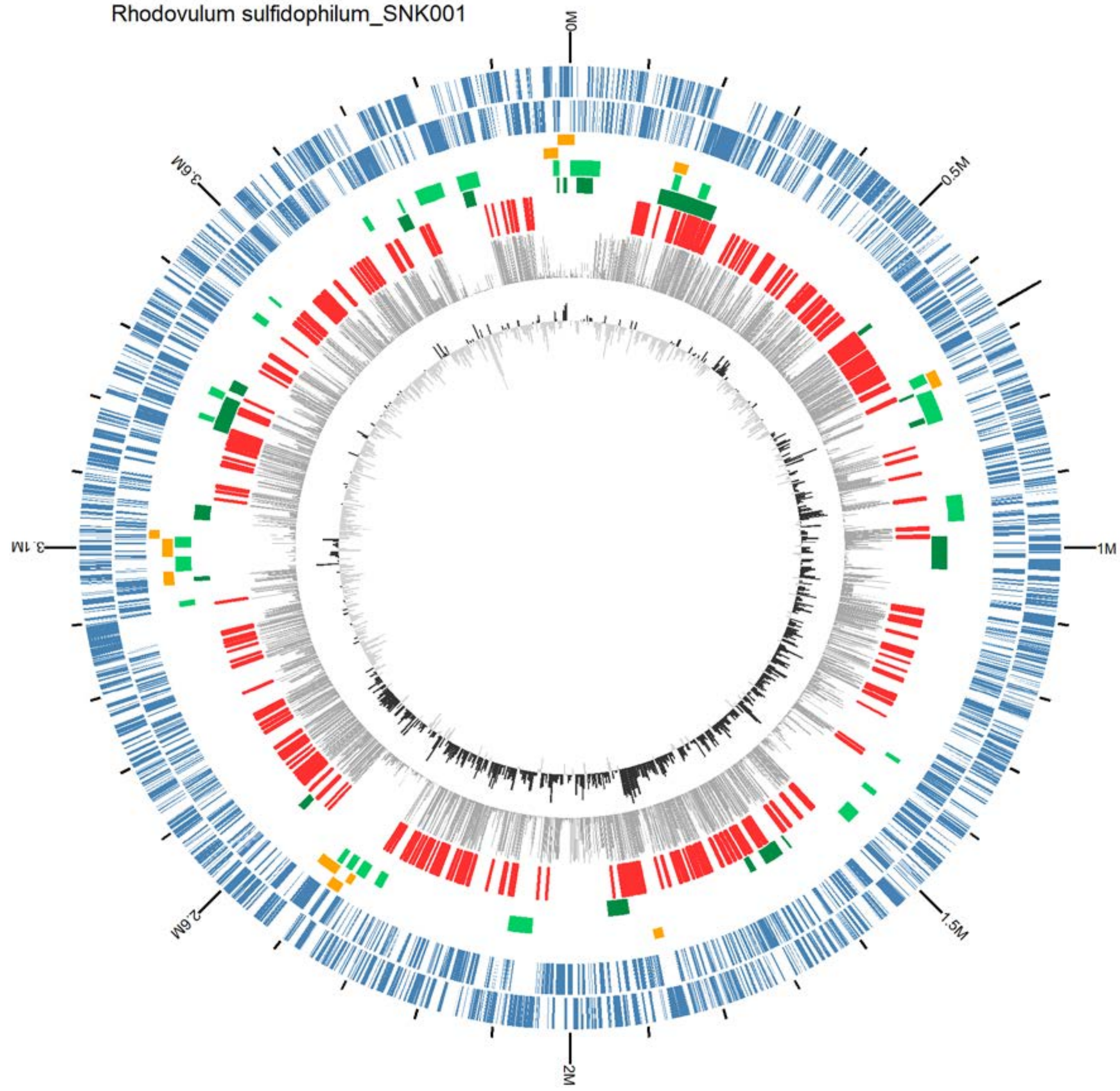
Rhodovulum sulfidophilum_SNK001
0M
0.5M
1M
1.5M
2M
2.6M
3.1M
3.6M

Roseibacterium elongatum DSM 19469_DFL-43

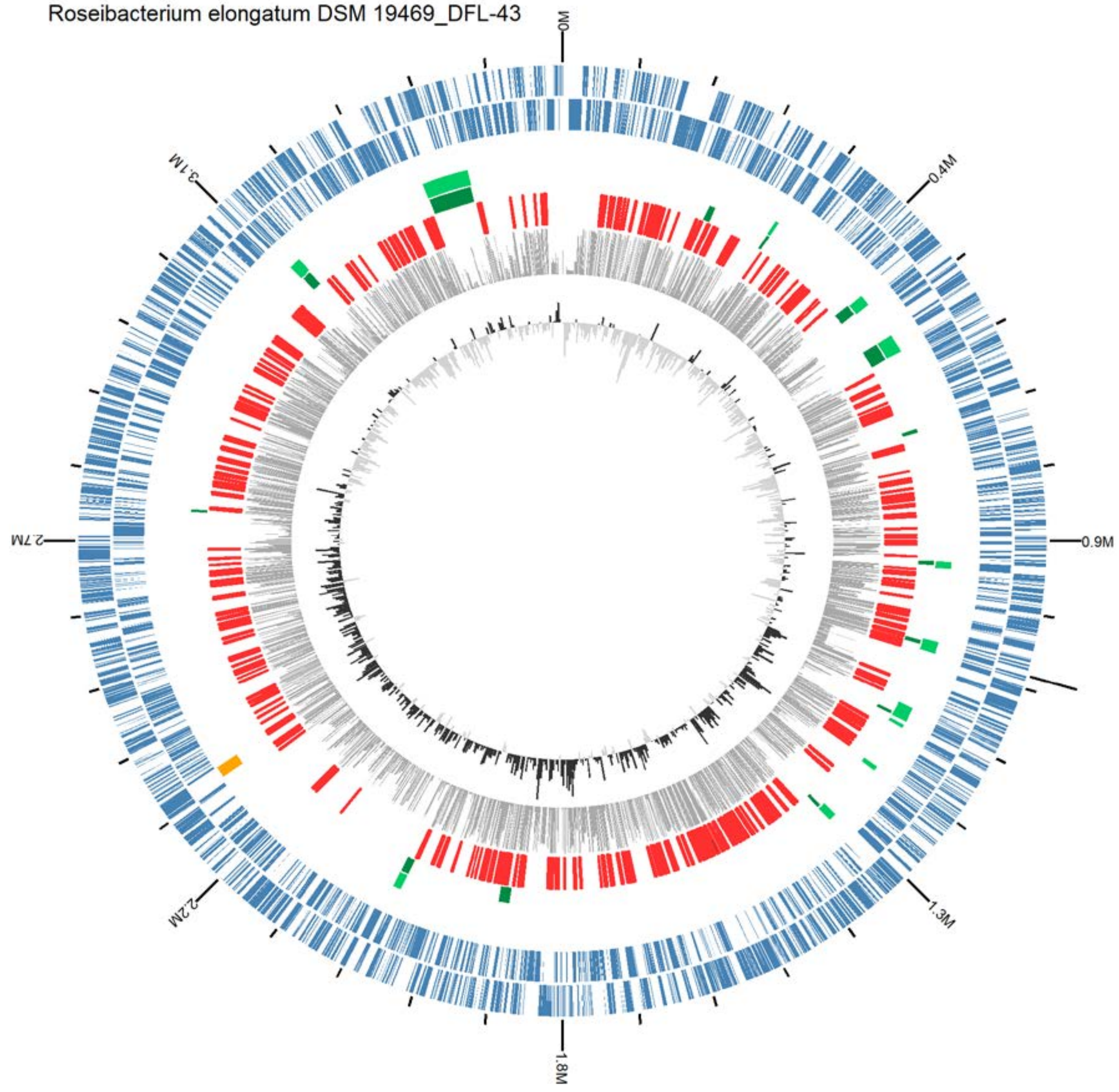

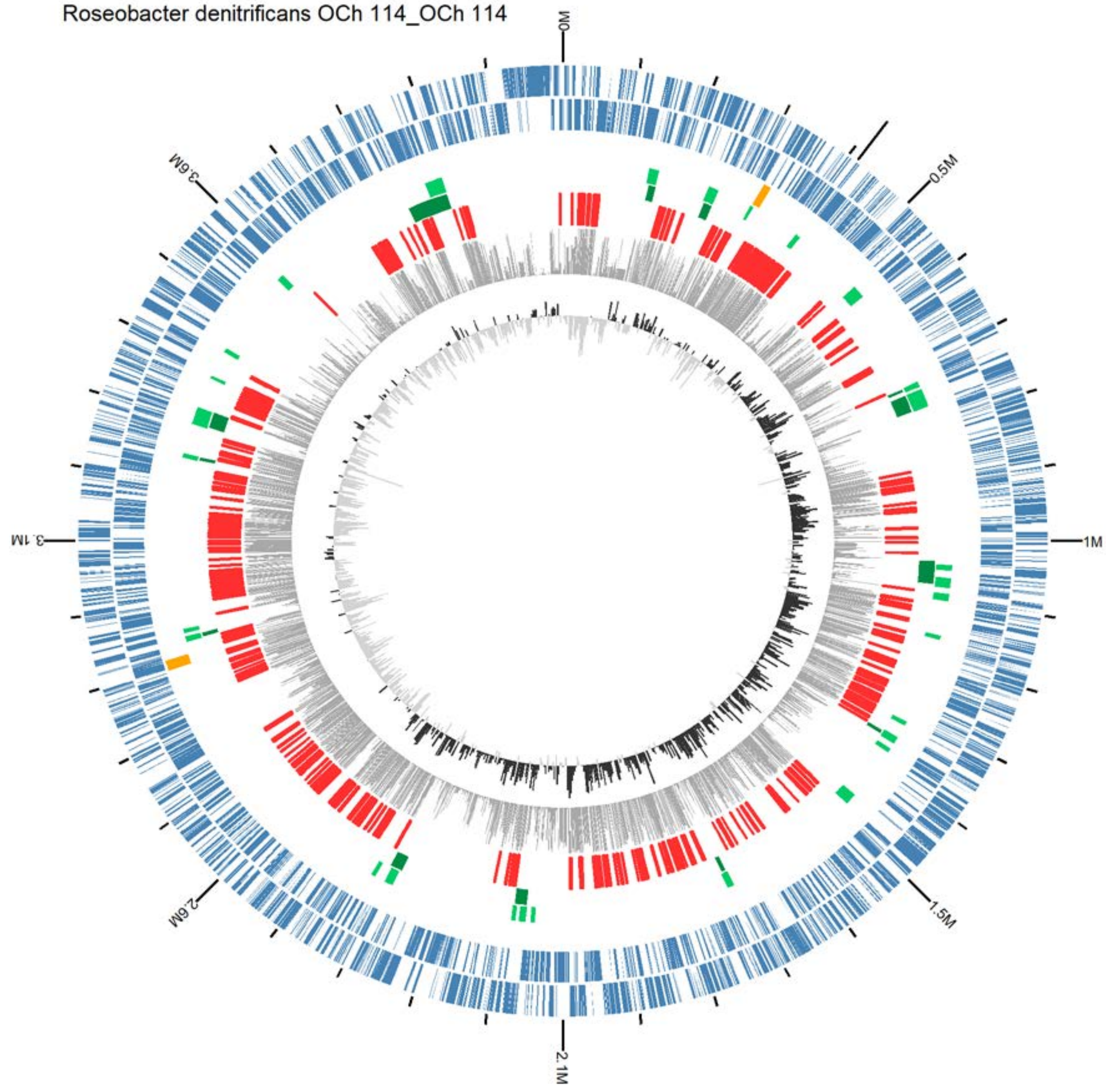
Roseobacter denitrificans OCh 114_OCh 114
0M
0.5M
1M
1.5M
2.1M
2.6M
3.1M
3.6M

Roseobacter denitrificans_FDAARGOS_309

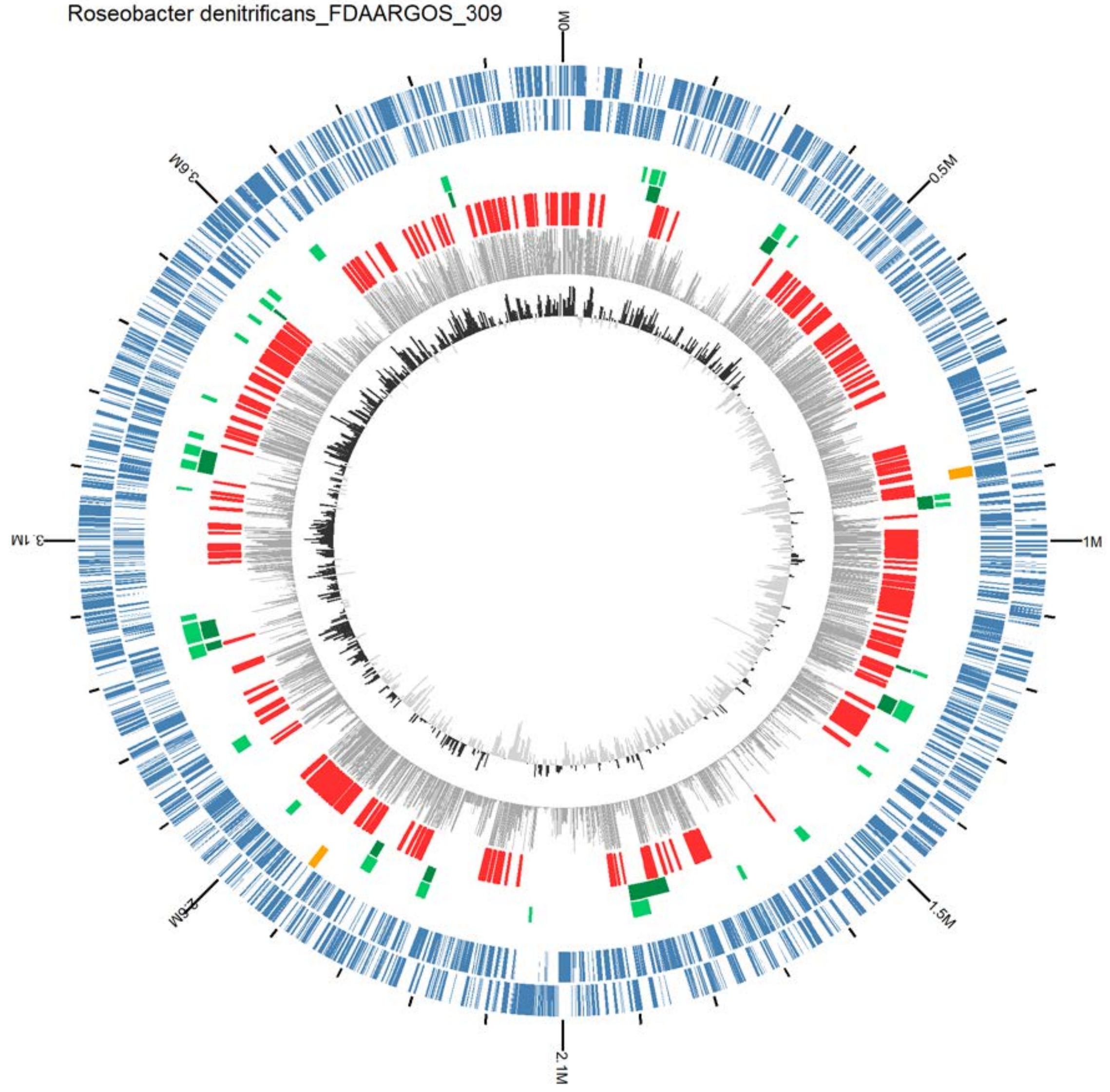

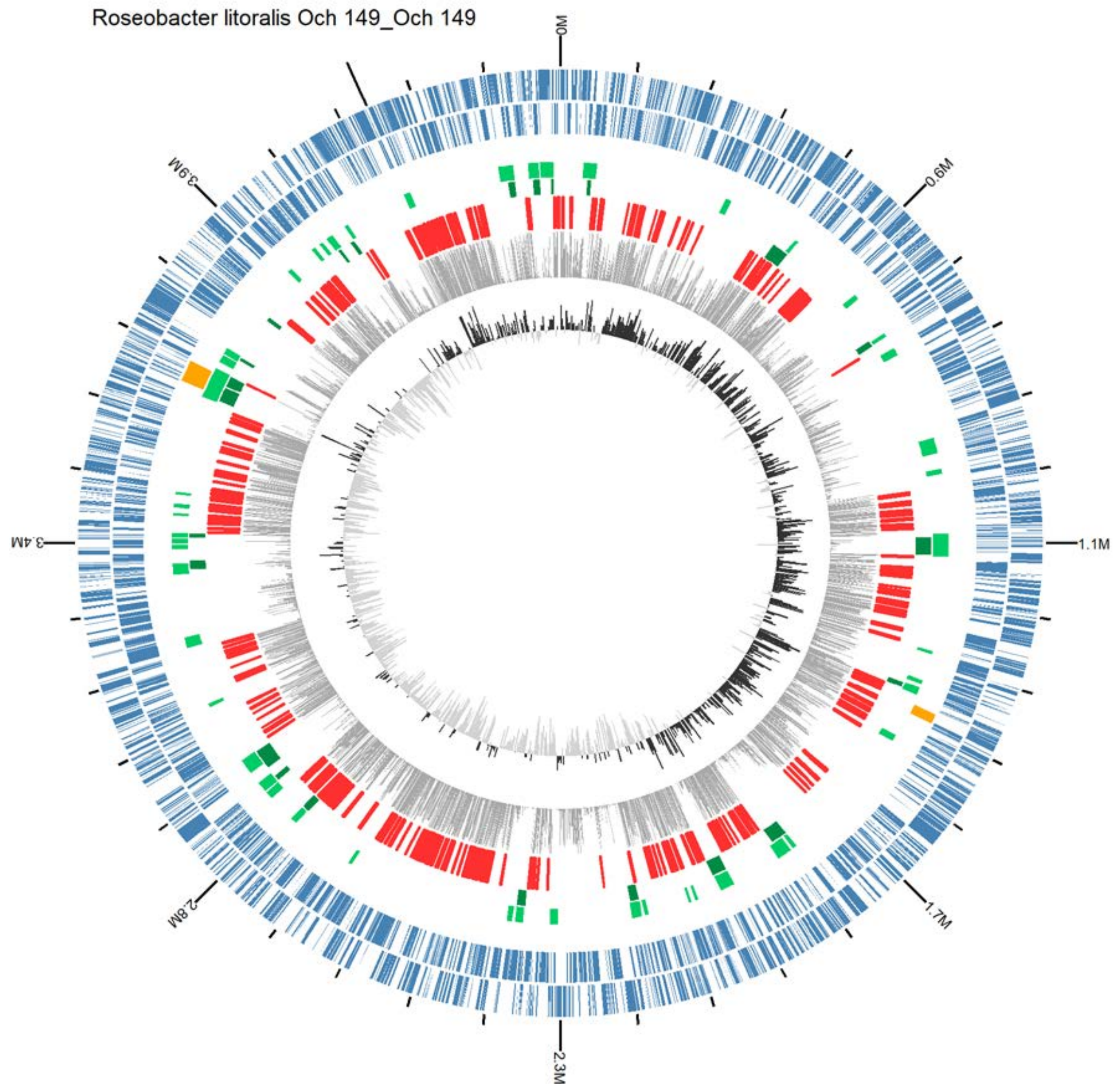
Roseobacter litoralis Och 149_Och 149
0M
0.6M
1.1M
1.7M
2.3M
2.8M
3.4M
3.9M

Roseovarius mucosus_SMR3

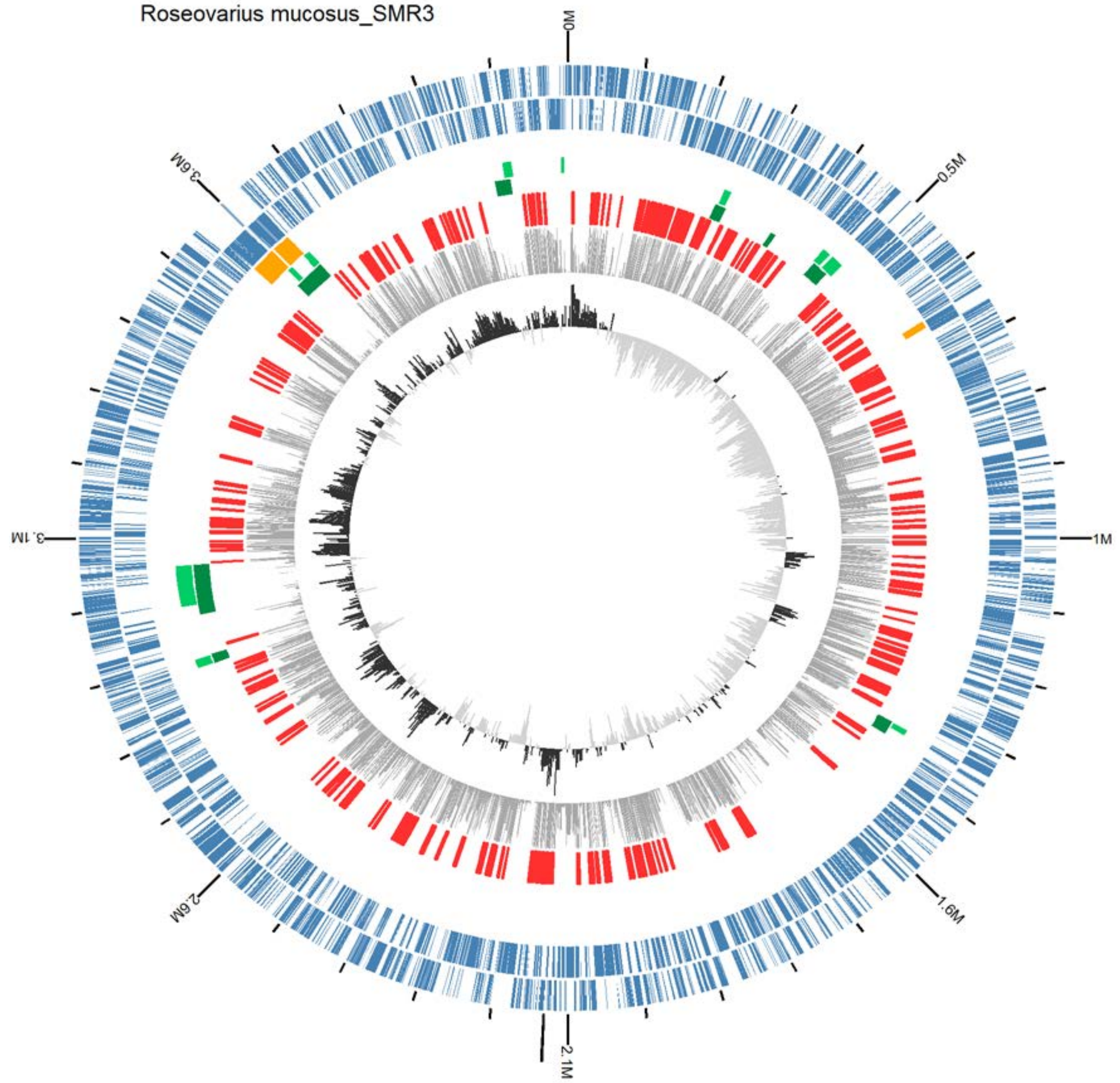

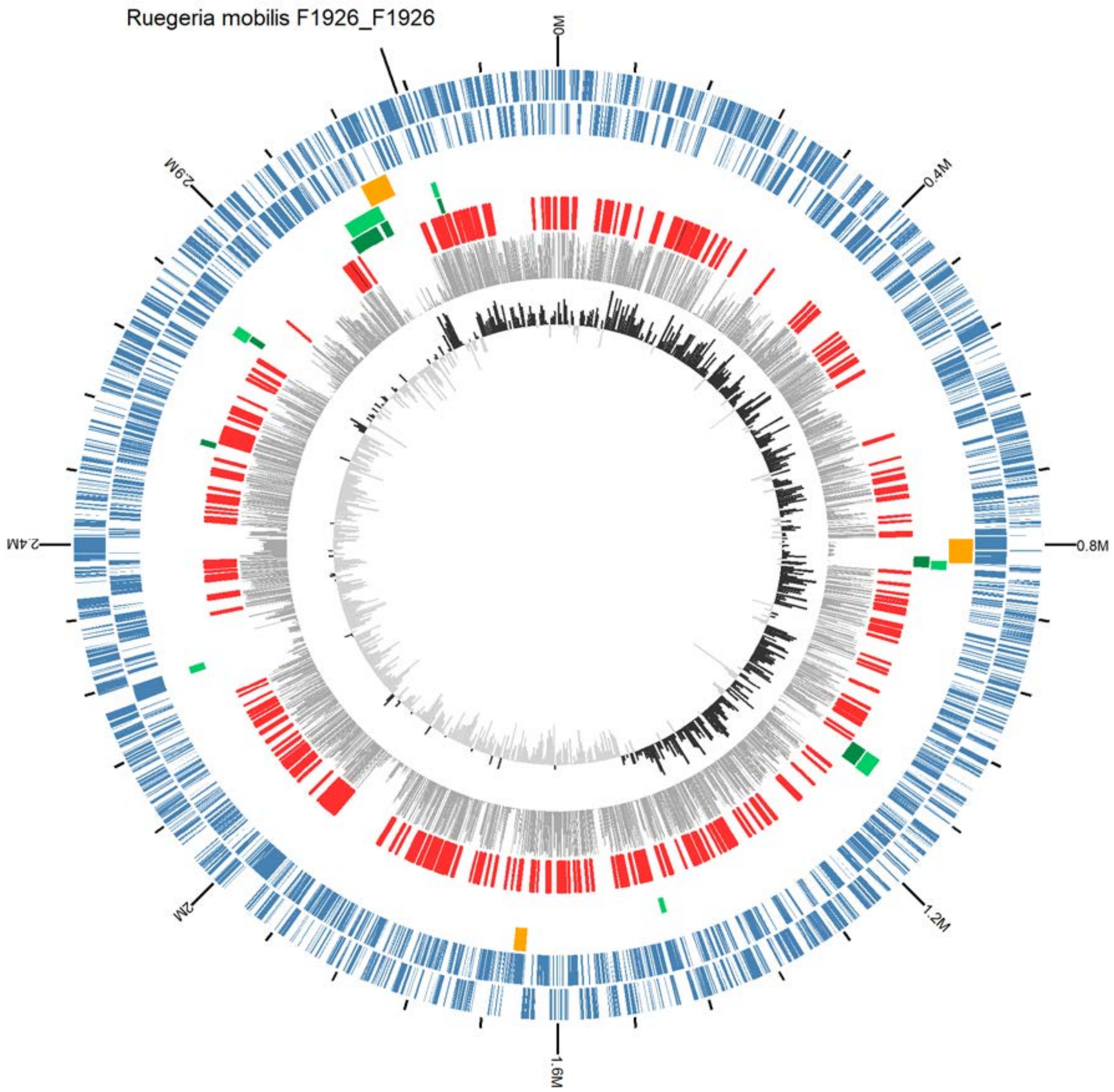
Ruegeria mobilis F1926_F1926
0M
0.4M
0.8M
1.2M
1.6M
2M
2.4M
2.9M

Ruegeria pomeroyi DSS-3_DSS-3

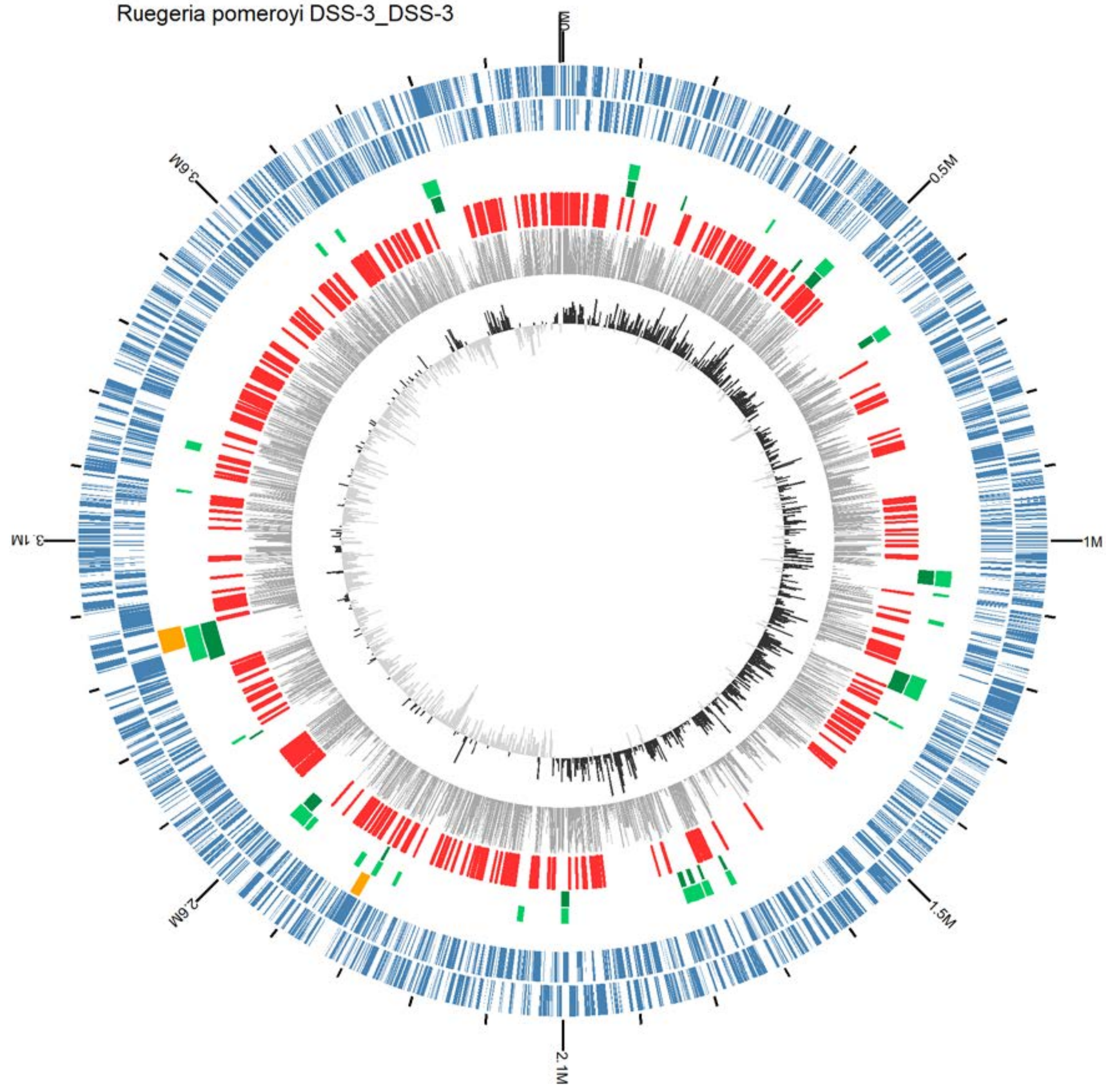

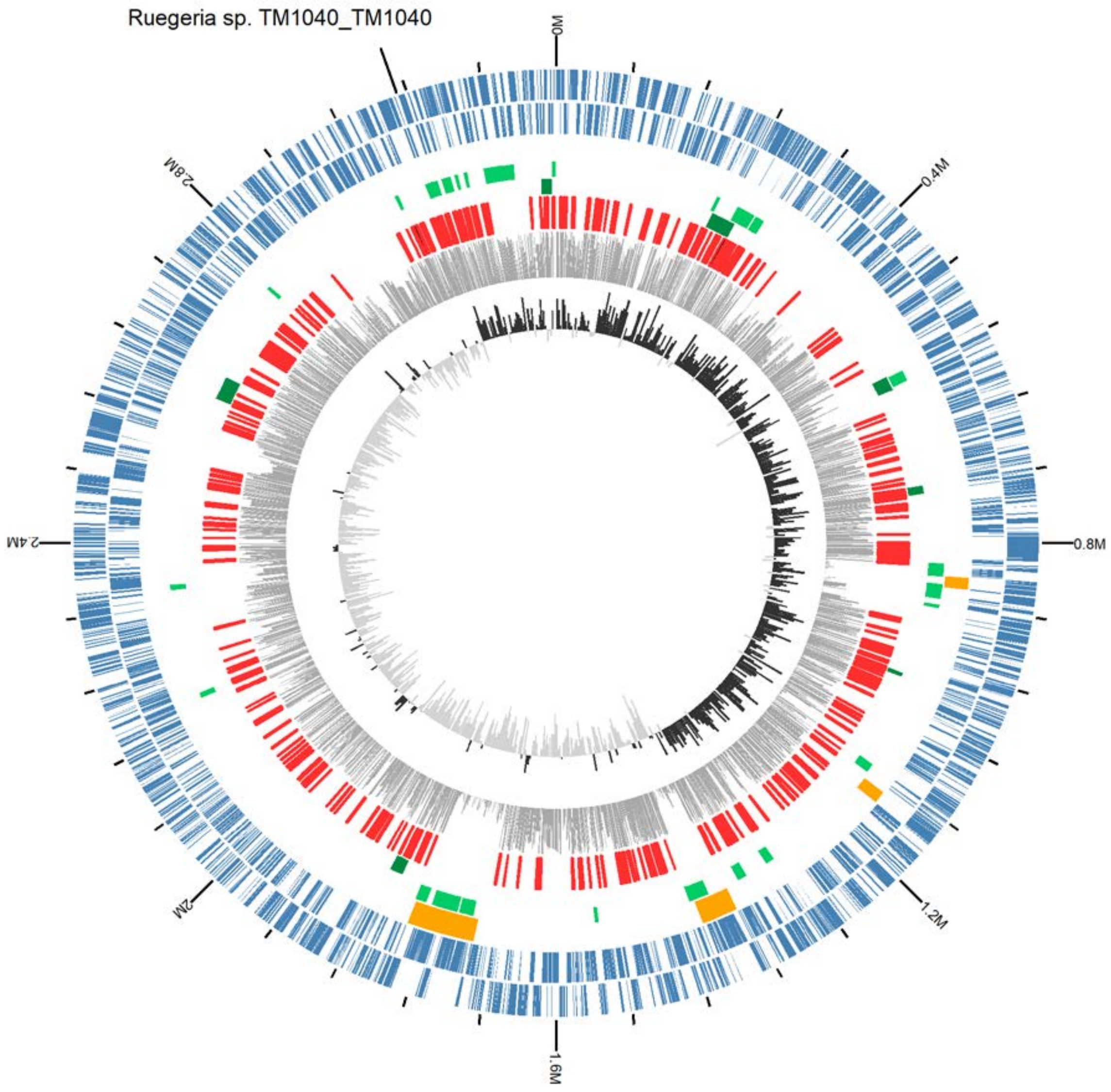
Ruegeria sp. TM1040_TM1040
0M
0.4M
0.8M
1.2M
1.6M
2M
2.4M
2.8M

Sagittula sp. P11_P11

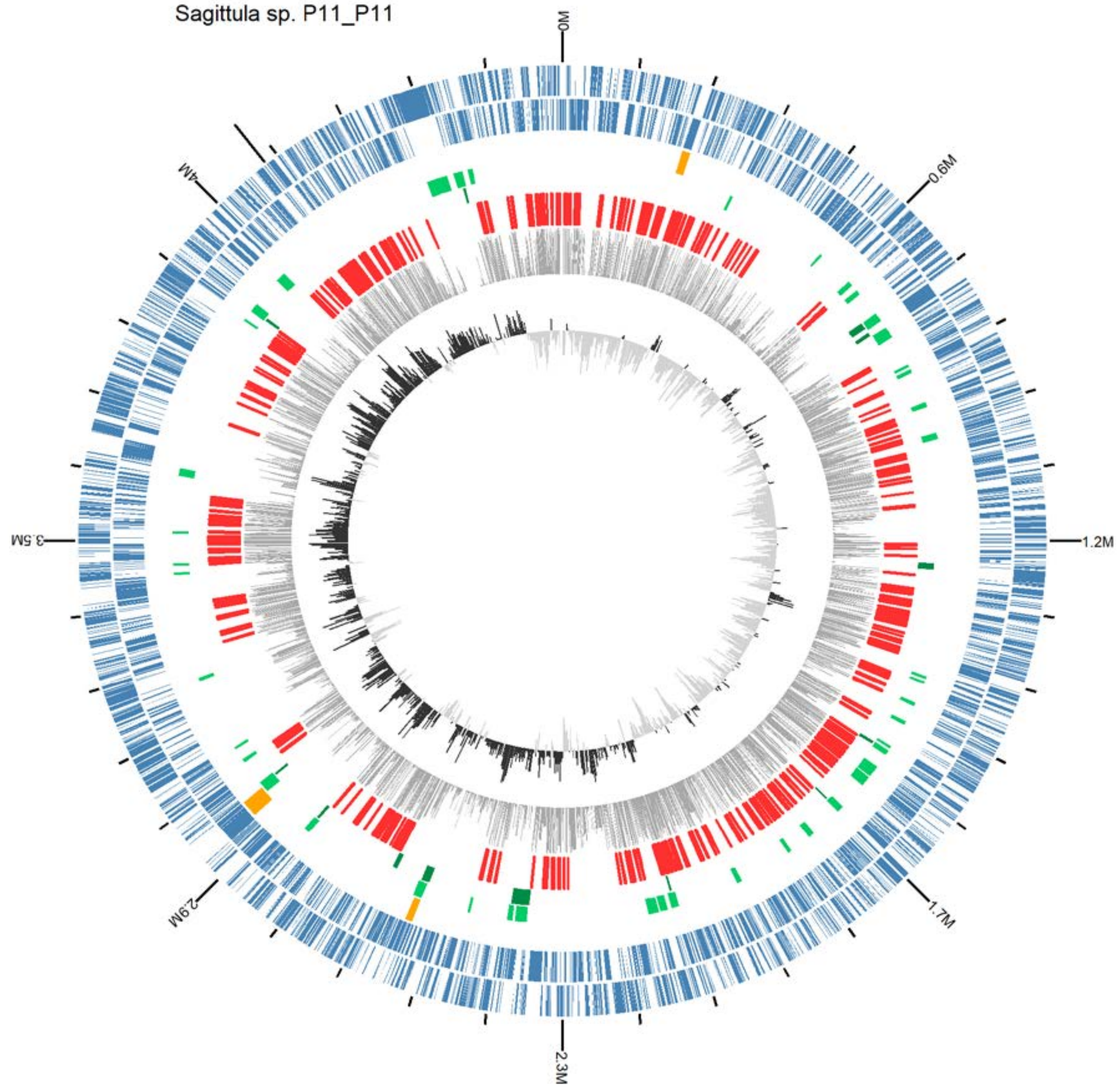

Sulfitobacter pseudonitzschiae_SMR1

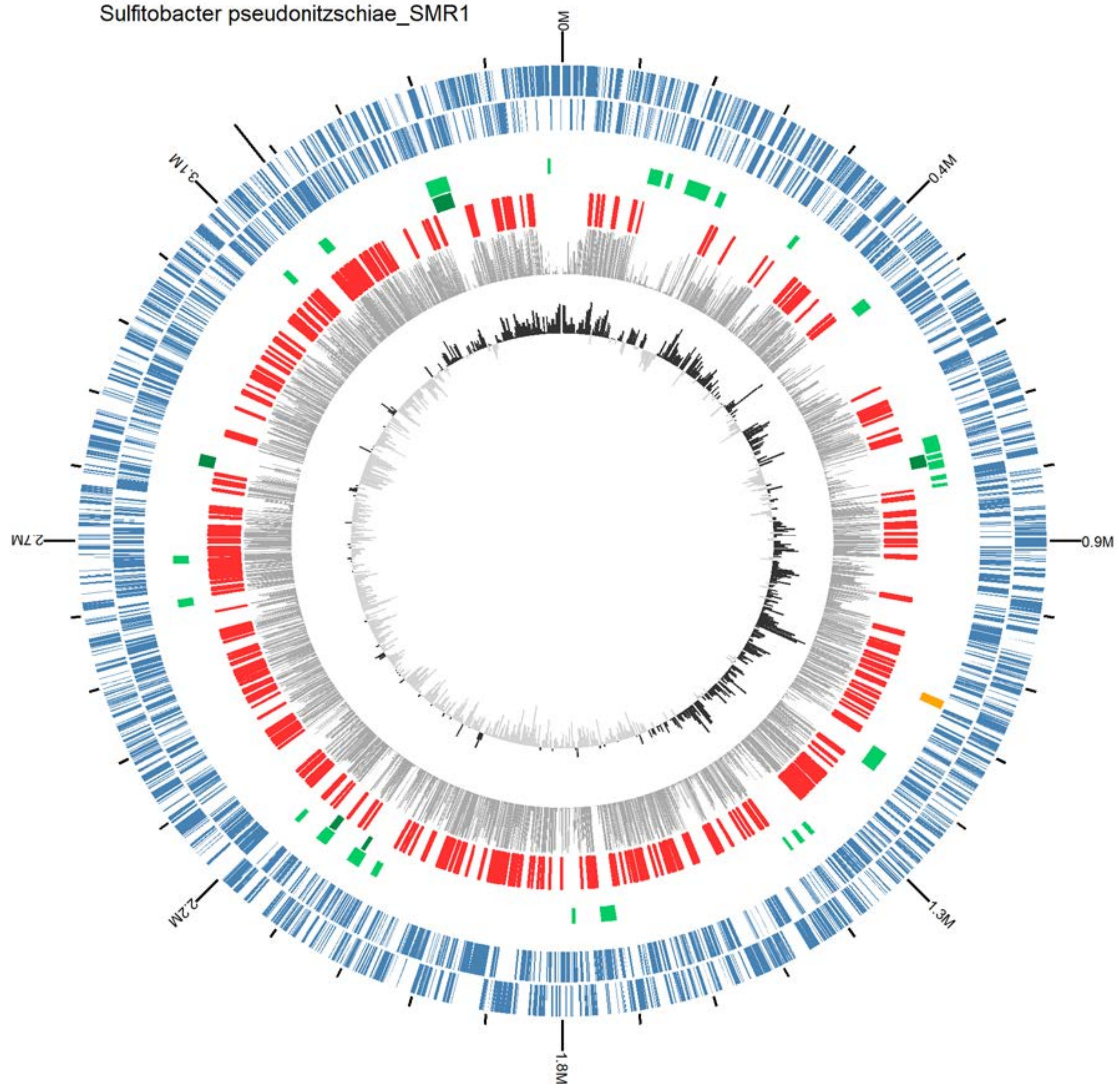

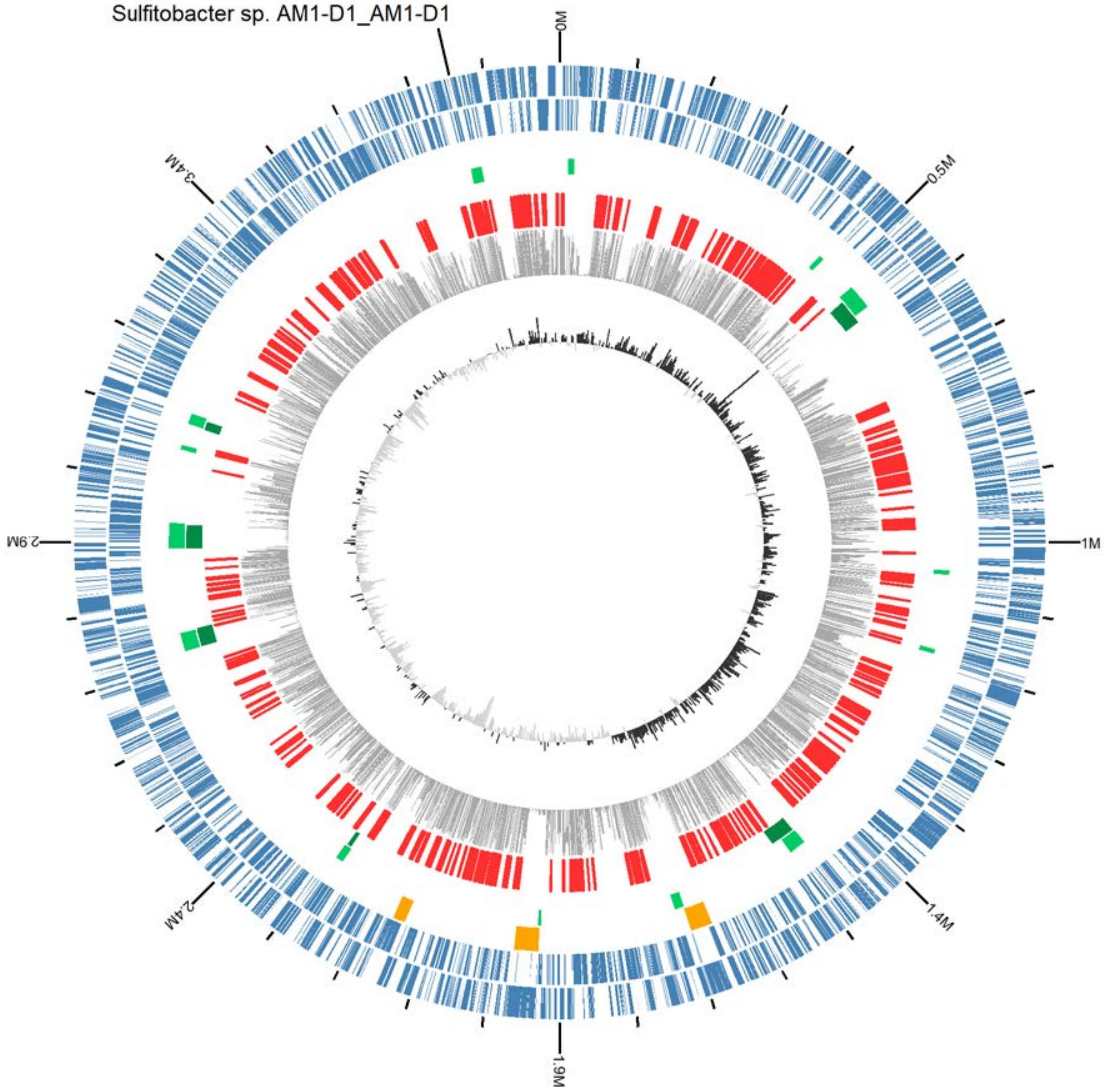

Sulfitobacter sp. AM1-D1_AM1-D1
0M
0.5M
1M
1.4M
1.9M
2.4M
2.9M
3.4M

Tateyamaria omphalii_DOK1-4

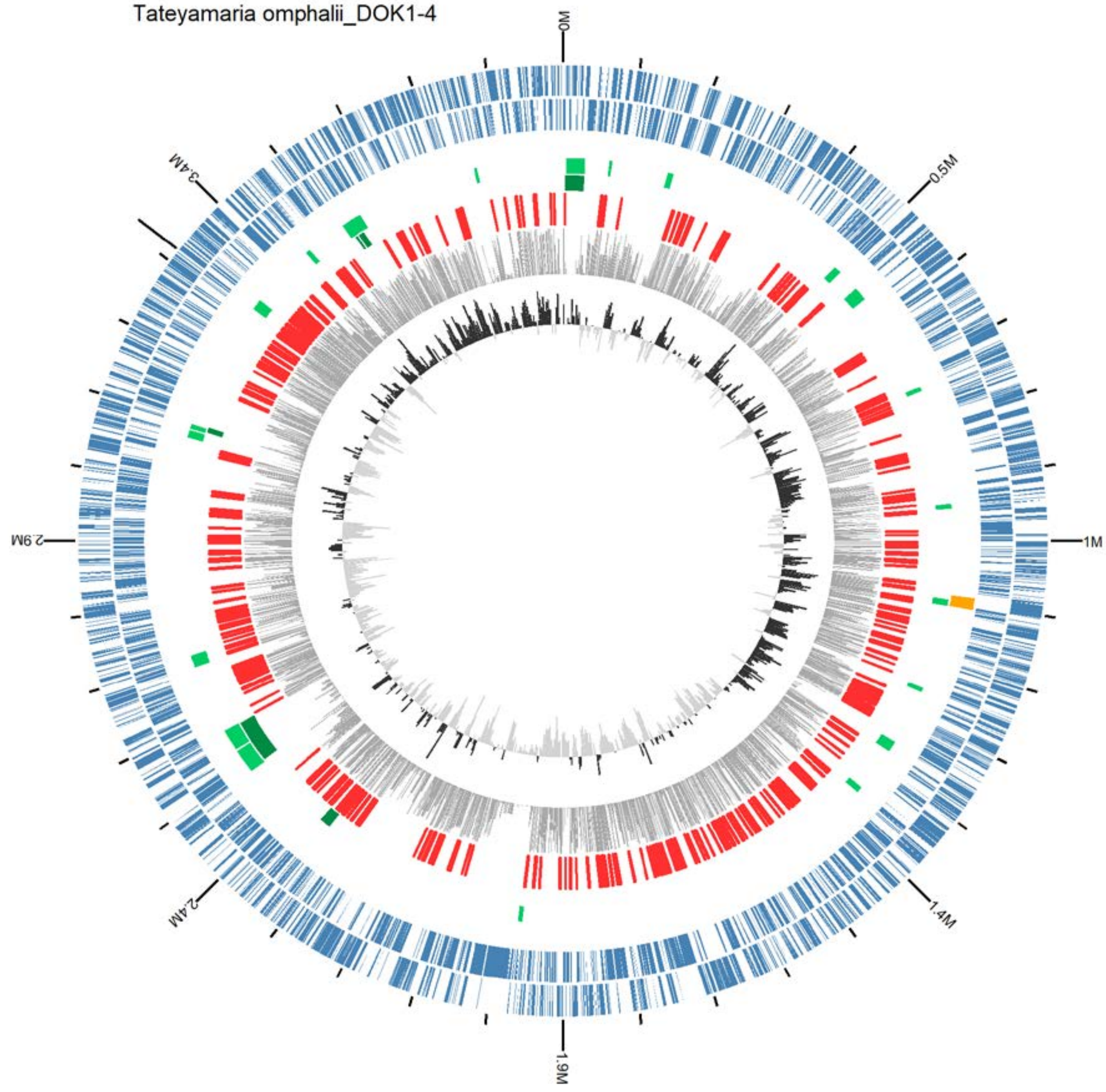

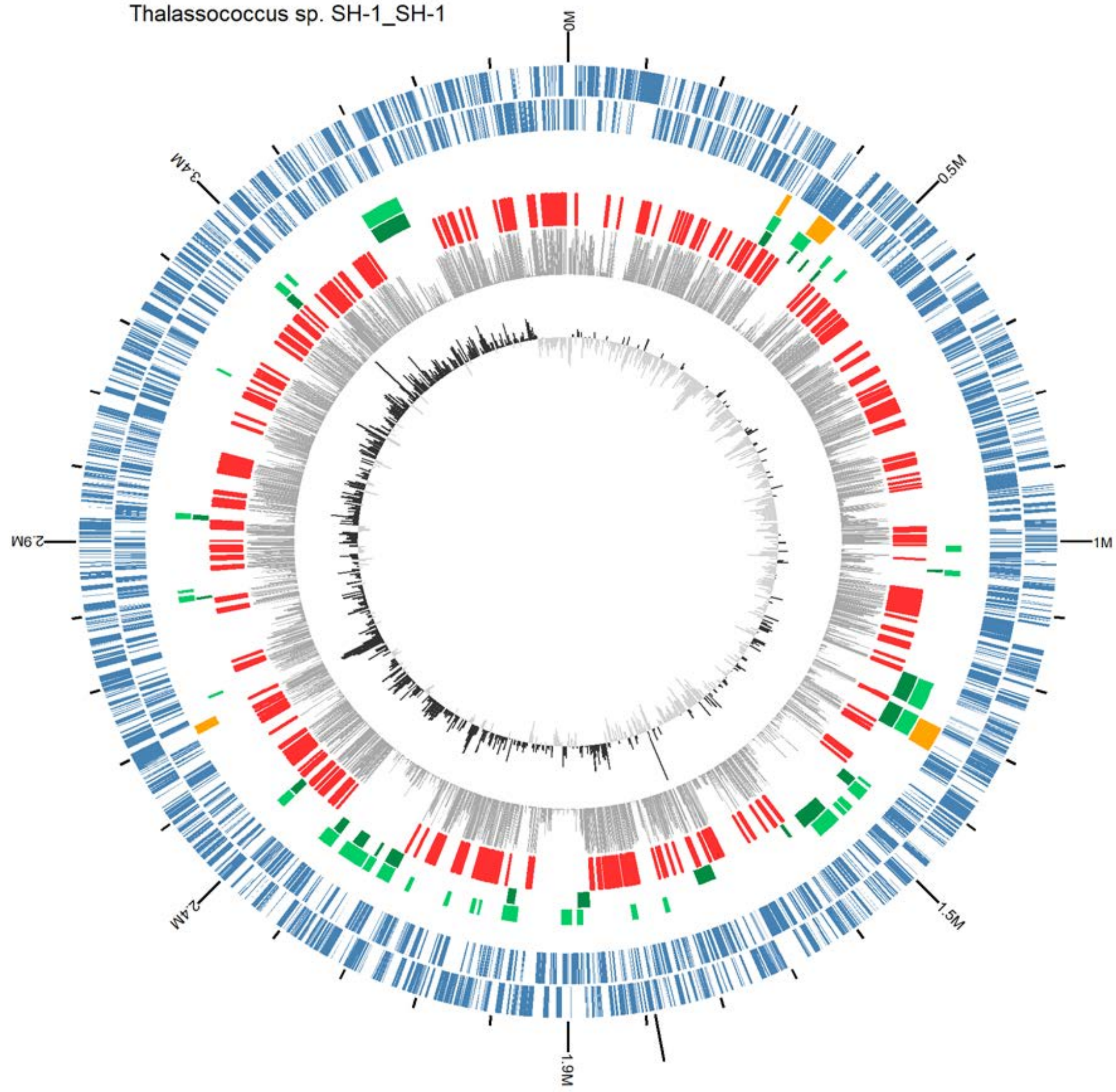
Thalassococcus sp. SH-1_SH-1
0M
0.5M
1M
1.5M
1.9M
2.4M
2.9M
3.4M

Thiobacimonas profunda_JLT2016

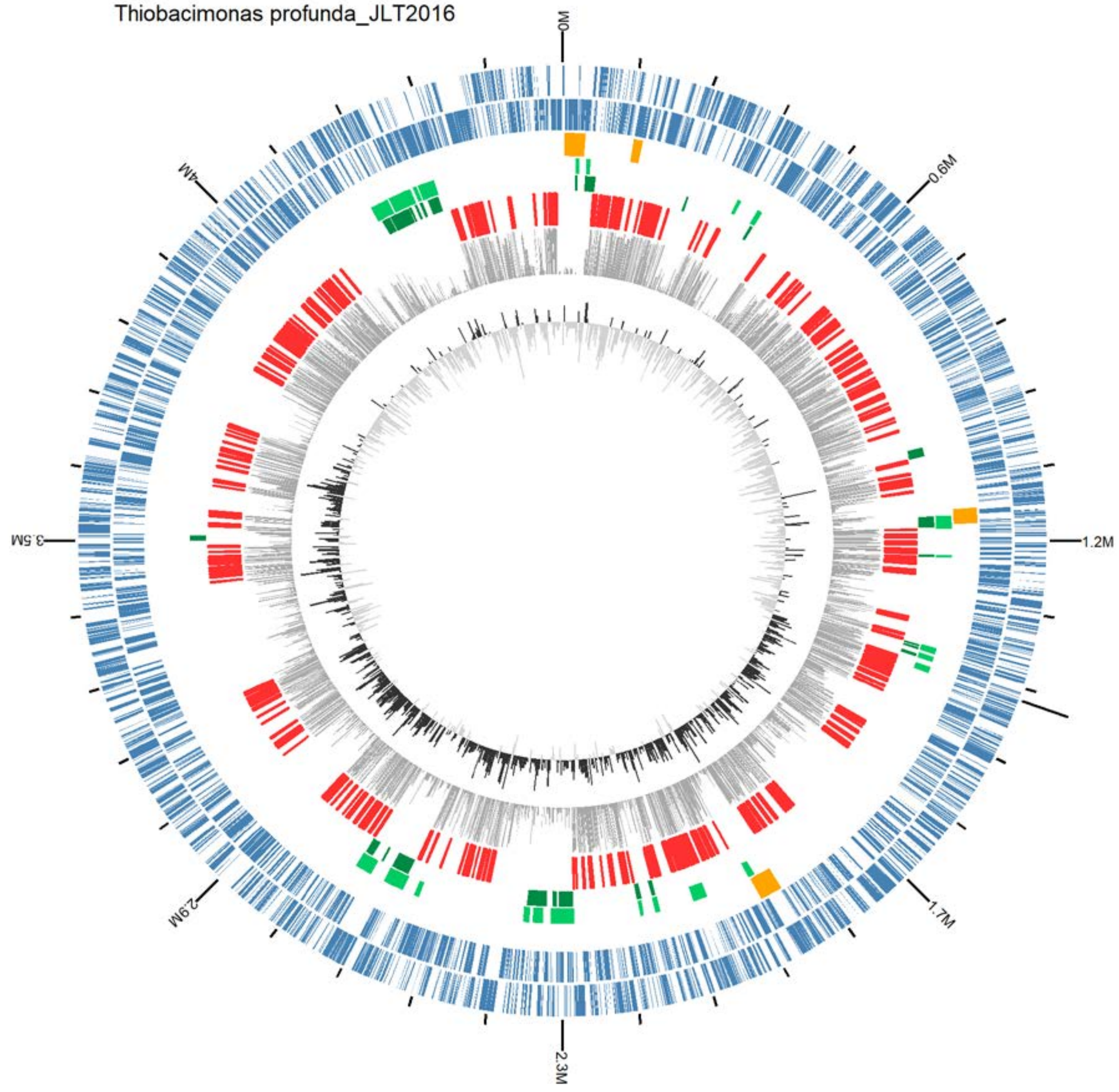

Thioclava nitratireducens_25B10_4

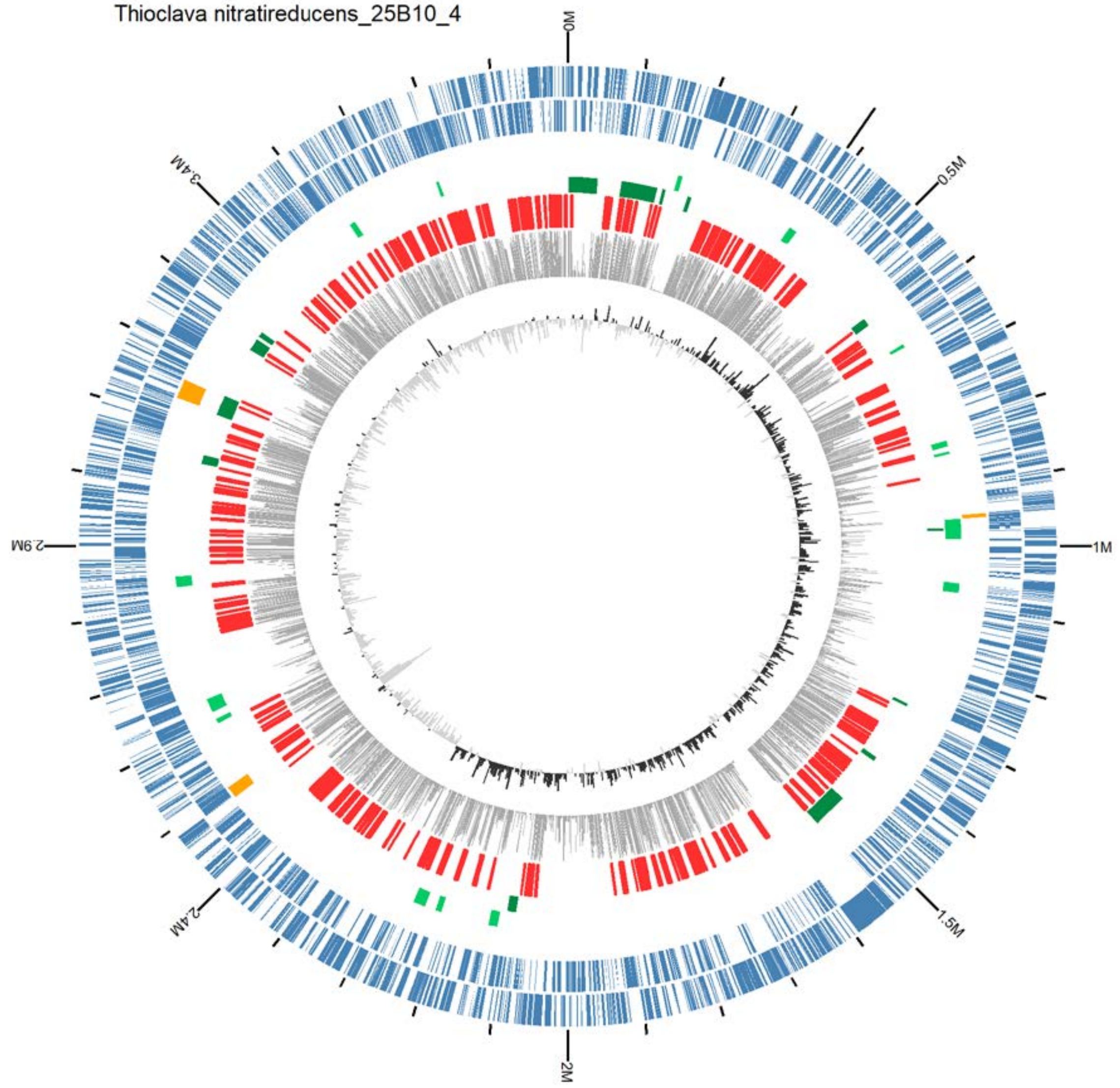

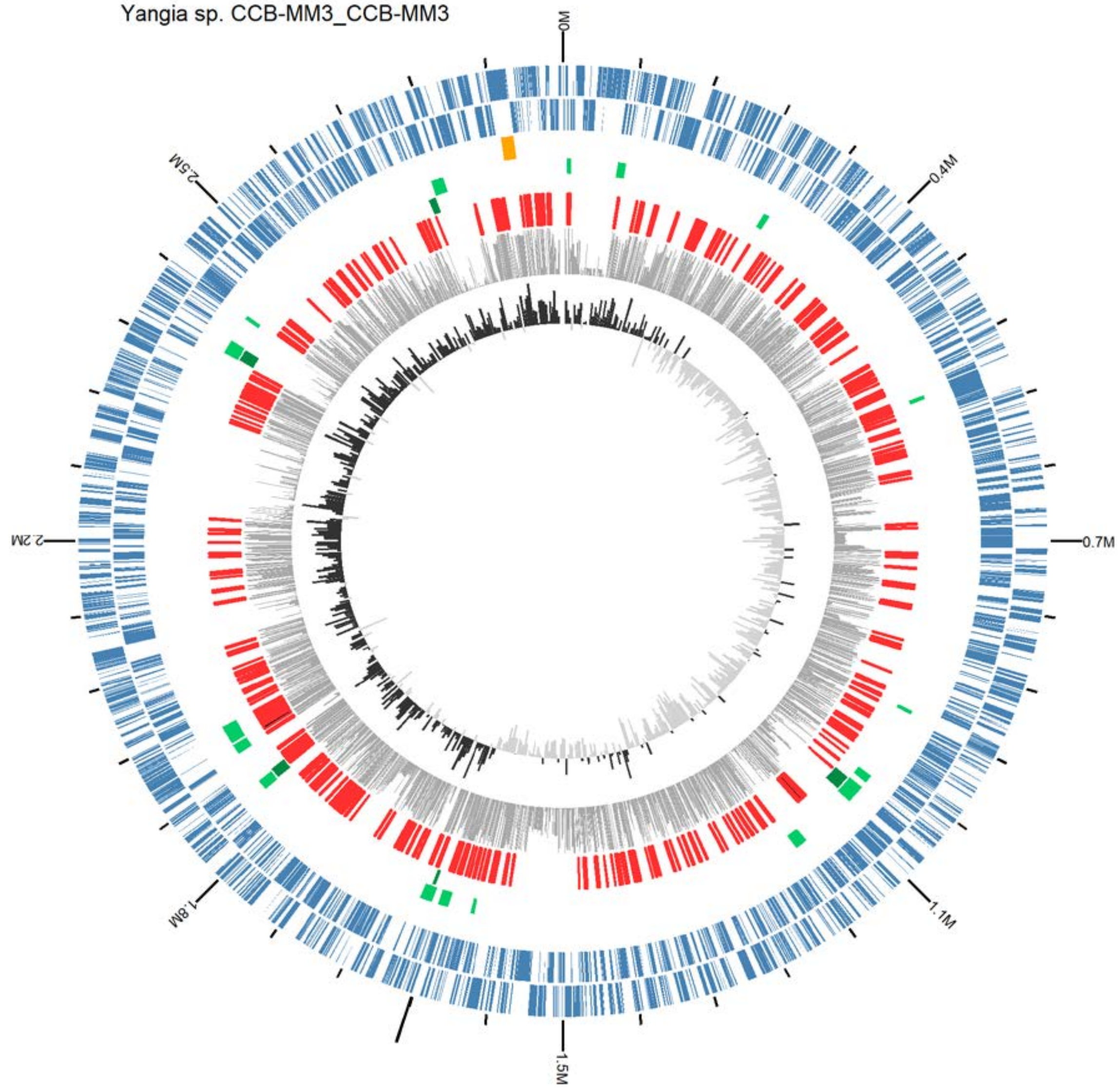
Yangia sp. CCB-MM3_CCB-MM3
0M
0.4M
0.7M
1.1M
1.5M
1.8M
2.2M
2.5M
